# Inhibitors of the Thioesterase Activity of *Mycobacterium tuberculosis* Pks13 Discovered Using DNA-Encoded Chemical Library Screening

**DOI:** 10.1021/acsinfecdis.3c00592

**Published:** 2024-04-05

**Authors:** Inna V. Krieger, Subbarao Yalamanchili, Paige Dickson, Curtis A. Engelhart, Matthew D Zimmerman, Jeremy Wood, Ethan Clary, Jasmine Nguyen, Natalie Thornton, Paolo A. Centrella, Betty Chan, John W Cuozzo, Martin Gengenbacher, Marie-Aude Guié, John P Guilinger, Corey Bienstock, Hajnalka Hartl, Christopher D. Hupp, Rachael Jetson, Takashi Satoh, John T. S. Yeoman, Ying Zhang, Veronique Dartois, Dirk Schnappinger, Anthony D. Keefe, James C. Sacchettini

**Affiliations:** 1 Department of Biochemistry & Biophysics, Texas A&M University, College Station, Texas 77843, United States; 2 X-Chem Inc., 100 Beaver Street, Waltham, Massachusetts 02453, United States; 3 Relay Therapeutics, 399 Binney Street, Cambridge, Massachusetts 02141, United States; 4 Ipsen Bioscience Inc., 1 Main Street, Cambridge, Massachusetts 02142, United States; 5 Valo Health, 75 Hayden Avenue, Lexington, Massachusetts 02141, United States; 6 EXO Therapeutics, 150 Cambridgepark Drive, suite 300, Cambridge, Massachusetts 02140, United States; 7 Department of Microbiology and Immunology, Weill Cornell Medicine, New York, New York 10021, United States; 8 Center for Discovery and Innovation, Hackensack Meridian Health, Nutley, New Jersey 07110, United States; 12 Hackensack Meridian School of Medicine, Hackensack Meridian Health, Nutley, New Jersey 07110, United States; 9 Orogen Therapeutics, 12 Gill Street, Woburn, Massachusetts 01801, United States; 10 Auron Therapeutics, 55 Chapel Street, Newton, Massachusetts 02458, United States; 11 Recludix Pharmaceuticals, 222 Third Street, Cambridge, Massachusetts 02142, United States

**Keywords:** DNA encoded chemical library, DEL, TB drug discovery, polyketide synthase 13, thioesterase, inhibitor

## Abstract

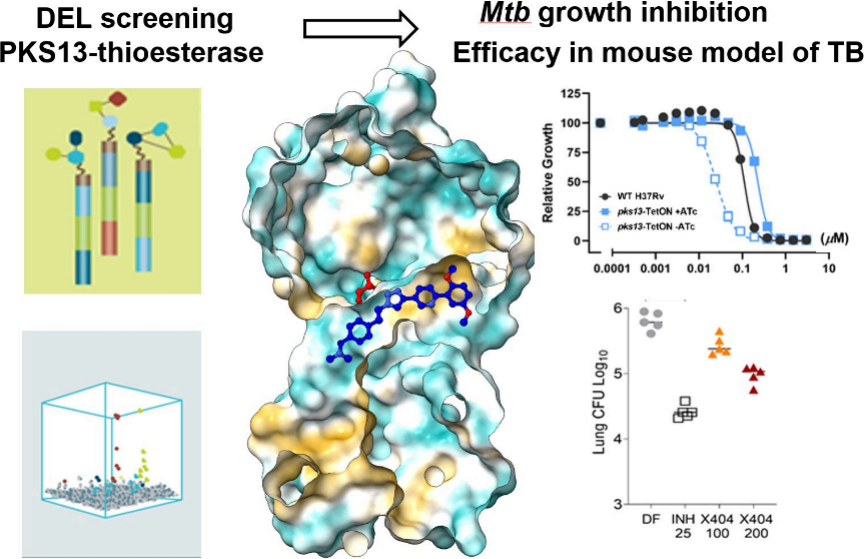

DNA-encoded chemical library (DEL) technology provides a time- and cost-efficient method to simultaneously screen billions of compounds for their affinity to a protein target of interest. Here we report its use to identify a novel chemical series of inhibitors of the thioesterase activity of polyketide synthase 13 (Pks13) from *Mycobacterium tuberculosis* (Mtb). We present three chemically distinct series of inhibitors along with their enzymatic and Mtb whole cell potency, the measure of on-target activity in cells, and the crystal structures of inhibitor-enzyme complexes illuminating their interactions with the active site of the enzyme. One of these inhibitors showed a favorable pharmacokinetic profile and demonstrated efficacy in an acute mouse model of tuberculosis (TB) infection. These findings and assay developments will aid in the advancement of TB drug discovery.

Tuberculosis (TB) is a significant global health challenge, causing considerable morbidity and mortality worldwide. In 2022 tuberculosis was the leading cause of death from infectious diseases resulting in approximately 1.3 million deaths, an increase compared to the previous years’ declining trend. TB ranks among the top ten causes of death worldwide, underscoring its severe impact on public health. This ongoing burden of the disease and its spread across different regions and populations is primarily attributed to the long and demanding treatment regime and widespread drug resistance. For a durable cure, standard TB treatment usually lasts for six months, while treatment for drug-resistant TB can extend to 9–20 months and has a high relapse rate.

Despite the availability of treatments that lead to a cure for most patients, the rise of multidrug-resistant TB (MDR-TB) and extensively drug-resistant TB (XDR-TB) has added a new layer of urgency to the situation. A recent study indicated that 7.5% of infected individuals tested were resistant to the first-line drug rifampicin.^1^ In addition, there was a 37% increase in diagnosed drug resistant cases documented in 2021 compared to 2020 (WHO). The rapid spread of drug-resistant *Mycobacterium tuberculosis* (Mtb), the causative agent of TB, is a stark reminder of the pressing need to repopulate the TB drug discovery pipeline with promising leads. Without a steady stream of new antibiotics, we risk losing our ability to effectively treat not only TB but also a wide range of other infectious diseases.

While there are many approaches used to identify starting points for structure-guided drug discovery, DNA-encoded chemical library screening (DEL) has emerged over the past decade as a revolutionary technology in the field of drug discovery.^2,3^ It offers a highly efficient, rapid, and cost-effective approach to screening billions of small molecules for their ability to bind to a specific target.^4^ Each molecule in the library is labeled with a unique oligonucleotide barcode, which serves as a record of the chemical building blocks and chemical scheme used to create it.^3^ This barcode enables the simultaneous testing of billions of molecules in a single screening event. The enrichment of library molecules binding to a protein target is achieved through affinity-mediated selection, followed by amplification of the encoding oligonucleotide sequence. Enriched library members can then be identified by sequencing the DNA barcodes, providing a direct readout of the molecular structure.

One of the significant advantages of DEL screening is its technical simplicity, which allows several screening variations to be performed in parallel in a single experiment. Indeed, information on the selectivity, binding site, and relative affinity of enriched library members can be accessed by counterscreening against mutants or orthologs, competitive screens in the presence of known substrates or inhibitors, or target concentration variation. This information can be used to prioritize the synthesis of enriched compounds based on their mode of action, selectivity, affinity, and structure activity relationship (SAR) information all of which are accessed directly from the screening output.

High efficiency and an ability to run selections in parallel allows for the rapid screening of large sets of targets for their ligandability as was demonstrated for 119 targets from *Acinetobacter baumannii* and *Staphylococcus aureus,* and for 42 targets from *Mycobacterium tuberculosis* (Mtb) by Machutta et al.^5^ There are also prior reports of successful DEL Hit ID screens having been conducted against Mtb targets. Encinas et al.^6^ report the discovery of one novel series of inhibitors to the Mtb enoyl–acyl-carrier protein (ACP) reductase, InhA, a validated target for the treatment of tuberculosis and the target for the approved drug isoniazid. A range of analogues were synthesized, but *in vivo* efficacy was not achieved. Subsequently, Soutter et al.^7^ reported the discovery of several novel inhibitors to the same target. All series were shown to have activity in cellular assays, and X-ray crystallography shows that they bind close to NADH and exhibit a range of binding modes including some previously unreported.

In this study, the thioesterase domain of Mtb polyketide synthase 13 (Pks13-TE) was chosen as a target for the DEL screening campaign. Mycobacteria, including Mtb, utilize specialized lipids called mycolic acids to form an additional protective layer in their cell wall.^8^ The mycolic acid biosynthetic pathway^9^ has proven to be a great source of Mtb drug targets. For example, the enzyme InhA, which is enoyl ACP reductase, is involved in synthesizing the fatty acyl precursors of mycolic acids and is the target of two approved drugs, isoniazid and ethionamide.^10,11^ Pks13 is a multidomain enzyme that performs the final step in the synthesis of mycolic acids through the condensation of a long-chain fatty acid (C24–C26) and a very long-chain fatty acid (C56).^12^ The product is transferred from Pks13 to trehalose by the Pks13-TE domain.^13^ Subsequently, trehalose monomycolate is transported across the membrane and used to build the outer layer of the cell wall. Pks13 has been shown as essential for viability of Mtb by multiple genetic experiments in vitro^14−16^ and in vivo.^17^

Pks13-TE is a validated target for the development of antibiotics for Mtb.^18−21^ In previous work, we identified TAM16, a potent lead molecule for Pks13-TE. TAM16 demonstrated outstanding efficacy in a mouse model of tuberculosis comparable to that of the first-line drug isoniazid. However, further development of these benzofuran inhibitors was impeded by their associated hERG toxicity.^22^ This work fueled a search for novel chemotypes that better target this critical enzyme.^23−25^ In addition, an inhibitor series based on a thiophene core with potent activity against mycobacteria was found to target the N-terminal acyl carrier protein domain of Pks13 enzyme.^26−28^ A screening campaign on Pks13-TE resulted in the discovery of an oxadiazole series with potent antimycobacterial activity, but the PK properties of these molecules proved to be insufficient for in vivo evaluation.^29^ Despite efforts by multiple laboratories, no new progressable lead has been reported to date. This is in part due to the challenges in assaying Pks13, as an assay using the natural mycolic acid substrate is not practical. The primary method that has been used to measure the Pks13-TE esterase activity employs a fluorescent substrate, 4-methylumbelliferyl heptanoate (4-MUH) with the 4-MUH linked to a C7 fatty acid via an ester bond that produces a fluorescence signal when cleaved by Pks13-TE.^18^ 4-MUH is a poor substrate for Pks13-TE. It is converted at a slow rate and generates a weak signal. In addition, the ester bond is not stable and undergoes hydrolysis in the solution. As a result, the 4-MUH-based assay has a poor signal-to-noise ratio and is challenging to use as a high-throughput screening (HTS) assay.

To find novel chemical inhibitors without relying on the 4-MUH-based assay, we used a DEL screen to select for molecules that would bind to the purified unliganded Pks13-TE and that were competitive with TAM16. This strategy permitted us to identify molecules that would likely bind at the active site of Pks13-TE. The hits were confirmed using a TAMRA activity probe binding displacement assay instead of the 4-MUH esterase assay. The most potent hits were expanded into chemical series in order to develop structure–activity relationships (SAR). Active inhibitors were tested for Mtb growth inhibition, and the most potent were evaluated for on-target activity, X-ray crystallography, and pharmacokinetics (PK). Our lead compound, X20404, showed efficacy in a model of acute TB, significantly reducing bacterial burden in lungs of infected mice.

## Results

### Protein Quality Assessment and Screening Conditions Optimization

Prior to implementing the DEL screen, the purified recombinant Pks13-TE domain with a 6 histidine affinity tag at the N terminus was characterized by multiple biophysical and biochemical methods to verify purity, integrity, and functional state (Figure S1). We used size-exclusion chromatography (SEC) to confirm homogeneity and the expected molecular weight (Figure S1B). The protein was monodisperse, and no aggregates were detected by SEC, which was further corroborated by Dynamic Light Scattering (DLS) (Figure S1A). Detergent, salts, and carrier DNA are required in selection buffers to reduce nonspecific binding. Immobilized Pks13-TE was active in the original assay buffer (Figure S1C) and in an alternative buffer optimized for DEL screening (Figure S1D). Pks13-TE retained its enzyme activity in the presence of salts and DNA, but a variety of detergents were found to inhibit the enzyme. Optimization efforts identified CHAPS as the preferred selection buffer detergent (Figure S1E). Additional experiments revealed that Pks13-TE activity was also inhibited in the presence of the Ni-NTA affinity matrix, and the substrate 5-MUH was autohydrolyzed in the presence of imidazole (Figure S1F,G). These results helped inform the final screening conditions, which were performed on His-cOmplete affinity matrix in an imidazole-free buffer. To confirm that Pks13-TE was functional under screening conditions, the catalytic activity of the immobilized enzyme was measured in the selection buffer using an activity assay.^18^ Substrate hydrolysis was observed to be time-dependent, demonstrating that Pks13-TE was indeed active while immobilized on His-cOmplete resin in selection buffer (Figure S1H) and further that it was inhibited by the control compound TAM16, as expected.

### DNA-Encoded Chemical Library Screening Using Affinity-Mediated Selection

**Figure 1:**
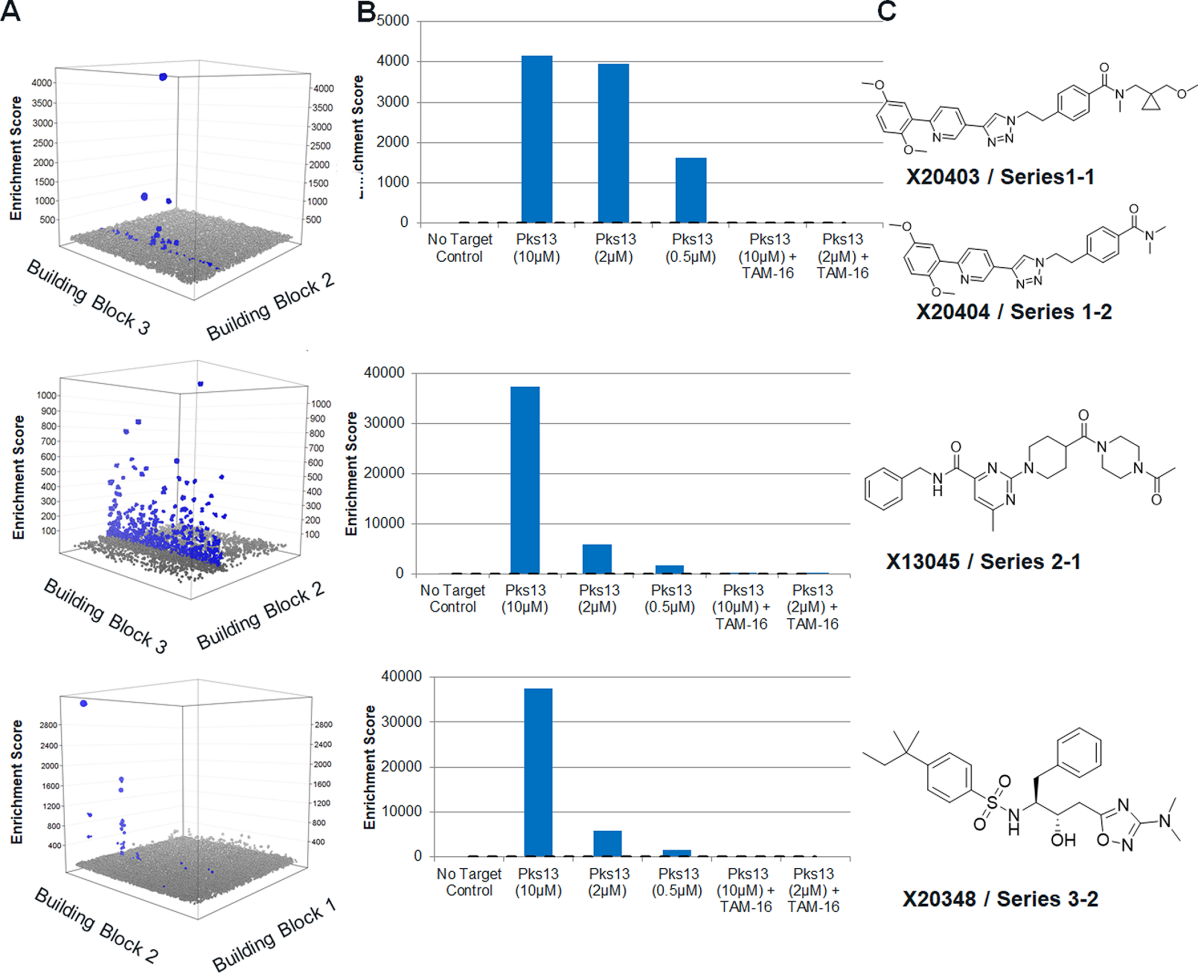
Selection profiles and output data used to discover individual DNA-Encoded Chemical Library Hits that defined Series 1, 2, and 3. (A) Enrichment data for building block combinations within each of the three libraries from which Series 1, 2, and 3 were derived. (B) The enrichment of indicated compounds across all selections. (C) The structure of relevant representative compounds for each of three series.

Fifty-nine DNA-encoded chemical libraries, representing about 125 billion unique compounds, were combined and then used for parallel screening of Pks13-TE at three protein concentrations of 0.5, 2, and 10 μM. These included the target protein Pks13-TE alone, Pks13-TE precomplexed with a known inhibitor TAM16, and a no-target control (Figure 1B, Table S1). After preincubation in solution in the absence or in the presence of TAM16, Pks13-TE was captured using the IMAC affinity matrix His-cOmplete, incubated with the library, followed by stringent washing, and heat-elution of retained library members. A second round of selection was performed using the output of the first round and fresh protein. The selection output was then PCR-amplified and sequenced using an Illumina HiSeq in high-output mode.

Sequenced barcodes were translated into chemical structures, and the statistical significance of each building block combination was calculated for every selection condition. Enriched library members were then clustered into families based on structural similarity. To prioritize chemical series for synthesis without attached DNA, several factors were considered, including clarity of the SAR within each cluster and the calculated physicochemical properties of exemplar compounds. Compounds prioritized for synthesis showed significant enrichment across all three concentrations of Pks13-TE but were not enriched in the selection condition where the protein was preincubated with TAM16 (50 μM) (Figure 1B, Table S2). Visualizations of the DEL screen output data as cube plots are shown in Figure 1A for each hit series, depicting the statistically significant enrichment of individual library members, along with histograms indicating the extent to which these compounds were enriched in each selection condition.

We then synthesized 80 compounds without DNA tags representing a total of fifty-nine clusters prioritized based on their selection profiles. These compounds were initially tested for enzyme inhibition using the 4-MUH-based assay.^18^ This assay was very challenging, as described above, which prompted us to develop an alternative approach to test the analogues using a competitive binding assay with a TAMRA fluorophore activity-based probe. This TAMRA probe has been typically used to label the serine side chain of serine hydrolases.^30,31^ It is composed of a TAMRA fluorophore attached to a C10 hydrocarbon linker followed by a fluorophosphonate, a catalytic serine specific reactive group, which we found binds to the Pks13-TE active site. Building on the literature of the successful use of fluorescence polarization-based assays,^32−34^ we adapted this probe for a competition assay to measure inhibitor affinities for the active site of Pks13-TE. There was good correlation in the rank order of IC_50_ values between the two assays in the cross-tested subset. Of 80 synthesized and assayed hits from the DEL screen, 30 were determined to be active in the biochemical assay (<50 μM) with 18 exhibiting IC_50_ values of less than 20 μM. These were clustered into 5 series.

Based on their ability to potently inhibit Pks13-TE, series 1–3 (representative compounds are shown in Figure 1C) were the focus of medicinal chemistry efforts to improve potency and develop an initial SAR.

**Figure 2:**
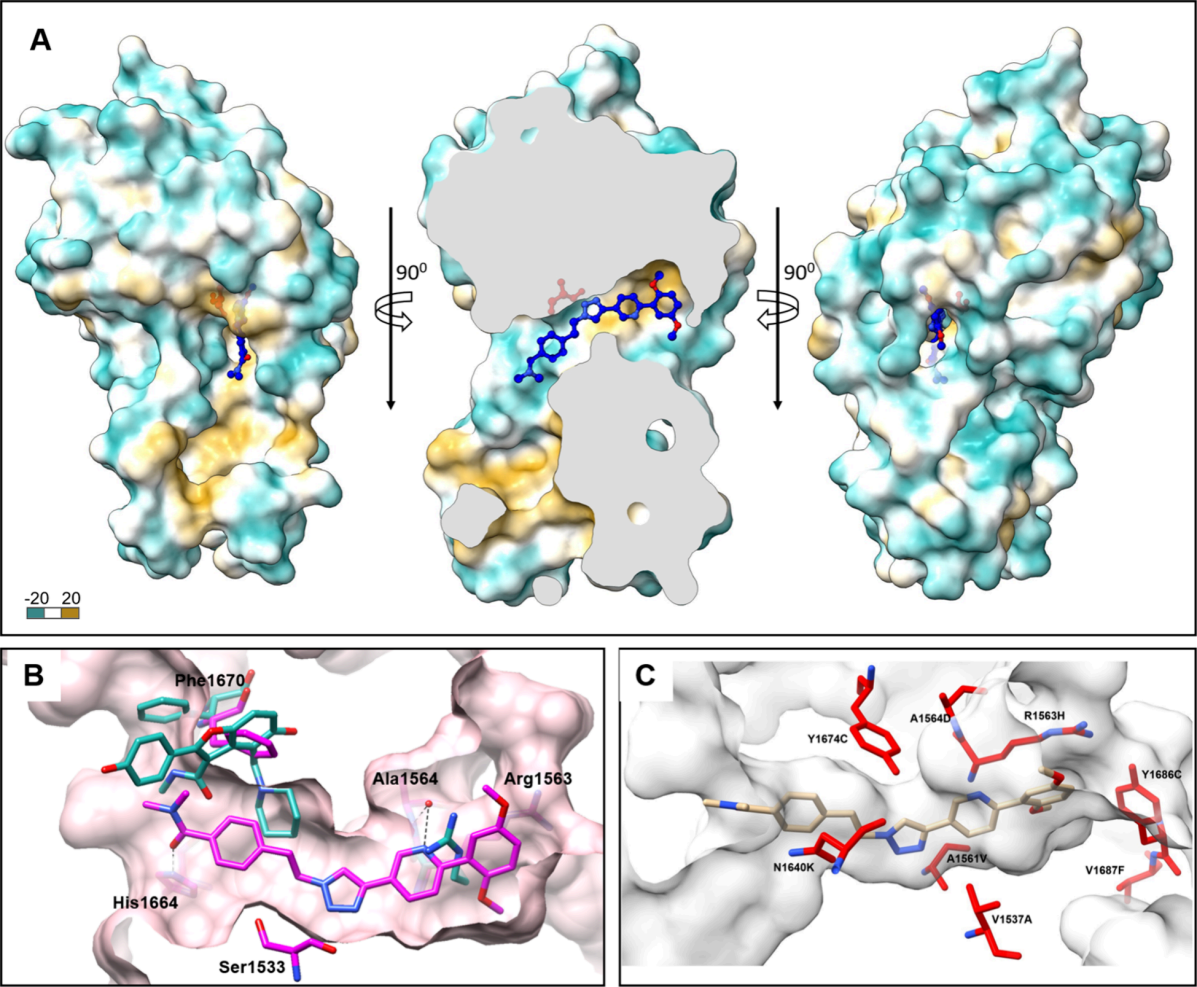
Binding mode of X20404 to Pks13-TE. (A) Surface view of Pks13-TE, colored by hydrophobicity on the Kyte-Doolittle scale (between teal −20 and yellow 20), with X20404 (pdb ID:8TR4, ball and stick model) binding in a substrate-accommodating channel; active site Ser1533 is displayed in red ball and stick. (B) X20404 bound to the active site of Pks13-TE (magenta carbons) superimposed with a TAM16 bound structure (pdb ID:5V3Y, teal carbons). The protein is shown as a semitransparent surface, with the residues making H-bonds with X20404; catalytic Ser14553 and residues with drastic position change when compared to TAM16-bound structure are shown as sticks. (C) Positions of mutations conferring resistance to X20404 (shown as sticks with red carbons) in the X20404 (shown as sticks with tan carbons) bound structure; the protein is displayed as a semitransparent surface.

The DEL screen series 1 hit X20403 (Figure 1C) came from a library of over 2.8 billion compounds. All members of this library (library 1) contain a common triazole core. This library was synthesized using three cycles of chemistry: installation of primary amines as the first cycle of chemistry, followed by acylation of Fmoc-amino acids and a last cycle of triazole formation chemistry. X20403 showed potent activity with an IC_50_ of 57 nM in the Pks13-TE enzyme assay and 0.5 μM in the TAMRA binding assay. The truncated molecule X20404 was designed based on SAR observed in the selection output and was also synthesized (Figure 2). Biochemical activity data indicated these compounds were approximately equipotent; therefore, the lower molecular weight X20404 was used for further characterization and SAR exploration of this series.

A second series was established by the DEL hit X13045, which came from library 2 (Figure 1C), a 16-million member encoded library. This library was synthesized with 3 cycles of chemistry: acylation of aryl-halide containing carboxylic acids, followed by S_N_Ar reaction with Boc-diamines. The deprotected amine function was then further derivatized with a diverse set of carboxylic acids. X13045 showed modest inhibitory activity in the Pks13-TE enzyme assay (IC_50_ = 15 μM) but was taken into further optimization based on the indication of whole cell activity.

The screen of library 3 yielded the inhibitor X14146 (Table S5). All members of series 3 contain an oxadiazole core. This library was synthesized with 2 cycles of chemistry: (1) Cycle B is a set of ∼650 amino acids that undergo a heterocyclization reaction to form the oxadiazole core and (2) Cycle C includes a diverse set of ∼6000 capping BBs for acylation. While showing reasonable enzyme potency of 2.0 μM, X14146 was not active against Mtb. Nine analogues were made (Table S5), and the best was X20348 (Figure 1C) with anIC_50_ of 0.47 μM that resulted in a complex crystal structure (described below). However, series 3 was eventually deprioritized because the inhibitors were not active on Mtb. Subsequently, series 1 and 2 became the focus of the study.

### Cell Efficacy and On-Target Activity

Pks13-TE DEL inhibitors with IC_50_ values below 10 μM in the Pks13-TE activity or the TAMRA assay were tested for the growth inhibition of Mtb in culture medium. Assays were conducted in parallel with wild-type (WT) Mtb H37Rv and an engineered strain of H37Rv in which the native promoter of *pks13* was exchanged with a strong synthetic promoter containing a binding site for the tetracycline repressor TetR. Repression of this promoter by TetR was relieved by the addition of anhydrotetracycline (ATC). Thus, in the absence of ATC, the mutant is hypomorphic as Pks13 levels in this strain are depleted by ∼20-fold compared to WT H37Rv. In the presence of ATC, Pks13 levels are increased by ∼4-fold compared to WT due to inactivation of TetR and the strength of the synthetic promoter driving transcription of *pks13*. TP2, a previously reported Mtb active inhibitor acting on the N-terminal ACP domain of Pks13,^26^ was used as a positive control in each assay to confirm on-target activities against the mutant pks13 strain ± ATC compared with WT H37Rv; rifampicin was used as a negative control. The whole cell activity of these inhibitors, together with their MIC for the modified strain in the absence and in the presence of ATC, is listed in Tables S3–S5. The majority of active molecules showed decreased potency against the Pks13-hypermorph (in the range of 1.5 to 17 folds) while the hypomorph was more sensitive (ranging from 1.5 to 3.5 folds), demonstrating that the inhibitors were on-target.

### Structural Biology of Novel Pks13 Inhibitors

Representative compounds from series 1, 2, and 3 with favorable enzyme potency and solubility were cocrystallized with Pks13-TE and used to help guide the medicinal chemistry efforts (data collection and refinement statistics in Table S6). Consistent with their selection profiles, all three DEL series inhibitors were observed to bind to the active site of the enzyme, showing strong connected electron density (Figure S2).

**Figure 3:**
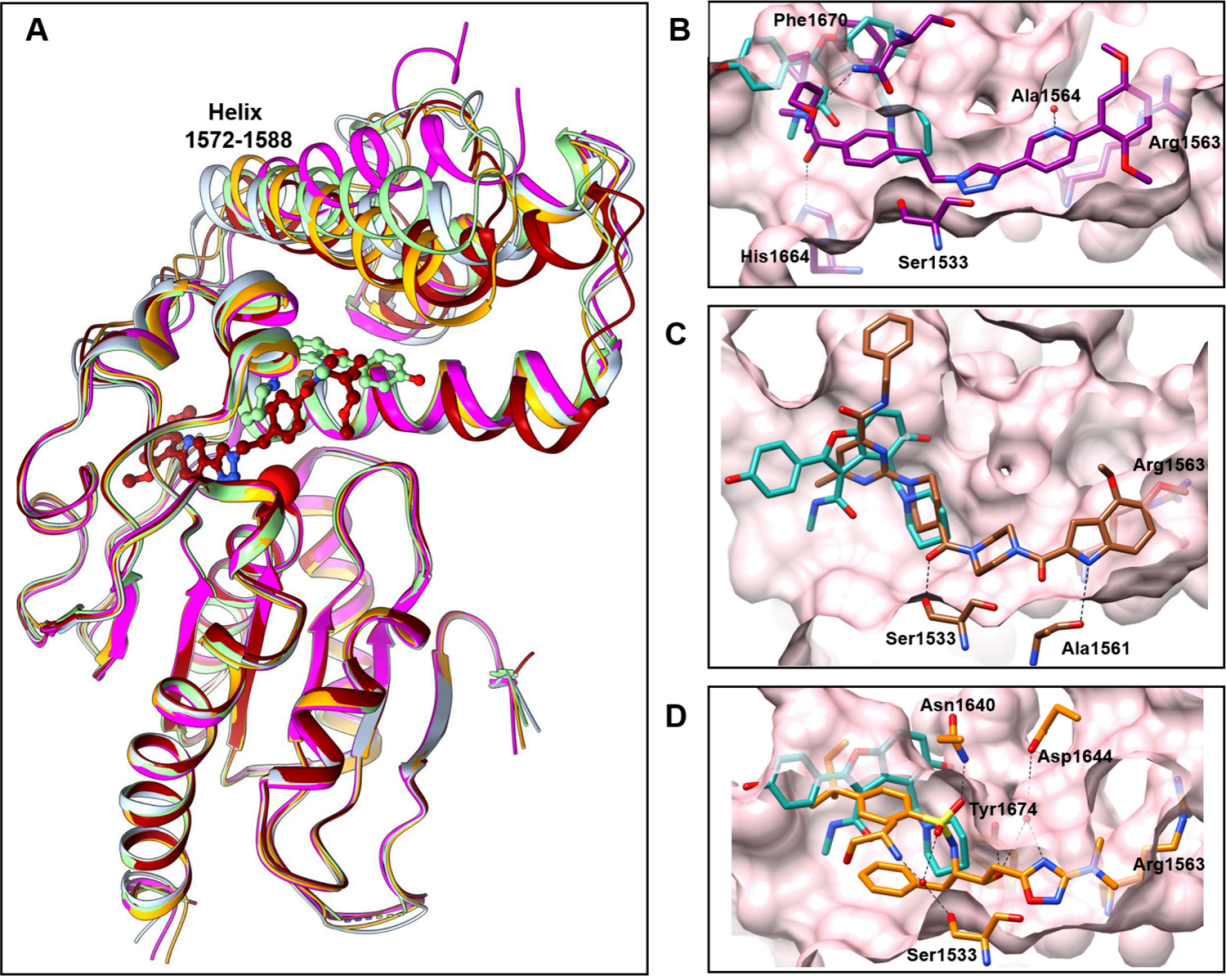
Binding of series 1–3 DEL-derived inhibitors to Pks13-TE. (A) Flexibility of the lid domain, evident by the change of the position of helix 1572–1588, from superposition of Pks13TE structures in a ribbon representation bound to TAM-16 (pdb ID:5V3Y, light green), X20403 (pdb ID:8TQV, maroon), X20404 (pdb ID:8TR4, magenta), X20419 (pdb ID:8TQG, light gray), X20348 (pdb ID:8TRY, orange); catalytic Ser1533 is shown as a red sphere, and TAM16 and X20403 are displayed as ball and stick models. X20403 (B), X20410 (C), and X20348 (D) bound to the active site of Pks13-TE superimposed with TAM16 bound structure (pdb ID:5V3Y, teal carbons model). The protein is shown as a semitransparent surface, with the residues making H-bonds with inhibitors; catalytic Ser14553, and residues with drastic position change when compared to TAM16-bound structure are shown as sticks.

Pks13-TE was preincubated with X20404 (series 1) prior to crystallization. Crystals were in the same *P*2_1_2_1_2 space group as previously reported for the TAM16 complex.^18^ There were two molecules of protein per asymmetric unit, each bound to one inhibitor. The crystals diffracted to a resolution of 2.1 Å (Table S6). Unlike TAM16, which inhibits by blocking the entrance to the catalytic center of Pks13-TE, X20404 binds closely to the catalytic serine (C23 of the X20404 is 3.25 Å from Ser1533 OG) and engages more extensively with the enzyme in a hydrophobic channel that likely binds the alkyl chain of the mycolic acid (Figure 2A,B). This channel passes through the central core of the enzyme, extending from one solvent-accessible side of Pks13-TE to the other (Figure 2A). The channel occupied by X20404 was not completely visible in the Pks13-TE-TAM16 structure because the side chain of Arg1563 blocked it. To accommodate the R2 group of X20404 required the displacement of the Arg1563 side chain, and an entire segment of the protein from Thr1645 to Gln1649 is also shifted by approximately 2.4 Å (as measured at CA of Thr1645) to open the channel compared to the TAM16 bound structure, as shown in Figure 3C. Binding interactions are dominated by van der Waals (VDW) contacts, which are expected given the hydrophobic character of the channel. Four hydrogen bonds contribute to the binding energy through the following interactions: O29 of R1 with NE2 of His1664; N16 of pyridine of R1 interacts with water connected to the backbone NH of Ala1564 and, through one more water, to the NE of the Arg1563 side chain. The hydroxyl of the catalytic Ser1533 is 3.25 Å from the C23 of the R1 phenyl and 3.38 Å from the C20 of the linker to the core. The Asn1640 side chain, an interaction previously described as being crucial to the potent activity of TAM16, stacks with the same phenyl ring pi electrons. The Phe1670s side chain, which flips to stack with the benzofuran core of TAM16, is positioned to form edge-on pi stacking interactions with the R2 phenyl of X20404. Only the R2 group of X20404 overlaps with the binding position of the piperidine and ketoamide groups of TAM16 (Figure 2B), consistent with the competitive selection profile observed in the selection output for this compound.

The cocrystal with X20403 solved at 2.0 Å resolution demonstrated a similar binding pose as X20404 (Figure 3B). Interestingly, the cyclopropyl group induced a shift of the α-helix formed by residues Lys1572-Lys1588 (Figure 3A), pulling the C-terminus of the helix closer to the inhibitor by 5.5 Å, as measured at the C-alpha of Phe1585. An additional H-bond was observed between the terminal methoxy O2 of the compound and ND2 of Asn1640. The side chains of Tyr1582 and Phe1585 are within VDW distance to C39 of the cyclopropyl group. Likely due to this gross change in the overall structure of the protein (Figure 3A), crystals obtained for the X20403 complex packed differently, and the structure was solved in the *P*222_1_ space group, containing two protein molecules in the asymmetric unit, each bound to an inhibitor.

The structure of the series 2 compound X20419 in complex with Pks13-TE was solved using X-ray diffraction data collected to 2.2 Å resolution. Crystals were in the tetragonal *P*4_1_2_1_2 space group with one molecule per asymmetric unit. X20419 was found to reside within the same channel as that for X20404, extending through the core of Pks13-TE via a conformational change in the Arg1563 side chain, as described above (Figure 3C). Most of the contacts between the protein and the inhibitor are VDW/hydrophobic interactions with the addition of two hydrogen bonds: between the N9 of the indole ring of R1 and the carbonyl of Ala1561, at 3.3 Å, and a weak bond between the catalytic Ser1533 OG to the carbonyl (O21) of the inhibitor, at 3.2 Å. The terminal aromatic ring system of R1 forms VDW interactions with the carbons of the Arg1563 side chain. Two methoxy substituents on the ring contact the Ile1648, Ile1643, and Tyr1686 side chains. The piperidine ring of R1 is angled to achieve a pi-stacking interaction between the pyrimidine ring and Phe1670. The piperidine overlaps with the position of the piperidine in the TAM16-bound structure, while the pyrimidine extends into a space occupied by the benzofuran of TAM16, without causing the Phe1670 side chain flip. The enamine linker of R2 places the terminal phenyl into a hydrophobic pocket created by the side chains of the Phe1670, Tyr1637, Arg1572, Arg1641, and Trp1579. The C16 of the piperidine is also 3.4 Å away from the catalytic Ser1533 OG, in addition to the H-bond to O21, thoroughly blocking substrate access.

X20348 from series 3 showed reasonable potency against Pks13-TE with an IC_50_ value of 0.9 μM in the TAMRA-based binding assay. We did not pursue inhibitors of this series because they did not inhibit Mtb growth. However, we included the structure of X20348 bound to Pks13-TE because of its unusual branched structure. Crystals of X20348 bound Pks13-TE formed in the *P*2_1_2_1_2_1_ space group with two molecules per asymmetric unit at a resolution of 2.35 Å. The binding site of X20348 was in the same channel as that described above for the series 1 and 2 inhibitors (Figure 3D). While an additional copy of X20348 was found to be located at the interface between two protein chains (Figure S2C), we are most interested in the molecule in the channel. The dimethylamino-oxadiazole resides in the same part of the channel as the R2 moieties of series 1 and 2, and although it is smaller, it induces the same displacement of the Arg1563 side chain as the other two inhibitor series. The binding was dominated by VDW interactions and several hydrogen bonds with the sulfone: O14 with the ND2 of the Asn1640 side chain (2.96 Å), O13–to water (3.15 Å), which in turn bonds with the OG of catalytic Ser1533 and the backbone nitrogen of Ala1477. The N15 of the inhibitor makes an H-bond with the OH of Tyr1674 (3.16 Å), and the carbonyl O25 is also within H-bonding distance of the same Tyr1674 (2.84 Å) and a water (3.18 Å), which links it to OD1 of Asp1644 (3.55 Å) and the N28 of the oxadiazole core of X20348 (2.93 Å). The catalytic Ser1533 OG is making VDW contacts with the C16, C24, and C26 (3.27, 3.4, and 2.9 Å, respectively) of the linker between the oxadiazole ring and the sulfonamide. The branched part of X20348 shows a conformation in which the benzyl is stacked with the isopentyl-phenyl and is accommodated in the more flexible part of the Pks13-TE pocket, with the labile 1572–1588 aa helix (Figure 3A) positioning the Phe1585 side chain to interact with the isopentyl.

### Preliminary SAR of Series 1 and 2 Inhibitors of Pks13-TE

**Figure 4:**
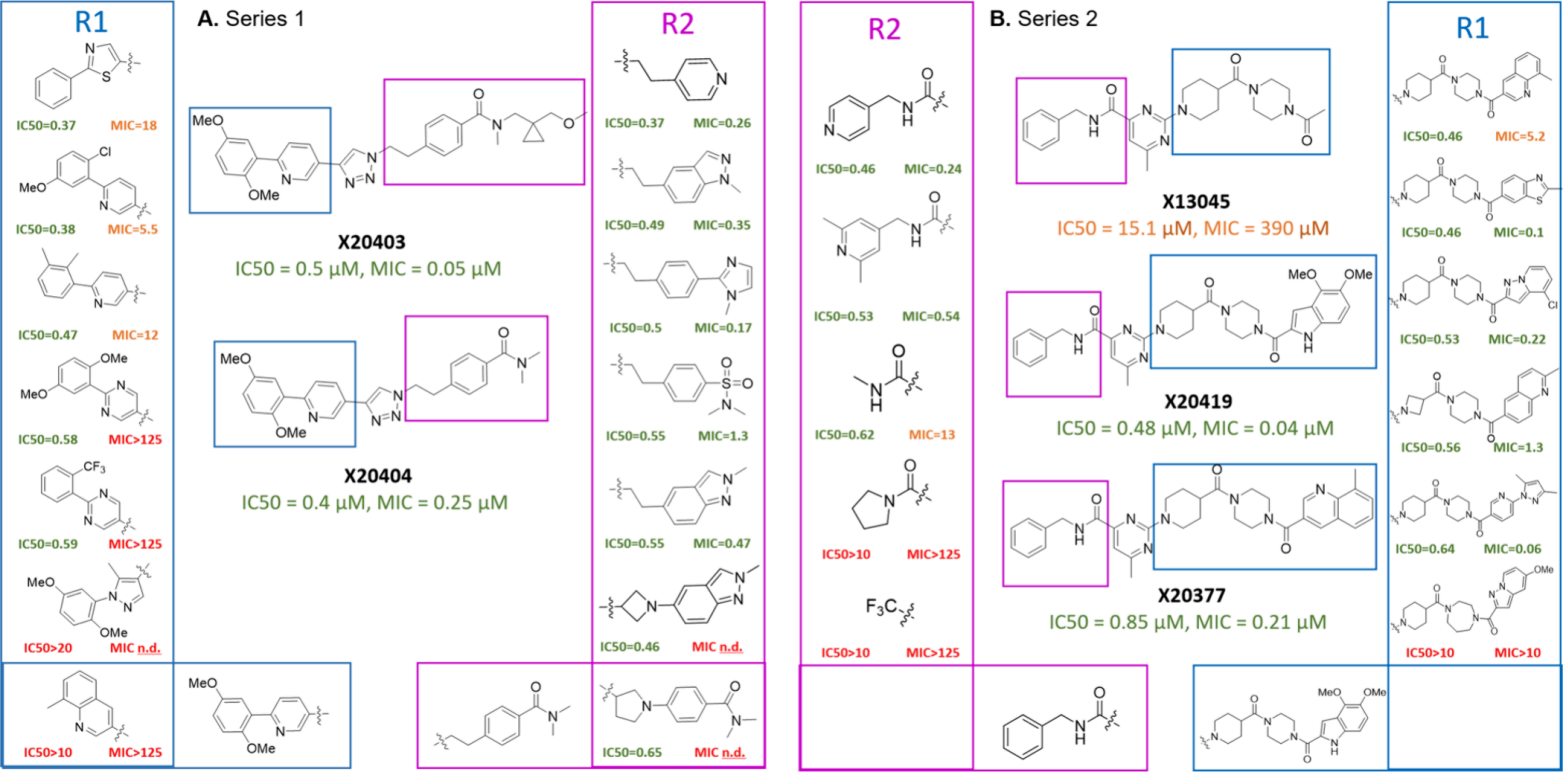
SAR of series 1 (A) and series 2 (B) with enzyme and Mtb inhibition listed. R1 substituents are marked by blue boxes, and R2 substituents are noted by magenta.

The design of additional inhibitors was informed by data derived directly from the DEL screen output along with cocrystal structure and enzymatic assay data. Activities against the enzyme and Mtb cells for series 1 and 2 founders and analogues are shown in Figure 4, and full data, with the hypo- and hypermorph Mtb activity fold changes for all three series, could be found in Tables S3–S5.

Modifications to the R1 region of the series 1 compound, X20404, were explored (Figure 4A, Table S3). R1 fits snugly inside the binding channel, contributing several VDW interactions, and forms three H-bonds to water molecules via N16 of pyridine and O2 and O7 of two methoxy groups. Modification of the electron-donating methoxy groups to either a chloro or trifluoromethyl caused no loss in activity (Table S3, entries 4, 5, and 9). Some tolerability of the pyridine portion of R1 was also observed, as inhibitory activity was maintained in the phenyl and pyrimidine analogues (Table S3, entries 3 and 7). However, a significant loss in potency occurred when the pyridine was modified to a pyrazole or quinoline (Table S3, entries 10–12). Furthermore, replacing the dimethoxy-phenyl portion of R1 with azetidine or an ether-linked analogue led to a complete loss of activity (Table S3, entries 23 and 24). These data suggest that R1 displays a steep SAR with significant loss of inhibitor potency when changes are made that modify the planarity and size of R1. Another interesting observation is a loss in potency when changing the triazole core to an oxadiazole (Table S3, entry 26). The empirical data collected from testing these analogues are consistent with the crystal structure, where the triaryl ring system is vital to binding.

The amide tail containing R2 of X20404 resides in the part of the channel that overlaps with the binding site of TAM16. There is a hydrogen bond formed between the carbonyl of the amide with side chain of His1664 and interactions with Phe1670 side chain, with the methyl substituents pointing toward the solvent. Modifications at the R2 region can be accommodated by this broad, solvent-exposed part of the binding site, with added positional flexibility of the 1572–1588 aa helix. Several follow-up analogues were synthesized and tested (Figure 4A, Table S3). Modification of the *para*-dimethyl amide phenyl to an unsubstituted pyridine maintained activity (Table S3, entry 13). Further changes to the amide, including an *N-*methyl indazole, methyl pyrazole, or sulfonamide, showed similar potency to X20404 (Table S3, entries 14, 15, and 16, respectively). Interestingly, even significant modifications, such as changing the ethylene linker to a pyrrolidine or azetidine, were tolerated (Table S3 entries 18, 19, 20, and 27, respectively). These data suggested that the R2 portion of Series 1 would be amenable to the addition of groups that might improve molecular properties, e.g., kinetic solubility and PK.

The series 2 inhibitor X13045 showed only modest enzyme inhibition (IC_50_ = 15 μM) and weak whole cell activity (MIC = 390 μM). Extending the R1 position with bulky aromatic groups to fill in the hydrophobic pocket made by the Arg1563 side-chain displacement resulted in a 30-fold improvement in enzyme inhibition for compound X20419 with indole appended by two methoxy groups and other fused heterocycle rings (Figure 4B, Table S4). Accurate placement of this hydrophobic group is important, as changing the relative angle and distance from the pyrimidine core by replacing the piperazine ring with a homopiperazine abolished enzyme inhibition (Table S4, entry 17). Consistent with the observation in the cocrystal structure, the indole NH forms an important H-bond with the carbonyl of Ala1561 (Table S4, entry 7), with an improved inhibitory activity of Pks13-TE and significant improvement in antibacterial activity while preserving the on-target activity signature. Further SAR exploration of the indole derivative is warranted for optimization of this series. Changes to R2 had little effect on enzyme inhibition activity, as expected from its proximity to the solvent. The phenyl to pyridine replacement (Table S4, entries 4 and 10) as well as truncation of the phenyl (entry 12) were both well-tolerated. The amine of R2 resides in a narrow part of the pocket (∼3.5 Å to Asn1640 or Phe1670 closest points), as a loss of activity is observed when benzylic methylene is replaced by a bulkier five-membered pyrrolidine (Table S4, entry 15). The ketoamide group of R2 appears to be important for binding, making VDW contacts, as its replacement with a trifluoromethyl group resulted in a loss of inhibitory activity (Table S4, entry 16). The R1 region of X13054 can be modified for additional ligand-protein interactions, while the opposite side of the molecule, R2, much like the R2 of series 1, can be used to affect properties, as many changes are tolerated due to the binding position facing the solvent and being surrounded by more flexible parts of the protein.

### Selection of Resistant Mutants

To determine if the inhibitor series were targeting Pks13 in Mtb, resistant mutants were selected in the presence of X20404 (MIC = 0.25 μM). Because the crystal structures indicated that series 1 and 2 compounds bind in the same location, we expected to see cross-resistance to inhibitors from both series. Resistant colonies were obtained with a frequency of resistance (FOR) of 4.7 × 10^–7^, which is similar to the FOR of the Pks13-TE inhibitor, TAM16,^18^ and significantly better than the FOR of isoniazid.^35^ Eleven colonies were picked from plates containing X20404 at 10× its MIC. These colonies were propagated, used to quantify the level of resistance, and then used to prepare genomic DNA for whole-genome sequencing. All sequenced isolates contained point mutations in the thioesterase domain of Pks13 (Table 1, Table S7). Moreover, there were no mutations observed in any other essential proteins,^14^ confirming that the inhibition of Pks13-TE was the mechanism of action. Several of these mutant strains showed cross-resistance to X21429 (Table S4), a series 2 inhibitor with potent Mtb activity (MIC = 0.24 μM). A complete list of mutations of all isolates can be found in Table S7.

**Table 1:** Representative Set of Pks13-TE Containing SNPs Resistant Mutants Selected against X20404 with Their Relative Sensitivity to Series 1 and 2 (X21429) Compounds as well as TAM16 and Two Controls^a^

	Fold Change in MIC (Mutant MIC/WT H37Rv MIC)				
Mutation in Pks13-TE (Missense)	Rifampicin	TAM16	TP2	X20404	X21429
R1563H	0.9	3.3	0.8	>63	1.6
A1561 V	1.2	1.4	1.0	40	>57
A1564D	0.8	140	0.8	>63	17
N1640K	1.5	>140	0.9	37	>57
V1537A	1.3	4	1.0	>63	8.8
V1687F	1.2	1.9	1	>63	2.3
Y1674C	1.6	87	0.8	>63	18
Y1686C	1	1	0.9	19	3.8

a Two controls: rifampicin and TP2.

Resistant mutant strains provide additional evidence of the on-target activity of these inhibitors inside of Mtb cells. All X20404 resistant mutations map into the inhibitor binding pocket of Pks13-TE (Figure 2C), and the level of resistance conferred was readily explainable by either the loss of binding interactions with the inhibitor or a steric clash with the inhibitor. X21429 (a series 2 inhibitor) was also tested on the resistant mutant strains. The level of resistance to series 2 compound (X21429) was lower compared to X20404 for most of the point mutations (Table 1) in Pks13-TE, except for two mutations: A1561, which when mutated to a bulkier Val likely prevents the accommodation of the core for either series molecules, and N1640K, which would clash with R1 of X20404 and the pyrimidine core of series 2. The mutations to Alanine or Phenylalanine of V1537, which is opposite to A1561 at the core-accommodating part of the active site, also provide a moderate (5–8 fold) resistance to series 2. A1564D introduces both extra atoms and a negative charge which likely influences the position of the Y1674 side chain.

### Pharmacokinetics and In Vivo Efficacy

**Figure 5:**
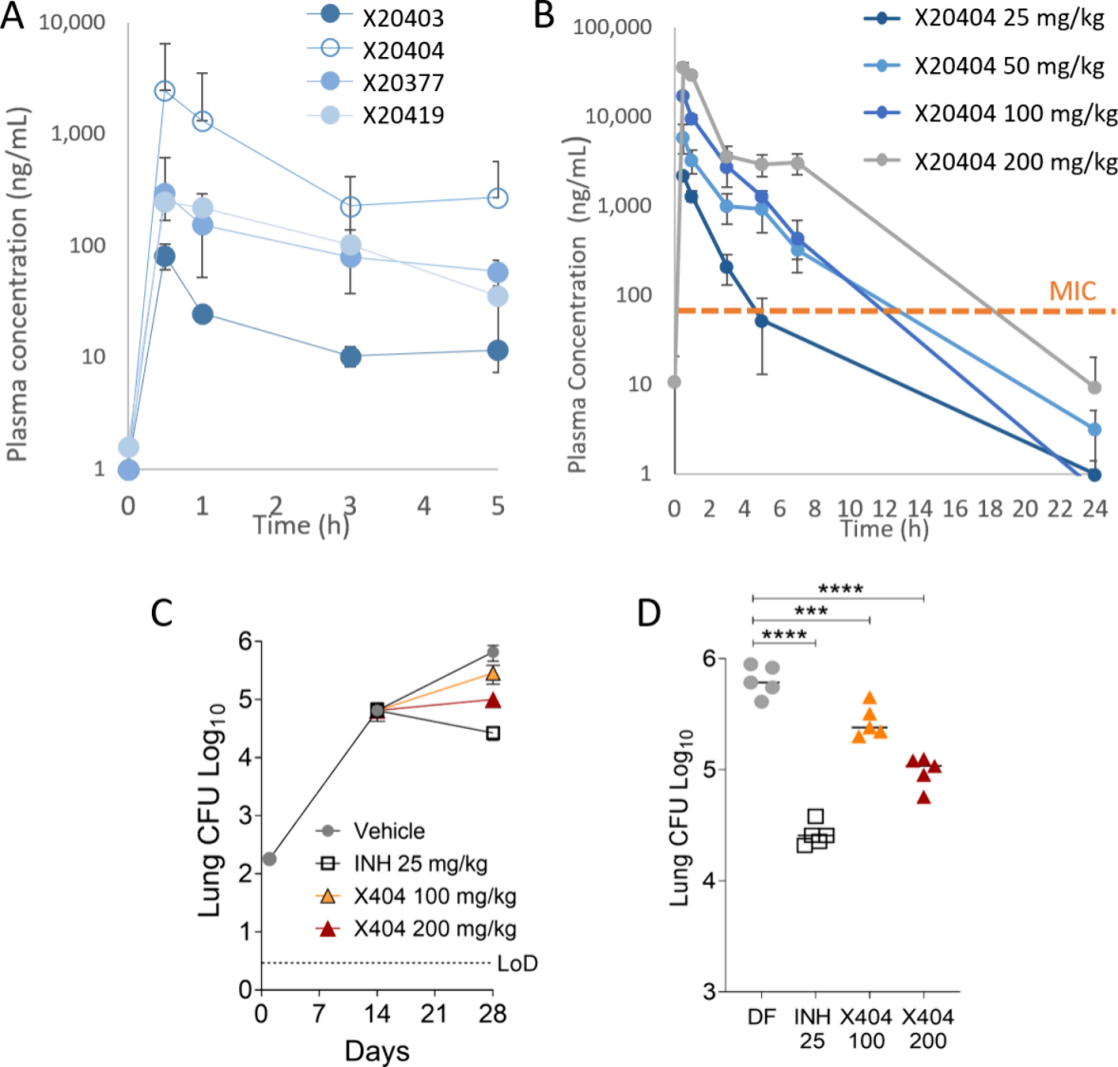
Mouse pharmacokinetic profiles of selected pks13 inhibitors and the efficacy of X20404 in the mouse model of acute infection. (A) Concentration–time profile of pks13-TE inhibitors in CD-1 female mouse plasma following a single oral dose of 25 mg/kg. Mean and standard deviations are shown: *n* = 2 per compound. (B) Concentration–time profile of X20404 in CD-1 mouse plasma following single oral doses, as indicated. Mean and standard deviations are shown, *n* = 3 mice per dose. (C) Schematic of the efficacy study and drug response. LoD: limit of detection. (D) Individual mouse lung burden data and statistical analysis. Lung burden data were analyzed using one-way ANOVA with multicomparison and Tukey’s posttest. *N* = 5; ***p < 0.001, ****p < 0.0001.

Two representative inhibitors from series 1 (X20403 and X20404) and two from series 2 (methoxy indole-containing X20419 and methylquinoline-containing X20377) were profiled in mouse snapshot PK studies. The results showed that X20404 achieved much higher exposure in mouse plasma following a single oral (p.o.) dose of 25 mg/kg (Figure 5A) compared to the other 3 inhibitors. X20404 showed ∼10-fold higher maximum concentration in blood compared to three other compounds (Figure 5A). In vitro and in vivo PK parameters of X20404 are summarized in Table 2.

**Table 2:** *In Vitro* and *In Vivo* Pharmacokinetic Parameters of X20404

PK properties	X20404
**in vitro**	
mouse PPB (%)	97.7 ± 0.1
human PPB (%)	95.6 ± 0.7
MLM *T*_1/2_	26.8 min
MLM CL_int_	26.2 μL/min/mg
**in vivo (mouse)**	
*V*_d_	0.3 L/kg
CL	6.2 mL/kg·min
elim *T*_1/2_	0.6 h
i.v. AUC[0–24] 5 mg/kg	13.5 μg·h/mL
Bioavailability (F)	8%
p.o. AUC[0–24] 25 mg/kg	3.7 μg·h/mL
p.o. AUC[0–24] 50 mg/kg	14.2 μg·h/mL
p.o. AUC[0–24] 100 mg/kg	33.3 μg·h/mL
p.o. AUC[0–24] 200 mg/kg	98.1 μg·h/mL

Dose-escalation PK studies with X20404 up to 200 mg/kg in mice revealed a slightly overproportional exposure-to-dose relationship, suggesting saturation of one or more elimination processes at the higher doses (Figure 5B). After five daily oral doses of 200 mg/kg, plasma concentrations were above the MIC (not corrected for plasma protein binding) for most of the dosing interval. Since this highest tested dose was well-tolerated for 5 consecutive days, we proceeded with an efficacy evaluation in a mouse model of acute TB infection. There was no overt toxicity or adverse effects noted in the dose-escalation studies.

An acute mouse efficacy study was conducted with X20404 using mice that were infected with Mtb H37Rv via the aerosol route with treatment initiated 14 days postinfection with X20404 at 100 and 200 mg/kg daily for 14 days. Isoniazid (INH) at 25 mg/kg (the human equivalent dose) served as a positive control and a cell wall inhibitor reference. X20404 achieved a statistically significant and dose-responsive reduction in lung bacterial burden after 2 weeks of treatment (Figure 5C,D): at 100 mg/kg—approximately 0.4 log_10_, and at 200 mg/kg—0.8 log_10_ reduction of the lung CFU compared to untreated control, with the INH control achieving ∼1.5 log_10_ reduction in CFU in the same experiment.

## Discussion

Inhibitors of cell wall biosynthesis have proven to be important mainstays in our arsenal of antibacterials. However, many of these drugs are reaching the end-of-life status due to widespread resistance from decades of use worldwide. This is true for the two TB first-line drugs isoniazid and ethambutol, which will be difficult to replace because they are inexpensive to produce and are generally well-tolerated for months of daily administration. While ethambutol targets the cell wall, isoniazid is a prodrug that is activated by a catalase peroxidase KatG and targets the enoyl reductase of the type II fatty acid synthase.^10^ The enzyme elongates fatty acids to make precursors for the Pks13 substrates. Since the activator enzyme KatG is not essential, mutations in KatG are common in clinical strains and the barrier to isoniazid resistance is low.^11^

Pks13 fulfills a critical function for Mtb by performing a condensation reaction of a long-chain fatty acid and a very long-chain meromycolic acid to form a mycolic acid. The entire surface of Mtb is coated with mycolic acids that are either covalently attached to the cell wall or intercalate into this outer membrane-like structure. Thus, mycolic acids are in very high demand during the growth and division of the bacterium. Therefore, disrupting mycolic synthesis through inhibition of Pks13 should undermine bacteria cell growth and viability and make it more permeable to other drugs. The outer mycolic acid layer has been shown to be responsible, at least in part, for the poor activity observed for many FDA-approved antibacterials and lead discovery molecules focused on Mtb, and in many cases, this has been attributed to their inability to penetrate this waxy coat. The shear mass of mycolates that must be made to support growth and cell division is an enormous burden for the cell, requiring many enzymes and metabolites. As is the synthesis of the very large 1733 amino acid, multidomain enzyme Pks13. Other downstream consequences of Pks13 inhibition are likely toxicity related to the buildup of fatty acid substrates and their degradation products in the cell.^36,37^ Together these outcomes from Pks13 inhibition are likely the reason why it is among the proteins most vulnerable to depletion using CRISPRi profiling experiments, with the score of minus 9.^16^ The vulnerability of Mtb to a Pks13 chemical inhibition was demonstrated by our discovery of a potent inhibitor of Mtb Pks13-TE, TAM16, which had comparable activity to first-line Mtb drugs, were active against a broad panel of TB drug resistant strains, and had a low frequency of resistance compared to isoniazid.^18^ And importantly, treatment with TAM16 was synergistic with a first-line antitubercular drug Rifampicin. Unfortunately, the TAM16 series showed hERG cardiotoxicity stemming from its apolar nature and a primary amine required for potent activity. There has been much effort on finding new inhibitors to this very impactful target.^19−22,25,26,29^ However, a challenging activity assay for Pks13-TE has hindered the discovery of new inhibitors using HTS. For example, a recent traditional HTS of Pks13-TE of 150,000 compounds produced only two confirmed hits and only one of them with a novel scaffold,^29^ thus underscoring the need to turn to larger more highly diverse compound libraries and alternative screening approaches, both provided by DEL screening.

When compared to more traditional screening approaches that use spatially separated individual compounds, DEL screening permits deeper sampling of chemical space and can be conducted in a more time- and resource-efficient manner. Individual DNA-encoded chemical libraries are constructed by the exhaustive combination of chemical building blocks within a defined synthetic scheme. These individual libraries, in turn, can be combined and screened as a mixture, thereby permitting simultaneous and rapid access to billions of different compounds, the vast majority of which will be genuinely novel. Screening compounds in a mixture enables parallel testing to inform upon the hit binding site, selectivity, and other mechanistic details. Because these libraries are synthesized in a combinatorial fashion, binding hits exist in clusters of structurally related compounds, and these data may be immediately utilized to guide compound advancement, as exemplified by the work on series 1–3 reported here.

In DEL screen of Pks13-TE we discovered five chemically distinct and novel inhibitor series, three of which demonstrated potency with IC_50_ values in the nanomolar range and a SAR. Surprisingly for target-based selected inhibitors, two of the three DEL hits also demonstrated potent Mtb inhibitory activity, which often is an unsurpassable hurdle in TB drug discovery. Moreover, representative compounds of these two series demonstrated no toxicity and reasonable PK. Of the four most potent Mtb inhibitors, representing two series, compound X20404 was the most suitable for acute TB infection mouse efficacy studies from the perspective of AUC/MIC. It was remarkable that the screening hit X20404 remained the lead inhibitor through the biochemical, in vivo, and PK testing, and when it was dosed to infected mice, it showed a dose-dependent reduction in TB burden when compared to the vehicle control in an acute model of infection. TAM16 administered in a similar mouse model of infection achieved a 0.9 log_10_ reduction of CFU in the lungs when dosed at 200 mg/kg, while INH reduced the burden by 1 log_10_. X20404 dosed at 200 mg/kg reduced lung CFU by 0.8 log_10_ with the INH control achieving ∼1.5 log_10_ reduction. This is not significantly worse than the efficacy of the extensively optimized TAM16, especially considering that X20404 is a screening hit. X20404 also showed similar clearance profiles in animals as TAM16, comparable *T*_max_ and *t*_1/2_, and comparable microsome stability. The difference that stands out is the plasma protein binding, at 73% for TAM16 and 98% for X20404, with the volume of distribution for X20404 consequently being low (0.3 L/kg) compared to TAM16 (4.2 L/kg). This difference is most likely responsible for lower efficacy, and the future optimization of X20404 aimed at reducing protein binding is expected to improve the volume of distribution and consequently the efficacy.

X-ray crystallography verified that compounds from all three different series were binding deep within the active site of Pks13-TE, displacing the side chain of Arg1563 with atoms within about 4 Å from the catalytic Ser1533 OG. These structures provide potential new contact information and also additional conformations of the active site to explore in virtual screens and drug design work: Arg1563 side chain relocation opening new part of the channel and connection to solvent, alternative positions of Phe1670 side chain, and the flexibility of the “lid domain” with significant movement of helix 1572–1588 (Figure 3A) changing the shape of the entrance cavity.

The results of DEL screening complemented by the biochemical and in vivo testing, in combination with the X-ray crystallography derived detailed atomic binding information presented in this paper, could serve as a starting point for lead development and optimization projects. The larger amount of data derived from DEL screening compared to traditional HTS outputs could also constitute a great training set for artificial intelligence (AI)-based discovery of new leads. The significant progress achieved in the past few years by AI-assisted drug discovery holds great promise to accelerate the drug development process and to do so in a more cost-effective way, which is especially critical for neglected diseases like TB. Discovery of new anti-mycobacterial leads and rapid dissemination of data like this is essential to expedite the drug development process, potentially bringing life-saving treatments to patients faster.

## Materials and Methods

### Pks13-TE Protein Expression and Purification

Pks13-TE domain (residues 1451–1733 of Rv3800c) with N-terminal TEV cleavable tag was expressed in *Escherichia coli* and purified as described in ref (18). For the DEL selection, the His-tag was left intact, and the protein was subjected to gel filtration directly after the initial Ni column purification step. For the activity assay and crystallization, the His-tag was cleaved by TEV protease as described in ref (18).

### Pks13-TE and Other Essential Proteins

All protein samples were centrifuged at 14,000*g* for 10 min at 4 °C and then filtered using a 0.2 um PES nanofilter vial (Thomson) prior to experimentation. Proteins were characterized by analytical Size Exclusion Chromatography (SEC) using an Agilent 1260 liquid chromatography stack equipped with a UV detector. Molecular weight was determined by calculating relative to a protein standard (Bio-Rad Gel Filtration Standard, #1511910). 10 μL of a 230 μM sample was injected onto a Superdex 200 Increase 5/150 GL column at a flow rate of 0.5 mL/min in 20 mM Tris pH 8.0 and 100 mM NaCl. Dynamic Light Scattering was performed on a Wyatt DynaPro NanoStar instrument at room temperature. Seven microliters of 230 μM His6-Pks13-TE was loaded into a disposable cuvette, and 5 measurements were recorded, each consisting of ten 10-s acquisitions. Data was analyzed in Wyatt’s Dynamics software to obtain reported values.

### Pks13-TE Activity Assay

Enzyme activity of Pks13-TE was assessed using 4-methylumbelliferyl heptanoate (4-MUH, Sigma) as a fluorogenic substrate in a 96-well plate format. To make initial velocity measurements, Pks13-TE (0.5 μM) in 0.1 M Tris-HCl, pH 7 buffer was incubated with different concentrations of 4-MUH (0.2–200 μM in DMSO, 1% DMSO final) in a 100 μL reaction volume, and the fluorescence of the hydrolyzed product 4-methylumbelliferone was read (excitation at 355 nm and emission at 460 nm) using a PolarStar Omega plate reader (BMG Labtech) at 5–10 min intervals over 120–140 min. The reaction rate was observed to be linear in the measured range. 4-MUH in buffer alone was included as a control to quantify its background hydrolysis. Data points were plotted as an average of duplicates and analyzed using Prism software (GraphPad) to determine the kinetic parameters *K*_m_, *V*_max_, and *k*_cat_. The experiment was repeated in 20 mM HEPES, 134 mM potassium acetate, 8 mM sodium acetate, 4 mM sodium chloride, and 0.8 mM magnesium acetate, pH 7.2.

### Immobilized Pks13-TE Enzyme Activity Assessment

His-Pks13-TE was immobilized onto His-cOmplete affinity resin in 60 μL of Selection Buffer (20 mM HEPES pH 7.2, 134 mM potassium acetate, 8 mM sodium acetate, 4 mM sodium chloride, 0.8 mM magnesium acetate) supplemented with 1 mg/mL Sheared Salmon Sperm DNA, and 0.02% CHAPS. Resin was washed three times with Selection Buffer, and then 60 μL of substrate (100 μM 4-MUH, 1% DMSO final) was incubated with the affinity resin with constant motion to allow reaction with immobilized protein. Time points were taken over the course of 190 min, and product fluorescence was measured to monitor protein activity.

### DNA-Encoded Library Selection

Fifty-nine different DNA-encoded chemical libraries (DELs) were mixed and incubated with His-Pks13-TE in 60 μL of Selection Buffer. Separate tubes were prepared to interrogate different DEL screening conditions, including a no-target control (NTC) lacking protein, His-Pks13-TE at three concentrations (10, 2, and 0.5 μM), and two concentrations of His-Pks13-TE (10 and 2 μM) preincubated with 50 μM of a known ligand (TAM16) for 45 min prior to library addition (conditions summarized in Table 1). 50 pmol of each library was added to each condition, and the library mixture was allowed to incubate for 1 h with each mixture. The solutions were then captured onto 5 μL of pre-equilibrated His-cOmplete IMAC matrix embedded within a 200 μL Phytip using the Phynexus ME200 automated multichannel pipettor. Following 30 min of protein capture onto the affinity matrix, the matrices were washed 8 times with Selection Buffer. Co-enriched DEL members were eluted from the matrix by heating it to 85 °C for 5 min in Selection Buffer. A second round of selection was performed using fresh aliquots of protein (and competitor, where appropriate) and the round one DEL eluant. The eluant from the second round of selection was PCR-amplified using primers that introduce READ1 and READ2 sequences for subsequent Illumina Next Generation Sequencing. The sequence sample was analyzed using an Illumina 2500 instrument in high-output mode, yielding 514 million single-end reads from all selection conditions and libraries.

### Hit Testing against Pks13-TE with Affinity Probe Competition Assay

The competition binding assay was used to assess compound biochemical activity. ActivX TAMRA-FP Serine Hydrolase Probe was purchased from ThermoFisher. Covalent binding of the probe to Pks13-TE was used to read fluorescence polarization and set up the assay based on that signal.^38^ The assay was done in all-black 384-well plates in a total volume of 25 μL. Pks13-TE in 100 mM Na phosphate buffer at pH 7.4 with 1.6 mM CHAPS at final concentration of 2 μM was preincubated with compounds for 10 min at RT, then TAMRA fluorescent probe was added to a final concentration of 80 nM, mixed and incubated for 40 min, followed by measuring fluorescence polarization signal at excitation 540 nm and emission of 590 nm on Clariostar BMG Plate reader. Percent inhibition for the dose-response plots was calculated using 2% final DMSO as a negative control and 20 μM TAM16 as a positive control.

### MIC Determination and Comparison for H37Rv and pks13-TetON

Wild-type (WT) H37Rv and *pks13*-TetON (which overexpresses Pks13 to about 400% of the WT level when grown with Anhydrotetracycline [ATc] and under-expresses Pks13 to about 20% of the WT level when grown without ATc)^22^ were each cultured in 10 mL of Middlebrook 7H9 (with Hygromycin [Hyg] at 50 μg/mL, Kanamycin [Kan] at 25 μg/mL, and ATc at 500 ng/mL for the mutant strain) supplemented with 0.2% (v/v) glycerol, 0.05% (v/v) Tyloxapol or Tween 80, and ADNaCl (for 0.5% [w/v] BSA, 0.2% [w/v] dextrose, and 0.085% [w/v] NaCl) or 10% (v/v) OADC, in a 25 cm^2^ tissue culture flask with a vented cap. After approximately 7 days at 37 °C and 5% CO_2_ in a humidified incubator, growing to an OD_580_ of approximately 1, each of the cultures was washed twice with fresh BSA-Free 7H9 supplemented with 0.2% (v/v) glycerol, 0.05% (v/v) Tyloxapol, 0.03% (w/v) Bacto Casitone, 0.2% (w/v) dextrose, and 0.085% (w/v) NaCl. The strains were suspended to an OD_580_ of 0.05 in 10 mL of BSA-Free 7H9 (with Hygromycin and Kananamycin as before for the mutant but both with and without ATc at 500 ng/mL) in a 25 cm^2^ tissue culture flask with a vented cap. After a further 5 days at 37 °C and 5% CO_2_ in a humidified incubator, the strains were diluted to an OD_580_ = 0.01 in BSA-Free 7H9, with Hygromycin and Kanamycin for the mutant and ± ATc as appropriate. Compounds were solubilized in DMSO and dispensed into black, clear-bottom 384-well tissue culture plates using an HP D300e Digital Dispenser as 14-point, 2-fold dilution series in triplicate. 50 μL of OD_580_ 0.01 cell suspension was pipetted or dispensed using a Thermo Fisher Multidrop Combi Reagent Dispenser into each well, and plates were incubated for 7–14 d at 37 °C in the same conditions as above in stacks of no more than six plates wrapped with aluminum foil. Final OD_580_ values were normalized from 0–100% to the averages of no-drug (100%) and no-growth (0%) control wells. MIC values were calculated by fitting the log(inhibitor) vs response data in GraphPad Prism to a Gompertz model provided by GraphPad (https://www.graphpad.com/support/faqid/1365/), with bottom and span best-fit values constrained to 0 and 100, respectively. When the calculated MIC was higher than the highest dose tested, it was reported to be greater than that dose. All MIC measurements were made at least twice in independent replicate experiments.

### Resistant Mutants Generation, Resistance Confirmation, Sequencing, and Analysis

WT H37Rv was cultured in 10 mL of Middlebrook 7H9 as described above, growing to an OD_580_ of approximately 1. The culture was washed with 7H9 and resuspended to an OD_580_ of approximately 2. 568 μL of this suspension (approximately 10^8^ bacteria) was plated on 25 mL of Middlebrook 7H10 agar supplemented with 0.5% (v/v) glycerol and 10% (v/v) OADC and containing 2.0 μM X20404 (approximately 10× its broth MIC) in a standard 10 cm diameter Petri plate. The initial number of bacteria was determined by plating dilutions of the original culture. The plates were bagged and incubated at 37 °C for 3 weeks, and colonies were counted. The FOR was calculated as the number of CFUs on the compound-containing plate divided by the total number of bacteria plated. Eleven of the 44 colonies counted on the compound-containing plate were picked at random and propagated to confirm resistance (sequencing data on all isolates can be found in Table S7).

MIC measurements were performed as above with the exception that WT H37Rv and the mutant candidates were cultured directly in BSA-Free 7H9 to an OD_580_ of approximately 1, then were diluted to OD_580_ = 0.01 and plated directly into assay-ready 384-well tissue culture plates with final OD_580_ values of the wells read on day 14. Data was normalized and MIC values calculated as written above.

Chromosomal DNA was isolated as previously described.^39^ Whole genome sequencing (WGS) libraries were prepared using the KAPA HyperPlus Kit (Roche catalog number 07962380001, 07962401001, and/or 07962428001) according to manufacturer’s instructions with the following modifications: in Step 1.5, fragmentation was carried out at 37 °C for 30 min; in Step A1.1, an additional 50 μL of molecular grade H_2_O was added to the 50 μL of DNA to be size-selected, and the volume of KAPA cleanup beads used was 70 μL, for a final volume of 170 μL per sample; in Step A1.6, the full 170 μL of supernatant from the first size cut was mixed with 20 μL of KAPA cleanup beads, for a final volume of 190 μL per sample. (5–10) × 10^6^ 50-bp paired-end reads were obtained for each sample on an Illumina HiSeq 4000 sequencer at 2 × 100 cycles pair-end reads. Postrun demultiplexing and adapter removal were performed by using bcl2fastq2 Conversion Software (Illumina). Per-cycle BCL base call files were converted into fastq files, and the fastq files were inspected using fastqc (http://www.bioinformatics.babraham.ac.uk/projects/fastqc). Trimmed fastq files were then aligned with the reference genome (*M. tuberculosis* str. H37RvCO; NCBI Reference Sequence: NZ_CM001515.1) using bwa mem.^40^ Bam files were sorted and merged using samtools.^41^ Read groups were added and bam files deduplicated using Picard tools, and GATK best-practices were followed for SNP and indel detection.^42^

### Co-Crystallization and X-ray Crystallography

Purified Pks13-TE with cleaved His-tag at concentration 5 mg/mL was mixed with inhibitors out of 100% DMSO stock solution at final concentration 250–500 μM and 5% DMSO. Hanging drops were set by adding 1:1 volume ratio of protein-inhibitor solution with mother liquor consisting of 0.1 M Tris-HCl pH 8.5, 1.8–2 M AmSO4 and 2–5% of PPG P400.

X20403, X20419, and X20348 bound crystals were collected at the 23ID beamline of Argonne National Lab synchrotron, and the X20404 bound crystal was collected at BL502 beamline of Lawrence Berkley National Lab synchrotron. Data were indexed, integrated, and scaled by the beamline autoprocessing pipeline: XDS,^43^ POINTLESS,^44^ and AIMLESS^45^ software packages. The structure was solved by molecular replacement with Molrep software^46^ using 5V3Y as a search model. This was followed by iterative cycles of refinement with PHENIX.REFINE^47^ and manual building in COOT.^48^ A ligand model and restraints were generated using the eLBOW tool.^49^ Final rounds of refinement were done with PDB-REDO.^50^ The data collection and refinement statistics are listed in Table S6.

### LC-MS/MS Analytical Methods for PK Parameter Assessments

Neat 1 mg/mL DMSO stock of X20404 was serially diluted in 50/50 acetonitrile (ACN)/Milli-Q water to create standard curves solutions. Standards were created by adding 10 μL of spiking solutions to 90 μL of drug-free plasma (CD-1 K2EDTA Mouse, Bioreclamation IVT). Five μL of control, standard, or study sample were added to 100 μL of ACN protein precipitation solvent containing 10 ng/mL of the internal standard Verapamil (Sigma-Aldrich). Extracts were vortexed for 5 min and centrifuged at 4000 rpm for 5 min. 75 μL of supernatant was transferred for HPLC-MS/MS analysis and diluted with 75 μL of Milli-Q deionized water.

LC-MS/MS analysis was performed on a Sciex Applied Biosystems Qtrap 6500+ triple-quadrupole mass spectrometer coupled to a Shimadzu Nexera X2 UHPLC system to quantify each drug in the plasma. Chromatography was performed on an Agilent SB-C8 (2.1 × 30 mm; particle size, 3.5 μm) using a reverse-phase gradient. Milli-Q deionized water with 0.1% formic acid was used for the aqueous mobile phase, and 0.1% formic acid in ACN was used for the organic mobile phase. Multiple-reaction monitoring of parent-daughter transitions in electrospray positive-ionization mode was used to quantify all analytes. The following MRM transitions were used for X20404 (458.08/254.00) and Verapamil (455.40/165.00). Sample analysis was accepted if the concentrations of the quality control samples were within 20% of the nominal concentration. Data processing was performed using Analyst software (version 1.6.2; Applied Biosystems Sciex).

### Plasma Protein Binding

DMSO stocks were spiked into plasma to a concentration of 10,000 ng/mL. 200 μL of spiked plasma was pipetted into the sample chamber of the rapid equilibrium dialysis (RED) cartridge. 350 μL of PBS was added to the adjacent cartridge. The plate containing the RED inserts was sealed and incubated at 37 °C on the thermomixer at 300 rpm for 4 h. After incubation, 50 μL aliquots of plasma were removed and added to 50 μL of blank plasma in a deep well plate (1:1). Similarly, 50 μL aliquots of PBS were removed and added to 50 μL of blank plasma. This created an identical matrix between buffer and nonbuffer samples. Samples were processed and quantified as specified in the LC-MS/MS analytical method.

### Metabolic Stability in Mouse Liver Microsomes (MLMs)

50 mM X20404 and control compound verapamil in DMSO (50:50) were prepared and diluted in 50/50 ACN/H_2_O to a final concentration of 500 μM. 100 μL of 200 μM sodium phosphate buffer, 20 μL of 50 mM MgCl_2_, 10 μL of 20 mg/mL mouse liver microsomes (Xenotech), 48 μL of Milli-Q Water, and 2 μL of 500 μM compound stock were combined. The mixture was allowed to incubate at 37 °C while mixing at 300 rpm uncovered on a thermomixer. After 5 min of incubation, 20 μL of 10 mM NADPH was added to activate microsomal metabolism or 20 μL of Milli-Q water was added to the no-NADPH controls, and samples were mixed by gentle aspiration and dispense. Time points were taken at 0, 5, 15, 30, and 45 min. At each time point 10 μL of mixture was pipetted into 100 μL of ACN containing 100 ng/mL Labetalol to quench the reaction and extract the compounds. Extracts were vortexed for 5 min and subsequently centrifuged at 4000 rpm for 5 min. 75 μL of extract was combined with 75 μL of Milli-Q water for LC-MS/MS analysis, performed as previously specified. The Analyst software was used to measure peak areas. The peak area ratio (Analyte/IS) was plotted vs time, and a half-life (*t*_1/2_) was determined using PK solver Excel add-in. The intrinsic clearance (Cl_int_) was calculated accordingly as Cl_int_ = microsomal protein concentration (V) × 0.693/*t*_1/2_.

### Pharmacokinetics Studies

Mouse experiments were ethically reviewed and approved by the Institutional Animal Care and Use committee of Hackensack Meridian Health. CD-1 female mice (22–25 g) were used in the oral pharmacokinetic studies. X20404 was initially administered as a single intravenous (IV) 5 mg/kg dose in 4% Cremophor EL or an oral gavage (PO) 25 mg/kg dose in 5% dimethylacetamide: 60% PEG300:35% - 5% Dextrose in water. Serial blood samples (50 μL) were taken by puncture of the lateral tail vein from each mouse (n = 3 per route and dose) at 1 min, 15 min, 1, 3, 7, and 24 h post dose for IV and 30 min, 1, 3, and 5 h postdose for PO. Blood was captured in CB300 blood collection tubes containing K_2_EDTA and stored on ice. Plasma was recovered after centrifugation and stored at −80 °C until it was analyzed by high-pressure liquid chromatography coupled to tandem mass spectrometry (LC-MS/MS). Dose-escalation pharmacokinetic profiling of X20404 in 20% Captisol was performed at doses of 25, 50, 100, and 200 mg/kg after 5 days of oral daily dosing to assess exposure linearity and tolerability. X20404 was solubilized in 20% Captisol. Pharmacokinetic time points were taken on day 5 of dosing at 0.5, 1, 3, 5, 7, and 24 h post dose and processed as specified above. Pharmacokinetic parameters were determined with the PK Solver Excel add-in using noncompartmental pharmacokinetic analysis.

### Mouse Efficacy Studies

Nine-week-old female BALB/c mice were infected with Mtb H37Rv with a Glas-Col inhalation system. An inoculum of 1 × 10^7^ cfu/ml bacteria was added to the nebulizer, and the initial bacterial lung load was determined by sacrificing four mice after 3 h of infection. After 2 weeks, five mice were sacrificed to determine the bacterial burden in the lungs before the start of the treatment. Mice were treated daily with 100 mg/kg X20404, 200 mg/kg X20404, 25 mg/kg isoniazid, or vehicle control administered by oral gavage daily 7 days a week for 2 weeks. X20404 was formulated in 20% Captisol, and INH was formulated in water. The mice were then sacrificed, the lungs harvested and homogenized, and serial dilutions of the homogenates were spread on Middlebrook 7H11 agar supplemented with 10% OADC and 0.5% glycerol. Plates were inculcated at 37 °C for 3–4 weeks prior to counting.

## Supporting Information Available

Target protein quality control, Fo-Fc electron difference density Polder omit map, DEL screening conditions, summary of the number and behavior of hit series from the Pks13 DEL screen, SAR of selected analogues of Series 1 and Series 2, Series 3, data collection and refinement statistics, sequencing results (PDF)
Synthesis of compounds used (PDF)

## References

[ref1] Dean A. S.; Tosas Auguet O.; Glaziou P.; Zignol M.; Ismail N.; Kasaeva T.; Floyd K. 25 Years of Surveillance of Drug-Resistant Tuberculosis: Achievements, Challenges, and Way Forward. Lancet Infect Dis 2022, 22 (7), e191–e196. 10.1016/S1473-3099(21)00808-2. 35248168 PMC8893725

[ref2] Song M.; Hwang G. T. DNA-Encoded Library Screening as Core Platform Technology in Drug Discovery: Its Synthetic Method Development and Applications in DEL Synthesis. J. Med. Chem. 2020, 63 (13), 6578–6599. 10.1021/acs.jmedchem.9b01782. 32039601

[ref3] Clark M. A.; Acharya R. A.; Arico-Muendel C. C.; Belyanskaya S. L.; Benjamin D. R.; Carlson N. R.; Centrella P. A.; Chiu C. H.; Creaser S. P.; Cuozzo J. W.; Davie C. P.; Ding Y.; Franklin G. J.; Franzen K. D.; Gefter M. L.; Hale S. P.; Hansen N. J. V.; Israel D. I.; Jiang J.; Kavarana M. J.; Kelley M. S.; Kollmann C. S.; Li F.; Lind K.; Mataruse S.; Medeiros P. F.; Messer J. A.; Myers P.; O’Keefe H.; Oliff M. C.; Rise C. E.; Satz A. L.; Skinner S. R.; Svendsen J. L.; Tang L.; van Vloten K.; Wagner R. W.; Yao G.; Zhao B.; Morgan B. Design, Synthesis and Selection of DNA-Encoded Small-Molecule Libraries. Nat. Chem. Biol. 2009, 5 (9), 647–654. 10.1038/nchembio.211. 19648931

[ref4] Goodnow R. A.; Dumelin C. E.; Keefe A. D. DNA-Encoded Chemistry: Enabling the Deeper Sampling of Chemical Space. Nat. Rev. Drug Discov 2017, 16 (2), 131–147. 10.1038/nrd.2016.213. 27932801

[ref5] Machutta C. A.; Kollmann C. S.; Lind K. E.; Bai X.; Chan P. F.; Huang J.; Ballell L.; Belyanskaya S.; Besra G. S.; Barros-Aguirre D.; Bates R. H.; Centrella P. A.; Chang S. S.; Chai J.; Choudhry A. E.; Coffin A.; Davie C. P.; Deng H.; Deng J.; Ding Y.; Dodson J. W.; Fosbenner D. T.; Gao E. N.; Graham T. L.; Graybill T. L.; Ingraham K.; Johnson W. P.; King B. W.; Kwiatkowski C. R.; Lelièvre J.; Li Y.; Liu X.; Lu Q.; Lehr R.; Mendoza-Losana A.; Martin J.; McCloskey L.; McCormick P.; O’Keefe H. P.; O’Keeffe T.; Pao C.; Phelps C. B.; Qi H.; Rafferty K.; Scavello G. S.; Steiginga M. S.; Sundersingh F. S.; Sweitzer S. M.; Szewczuk L. M.; Taylor A.; Toh M. F.; Wang J.; Wang M.; Wilkins D. J.; Xia B.; Yao G.; Zhang J.; Zhou J.; Donahue C. P.; Messer J. A.; Holmes D.; Arico-Muendel C. C.; Pope A. J.; Gross J. W.; Evindar G. Prioritizing Multiple Therapeutic Targets in Parallel Using Automated DNA-Encoded Library Screening. Nat. Commun. 2017, 8, 16081 10.1038/ncomms16081. 28714473 PMC5520047

[ref6] Encinas L.; O’Keefe H.; Neu M.; Remuiñán M. J.; Patel A. M.; Guardia A.; Davie C. P.; Pérez-Macías N.; Yang H.; Convery M. A.; Messer J. A.; Pérez-Herrán E.; Centrella P. A.; Alvarez-Gómez D.; Clark M. A.; Huss S.; O’Donovan G. K.; Ortega-Muro F.; McDowell W.; Castañeda P.; Arico-Muendel C. C.; Pajk S.; Rullás J.; Angulo-Barturen I.; Alvarez-Ruíz E.; Mendoza-Losana A.; Ballell Pages L.; Castro-Pichel J.; Evindar G. Encoded Library Technology as a Source of Hits for the Discovery and Lead Optimization of a Potent and Selective Class of Bactericidal Direct Inhibitors of Mycobacterium Tuberculosis. InhA. J. Med. Chem. 2014, 57 (4), 1276–1288. 10.1021/jm401326j. 24450589

[ref7] Soutter H. H.; Centrella P.; Clark M. A.; Cuozzo J. W.; Dumelin C. E.; Guie M.-A.; Habeshian S.; Keefe A. D.; Kennedy K. M.; Sigel E. A.; Troast D. M.; Zhang Y.; Ferguson A. D.; Davies G.; Stead E. R.; Breed J.; Madhavapeddi P.; Read J. A. Discovery of Cofactor-Specific, Bactericidal Mycobacterium Tuberculosis InhA Inhibitors Using DNA-Encoded Library Technology. Proc. Natl. Acad. Sci. U. S. A. 2016, 113 (49), E7880–E7889. 10.1073/pnas.1610978113. 27864515 PMC5150407

[ref8] Batt S. M.; Minnikin D. E.; Besra G. S. The Thick Waxy Coat of Mycobacteria, a Protective Layer against Antibiotics and the Host’s Immune System. Biochem. J. 2020, 477 (10), 1983–2006. 10.1042/BCJ20200194. 32470138 PMC7261415

[ref9] Marrakchi H.; Lanéelle M.-A.; Daffé M. Mycolic Acids: Structures, Biosynthesis, and Beyond. Chem. Biol. 2014, 21 (1), 67–85. 10.1016/j.chembiol.2013.11.011. 24374164

[ref10] Rozwarski D. A.; Grant G. A.; Barton D. H.; Jacobs W. R.; Sacchettini J. C. Modification of the NADH of the Isoniazid Target (InhA) from Mycobacterium Tuberculosis. Science 1998, 279 (5347), 98–102. 10.1126/science.279.5347.98. 9417034

[ref11] Larsen M. H.; Vilchèze C.; Kremer L.; Besra G. S.; Parsons L.; Salfinger M.; Heifets L.; Hazbon M. H.; Alland D.; Sacchettini J. C.; Jacobs W. R. Overexpression of inhA, but Not kasA, Confers Resistance to Isoniazid and Ethionamide in Mycobacterium Smegmatis, M. Bovis BCG and M. Tuberculosis. Mol. Microbiol. 2002, 46 (2), 453–466. 10.1046/j.1365-2958.2002.03162.x. 12406221

[ref12] Kim S. K.; Dickinson M. S.; Finer-Moore J.; Guan Z.; Kaake R. M.; Echeverria I.; Chen J.; Pulido E. H.; Sali A.; Krogan N. J.; Rosenberg O. S.; Stroud R. M. Structure and Dynamics of the Essential Endogenous Mycobacterial Polyketide Synthase Pks13. Nat. Struct Mol. Biol. 2023, 30 (3), 296–308. 10.1038/s41594-022-00918-0. 36782050 PMC10312659

[ref13] Gavalda S.; Bardou F.; Laval F.; Bon C.; Malaga W.; Chalut C.; Guilhot C.; Mourey L.; Daffé M.; Quémard A. The Polyketide Synthase Pks13 Catalyzes a Novel Mechanism of Lipid Transfer in Mycobacteria. Chem. Biol. 2014, 21 (12), 1660–1669. 10.1016/j.chembiol.2014.10.011. 25467124

[ref14] DeJesus M. A.; Gerrick E. R.; Xu W.; Park S. W.; Long J. E.; Boutte C. C.; Rubin E. J.; Schnappinger D.; Ehrt S.; Fortune S. M.; Sassetti C. M.; Ioerger T. R. Comprehensive Essentiality Analysis of the Mycobacterium Tuberculosis Genome via Saturating Transposon Mutagenesis. mBio 2017, 8 (1), e02133–16. 10.1128/mBio.02133-16. 28096490 PMC5241402

[ref15] Long J. E.; DeJesus M.; Ward D.; Baker R. E.; Ioerger T.; Sassetti C. M. Identifying Essential Genes in Mycobacterium Tuberculosis by Global Phenotypic Profiling. Methods Mol. Biol. 2015, 1279, 79–95. 10.1007/978-1-4939-2398-4_6. 25636614

[ref16] Bosch B.; DeJesus M. A.; Poulton N. C.; Zhang W.; Engelhart C. A.; Zaveri A.; Lavalette S.; Ruecker N.; Trujillo C.; Wallach J. B.; Li S.; Ehrt S.; Chait B. T.; Schnappinger D.; Rock J. M. Genome-Wide Gene Expression Tuning Reveals Diverse Vulnerabilities of M. Tuberculosis. Cell 2021, 184 (17), 4579–4592.e24. 10.1016/j.cell.2021.06.033. 34297925 PMC8382161

[ref17] Meade R. K.; Long J. E.; Jinich A.; Rhee K. Y.; Ashbrook D. G.; Williams R. W.; Sassetti C. M.; Smith C. M. Genome-Wide Screen Identifies Host Loci That Modulate Mycobacterium Tuberculosis Fitness in Immunodivergent Mice. G3 (Bethesda) 2023, 13 (9), jkad147 10.1093/g3journal/jkad147. 37405387 PMC10468300

[ref18] Aggarwal A.; Parai M. K.; Shetty N.; Wallis D.; Woolhiser L.; Hastings C.; Dutta N. K.; Galaviz S.; Dhakal R. C.; Shrestha R.; Wakabayashi S.; Walpole C.; Matthews D.; Floyd D.; Scullion P.; Riley J.; Epemolu O.; Norval S.; Snavely T.; Robertson G. T.; Rubin E. J.; Ioerger T. R.; Sirgel F. A.; van der Merwe R.; van Helden P. D.; Keller P.; Böttger E. C.; Karakousis P. C.; Lenaerts A. J.; Sacchettini J. C. Development of a Novel Lead That Targets M. Tuberculosis Polyketide Synthase 13. Cell 2017, 170 (2), 249–259. 10.1016/j.cell.2017.06.025. 28669536 PMC5509550

[ref19] Zhang W.; Lun S.; Wang S.-H.; Jiang X.-W.; Yang F.; Tang J.; Manson A. L.; Earl A. M.; Gunosewoyo H.; Bishai W. R.; Yu L.-F. Identification of Novel Coumestan Derivatives as Polyketide Synthase 13 Inhibitors against Mycobacterium Tuberculosis. J. Med. Chem. 2018, 61 (3), 791–803. 10.1021/acs.jmedchem.7b01319. 29328655

[ref20] Zhang W.; Lun S.; Wang S.-S.; Cai Y.-P.; Yang F.; Tang J.; Bishai W. R.; Yu L.-F. Structure-Based Optimization of Coumestan Derivatives as Polyketide Synthase 13-Thioesterase(Pks13-TE) Inhibitors with Improved hERG Profiles for Mycobacterium Tuberculosis Treatment. J. Med. Chem. 2022, 65 (19), 13240–13252. 10.1021/acs.jmedchem.2c01064. 36174223

[ref21] Lun S.; Xiao S.; Zhang W.; Wang S.; Gunosewoyo H.; Yu L.-F.; Bishai W. R. Therapeutic Potential of Coumestan Pks13 Inhibitors for Tuberculosis. Antimicrob. Agents Chemother. 2021, 65 (5), e02190 10.1128/AAC.02190-20. PMC8092898 33558290

[ref22] Wilson C.; Ray P.; Zuccotto F.; Hernandez J.; Aggarwal A.; Mackenzie C.; Caldwell N.; Taylor M.; Huggett M.; Mathieson M.; Murugesan D.; Smith A.; Davis S.; Cocco M.; Parai M. K.; Acharya A.; Tamaki F.; Scullion P.; Epemolu O.; Riley J.; Stojanovski L.; Lopez-Román E. M.; Torres-Gómez P. A.; Toledo A. M.; Guijarro-Lopez L.; Camino I.; Engelhart C. A.; Schnappinger D.; Massoudi L. M.; Lenaerts A.; Robertson G. T.; Walpole C.; Matthews D.; Floyd D.; Sacchettini J. C.; Read K. D.; Encinas L.; Bates R. H.; Green S. R.; Wyatt P. G. Optimization of TAM16, a Benzofuran That Inhibits the Thioesterase Activity of Pks13; Evaluation toward a Preclinical Candidate for a Novel Antituberculosis Clinical Target. J. Med. Chem. 2022, 65 (1), 409–423. 10.1021/acs.jmedchem.1c01586. 34910486 PMC8762665

[ref23] Taira J.; Murakami K.; Monobe K.; Kuriki K.; Fujita M.; Ochi Y.; Sakamoto H.; Aoki S. Identification of Novel Inhibitors for Mycobacterial Polyketide Synthase 13 via in Silico Drug Screening Assisted by the Parallel Compound Screening with Genetic Algorithm-Based Programs. J. Antibiot (Tokyo) 2022, 75 (10), 552–558. 10.1038/s41429-022-00549-z. 35941150

[ref24] Wang X.; Zhao W.; Wang B.; Ding W.; Guo H.; Zhao H.; Meng J.; Liu S.; Lu Y.; Liu Y.; Zhang D. Identification of Inhibitors Targeting Polyketide Synthase 13 of Mycobacterium Tuberculosis as Antituberculosis Drug Leads. Bioorg Chem. 2021, 114, 105110 10.1016/j.bioorg.2021.105110. 34175719

[ref25] Zhang W.; Liu L.-L.; Lun S.; Wang S.-S.; Xiao S.; Gunosewoyo H.; Yang F.; Tang J.; Bishai W. R.; Yu L.-F. Design and Synthesis of Mycobacterial Pks13 Inhibitors: Conformationally Rigid Tetracyclic Molecules. Eur. J. Med. Chem. 2021, 213, 113202 10.1016/j.ejmech.2021.113202. 33516983 PMC8689393

[ref26] Wilson R.; Kumar P.; Parashar V.; Vilchèze C.; Veyron-Churlet R.; Freundlich J. S.; Barnes S. W.; Walker J. R.; Szymonifka M. J.; Marchiano E.; Shenai S.; Colangeli R.; Jacobs W. R.; Neiditch M. B.; Kremer L.; Alland D. Antituberculosis Thiophenes Define a Requirement for Pks13 in Mycolic Acid Biosynthesis. Nat. Chem. Biol. 2013, 9 (8), 499–506. 10.1038/nchembio.1277. 23770708 PMC3720791

[ref27] Maddry J. A.; Ananthan S.; Goldman R. C.; Hobrath J. V.; Kwong C. D.; Maddox C.; Rasmussen L.; Reynolds R. C.; Secrist J. A.; Sosa M. I.; White E. L.; Zhang W. Antituberculosis Activity of the Molecular Libraries Screening Center Network Library. Tuberculosis (Edinb) 2009, 89 (5), 354–363. 10.1016/j.tube.2009.07.006. 19783214 PMC2792876

[ref28] Thanna S.; Knudson S. E.; Grzegorzewicz A.; Kapil S.; Goins C. M.; Ronning D. R.; Jackson M.; Slayden R. A.; Sucheck S. J. Synthesis and Evaluation of New 2-Aminothiophenes against Mycobacterium Tuberculosis. Org. Biomol Chem. 2016, 14 (25), 6119–6133. 10.1039/C6OB00821F. 27251120 PMC4918453

[ref29] Green S. R.; Wilson C.; Eadsforth T. C.; Punekar A. S.; Tamaki F. K.; Wood G.; Caldwell N.; Forte B.; Norcross N. R.; Kiczun M.; Post J. M.; Lopez-Román E. M.; Engelhart C. A.; Lukac I.; Zuccotto F.; Epemolu O.; Boshoff H. I. M.; Schnappinger D.; Walpole C.; Gilbert I. H.; Read K. D.; Wyatt P. G.; Baragaña B. Identification and Optimization of Novel Inhibitors of the Polyketide Synthase 13 Thioesterase Domain with Antitubercular Activity. J. Med. Chem. 2023, 66 (22), 15380–15408. 10.1021/acs.jmedchem.3c01514. 37948640 PMC10683028

[ref30] Simon G. M.; Cravatt B. F. Activity-Based Proteomics of Enzyme Superfamilies: Serine Hydrolases as a Case Study. J. Biol. Chem. 2010, 285 (15), 11051–11055. 10.1074/jbc.R109.097600. 20147750 PMC2856978

[ref31] Bachovchin D. A.; Brown S. J.; Rosen H.; Cravatt B. F. Identification of Selective Inhibitors of Uncharacterized Enzymes by High-Throughput Screening with Fluorescent Activity-Based Probes. Nat. Biotechnol. 2009, 27 (4), 387–394. 10.1038/nbt.1531. 19329999 PMC2709489

[ref32] Bachovchin D. A.; Mohr J. T.; Speers A. E.; Wang C.; Berlin J. M.; Spicer T. P.; Fernandez-Vega V.; Chase P.; Hodder P. S.; Schürer S. C.; Nomura D. K.; Rosen H.; Fu G. C.; Cravatt B. F. Academic Cross-Fertilization by Public Screening Yields a Remarkable Class of Protein Phosphatase Methylesterase-1 Inhibitors. Proc. Natl. Acad. Sci. U. S. A. 2011, 108 (17), 6811–6816. 10.1073/pnas.1015248108. 21398589 PMC3084096

[ref33] Rohe A.; Henze C.; Erdmann F.; Sippl W.; Schmidt M. A Fluorescence Anisotropy-Based Myt1 Kinase Binding Assay. Assay Drug Dev Technol. 2014, 12 (2), 136–144. 10.1089/adt.2013.534. 24229357

[ref34] Hall M. D.; Yasgar A.; Peryea T.; Braisted J. C.; Jadhav A.; Simeonov A.; Coussens N. P. Fluorescence Polarization Assays in High-Throughput Screening and Drug Discovery: A Review. Methods Appl. Fluoresc 2016, 4 (2), 022001 10.1088/2050-6120/4/2/022001. 28809163 PMC5563979

[ref35] Bergval I. L.; Schuitema A. R. J.; Klatser P. R.; Anthony R. M. Resistant Mutants of Mycobacterium Tuberculosis Selected in Vitro Do Not Reflect the in Vivo Mechanism of Isoniazid Resistance. J. Antimicrob. Chemother. 2009, 64 (3), 515–523. 10.1093/jac/dkp237. 19578178 PMC2724981

[ref36] Puckett S.; Trujillo C.; Wang Z.; Eoh H.; Ioerger T. R.; Krieger I.; Sacchettini J.; Schnappinger D.; Rhee K. Y.; Ehrt S. Glyoxylate Detoxification Is an Essential Function of Malate Synthase Required for Carbon Assimilation in Mycobacterium Tuberculosis. Proc. Natl. Acad. Sci. U. S. A. 2017, 114 (11), E2225–E2232. 10.1073/pnas.1617655114. 28265055 PMC5358392

[ref37] Beites T.; Jansen R. S.; Wang R.; Jinich A.; Rhee K. Y.; Schnappinger D.; Ehrt S. Multiple Acyl-CoA Dehydrogenase Deficiency Kills Mycobacterium Tuberculosis in Vitro and during Infection. Nat. Commun. 2021, 12 (1), 6593 10.1038/s41467-021-26941-1. 34782606 PMC8593149

[ref38] Lea W. A.; Simeonov A. Fluorescence Polarization Assays in Small Molecule Screening. Expert Opin Drug Discov 2011, 6 (1), 17–32. 10.1517/17460441.2011.537322. 22328899 PMC3277431

[ref39] Green S. R.; Davis S. H.; Damerow S.; Engelhart C. A.; Mathieson M.; Baragaña B.; Robinson D. A.; Tamjar J.; Dawson A.; Tamaki F. K.; Buchanan K. I.; Post J.; Dowers K.; Shepherd S. M.; Jansen C.; Zuccotto F.; Gilbert I. H.; Epemolu O.; Riley J.; Stojanovski L.; Osuna-Cabello M.; Pérez-Herrán E.; Rebollo M. J.; Guijarro López L.; Casado Castro P.; Camino I.; Kim H. C.; Bean J. M.; Nahiyaan N.; Rhee K. Y.; Wang Q.; Tan V. Y.; Boshoff H. I. M.; Converse P. J.; Li S.-Y.; Chang Y. S.; Fotouhi N.; Upton A. M.; Nuermberger E. L.; Schnappinger D.; Read K. D.; Encinas L.; Bates R. H.; Wyatt P. G.; Cleghorn L. A. T. Lysyl-tRNA Synthetase, a Target for Urgently Needed M. Tuberculosis Drugs. Nat. Commun. 2022, 13 (1), 5992 10.1038/s41467-022-33736-5. 36220877 PMC9552147

[ref40] Li H.; Durbin R. Fast and Accurate Short Read Alignment with Burrows-Wheeler Transform. Bioinformatics 2009, 25 (14), 1754–1760. 10.1093/bioinformatics/btp324. 19451168 PMC2705234

[ref41] Li H.; Handsaker B.; Wysoker A.; Fennell T.; Ruan J.; Homer N.; Marth G.; Abecasis G.; Durbin R. 1000 Genome Project Data Processing Subgroup. The Sequence Alignment/Map Format and SAMtools.. Bioinformatics 2009, 25 (16), 2078–2079. 10.1093/bioinformatics/btp352. 19505943 PMC2723002

[ref42] DePristo M. A.; Banks E.; Poplin R.; Garimella K. V.; Maguire J. R.; Hartl C.; Philippakis A. A.; del Angel G.; Rivas M. A.; Hanna M.; McKenna A.; Fennell T. J.; Kernytsky A. M.; Sivachenko A. Y.; Cibulskis K.; Gabriel S. B.; Altshuler D.; Daly M. J. A Framework for Variation Discovery and Genotyping Using Next-Generation DNA Sequencing Data. Nat. Genet. 2011, 43 (5), 491–498. 10.1038/ng.806. 21478889 PMC3083463

[ref43] Kabsch W. XDS. Acta Crystallogr. D Biol. Crystallogr. 2010, 66 (2), 125–132. 10.1107/S0907444909047337. 20124692 PMC2815665

[ref44] Evans P. Scaling and Assessment of Data Quality. Acta Crystallogr. D Biol. Crystallogr. 2006, 62 (1), 72–82. 10.1107/S0907444905036693. 16369096

[ref45] Evans P. R.; Murshudov G. N. How Good Are My Data and What Is the Resolution?. Acta Crystallogr. D Biol. Crystallogr. 2013, 69 (7), 1204–1214. 10.1107/S0907444913000061. 23793146 PMC3689523

[ref46] Vagin A.; Teplyakov A. Molecular Replacement with MOLREP. Acta Crystallogr. D Biol. Crystallogr. 2010, 66 (1), 22–25. 10.1107/S0907444909042589. 20057045

[ref47] Liebschner D.; Afonine P. V.; Baker M. L.; Bunkóczi G.; Chen V. B.; Croll T. I.; Hintze B.; Hung L. W.; Jain S.; McCoy A. J.; Moriarty N. W.; Oeffner R. D.; Poon B. K.; Prisant M. G.; Read R. J.; Richardson J. S.; Richardson D. C.; Sammito M. D.; Sobolev O. V.; Stockwell D. H.; Terwilliger T. C.; Urzhumtsev A. G.; Videau L. L.; Williams C. J.; Adams P. D. Macromolecular Structure Determination Using X-Rays, Neutrons and Electrons: Recent Developments in Phenix. Acta Crystallogr. D Struct Biol. 2019, 75 (10), 861–877. 10.1107/S2059798319011471. 31588918 PMC6778852

[ref48] Emsley P.; Cowtan K. Coot: Model-Building Tools for Molecular Graphics. Acta Crystallogr. D Biol. Crystallogr. 2004, 60 (12), 2126–2132. 10.1107/S0907444904019158. 15572765

[ref49] Moriarty N. W.; Grosse-Kunstleve R. W.; Adams P. D. Electronic Ligand Builder and Optimization Workbench (eLBOW): A Tool for Ligand Coordinate and Restraint Generation. Acta Crystallogr. D Biol. Crystallogr. 2009, 65 (10), 1074–1080. 10.1107/S0907444909029436. 19770504 PMC2748967

[ref50] Joosten R. P.; Long F.; Murshudov G. N.; Perrakis A. The PDB_REDO Server for Macromolecular Structure Model Optimization. IUCrJ. 2014, 1 (4), 213–220. 10.1107/S2052252514009324. 25075342 PMC4107921

