# Supplementary material for: Inhibitors of the Thioesterase Activity of Mycobacterium tuberculosis Pks13 Discovered Using DNA-Encoded Chemical Library Screening
Source: ACS Infect Dis. 2024 Apr 5;10(5):1561–75. doi: 10.1021/acsinfecdis.3c00592 (PMC11091879; doi:10.1021/acsinfecdis.3c00592)
Supplement: Supplementary file 1 — id3c00592_si_001.pdf [file id3c00592_si_001.pdf]

Supporting Information

Inhibitors of the Thioesterase Activity of Mycobacterium tuberculosis Pks13 Discovered using DNA-Encoded Chemical Library Screening

Inna V. Krieger[1], Subbarao Yalamanchili[2], Paige Dickson[2], Curtis A. Engelhart[7], Matthew D Zimmerman[8], Jeremy Wood[1], Ethan Clary[1], Jasmine Nguyen[1], Natalie Thornton[7], Paolo A. Centrella[2], Betty Chan[2,10], John W Cuozzo[2,3], Martin Gengenbacher[8], Marie-Aude Guié[2], John P Guilinger[2], Corey Bienstock[2], Hajnalka Hartl[2,9], Christopher D. Hupp[2,4], Rachael Jetson[2,5], Takashi Satoh[2,6], John T. S. Yeoman[2,11], Ying Zhang[2], Veronique Dartois[8], Dirk Schnappinger[7], Anthony D. Keefe[2]*, James C. Sacchettini[1]*

* corresponding authors

Email: Anthony D. Keefe akeefe@x-chemrx.com, James C. Sacchettini sacchett@tamu.edu

Affiliations:

1 Department of Biochemistry & Biophysics, Texas A&M University, College Station, Texas, 77843, USA

2 X-Chem Inc., 100 Beaver Street, Waltham, Massachusetts, 02453, USA

3 Relay Therapeutics, 399 Binney Street, Cambridge, Massachusetts, 02141, USA.

4 Ipsen Bioscience Inc., 1 Main Street, Cambridge, Massachusetts, 02142, USA.

5 Valo Health, 75 Hayden Avenue, Lexington, Massachusetts, 02141, USA.

6 EXO Therapeutics, 150 Cambridgepark Drive, suite 300, Cambridge, Massachusetts, 02140, USA.

7 Department of Microbiology and Immunology, Weill Cornell Medicine, New York, New York, 10021, USA.

8 Center for Discovery and Innovation, Hackensack Meridian Health, Nutley, New Jersey, 07110, USA

Hackensack Meridian School of Medicine, Hackensack Meridian Health, Nutley, New Jersey, 07110, USA

9 Orogen Therapeutics, 12 Gill Street, Woburn, Massachusetts, 01801, USA.

10 Auron Therapeutics, 55 Chapel Street, Newton, Massachusetts, 02458, USA.

11 Recludix Pharmaceuticals, 222 Third Street, Cambridge, Massachusetts, 02142, USA.

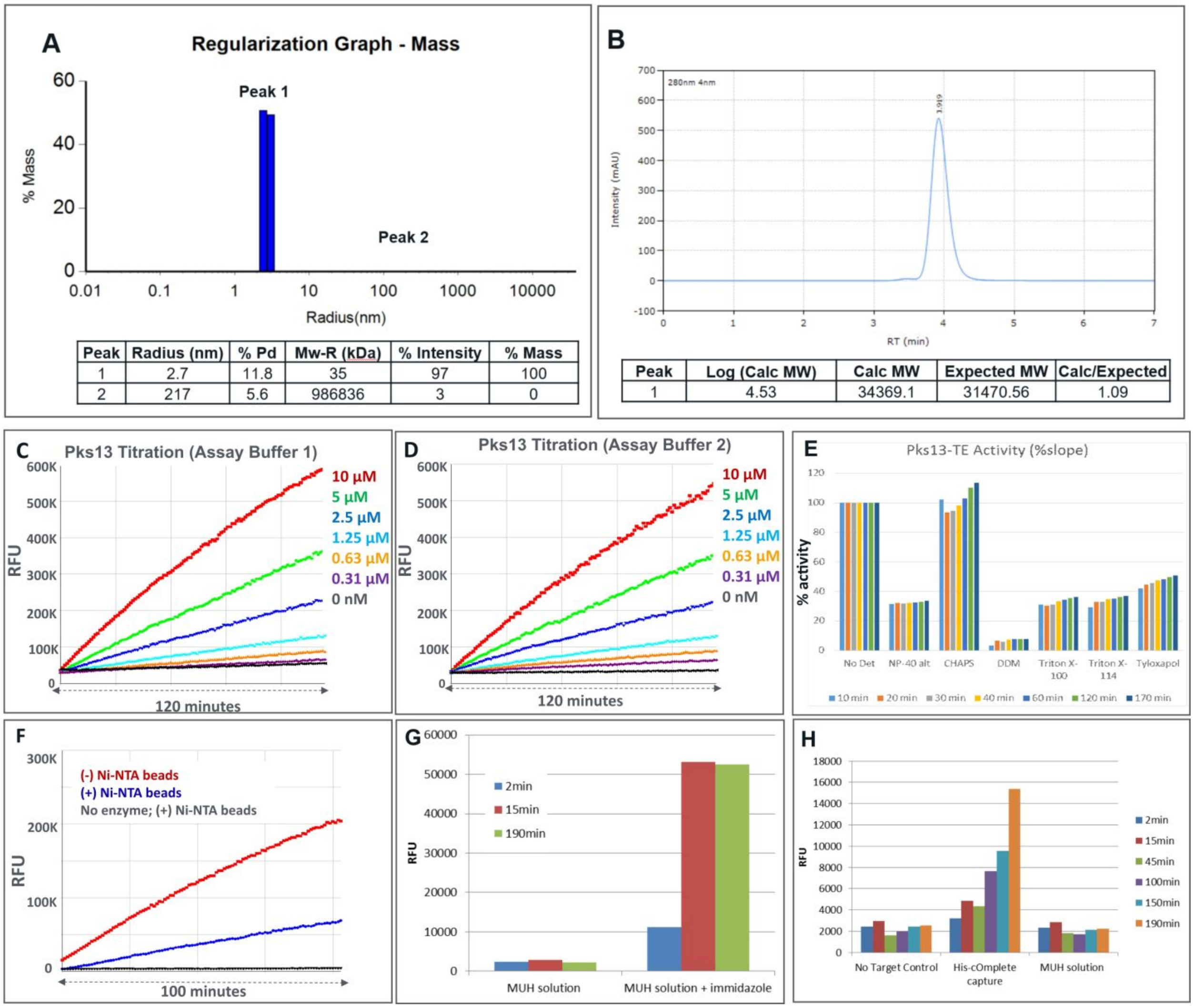

Figure S1 Target protein quality control

A. Dynamic Light Scattering analysis of Pks13. No aggregates are observed and the molecular weight corresponds to a monomer.
B. SEC-MALS characterization of Pks13. The protein elutes as a single, clean peak with molecular weight corresponding to the monomer.

C. Pks13 protein titration in the originally established assay buffer
D. Pks13 protein titration in an alternative buffer to be used for DEL screening
E. Assay buffer optimization for Pks13 activity. Using an enzymatic turnover assay in Buffer 1 containing various detergents, it was determined that CHAPS was the optimal buffer additive for DEL screening
F. Pks13 is inhibited by Ni-NTA
G. Assay substrate 2-MUH is hydrolyzed in the presence of imidazole, resulting in high assay background signal
H. Immobilized protein activity under the optimized conditions

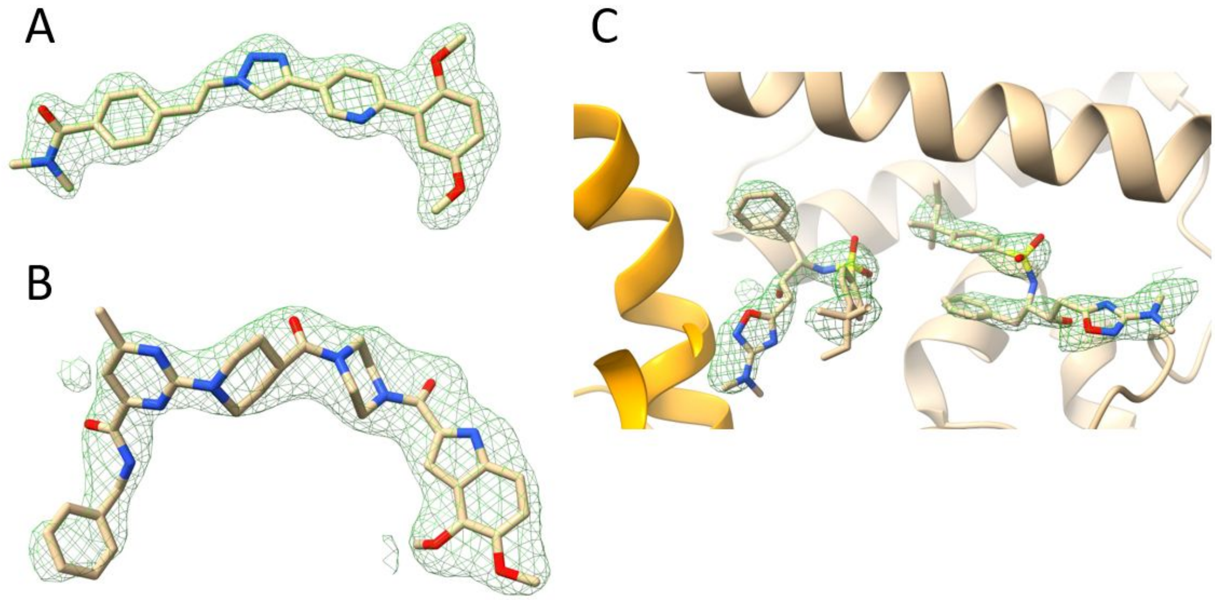

Figure S2 Fo-Fc electron difference density Polder omit map for **A.** X20404 (contoured at 5 σ), **B.** X20419 (contoured at 4.5 σ) and **C.** X20348 (contoured at 4 σ), with two protein chains shown as ribbons in tan and gold.

Table S1: DEL screening conditions

	Construct	Resin	Conc (μM)	TAM16 (50μM)	Rationale
1	N/A	His-cOmplete	0		No target control
2	His-Pks13(TE domain)	His-cOmplete	10		Determine binders
3	His-Pks13(TE domain)	His-cOmplete	2		Determine affinity
4	His-Pks13(TE domain)	His-cOmplete	0.5		Determine affinity
5	His-Pks13(TE domain)	His-cOmplete	10	Yes	Binding site identity
6	His-Pks13(TE domain)	His-cOmplete	2	Yes	Binding site identity

Table S2: A summary of the number and behavior of hit series from the Pks13 DEL screen. Each hit series is assigned a single profile, which represents the enrichment of that series across each selection condition. Hit series which were found to be enriched with two or three concentrations of Pks13, and bound competitively with TAM16 were prioritized for hit resynthesis. Number of series indicates the number of different compound series that were identified in each selection profile.

	Observed enrichment of the series across each selection condition					
Profile	Pks13 (10uM)	Pks13 (2uM)	Pks13 (0.5uM)	Pks13 + TAM16	# of Series	Priority
1	+	+	+	-	29	High
2	+	+	-	-	235	High
3	+	-	-	-	211	Medium
4	+	Sometimes		+	5	Low

Table S3. SAR of selected analogs of Series 1 (X20404).

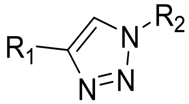

Entry	XID (Series)	R_1	R_2	Core	IC50 (uM)	WT H37Rv MIC (uM)	Hypermorph MIC fold shift (+ATc /WT)	Hypomorph MIC fold shift (WT/-ATc)
1	X20403	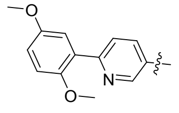	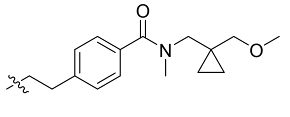	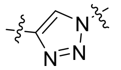	0.5	0.05	2.3	0.88
2	X20404	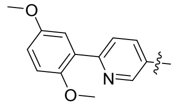	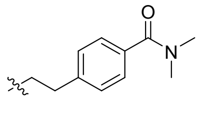	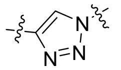	0.43	0.25	2.3	2.9
3	X21546	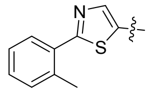	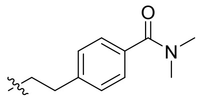	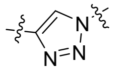	0.37	18	n.d.	1.9
4	X21464	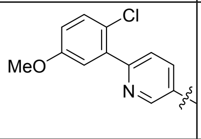	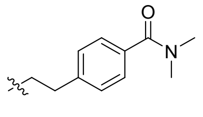	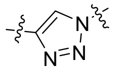	0.38	5.5	3.8	2.4
5	X21434	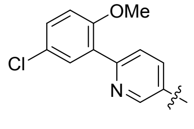	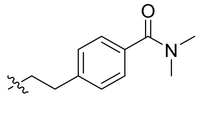	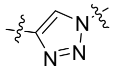	0.46	1.1	5.4	2.4
6	X21478	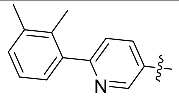	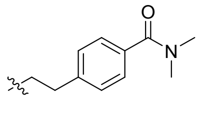	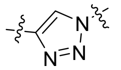	0.47	12	17	1.9
7	X21435	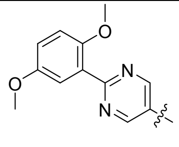	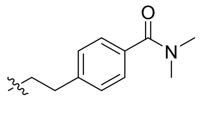	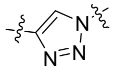	0.58	>125	n.d.	n.d.
8	X21499	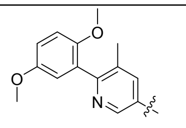	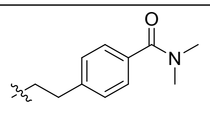	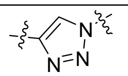	0.58	18	n.d.	1.6

9	X21502	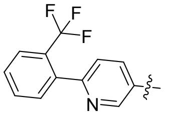	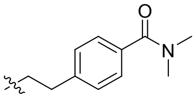	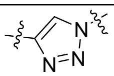	0.59	>125	n.d.	n.d.
10	X21558	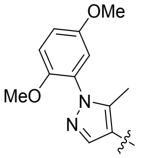	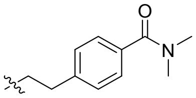	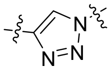	>20	n.d.	n.d.	n.d.
11	X21774	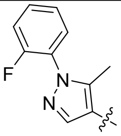	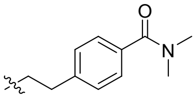	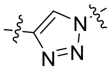	>10	>125	n.d.	n.d.
12	X21497	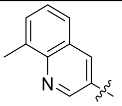	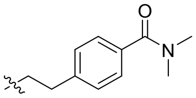	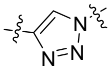	>10	>125	n.d.	n.d.
13	X21498	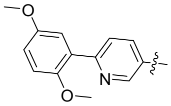	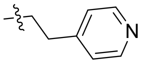	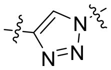	0.37	0.26	2.7	1.5
14	X21543	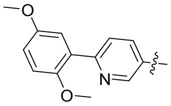	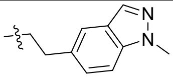	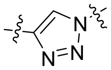	0.49	0.35	1.7	3.4
15	X21542	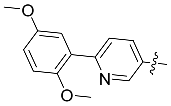	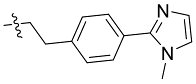	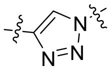	0.5	0.17	2.7	1.9
16	X21545	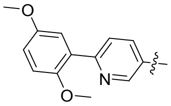	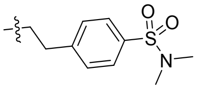	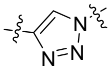	0.53	1.3	2.8	3.2
17	X21544	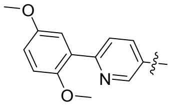	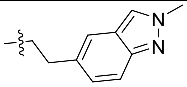	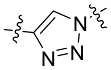	0.55	0.47	1.5	2.7
18	X23546	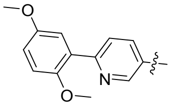	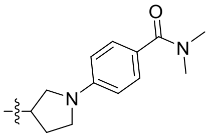	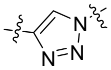	0.65	n.d.	n.d.	n.d.
19	X23479	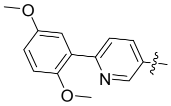	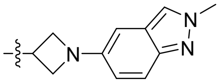	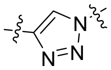	0.46	n.d.	n.d.	n.d.
20	X23488	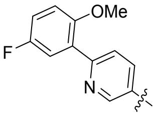	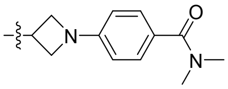	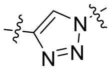	0.62	n.d.	n.d.	n.d.

21	X22309	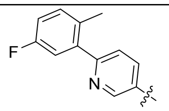	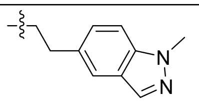	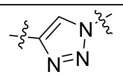	0.62	>125	n.d.	n.d.
22	X22311	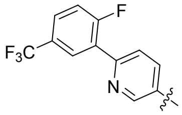	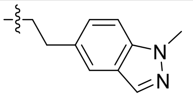	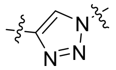	0.77	>125	n.d.	n.d.
23	X22346	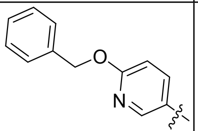	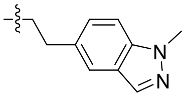	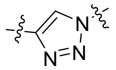	>10	>125	n.d.	n.d.
24	X22317	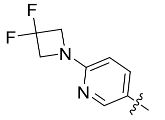	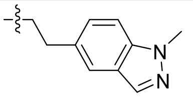	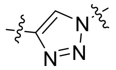	>10	>125	n.d.	n.d.
25	X22307	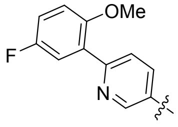	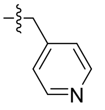	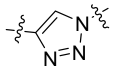	>10	>125	n.d.	n.d.
26	X21475	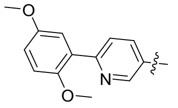	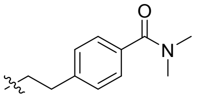	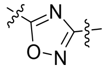	>20	n.d.	n.d.	n.d.
27	X23436	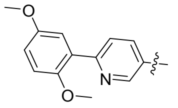	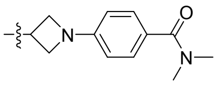	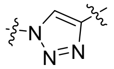	0.37	n.d.	n.d.	n.d.

Table S4. SAR of selected analogs of Series 2 (X13045).

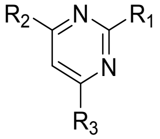

Entry	XID (Series)	R_1	R_2	R_3	IC50 (uM)	WT H37Rv MIC (uM)	Hypermorph MIC fold shift (+ATc/WT)	Hypomorph MIC fold shift (WT/-ATc)
1	X13045	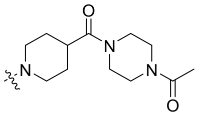	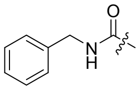	$-CH_3$	15.1	390	n.d.	1.5
2	X20377	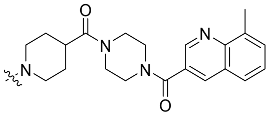	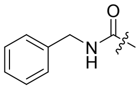	$-CH_3$	0.85	0.21	2.6	3.5
3	X21352	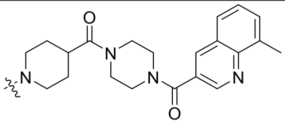	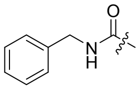	$-CH_3$	0.46	5.2	6.1	1.7
4	X21429	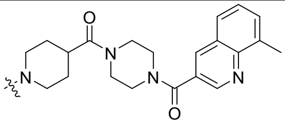	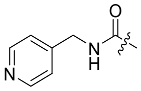	$-CH_3$	0.42	0.24	3.9	2.3
5	X21424	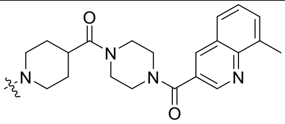	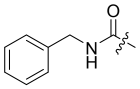	-H	0.39	2.6	5.8	3.0
6	X21408	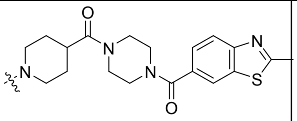	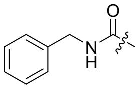	$-CH_3$	0.46	0.10	5.4	1.5
7	X20419	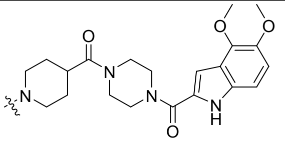	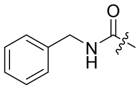	$-CH_3$	0.48	0.044	2.5	1.8
8	X21411	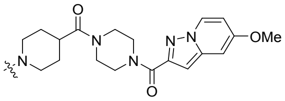	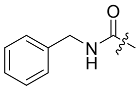	$-CH_3$	0.48	0.21	6.0	2.1

9	X22324	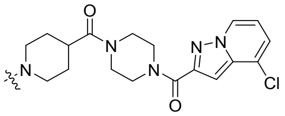	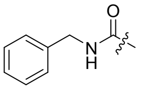	$-CH_3$	0.53	0.22	3.7	3.3
10	X22277	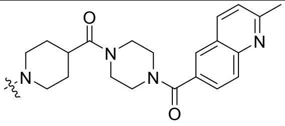	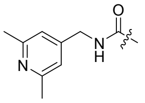	$-CH_3$	0.53	0.54	1.8	2.6
11	X22327	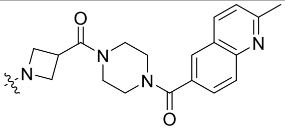	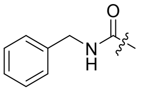	$-CH_3$	0.56	1.3	3.6	3.1
12	X21426	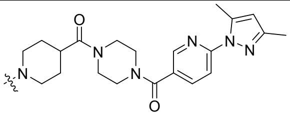	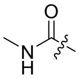	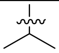	0.62	13	4.8	1.5
13	X22275	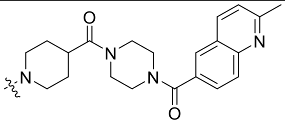	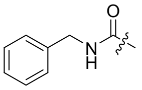	$-CH_3$	0.62	0.048	2.7	2.3
14	X22239	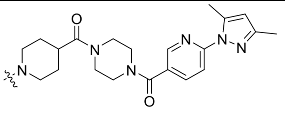	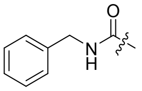	$-CH_3$	0.64	0.066	3.7	1.9
15	X22276	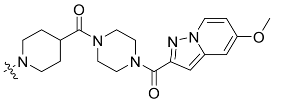	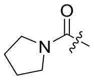	$-CH_3$	>10	>125	n.d.	n.d.
16	X22279	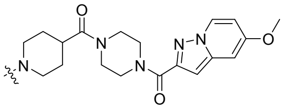	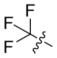	$-CH_3$	>10	>125	n.d.	n.d.
17	X22280	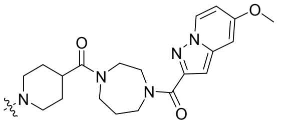	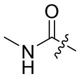	$-CH_3$	>10	>125	n.d.	n.d.

Table S5. SAR of selected analogs of Series 3 (X14146).

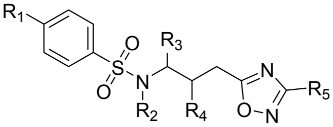

Entry	XCMPDID (Series)	R_1	R_2	R_3	R_4	R_5	IC50 (uM)	WT H37Rv MIC (uM)	Hypermorph MIC fold shift (+ATc/WT)	Hypomorph MIC fold shift (WT/-ATc)
	X14146	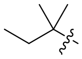	H	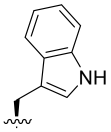	H	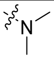	1.98	>125	n.d.	n.d.
2	X22258	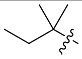	$-CH_3$	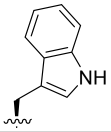	H	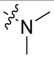	0.58	>125	n.d.	n.d.
3	X21403	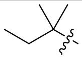	H	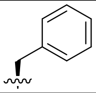	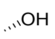	$-CH_3$	0.73	>125	n.d.	n.d.
4	X21413	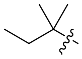	H	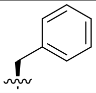	H	$-CH_3$	0.78	>125	n.d.	n.d.
5	X20425	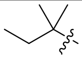	H	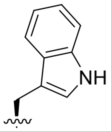	H	$-CH_3$	0.89	>250	n.d.	n.d.
6	X21364	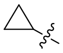	H	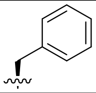	H	$-CH_3$	3.84	>125	n.d.	n.d.
7	X21522	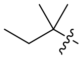	H	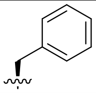	H	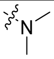	5.39	n.d.	n.d.	n.d.
8	X21365	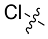	H	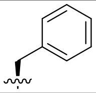	H	$-CH_3$	16.9	>125	n.d.	n.d.
9	X20462	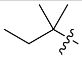	H	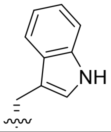	H	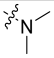	>20	>250	n.d.	n.d.
10	X20348	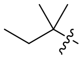	H	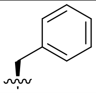	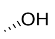	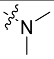	0.47	>250	n.d.	n.d.

Table S6 Data collection and refinement statistics of Pks13-TE bound to DEL inhibitors structures

	X20403 bound (8TQV)	X20404 bound (8TR4)	X20419 bound (8TQG)	X20348 bound (8TRY)
**Data collection**				
Space group	$P222_1$	$P22_12_1$	$P4_12_12$	$P2_12_12_1$
Cell dimensions				
a, *b*, *c* (Å)	64.56, 84.51, 124.7	57.94, 87.59, 109.38	66.63, 66.63, 129.95	54.86, 96.62, 130.97
α, β, γ (°)	90.00, 90.00, 90.00	90.00, 90.00, 90.00	90.00, 90.00, 90.00	90.00, 90.00, 90.00
Resolution (Å)	47.49 – 2.0 (2.05-2.0)	48.37-2.1 (2.16-2.1)	66.63-2.2 (2.27-2.2)	48.36-2.35 (2.43-2.35)
R_{merge}	0.062 (1.128)	0.088 (0.648)	0.113 (2.24)	0.174 (1.097)
$I/\sigma I$	10.4 (1.3)	10.1 (1.8)	8.9 (1.1)	5.3 (1.4)
Completeness (%)	98.9 (99.9)	92.1 (68)	99 (99.3)	85.8 (88.6)
Redundancy<br>CC1/2	5 (5.2)<br>0.998 (0.644)	3.3 (2.6)<br>0.997 (0.633)	6.2 (6.4)<br>0.998 (0.598)	3.4 (3.4)<br>0.986 (0.396)
**Refinement**				
Resolution (Å)	46.93 – 2.0	48.37 - 2.1	49.29 - 2.2	48.36 - 2.35
No. reflections	43951	28866	15293	23988
$R_{work/}$ R_{free}	0.224 / 0.246	0.185 / 0.238	0.242 / 0.313	0.196 / 0.256
No. atoms:				
Protein	4218	4313	2177	4356
Ligand(s) of interest	80	68	46	136
Water	95	272	13	82
B-factors (median):				
Protein	61	32	59	45
Ligand(s) of interest	60	24	78	45
R.m.s deviations:				
Bond lengths (Å)	0.005	0.009	0.0081	0.018
Bond angles (º)	0.886	1.092	1.0079	1.592

Table S7. Resistant to X20404 Mtb isolates sequencing results.

Mutant no.	Pks13 Missense Mutation	Additional SNP(s) Detected	Gene Name	Gene Description	Mutation Type	Amino Acid Change
1	R1563H	Rv1929c	NA	Conserved hypothetical protein	Missense	T172S
1	R1563H	Rv2931	ppsA	Phenolpthiocerol synthesis type-I polyketide synthase PpsA	Missense	W1294*
1	R1563H	Rv3645	NA	Probable conserved transmembrane protein	Frameshift Ins	NA
1	R1563H	Rv3903c	NA	Hypothetical alanine and proline rich protein	In-frame Ins	NA
2	R1563H	Rv0647c	NA	Conserved protein	Missense	G6R
2	R1563H	Rv0647c	NA	Conserved protein	Silent	NA
2	R1563H	Rv2565	NA	Conserved protein	Missense	G567R
2	R1563H	Rv2931	ppsA	Phenolpthiocerol synthesis type-I polyketide synthase PpsA	Missense	W1294*
2	R1563H	Rv2933	ppsC	Phenolpthiocerol synthesis type-I polyketide synthase PpsC	Missense	E473G
2	R1563H	Rv3169	NA	Conserved protein	Missense	A190G
3	V1687F	Rv0007	NA	Possible conserved membrane protein	Missense	A180G
3	V1687F	Rv0007	NA	Possible conserved membrane protein	Missense	R181G
3	V1687F	Rv2226	NA	Conserved protein	Frameshift Ins	NA
3	V1687F	Rv2339	mmpL9	Probable conserved transmembrane transport protein MmpL9	Missense	T871A
3	V1687F	Rv2931	ppsA	Phenolpthiocerol synthesis type-I polyketide synthase PpsA	Frameshift Ins	NA
3	V1687F	Rv3081	NA	Conserved hypothetical protein	Frameshift Ins	NA
3	V1687F	Rv3081	NA	Conserved hypothetical protein	Missense	P217R
3	V1687F	Rv3736	NA	Transcriptional regulatory protein (probably AraC/XylS-family)	Missense	T87P
3	V1687F	Rv3903c	NA	Hypothetical alanine and proline rich protein	Frameshift Ins	NA
4	A1561V	Rv2933	ppsC	Phenolpthiocerol synthesis type-I polyketide synthase PpsC	Frameshift Ins	NA
4	A1561V	Rv3081	NA	Conserved hypothetical protein	In-frame Ins	NA
4	A1561V	Rv3081	NA	Conserved hypothetical protein	Missense	P217R
4	A1561V	Rv3169	NA	Conserved protein	Missense	A190G
4	A1561V	Rv3169	NA	Conserved protein	In-frame Ins	NA
5	V1687F	Rv2934	ppsD	Phenolpthiocerol synthesis type-I polyketide synthase PpsD	Missense	A39E
5	V1687F	Rv3081	NA	Conserved hypothetical protein	Frameshift Ins	NA
5	V1687F	Rv3081	NA	Conserved hypothetical protein	Missense	P217R

5	V1687F	Rv3903c	NA	Hypothetical alanine and proline rich protein	Frameshift Ins	NA
6	V1537A	Rv0425c	ctpH	Possible metal cation transporting P-type ATPase CtpH	Missense	V1052M
6	V1537A	Rv0647c	NA	Conserved protein	Missense	G6R
6	V1537A	Rv0647c	NA	Conserved protein	Silent	NA
6	V1537A	Rv2933	ppsC	Phenolpthiocerol synthesis type-I polyketide synthase PpsC	Frameshift Ins	NA
6	V1537A	Rv3081	NA	Conserved hypothetical protein	In-frame Ins	NA
6	V1537A	Rv3081	NA	Conserved hypothetical protein	Missense	P217R
7	V1687F	Rv0425c	ctpH	Possible metal cation transporting P-type ATPase CtpH	Missense	V1052M
7	V1687F	Rv1263	amiB2	Probable amidase AmiB2 (aminohydrolase)	Missense	A346G
7	V1687F	Rv1263	amiB2	Probable amidase AmiB2 (aminohydrolase)	In-frame Ins	NA
7	V1687F	Rv2187	fadD15	Long-chain-fatty-acid-CoA ligase FadD15 (fatty-acid-CoA synthetase) (fatty-acid-CoA synthase)	Missense	A354P
7	V1687F	Rv2187	fadD15	Long-chain-fatty-acid-CoA ligase FadD15 (fatty-acid-CoA synthetase) (fatty-acid-CoA synthase)	Missense	G352A
7	V1687F	Rv2187	fadD15	Long-chain-fatty-acid-CoA ligase FadD15 (fatty-acid-CoA synthetase) (fatty-acid-CoA synthase)	Missense	G352R
7	V1687F	Rv2187	fadD15	Long-chain-fatty-acid-CoA ligase FadD15 (fatty-acid-CoA synthetase) (fatty-acid-CoA synthase)	Missense	G353A
7	V1687F	Rv2187	fadD15	Long-chain-fatty-acid-CoA ligase FadD15 (fatty-acid-CoA synthetase) (fatty-acid-CoA synthase)	Missense	G353R
7	V1687F	Rv2187	fadD15	Long-chain-fatty-acid-CoA ligase FadD15 (fatty-acid-CoA synthetase) (fatty-acid-CoA synthase)	Frameshift Del	NA
7	V1687F	Rv2187	fadD15	Long-chain-fatty-acid-CoA ligase FadD15 (fatty-acid-CoA synthetase) (fatty-acid-CoA synthase)	Missense	S351P
7	V1687F	Rv2187	fadD15	Long-chain-fatty-acid-CoA ligase FadD15 (fatty-acid-CoA	Missense	V350A

				synthetase) (fatty-acid-CoA synthase)		
7	V1687F	Rv2933	ppsC	Phenolpthiocerol synthesis type-I polyketide synthase PpsC	Frameshift Ins	NA
7	V1687F	Rv3081	NA	Conserved hypothetical protein	In-frame Ins	NA
7	V1687F	Rv3081	NA	Conserved hypothetical protein	Missense	P217R
7	V1687F	Rv3169	NA	Conserved protein	In-frame Ins	NA
7	V1687F	Rv3736	NA	Transcriptional regulatory protein (probably AraC/XylS-family)	Missense	A89P
7	V1687F	Rv3903c	NA	Hypothetical alanine and proline rich protein	Frameshift Ins	NA
8	Y1686C	Rv0592	mce2D	Mce-family protein Mce2D	Missense	F268L
8	Y1686C	Rv2769c	PE27	PE family protein PE27	Silent	NA
8	Y1686C	Rv2931	ppsA	Phenolpthiocerol synthesis type-I polyketide synthase PpsA	Frameshift Ins	NA
8	Y1686C	Rv2933	ppsC	Phenolpthiocerol synthesis type-I polyketide synthase PpsC	Missense	E473G
8	Y1686C	Rv3081	NA	Conserved hypothetical protein	Frameshift Ins	NA
8	Y1686C	Rv3081	NA	Conserved hypothetical protein	Missense	P217R
8	Y1686C	Rv3736	NA	Transcriptional regulatory protein (probably AraC/XylS-family)	Missense	A89P
9	A1564D	Rv2931	ppsA	Phenolpthiocerol synthesis type-I polyketide synthase PpsA	Frameshift Ins	NA
9	A1564D	Rv3081	NA	Conserved hypothetical protein	In-frame Ins	NA
9	A1564D	Rv3081	NA	Conserved hypothetical protein	Missense	P217R
10	Y1674C	Rv2930	fadD26	Fatty-acid-AMP ligase FadD26 (fatty-acid-AMP synthetase) (fatty-acid-AMP synthase)	Frameshift Del	NA
10	Y1674C	Rv2933	ppsC	Phenolpthiocerol synthesis type-I polyketide synthase PpsC	Missense	E473G
10	Y1674C	Rv3081	NA	Conserved hypothetical protein	Missense	P217R
10	Y1674C	Rv3645	NA	Probable conserved transmembrane protein	In-frame Ins	NA
10	Y1674C	Rv3903c	NA	Hypothetical alanine and proline rich protein	Frameshift Ins	NA
11	N1640K	Rv0647c	NA	Conserved protein	Missense	G6R
11	N1640K	Rv2931	ppsA	Phenolpthiocerol synthesis type-I polyketide synthase PpsA	Frameshift Ins	NA
11	N1640K	Rv3081	NA	Conserved hypothetical protein	Missense	P217R
11	N1640K	Rv3533c	PPE62	PPE family protein PPE62	Missense	G251A
11	N1640K	Rv3533c	PPE62	PPE family protein PPE62	Missense	L258I
11	N1640K	Rv3533c	PPE62	PPE family protein PPE62	Missense	N255I
11	N1640K	Rv3533c	PPE62	PPE family protein PPE62	Silent	NA
11	N1640K	Rv3533c	PPE62	PPE family protein PPE62	Silent	NA
11	N1640K	Rv3533c	PPE62	PPE family protein PPE62	Silent	NA

11	N1640K	Rv3533c	PPE62	PPE family protein PPE62	Silent	NA
11	N1640K	Rv3533c	PPE62	PPE family protein PPE62	Frameshift Ins	NA
11	N1640K	Rv3533c	PPE62	PPE family protein PPE62	Frameshift Ins	NA
11	N1640K	Rv3533c	PPE62	PPE family protein PPE62	Missense	V253L
