# Supplementary material for: Inhibitors of the Thioesterase Activity of Mycobacterium tuberculosis Pks13 Discovered Using DNA-Encoded Chemical Library Screening
Source: ACS Infect Dis. 2024 Apr 5;10(5):1561–75. doi: 10.1021/acsinfecdis.3c00592 (PMC11091879; doi:10.1021/acsinfecdis.3c00592)
Supplement: Supplementary file 2 — id3c00592_si_002.pdf [file id3c00592_si_002.pdf]

Supporting Information - Chemistry

Inhibitors of the Thioesterase Activity of Mycobacterium tuberculosis Pks13 Discovered using DNA-Encoded Chemical Library Screening

Inna V. Krieger[1], Subbarao Yalamanchili[2], Paige Dickson[2], Curtis A. Engelhart[7], Matthew D Zimmerman[8], Jeremy Wood[1], Ethan Clary[1], Jasmine Nguyen[1], Natalie Thornton[7], Paolo A. Centrella[2], Betty Chan[2,10], John W Cuozzo[2,3], Martin Gengenbacher[8], Marie-Aude Guié[2], John P Guilinger[2], Corey Bienstock[2], Hajnalka Hartl[2,9], Christopher D. Hupp[2,4], Rachael Jetson[2,5], Takashi Satoh[2,6], John T. S. Yeoman[2,11], Ying Zhang[2], Veronique Dartois[8], Dirk Schnappinger[7], Anthony D. Keefe[2]*, James C. Sacchettini[1]*
* corresponding authors
Email: Anthony D. Keefe akeefe@x-chemrx.com, James C. Sacchettini sacchett@tamu.edu

Affiliations:
1 Department of Biochemistry & Biophysics, Texas A&M University, College Station, Texas, 77843, USA
2 X-Chem Inc., 100 Beaver Street, Waltham, Massachusetts, 02453, USA
3 Relay Therapeutics, 399 Binney Street, Cambridge, Massachusetts, 02141, USA.
4 Ipsen Bioscience Inc., 1 Main Street, Cambridge, Massachusetts, 02142, USA.
5 Valo Health, 75 Hayden Avenue, Lexington, Massachusetts, 02141, USA.
6 EXO Therapeutics, 150 Cambridgepark Drive, suite 300, Cambridge, Massachusetts, 02140, USA.
7 Department of Microbiology and Immunology, Weill Cornell Medicine, New York, New York, 10021, USA.
8 Center for Discovery and Innovation, Hackensack Meridian Health, Nutley, New Jersey, 07110, USA

Hackensack Meridian School of Medicine, Hackensack Meridian Health, Nutley, New Jersey, 07110, USA

9 Orogen Therapeutics, 12 Gill Street, Woburn, Massachusetts, 01801, USA.
10 Auron Therapeutics, 55 Chapel Street, Newton, Massachusetts, 02458, USA.
11 Recludix Pharmaceuticals, 222 Third Street, Cambridge, Massachusetts, 02142, USA.

Contents

Synthesis of X20404 .... S3

Synthesis of X13045 .... S5

Synthesis of X21434 .... S7

Synthesis of X21464 .... S10

Synthesis of X22317 .... S11

Synthesis of X21426 .... S13

Synthesis of X23488 .... S15

Synthesis of X21352 .... S18

Synthesis of X21411 .... S20

Synthesis of X21408 .... S21

Synthesis of X22307 .... S22

Synthesis of X22346 .... S23

Synthesis of X23546 .... S24

Synthesis of X23479 .... S27

Synthesis of X22311 .... S29

Synthesis of X21424 .... S31

Synthesis of X22309 .... S32

Synthesis of X21429 .... S34

Synthesis of X23436 .... S36

Synthesis of X20377 and X20419 .... S38

Synthesis of X22324 .... S40

Synthesis of X22239 .... S41

Synthesis of X22275 .... S43

Synthesis of X22276 .... S44

Synthesis of X22277 .... S46

Synthesis of X22279 .... S48

Synthesis of X22327 .... S50

Synthesis of X22280 .... S51

Synthesis of X21774 .... S53

Synthesis of X21475 .... S55
Synthesis of X21498 .... S58
Synthesis of X21542 .... S60
Synthesis of X21543 .... S61
Synthesis of X21544 .... S63
Synthesis of X21499 .... S64
Synthesis of X21545 .... S67
Synthesis of X20403 .... S70
Synthesis of X21546 .... S73
Synthesis of X21478 .... S74
Synthesis of X21435 .... S75
Synthesis of X21502 .... S77
Synthesis of X21558 .... S78
Synthesis of X21497 .... S80

Synthesis of X20404

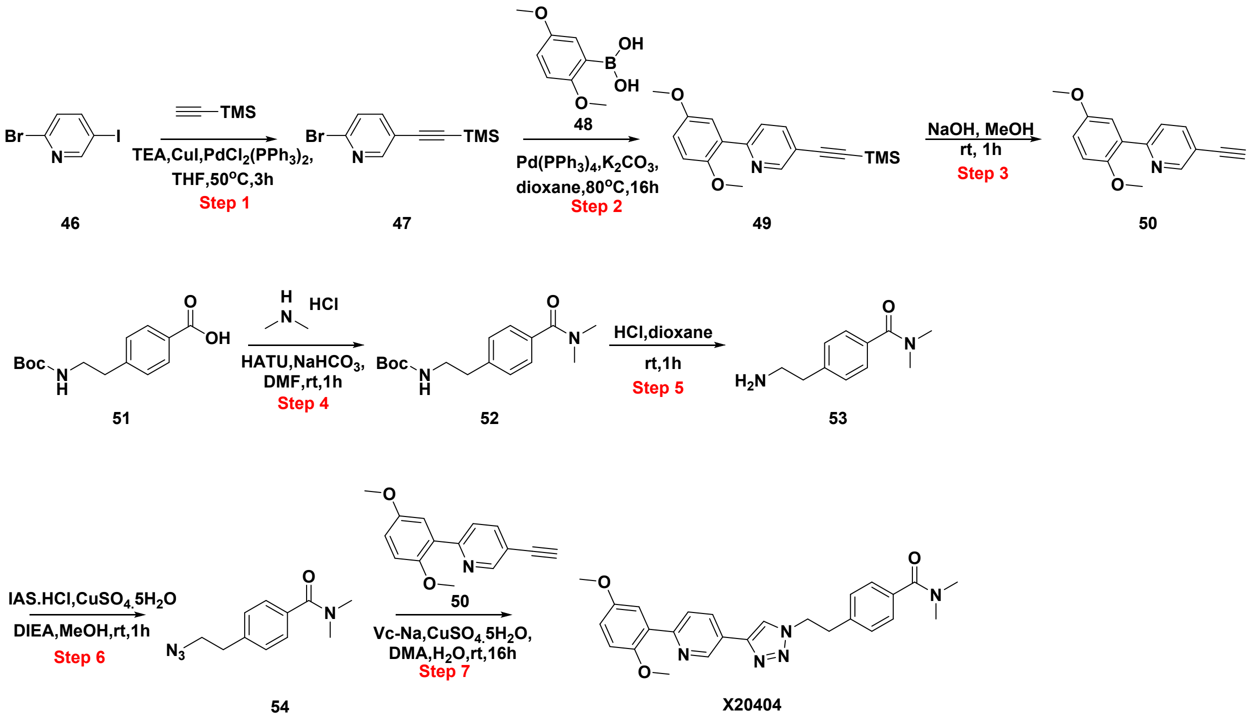

Synthesis of 2-bromo-5-((trimethylsilyl)ethynyl)pyridine (47):

To a stirred solution of 2-bromo-5-iodopyridine (**46**, 1.00 g, 3.52 mmol, 1.00 equiv) and trimethylsilylacetylene (519 mg, 5.28 mmol, 1.50 equiv) in THF (20 mL) were added TEA (713 mg, 7.04 mmol, 2.00 equiv), CuI (335 mg, 1.76 mmol, 0.50 equiv) and $Pd(PPh_3)_2Cl_2$ (494 mg, 0.70 mmol, 0.20 equiv). The resulting mixture was stirred at 50 ^oC for 3 h under nitrogen atmosphere. The resulting mixture was purified using flash chromatography with the following conditions (Mobile Phase A: Water, Mobile Phase B: ACN; Flow rate: 60 mL/min; Gradient: 0% B to 100% B in 25 min; 254/220 nm) to afford 2-bromo-5-[2-(trimethylsilyl)ethynyl]pyridine (**47**, 660 mg, 73.7%) as a yellow solid. $[M+H]^+$ = 254/256

Synthesis of 2-(2,5-dimethoxyphenyl)-5-((trimethylsilyl)ethynyl)pyridine (49) :

To a stirred solution of 2-bromo-5-[2-(trimethylsilyl)ethynyl]pyridine (**47**, 640 mg, 2.52 mmol, 1.00 equiv) and 2,5-dimethoxyphenylboronic acid (**48**, 550 mg, 3.02 mmol, 1.20

equiv) in dioxane (10 mL) were added $Pd(PPh_3)_4$ (145 mg, 0.13 mmol, 0.05 equiv) and K_2CO_3(696 mg, 5.04 mmol, 2.00 equiv). The resulting mixture was stirred at 80 ^oC overnight under nitrogen atmosphere. The resulting mixture was purified using flash chromatography with the following conditions (Mobile Phase A: Water, Mobile Phase B: ACN; Flow rate: 60 mL/min; Gradient: 0% B to 100% B in 25 min; 254/220 nm) to afford 2-(2,5-dimethoxyphenyl)-5-[2-(trimethylsilyl)ethynyl]pyridine (**49**, 530 mg, 67.6%) as a yellow solid. $[M+H]^+$ = 312

Synthesis of 2-(2,5-dimethoxyphenyl)-5-ethynylpyridine (50)

A solution of 2-(2,5-dimethoxyphenyl)-5-[2-(trimethylsilyl)ethynyl]pyridine (**49**, 480 mg, 1.54 mmol, 1.00 equiv) and NaOH (185 mg, 4.62 mmol, 3.00 equiv) in MeOH (5 mL) and H_2O (3 ML) was stirred at room temperature for 3 h. The resulting mixture was purified using flash chromatography with the following conditions (Mobile Phase A: Water, Mobile Phase B: ACN; Flow rate: 60 mL/min; Gradient: 0% B to 100% B in 25 min; 254/220 nm) to afford 2-(2,5-dimethoxyphenyl)-5-ethynylpyridine (**50**, 230 mg, 62.37%) as a yellow solid. $[M+H]^+$ = 240

Synthesis of tert-butyl (4-(dimethylcarbamoyl)phenethyl)carbamate (52)

To a stirred solution of P-formylphenoxyacetic acid (**51**, 500 mg, 2.78 mmol, 1.00 equiv) and dimethylamine hydrochloride (453 mg, 5.55 mmol, 2.00 equiv) in DMF (8 mL) were added HATU (1.27 g, 3.33 mmol, 1.20 equiv) and $NaHCO_3$ (699 mg, 8.33 mmol, 3.00 equiv). The resulting mixture was stirred at room temperature for 1 h. The resulting mixture was purified using flash chromatography with the following conditions (Mobile Phase A: Water, Mobile Phase B: ACN; Flow rate: 60 mL/min; Gradient: 0% B to 100% B in 25 min; 254/220 nm) to afford 2-(4-formylphenoxy)-*N,N*-dimethylacetamide (**52**, 320 mg, 55.6%) as a white solid. $[M+H]^+$ = 293

Synthesis of 4-(2-aminoethyl)-N,N-dimethylbenzamide (53)

A solution of tert-butyl N-[2-[4-(dimethylcarbamoyl)phenyl]ethyl]carbamate (**7**, 300 mg, 1.03 mmol, 1.00 equiv) and HCl (4M in 1,4-dioxane, 5 mL) was stirred at room temperature for 1 h. The resulting mixture was concentrated under vacuum. This resulted in 4-(2-aminoethyl)-N,N-

dimethylbenzamide (**53**, 200 mg, crude) as a white solid. $[M+H]^+$ = 193

Synthesis of 4-(2-azidoethyl)-N,N-dimethylbenzamide (54)

To a stirred solution of 4-(2-aminoethyl)-N,N-dimethylbenzamide (**53**, 190 mg, 0.99 mmol, 1.00 equiv) and ISA.HCl (413 mg, 1.98 mmol, 2.00 equiv) in MeOH (4 mL) were added $CuSO_4.5H_2O$ (123 mg, 0.49 mmol, 0.50 equiv) and DIEA (255 mg, 1.99 mmol, 2.00 equiv). The resulting mixture was stirred at room temperature for 1 h. The resulting mixture was diluted with water. The resulting mixture was extracted with EtOAc. The combined organic layers were washed with brine, dried over anhydrous Na_2SO_4. After filtration, the filtrate was concentrated under reduced pressure. This resulted in 4-(2-azidoethyl)-*N,N*-dimethylbenzamide (**54**, 200 mg, crude) as a yellowsolid. $[M+H]^+$ = 219

Synthesis of 4-(2-(4-(6-(2,5-dimethoxyphenyl)pyridin-3-yl)-1H-1,2,3-triazol-1-yl)ethyl)-N,N-dimethylbenzamide (X20404)

To a stirred solution of 4-(2-azidoethyl)-*N,N*-dimethylbenzamide (**54**, 190 mg, 0.87 mmol, 1.00 equiv) and 2-(2,5-dimethoxyphenyl)-5-ethynylpyridine (**50**, 208 mg, 0.87 mmol, 1.00 equiv) in DMA (2 mL) and H_2O (2 mL) were added $CuSO_4.5H_2O$ (217 mg, 0.87 mmol, 1.00 equiv) and Vc-Na (172 mg, 0.87 mmol, 1.00 equiv). The resulting mixture was stirred at room temperature overnight. The resulting mixture was purified by Prep-HPLC with the following conditions (Column: XBridge Prep OBD C18 Column, 30×150mm 5um; Mobile Phase A: Water (10mm NH_4HCO_3), Mobile Phase B: ACN; Flow rate:60 mL/min; Gradient:30% B to 45% B in 8 min, 254/220 nm; RT1: 7.17 min). This resulted in 4-(2-[4-[6-(2,5-dimethoxyphenyl)pyridin-3-yl]-1,2,3-triazol-1-yl]ethyl)-*N,N*-dimethylbenzamide (**X20404**, 24.1 mg, 6.05%) as a white solid. $[M+H]^+$ = 458. ^{1}H NMR (DMSO-d_6, 400 MHz) δ 2.87 (3H, s), 2.96 (3H, s), 3.28 (2H, t), 3.77 (3H, s), 3.81 (3H, s), 4.73 (2H, t), 7.00 (1H, dd), 7.11 (1H, d), 7.25 – 7.36 (4H, m), 7.40 (1H, d), 7.98 (1H, d), 8.19 (1H, dd), 8.66 (1H, s), 9.08 (1H, d).

Synthesis of X13045

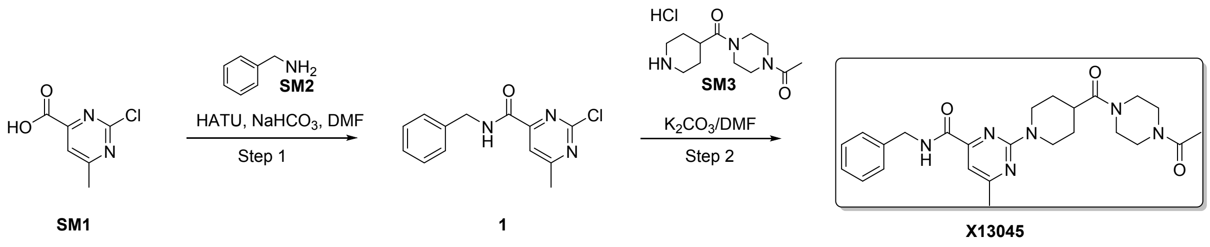

Synthesis of N-benzyl-2-chloro-6-methylpyrimidine-4-carboxamide (1)

A solution of **SM1** (500 mg, 2.90 mmol), **SM2** (311 mg, 2.90 mmol), HATU (2200 mg, 5.80 mmol) and $NaHCO_3$ (487 mg, 5.80 mmol) in DMF (5 mL) was stirred at rt for overnight. The reaction mixture was diluted with H_2O (20 mL), and extracted with Ethyl Acetate (20 mLx3), the combined organic layer was washed with brine (10 mLx3), then dried over with anhydrous Na_2SO_4. After filtration, the solution was concentrated under vacuum to give the crude product, the crude product was purified using CombiFlash to give intermediate **1** (450 mg, 59 %). LC-MS $(M+H)^+$ = 262.

Synthesis of 2-(4-(4-acetylpiperazine-1-carbonyl)piperidin-1-yl)-N-benzyl-6-methylpyrimidine-4-carboxamide (X13045)

To a solution of **1** (200 mg, 0.76 mmol) in DMF (5 ml) were added **SM3** (181 mg, 0.76 mmol) and K_2CO_3 (314 mg, 2.28 mmol). The mixture was stirred at rt for overnight. After the reaction completed, H_2O (20 mL) was added and then extracted with Ethyl Acetate (20 mLx3). The combined organic layer was washed with brine (5 mLx3), then dried over with anhydrous Na_2SO_4. After filtration, the solution was concentrated under vacuum and purified by prep-HPLC to give ***X13045*** (37 mg, 10%) as a yellow solid. LC-MS $(M+H)^+$ = 465. ^1H NMR (400 MHz, MeOD) δ 7.37 – 7.30 (m, 4H), 7.29 – 7.22 (m, 1H), 7.15 (s, 1H), 4.92 (d, J = 13.5 Hz, 2H), 4.60 (s, 2H), 3.78 – 3.50 (m, 8H), 3.08 (m, 3H), 2.46 (s, 3H), 2.15 (s, 3H), 1.91 – 1.63 (m, 4H).

Synthesis of X21434

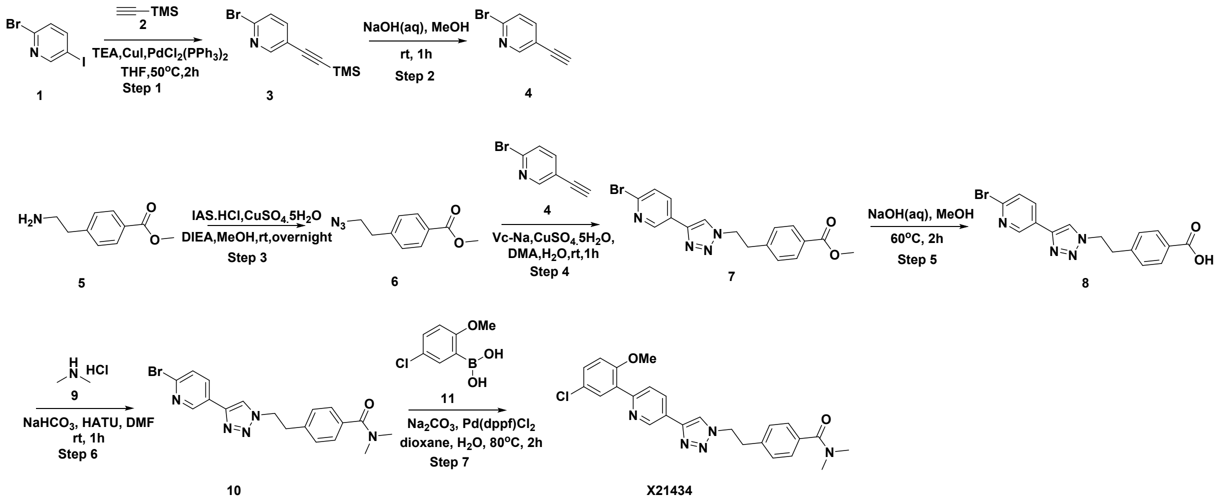

Synthesis of 2-bromo-5-[2-(trimethylsilyl)ethynyl]pyridine (3):

To a stirred mixture of 2-bromo-5-iodopyridine (**1**, 15.00 g, 52.8 mmol, 1.00 equiv) and TEA (10.69 g, 105.7 mmol, 2.00 equiv) in THF (200 mL) were added trimethylsilylacetylene (**2**, 6.23 g, 63.4 mmol, 1.20 equiv) and CuI (5.03 g, 26.4 mmol, 0.50 equiv) and $Pd(PPh_3)_2Cl_2$ (3.71 g, 5.28 mmol, 0.10 equiv) in portions at room temperature under N_2. The resulting mixture was stirred at 50°C for 2 hours under N_2. The mixture was extracted with EtOAc, the combined organic layers were washed with brine, dried over anhydrous Na_2SO_4. After filtration, the filtrate was concentrated under reduced pressure. The residue was purified by silica gel column chromatography, eluted with PE / EA (40:1) to afford 2-bromo-5-[2-(trimethylsilyl)ethynyl]pyridine (**3**, 9.00 g, 67.0%) as a light yellow solid. $[M+H]^+$ = 254/256

Synthesis of 2-bromo-5-ethynylpyridine (4):

To a stirred solution of 2-bromo-5-[2-(trimethylsilyl)ethynyl]pyridine (**3**, 5.00 g, 19.7 mmol, 1.00 equiv) in MeOH (50 mL) was added NaOH (2 M, 20 mL) at room temperature. The resulting mixture was stirred at room temperature for 1 hour. The mixture was diluted with water. The resulting mixture was extracted with EtOAc. The combined organic layers were washed with brine, dried over anhydrous Na_2SO_4. After filtration, the filtrate was concentrated under reduced pressure. This resulted in 2-bromo-5-ethynylpyridine (**4**, 3.50 g, crude) as a light yellow solid. $[M+H]^+$ = 182/184

Synthesis of methyl 4-(2-azidoethyl)benzoate (6):

To a stirred mixture of methyl 4-(2-aminoethyl)benzoate (**5**, 6.00 g, 33.48 mmol, 1.00 equiv) and DIEA (17.31 g, 133.9 mmol, 4.00 equiv) in MeOH (100 mL) were added $CuSO_4 \cdot 5H_2O$ (4.18 g, 16.7 mmol, 0.50 equiv) and ISA.HCl (13.99 g, 67.0 mmol, 2.00 equiv) in portions at room temperature. The resulting mixture was stirred at room temperature overnight. The mixture was diluted with water. The resulting mixture was extracted with EtOAc. The combined organic layers were washed with brine, dried over anhydrous Na_2SO_4. After filtration, the filtrate was concentrated under reduced pressure. This resulted in methyl 4-(2-azidoethyl)benzoate (**6**, 6.00 g, crude) as a light yellow oil. $[M+H]^+$ = 206

Synthesis of methyl 4-{2-[4-(6-bromopyridin-3-yl)-1,2,3-triazol-1-yl]ethyl}benzoate (7):

To a stirred mixture of methyl 4-(2-azidoethyl)benzoate (**6**, 3.00 g, 14.6 mmol, 1.00 equiv) and 2-bromo-5-ethynylpyridine (**4**, 2.66 g, 14.6 mmol, 1.00 equiv) in DMA (30 mL) and H_2O (30 mL) were added $CuSO_4 \cdot 5H_2O$ (10.95 g, 43.9 mmol, 3.00 equiv) and VcNa (8.69 g, 43.9 mmol, 3.00 equiv) in portions at room temperature. The resulting mixture was stirred at room temperature for 1 hour. The mixture was diluted with water. The resulting mixture was extracted with EtOAc. The combined organic layers were washed with brine, dried over anhydrous Na_2SO_4. After filtration, the filtrate was concentrated under reduced pressure. This resulted in methyl 4-{2-[4-(6-bromopyridin-3-yl)-1,2,3-triazol-1-yl]ethyl}benzoate (**7**, 3.00 g, crude) as a light yellow solid. $[M+H]^+$ = 387/389

Synthesis of 4-{2-[4-(6-bromopyridin-3-yl)-1,2,3-triazol-1-yl]ethyl}benzoic acid (8):

To a stirred solution of methyl 4-{2-[4-(6-bromopyridin-3-yl)-1,2,3-triazol-1-yl]ethyl}benzoate (**7**, 3.00 g, 7.75 mmol, 1.00 equiv) in MeOH (30 mL) was added NaOH (2 M, 10 mL) dropwise at room temperature. The resulting mixture was stirred at 60°C for 2 hours. The mixture was acidified to pH 5 with HCl (1 M). The mixture was extracted with EtOAc. The combined organic layers were washed with brine, dried over anhydrous Na_2SO_4. After filtration, the filtrate was concentrated under reduced pressure. This resulted in 4-{2-[4-(6-bromopyridin-3-yl)-1,2,3-triazol-1-yl]ethyl}benzoic acid (**8**, 2.50 g, crude) as a light yellow solid. $[M+H]^+$ = 373/375

Synthesis of 4-{2-[4-(6-bromopyridin-3-yl)-1,2,3-triazol-1-yl]ethyl}-N,N-

dimethylbenzamide (10):

To a stirred mixture of 4-{2-[4-(6-bromopyridin-3-yl)-1,2,3-triazol-1-yl]ethyl}benzoic acid (**8**, 2.50 g, 6.70 mmol, 1.00 equiv) and dimethylamine hydrochloride (**9**, 1.09 g, 13.4 mmol, 2.00 equiv) in DMF (30 mL) were added $NaHCO_3$ (2.81 g, 33.5 mmol, 5.00 equiv) and HATU (3.06 g, 8.04 mmol, 1.20 equiv) in portions at room temperature. The resulting mixture was stirred at room temperature for 1 hour. The mixture was purified using flash chromatography with the following conditions (Mobile Phase A: Water, Mobile Phase B: ACN; Flow rate: 60 mL/min; Gradient: 0% B to 65% B in 50 min; 254/220 nm). This resulted in 4-{2-[4-(6-bromopyridin-3-yl)-1,2,3-triazol-1-yl]ethyl}-*N,N*-dimethylbenzamide (**10**, 750 mg, 28.0%) as a light yellow solid. $[M+H]^+$ = 400/402

Synthesis of 4-(2-{4-[6-(5-chloro-2-methoxyphenyl)pyridin-3-yl]-1,2,3-triazol-1-yl}ethyl)-N,N-dimethylbenzamide (X21434):

To a stirred mixture of 4-{2-[4-(6-bromopyridin-3-yl)-1,2,3-triazol-1-yl]ethyl}-*N,N*-dimethylbenzamide (**10**, 150 mg, 0.38 mmol, 1.00 equiv) and 5-chloro-2-methoxyphenylboronic acid (**11**, 210 mg, 1.12 mmol, 3.00 equiv) in dioxane (2 mL) were added Na_2CO_3 (119 mg, 1.12 mmol, 3.00 equiv), H_2O (0.5 mL) and Pd(dppf)Cl_2 (27 mg, 0.04 mmol, 0.10 equiv) at room temperature under N_2. The resulting mixture was stirred at 80°C for 1 hour under N_2. The mixture was concentrated under reduced pressure. The residue was purified using flash chromatography with the following conditions (Mobile Phase A: Water, Mobile Phase B: ACN; Flow rate: 60 mL/min; Gradient: 0% B to 65% B in 50 min; 254/220 nm). This resulted in 4-(2-{4-[6-(5-chloro-2-methoxyphenyl)pyridin-3-yl]-1,2,3-triazol-1-yl}ethyl)-*N,N*-dimethylbenzamide (**X21434**, 43 mg, 25.0%) as a white solid. $[M+H]^+$ = 462. ^{1}H NMR (DMSO-d_6, 400 MHz) δ 2.91 (6H, d), 3.28 (2H, t), 3.88 (3H, s), 4.73 (2H, t), 7.21 (1H, d), 7.27 – 7.34 (4H, m), 7.47 (1H, dd), 7.84 (1H, d), 8.00 (1H, dd), 8.22 (1H, dd), 8.69 (1H, s), 9.10 (1H, d).

Synthesis of X21464

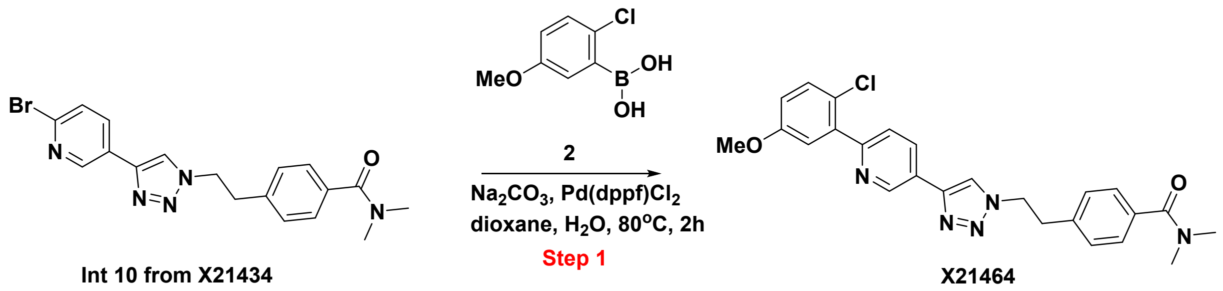

Synthesis of 4-(2-{4-[6-(2-chloro-5-methoxyphenyl)pyridin-3-yl]-1,2,3-triazol-1-yl}ethyl)-N,N-dimethylbenzamide (X21464):

To a stirred mixture of 4-{2-[4-(6-bromopyridin-3-yl)-1,2,3-triazol-1-yl]ethyl}-*N,N*-dimethylbenzamide (int **10 from X21434**, 100 mg, 0.25 mmol, 1.00 equiv) and 2-chloro-5-methoxyphenylboronic acid (**2**, 46.6 mg, 0.75 mmol, 3.00 equiv) in dioxane (1.00 mL) were added Na_2CO_3 (79 mg, 0.75 mmol, 3.00 equiv), H_2O (0.3 mL) and Pd(dppf)Cl_2 (18 mg, 0.02 mmol, 0.10 equiv) at room temperature under N_2. The resulting mixture was stirred at 80°C for 2 hours under N_2. The mixture was concentrated under reduced pressure. The residue was purified using Prep-HPLC with the following conditions (Column: XBridge Prep OBD C18 Column, 30*150 mm, 5 µm; Mobile Phase A: Water (10 mmol/L NH_4HCO_3), Mobile Phase B: ACN; Flow rate: 60 mL/min; Gradient: 35% B to 45% B in 7 min, Wave Length: 220 nm; RT: 7.82 min) to afford 4-(2-{4-[6-(2-chloro-5-methoxyphenyl)pyridin-3-yl]-1,2,3-triazol-1-yl}ethyl)-N,N-dimethylbenzamide (**X21464**, 28.5 mg, 24.7%) as a white solid. $[M+H]^+ = 462$. ^1H NMR (DMSO-d_6, 400 MHz) δ 2.91 (6H, d), 3.29 (2H, t), 3.82 (3H, s), 4.74 (2H, t), 7.06 (1H, dd), 7.17 (1H, d), 7.25 – 7.35 (4H, m), 7.49 (1H, d), 7.76 (1H, d), 8.27 (1H, dd), 8.71 (1H, s), 9.11 (1H, dd).

Synthesis of X22317

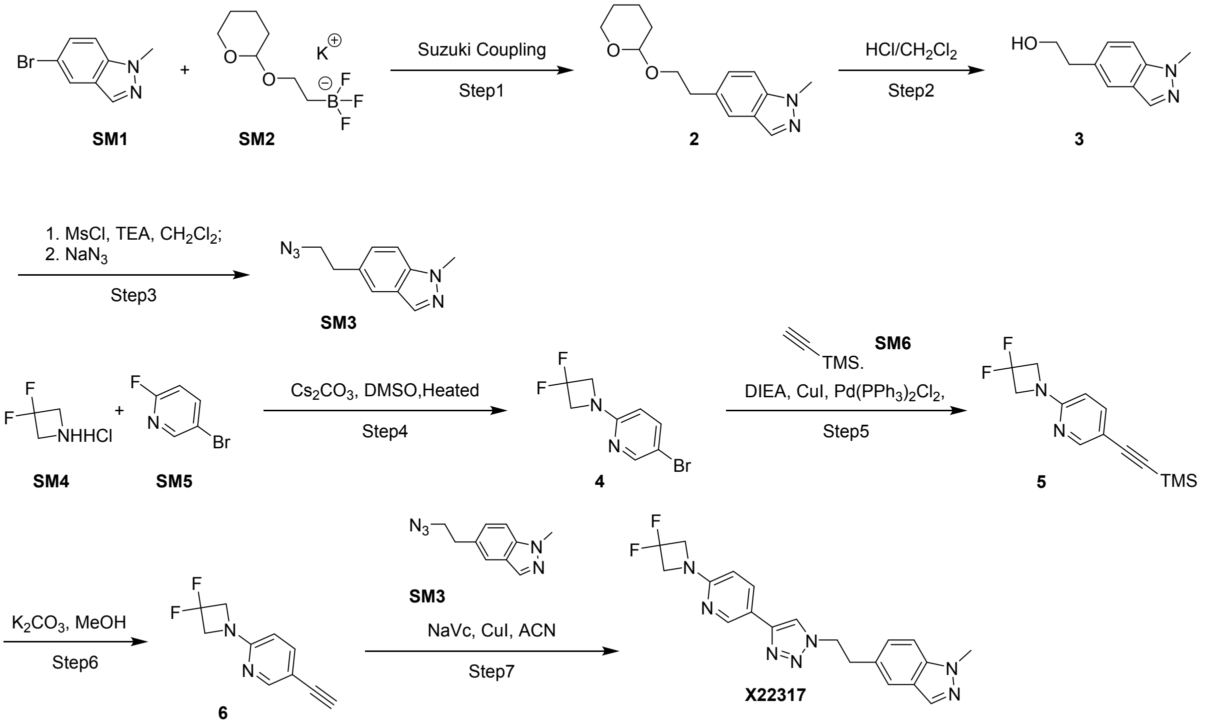

1-methyl-5-(2-((tetrahydro-2H-pyran-2-yl)oxy)ethyl)-1H-indazole (2)

To a mixture of **SM1** (4.9 g, 23.3 mmol), **SM2** (5.66 g, 24.0 mmol) and Cs_2CO_3 (15.32 g, 47.0 mmol) in Toluene/H_2O (100 mL/20 mL) was added Pd catalyst (CAS: 887919-35-9) (1.65 g, 2.33 mmol) and stirred at 100 degrees for overnight under N_2 atmosphere. The mixture was filtrated through a pad of celite. The filtrate was concentrated in vacuum to give the residue, which was redissolved in dichloromethane (100 mL), washed with brine (100 mL) and extracted with dichloromethane (100 mL x 3). The combined organic layers were dried over Na_2SO_4, filtered off and concentrated in vacuo to give the residue, which was purified by flash column chromatography (petroleum ether/ethyl acetate, 1/2) to furnish **2** (2.61 g, 43%) as a white solid. LC-MS $(M+H)^+$ =261.1

2-(1-methyl-1H-indazol-5-yl)ethan-1-ol (3)

To a solution of **2** (2.61 g, 10.0 mmol) in dichloromethane (30 mL) was added 2M aq. HCl (10 mL) and stirred at room temperature for 4 h. The reaction was quenched with saturated aq. $NaHCO_3$ (20 mL) and separated. The aqueous phase was extracted with dichloromethane (50 mL x 3). The combined organic layers were dried over Na_2SO_4,

filtered off and concentrated in vacuo to give the residue, which was purified by flash column chromatography (petroleum ether/ethyl acetate, 1/1) to furnish **3** (1.5 g, 85%) as a white solid. LC-MS $(M+H)^+$ =177.1

5-(2-azidoethyl)-1-methyl-1H-indazole (SM3)

To a solution of **3** (1.5 g, 8.8 mmol) and TEA (1.76 g, 17.6 mmol) in dichloromethane (50 mL) was slowly added a solution of MsCl (1.15 g, 10.0 mmol) in dichloromethane (20 mL) at 0 degrees and stirred at room temperature for overnight. The mixture was washed saturated aq. $NaHCO_3$ (30 mL) and extracted with dichloromethane (50 mL x 3). The combined organic layers were dried over Na_2SO_4, filtered off and concentrated in vacuo to give the residue. The residue was dissolved in dry DMF (15 mL) and NaN_3 (572.0 mg, 8.8 mmol) was added and stirred at room temperature for overnight. The mixture was washed saturated aq. $NaHCO_3$ (20 mL) and extracted with dichloromethane (30 mL x 3). The combined organic layers were dried over Na_2SO_4, filtered off and concentrated in vacuo to give the residue, which was purified by flash column chromatography (petroleum ether/ethyl acetate, 1/1) to furnish product **SM3** (1.3 g, 73%) as a white solid. LC-MS $(M+H)^+$ =202.0

5-bromo-2-(3,3-difluoroazetidin-1-yl)pyridine (4)

A reaction mixture of SM4 (500 mg, 3.38 mmol), SM5 (678 mg, 3.88 mmol) and Cs_2CO_3 (2525 mg, 7.75 mmol) in DMSO (10 mL) was stirred at 80 degree for 2h. The solvent was removed in vacuum and the residue was purified by Combiflush column (CH_2Cl_2: MeOH=100:1- 10:1) to give **4** (330 mg, 34%yield) as a white solid. LC-MS $(M+H)^+$ = 248.9.

2-(3,3-difluoroazetidin-1-yl)-5-((trimethylsilyl)ethynyl)pyridine (5)

A reaction mixture of **4** (310 mg, 1.25 mmol), SM3 (184 mg, 1.88 mmol), $Pd(PPh_3)_2Cl_2$ (88 mg, 0.13 mmol), CuI (47 mg, 0.25 mmol) and DIEA (323mg, 2.50 mmol) in dioxane was stirred at 80 degrees centigrade under N_2 overnight. The solvent was removed in vacuum and the residue was purified by Combiflush column (CH_2Cl_2: MeOH=100:1-10:1) to give **5** (130 mg, 39% yield) as a white solid. LC-MS $(M+H)^+$ = 267.0.

2-(3,3-difluoroazetidin-1-yl)-5-ethynylpyridine (6)

A reaction mixture of 3 (120 mg, 0.45 mmol), SM2 (81 mg, 0.45 mmol) and K_2CO_3 (124 mg, 0.90 mmol) in MeOH (5 mL) was stirred at rt for 2h. The solvent was removed in vacuum and the residue was purified by Combiflush column (CH_2Cl_2: MeOH=100:1- 10:1) to give **6** (60 mg, 69%yield) as a white solid. LC-MS $(M+H)^+$ = 195.0.

5-(2-(4-(6-(3,3-difluoroazetidin-1-yl)pyridin-3-yl)-1H-1,2,3-triazol-1-yl)ethyl)-1-methyl-1H-indazole (X22317)

A reaction mixture of 6 (40 mg, 1.25 mmol), **SM3** (62 mg, 0.31mmol), NaVc (82 mg, 0.41mmol) and CuI (8 mg, 0.04mmol) in ACN (3 mL) was stirred at 80 degrees centigrade under N_2 overnight. The solvent was removed in vacuum and the residue was purified by Combiflush column (CH_2Cl_2: MeOH=100:1- 10:1) and Prep-HPLC (FA) to give **X22317** (8 mg, 10% yield) as a white solid. LC-MS $(M+H)^+$ = 396.1. ^{1}H NMR (400 MHz, DMSO-d_6) δ 8.55 (d, *J* = 1.6 Hz, 1H), 8.48 (s, 1H), 8.04 (dd, *J* = 8.6, 2.2 Hz, 1H), 7.95 (s, 1H), 7.55 (d, *J* = 5.7 Hz, 2H), 7.27 (dd, *J* = 8.8, 1.3 Hz, 1H), 6.71 (d, *J* = 8.6 Hz, 1H), 4.71 – 4.66 (m, 2H), 4.48-4.41 (m, 4H), 4.00 (s, 3H), 3.34 – 3.28 (m, 2H).

Synthesis of X21426

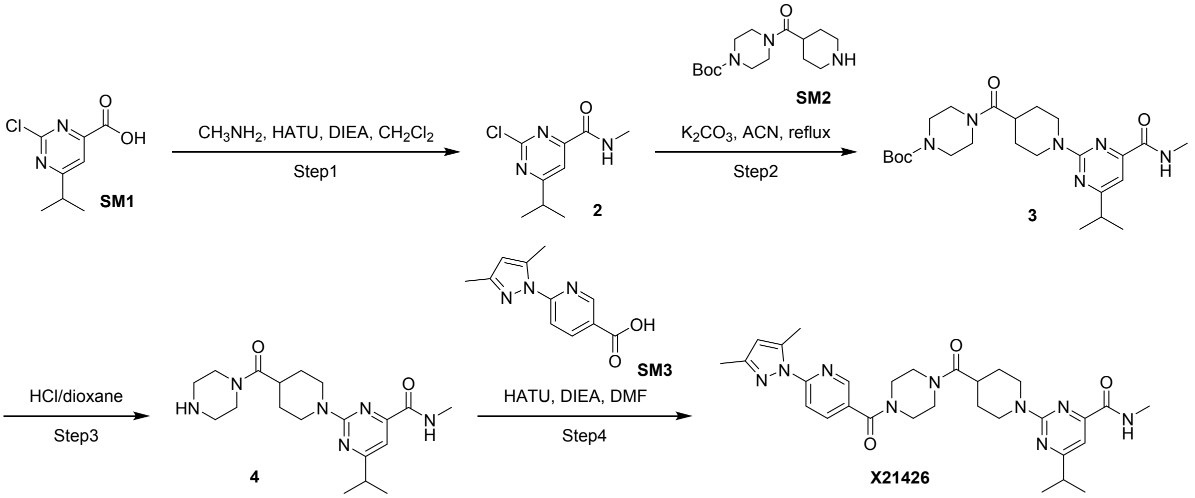

2-chloro-6-isopropyl-N-methylpyrimidine-4-carboxamide (2)

A mixture of **SM1** (400 mg, 2.0 mmol), $CH_3NH.HCl$ (272 mg, 4.0 mmol), HATU (1.14 g, 3.0 mmol) and DIEA (516 mg, 4.0 mmol) in CH_2Cl_2 (13 mL) was stirred at room temperature for 2 h. The mixture was washed with water (15 mL) and extracted with CH_2Cl_2 (10 mL) for three times. The combined organic layers were dried over Na_2SO_4, filtrated and concentrated under reduced pressure to give the residue, which was purified by combiflash to afford **2** (270 mg, 64 %) as a white solid. LC-MS $(M+H)^+$= 214.0

tert-butyl 4-(1-(4-isopropyl-6-(methylcarbamoyl)pyrimidin-2-yl)piperidine-4-carbonyl)piperazine-1-carboxylate (3)

A mixture of **2** (260 mg, 1.41 mmol), **SM2** (628 mg, 2.12 mmol), and K_2CO_3 (389 mg, 2.82 mmol) in ACN (13 mL) was stirred at 60 degrees for 3 h. The solvent was removed to give residue, which was dissolved in dichloromethane (10 mL), washed with water (20 mL) and extracted with dichloromethane (15 mL) for three times. The combined organic layers were dried over Na_2SO_4, filtrated and concentrated under reduced pressure to give the residue, which was purified by combiflash to afford **3** (400 mg, 60 %) as a white solid. LC-MS $(M+H)^+$= 475.2

6-isopropyl-N-methyl-2-(4-(piperazine-1-carbonyl)piperidin-1-yl)pyrimidine-4-carboxamide (4)

To a solution of **3** (390 mg, 0.82 mmol) in dichloromethane (9 mL) was added HCl/dioxane (4M, 1 mL) and the solution was stirred at room temperature for 2 h. The reaction solution was concentrated under vacuum to give **4** (270 mg, 88 %) as a white solid, which was directly used for the next step without further purification. LC-MS $(M+H)^+$= 375.2

2-(4-(4-(6-(3,5-dimethyl-1H-pyrazol-1-yl)nicotinoyl)piperazine-1-carbonyl)piperidin-1-yl)-6-isopropyl-N-methylpyrimidine-4-carboxamide (X21426)

A mixture of **SM3** (120 mg, 0.55 mmol), **4** (206 mg, 0.55 mmol), HATU (315 mg, 0.83 mmol) and DIEA (142 mg, 1.10 mmol) in DMF (6 mL) was stirred at room temperature for 2 h. The mixture was washed with water (30 mL) and extracted with ethyl acetate (20 mL) for three times. The combined organic layers were dried over Na_2SO_4, filtrated and concentrated in vacuum to give the residue, which was purified by Prep-HPLC to afford the product **X21426** (35 mg, 11 %) as a white solid.

^{1}H NMR (400 MHz, DMSO-d_6): δ 8.67 (d, J = 4.6 Hz, 1H), 8.53 (d, J = 2.0 Hz, 1H), 8.01 (dd, J = 8.5, 2.2 Hz, 1H), 7.90 (dd, J = 8.5, 0.5 Hz, 1H), 7.01 (s, 1H), 6.16 (s, 1H), 4.90-4.80 (m, 2H), 3.75-3.54 (m, 8H), 3.39 – 3.33 (m, 1H), 3.00-2.93 (m, 2H), 2.91-2.84 (m, 1H), 2.80 (d, J = 4.9 Hz, 3H), 2.62 (d, J = 0.6 Hz, 3H), 2.22 (s, 3H), 1.78-1.69 (m, 2H), 1.56-1.41 (m, 2H), 1.20 (d, J = 6.9 Hz, 6H). LC-MS $(M+H)^+$= 574.3

Synthesis of X23488

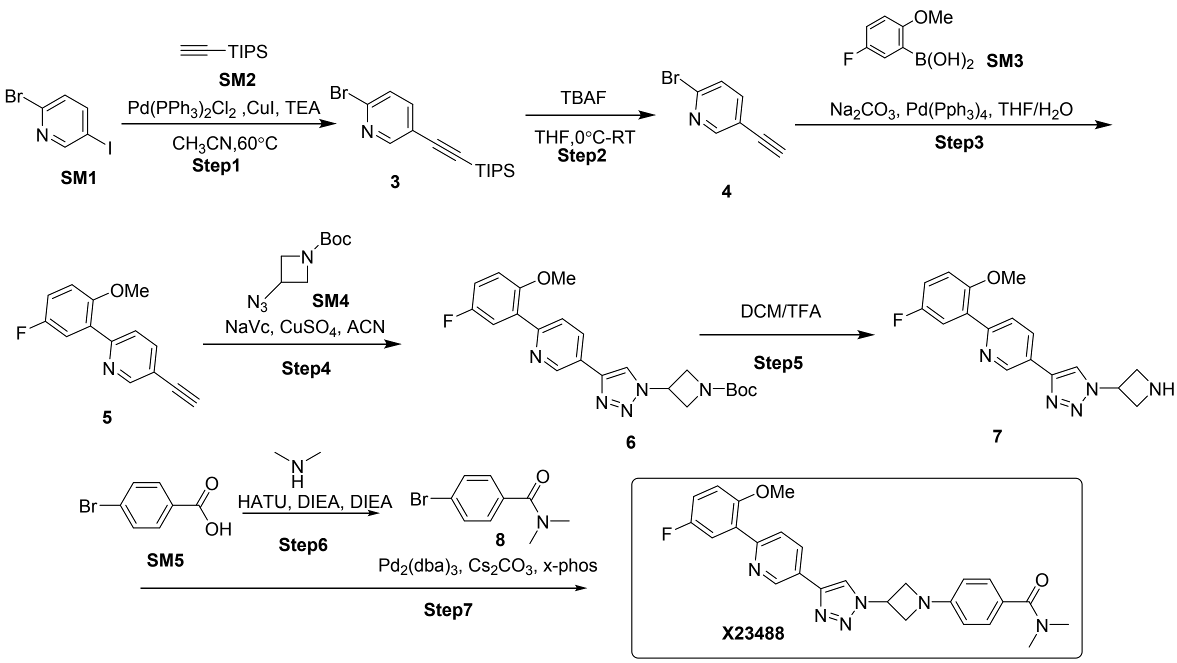

Synthesis of 2-bromo-5-((triisopropylsilyl)ethynyl)pyridine (3)

To a solution of **SM1** (2.84 g, 10.0 mmol) in CH_3CN (50 mL) were added **SM2** (2 g, 11.0 mmol), $Pd(PPh_3)_2Cl_2$ (702 mg, 1.0 mmol), CuI (191 mg, 1.0 mmol) and TEA (3 g, 30.0 mmol), the mixture was stirred at 60°C for 6h. The mixture was concentrated in vacuo to remove organic, extracted by EA (60 mL x 3) and H_2O (50 mL). The combined organic

layer was washed with brine (60 mL), then dried over with anhydrous Na_2SO_4. After filtration, the filtrate was concentrated, purified by silica gel chromatography (PE/EA=2/1) to give **3** (3 g, 89.6 %) as a white solid. LC-MS $(M+H)^+$ = 338.1.

2-(2,5-dimethoxyphenyl)-5-ethynylpyridine (4)

To a solution of **3** (722 mg, 1.8 mmol) in THF (20 mL) was added TBAF (3.6 mmol, 3.6 mL, 1.0 M in THF) at 0°C. Then stirred at RT for 2h. The mixture was extracted by EA (40 mL × 3) and H_2O (50 mL). The combined organic layer was washed with brine (50 mL), then dried over with anhydrous Na_2SO_4. After filtration, the filtrate was concentrated, purified by silica gel chromatography (PE/EA=3/1) to give **4** (400 mg, 92.1 %) as a white solid. LC-MS $(M+H)^+$ = 240.1.

Synthesis of 5-ethynyl-2-(5-fluoro-2-methoxyphenyl)pyridine (5)

To a solution of **4** (700 mg, 2.75 mmol) in THF/H_2O (10 mL/10 mL) were added SM3 (93 7 mg, 5.5 mmol), $Pd(PPh_3)$ (318 mg, 0.27 mmol) and Na_2CO_3 (583 mg, 5.5 mmol), the m ixture was stirred at 70°C for 8h under N_2. The mixture was concentrated in vacuo and d iluted with EtOAc (100 mL). The mixture was washed with water (50 mL) and brine (50 mL x 2), the separated organic layer was dried over Na_2SO_4 and concentrated in vacuo. The residue was purified by silica gel column (200-300 mesh, PE:EtOAc=10:1) to give **5** (368 mg, 58.8% yield) as a yellow solid. LC-MS $(M+H)^+$ = 228.1.

Synthesis of tert-butyl 3-(4-(6-(5-fluoro-2-methoxyphenyl)pyridin-3-yl)-1H-1,2,3-tria zol-1-yl)azetidine-1-carboxylate (6)

To a solution of **5** (368 mg, 1.62 mmol) in ACN (10 mL) were added SM4 (320 mg, 1.62 mmol), $CuSO_4$ (51.7 mg, 0.324 mmol) and NaVc (128 mg, 0.648 mmol), the mixture was stirred at 80°C for 24h. The mixture was concentrated in vacuo and diluted with EtOAc (100 mL). The mixture was washed with water (100 mL) and brine (50 mL x 2), the separ ated organic layer was dried over Na_2SO_4 and concentrated in vacuo. The residue was purified by silica gel column (200-300 mesh, PE:EtOAc = 1:1) to give **6** (540 mg,78% yie ld) as a yellow solid. LC-MS $(M+H)^+$ = 426.0.

Synthesis of 5-(1-(azetidin-3-yl)-1H-1,2,3-triazol-4-yl)-2-(5-fluoro-2-methoxyphenyl) pyridine (7)

To a solution of **6** (540 mg, 1.27 mmol) in DCM (10 mL) was added TFA (3 mL), the mixture was stirred at room temperature for 2h. The mixture was concentrated in vacuo to give **7** (430 mg crude) as a yellow oil. LC-MS $(M+H)^+$ = 326.1.

Synthesis of 4-bromo-N,N-dimethylbenzamide (8)

To a solution of **SM5** (500 mg, 2.5 mmol) in DCM (15 mL) were added dimethylamine (3 25 mg, 3.0 mmol), HATU (1.1 g, 3 mmol) and DIEA (967 mg, 7.5 mmol), the mixture wa s stirred at room temperature for 6h. The mixture was diluted with DCM (50 mL) and wa shed brine (50 mL x 2). The separated organic layer was dried over Na_2SO_4 and concen trated in vacuo. The residue was purified by silica gel column (200-300 mesh, PE:EtOA c = 5:1) to give **8** (500 mg, 88.0% yield) as a yellow oil. LC-MS $(M+H)^+$ = 228.1.

Synthesis of 4-(3-(4-(6-(5-fluoro-2-methoxyphenyl)pyridin-3-yl)-1H-1,2,3-triazol-1-yl)azetidin-1-yl)-N,N-dimethylbenzamide (X23488)

To a solution of **7** (100 mg, 0.31 mmol) in ACN (10 mL) were added **8** (91 mg, 0.40mmo l), $Pd_2(dba)_3$ (44 mg, 0.077 mmol), Cs_2CO_3 (302 mg, 0.93 mmol) and X-PHOS (73 mg, 0 .154 mmol), the mixture was stirred at 80°C for 7h under N_2。The mixture was concentra ted in vacuo and diluted with EtOAc (30 mL). The mixture was washed with water (50 m L) and brine (50 mL x 2), the separated organic layer was dried over Na_2SO_4 and conce ntrated in vacuo. The residue was purified by prep-HPLC to give **X23488** (43.3 mg ,29.8 % yield.98.8% purity) as a white solid. LC-MS (M+H)+ = 473.0.

^{1}H NMR (400 MHz, DMSO) δ 9.17 (dd, *J* = 2.2, 0.7 Hz, 1H), 9.09 (s, 1H), 8.30 (dd, *J* = 8 .3, 2.3 Hz, 1H), 8.05 (dd, *J* = 8.3, 0.7 Hz, 1H), 7.65 (dd, *J* = 9.8, 3.2 Hz, 1H), 7.33 - 7.33 (m, 2H), 7.30 - 7.23 (m, 1H), 7.20 (dd, *J* = 9.1, 4.6 Hz, 1H), 6.60 - 6.56 (m, 2H), 5.80 - 5. 70 (m, 1H), 4.51 (t, *J* = 8.0 Hz, 2H), 4.29 (dd, *J* = 8.4, 5.2 Hz, 2H), 3.87 (s, 3H), 2.97 (s, 6H).

Synthesis of X21352

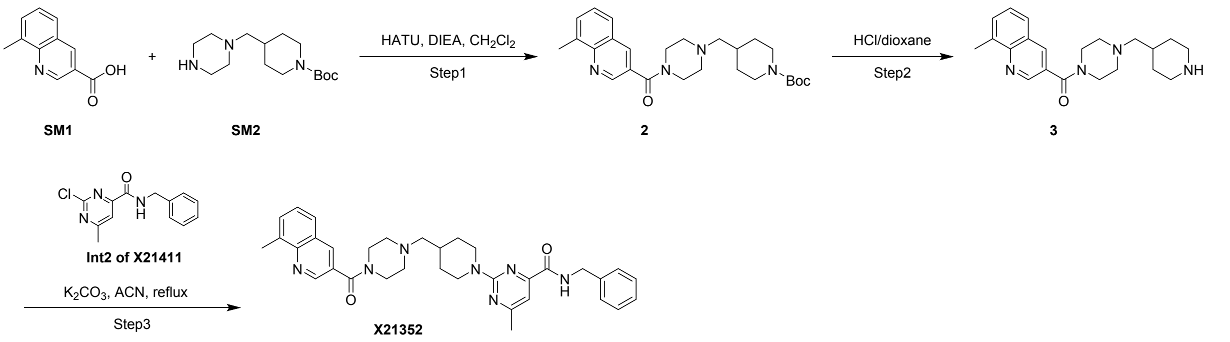

***tert*-butyl 4-((4-(8-methylquinoline-3-carbonyl)piperazin-1-yl)methyl)piperidine-1-carboxylate (2)**

To a solution of **SM1** (187 mg, 1.00 mmol), HATU (425 mg, 1.50 mmol) in CH2Cl2 (5 mL) were added **SM2** (283 mg, 1.50 mmol), DIEA (258 mg, 2.00 mmol) at room temperature. The reaction mixture was stirred at room temperature for 2 h. The mixture was diluted with H_2O (30 mL) and extracted with CH_2Cl_2 (30 mLx3). The organic layer was dried over anhydrous Na_2SO_4. After filtration, the filtrate was concentrated under vacuum to give the crude product, which was purified by Combiflush column (CH_2Cl_2: MeOH=100:1- 10:1) to give **2** (200 mg, 44%) as a white solid. LC-MS $(M+H)^+$ = 452.6.

(8-methylquinolin-3-yl)(4-(piperidin-4-ylmethyl)piperazin-1-yl)methanone (3)

A solution of **2** (200 mg, 0.44 mmol) in 4 M HCl/dioxane (3 mL) was stirred at rt for 1h. The mixture was concentrated to give **3** (120 mg, crude) as a brown oil which was used for next step without further purification. LC-MS $(M+H)^+$ = 352.5.

N-benzyl-6-methyl-2-(4-((4-(8-methylquinoline-3-carbonyl)piperazin-1-yl)methyl)piperidin-1-yl)pyrimidine-4-carboxamide (X21352)

To a solution of 3 (120 mg, crude), **Int2 of X21411** (133 mg, 0.51 mmol), K_2CO_3 (94 mg, 0.68 mmol) in CH_3CN (3 mL) was stirred at 80°C for 2 h. The reaction mixture was concentrated under vacuum. The residue was diluted H_2O (30 mL) and extracted with CH_2Cl_2 (30 mLx3). The organic layer was dried over anhydrous Na_2SO_4. After filtration, the filtrate was concentrated under vacuum to give the crude product, which was purified by Prep-HPLC (FA) to give the product **X21352** (9.9 mg, 4%) as a white solid. LC-MS $(M+H)^+$ = 577.7.

^{1}H NMR (400 MHz, d6-DMSO) δ 9.25 (t, J = 6.5 Hz, 1H), 8.92 (d, J = 2.2 Hz, 1H), 8.43 (d, J = 2.2 Hz, 1H), 7.90 (d, J = 8.0 Hz, 1H), 7.70 (d, J = 7.0 Hz, 1H), 7.60 – 7.53 (m, 1H), 7.38 – 7.16 (m, 5H), 7.00 (s, 1H), 4.82 (d, J = 8.1 Hz, 2H), 4.48 (d, J = 6.5 Hz, 2H), 3.71 (s, 2H), 3.42 (s, 2H), 2.87 (t, J = 7.9 Hz, 2H), 2.74 (s, 3H), 2.48 (s, 2H), 2.35 (s, 5H), 2.20 (d, J = 6.7 Hz, 2H), 1.79 (d, J = 8.0 Hz, 3H), 1.12-0.98 (m, 2H).

Synthesis of X21411

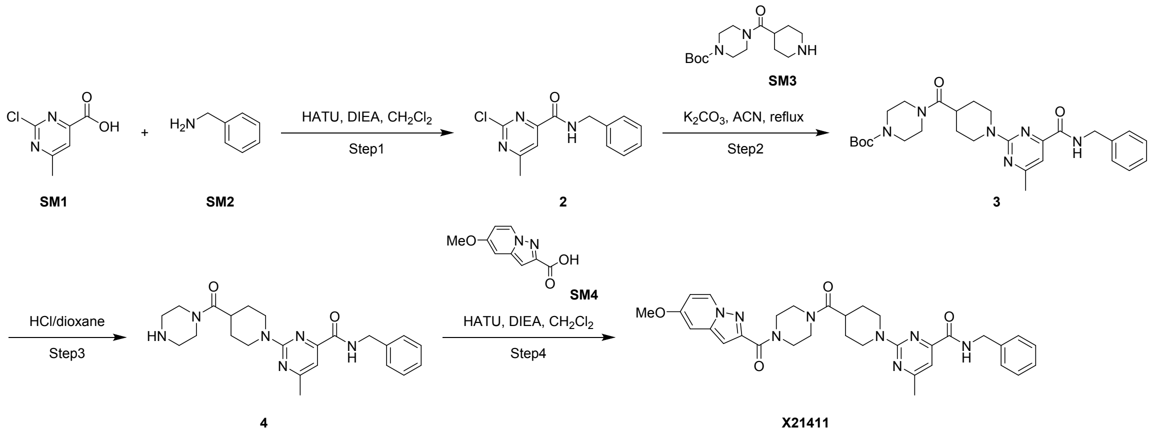

N-benzyl-2-chloro-6-methylpyrimidine-4-carboxamide (2)

A solution of **SM1** (3.0 g, 17.44 mmol), **SM2** (1.868 g, 17.44 mmol), HATU (9.942 g, 26.16 mmol) and DIEA (4.5 g, 34.88 mmol) in CH_2Cl_2 (100 mL) was stirred at room temperature for 2h. The mixture was washed with aq. citric acid (10%, 200 mL), saturated aq. $NaHCO_3$ (200 mL) and brine (200 mL). The organic layer was dried over anhydrous Na_2SO_4. After filtration, the solvent was removed in vacuum and the residue was purified by Combiflush column (CH_2Cl_2: MeOH=100:1- 10:1) to give **2** (1.9 g, 42%) as a yellow solid. LC-MS $(M+H)^+$ = 262.0.

tert-butyl 4-(1-(4-(benzylcarbamoyl)-6-methylpyrimidin-2-yl)piperidine-4-carbonyl)piperazine-1-carboxylate (3)

A solution of **2** (1.9 g, 7.28 mmol), **SM3** (2.163 g, 7.28 mmol) and K_2CO_3 (2.0 g, 14.56 mmol) in MeCN (50 mL) was stirred at 80 degree for 2h. The solvent was removed in vacuum and the residue was purified by Combiflush column (CH_2Cl_2: MeOH=100:1- 10:1) to give **3** (2.3 g, 61%) as a brown oil. LC-MS $(M+H)^+$ = 522.8.

N-benzyl-6-methyl-2-(4-(piperazine-1-carbonyl)piperidin-1-yl)pyrimidine-4-carboxamide (4)

To a solution of **3** (2.3 g, 4.40 mmol) in dichloromethane (10 mL) was added HCl/dioxane (4M, 2 mL) was stirred at room temperature for 1h. The mixture was concentrated to give **4** (2.5 g, crude) as a brown oil which was used for next step without further purification. LC-MS $(M+H)^+$ = 422.8.

N-benzyl-2-(4-(4-(5-methoxypyrazolo[1,5-a]pyridine-2-carbonyl)piperazine-1-carbonyl)piperidin-1-yl)-6-methylpyrimidine-4-carboxamide (X21411)

A solution of **4** (100 mg, 0.24 mmol), **SM4** (45 mg, 0.24 mmol), HATU (135 mg, 0.36 mmol) and DIEA (61 mg, 0.47 mmol) in DMF (3 mL) was stirred at r.t for 2h. The mixture was diluted with ethyl acetate (15 mL), washed with aq. citric acid (10%, 20 mL), saturated $NaHCO_3$ aq. (20 mL) and brine (20 mL). The organic layer was separated and aqueous phase was extracted with ethyl acetate (5 mL x 3). The combined organic layers were dried over anhydrous Na_2SO_4. After filtration, the solvent was removed in vacuum and the residue was purified using Prep-HPLC (TFA) to give **X21411** (42 mg, 31%) as a light-yellow solid.

^1H NMR (400 MHz, DMSO-d_6): δ 9.28 (t, J = 6.2 Hz, 1H), 8.56 (t, J = 6.2 Hz, 1H), 7.34 – 7.28 (m, 4H), 7.23 (t, J = 7.5 Hz, 1H), 7.09 (s, 1H), 7.03 (s, 1H), 6.68 (d, J = 6.4 Hz, 2H), 4.86 (d, J = 10.4 Hz, 2H), 4.49 (d, J = 6.4 Hz, 2H), 3.95 (s, 1H), 3.84 (s, 4H), 3.75 -3.45 (m, 6H), 3.06 – 2.83 (m, 3H), 2.37 (s, 3H), 1.80 – 1.65 (m, 2H), 1.60 – 1.45 (m, 2H). LC-MS $(M+H)^+$ = 596.7.

Synthesis of X21408

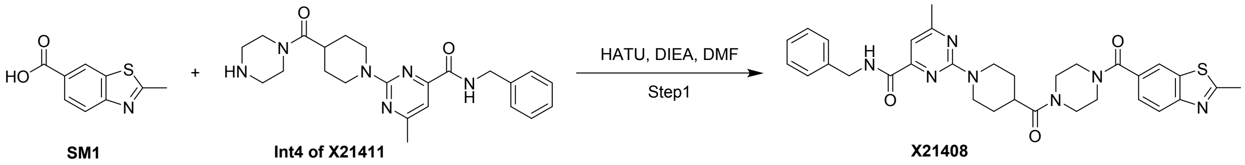

N-benzyl-6-methyl-2-(4-(4-(2-methylbenzo[d]thiazole-6-carbonyl)piperazine-1-carbonyl)piperidin-1-yl)pyrimidine-4-carboxamide (X21408)

A solution of **Int4** of **X21411** (100 mg, 0.24 mmol), **SM1** (46 mg, 0.24 mmol), HATU (135 mg, 0.36 mmol) and DIEA (61 mg, 0.47 mmol) in DMF (3 mL) was stirred at r.t for 2h. The mixture

was washed with aq. citric acid (10%, 20 mL), aq. saturated $NaHCO_3$ (20 mL), brine (20 mL) and extracted with ethyl acetate. The organic layer was dried over anhydrous Na_2SO_4. After filtration, the solvent was removed in vacuum and the residue was purified using Prep-HPLC (TFA) to give **X21408** (46 mg, 33%) as a light-yellow solid.

^{1}H NMR (400 MHz, DMSO-d_6): δ 9.28 (t, J = 6.4 Hz, 1H), 8.16 (s, 1H), 7.97 (d, J = 8.3 Hz, 1H), 7.53 (d, J = 8.3 Hz, 1H), 7.38 – 7.27 (m, 4H), 7.24 (t, J = 5.9 Hz, 1H), 7.03 (s, 1H), 4.85 (d, J = 11.9 Hz, 2H), 4.49 (d, J = 6.4 Hz, 2H), 3.76-3.31 (m, 8H), 3.04-2.86 (m, 3H), 2.83 (s, 3H), 2.37 (s, 3H), 1.72 (d, J = 12.0 Hz, 2H), 1.58 – 1.43 (m, 2H). LC-MS $(M+H)^+$ = 597.7

Synthesis of X22307

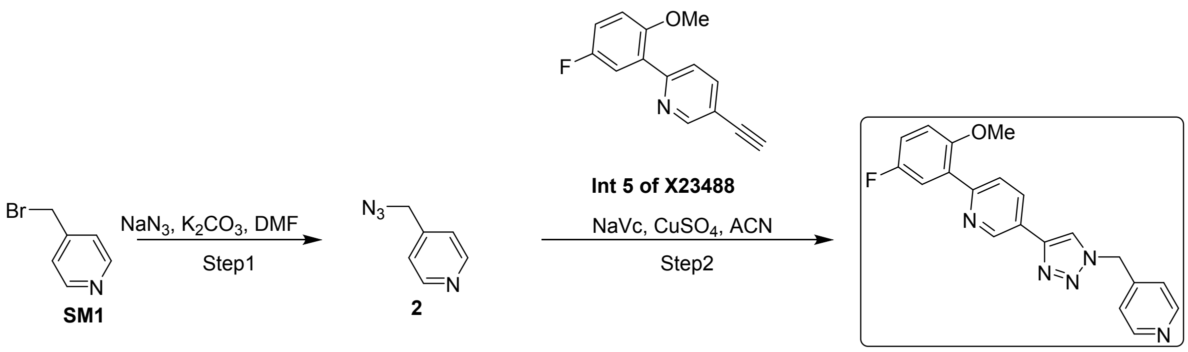

X22307

4-(azidomethyl)pyridine (2)

A mixture of **SM1** (300 mg, 1.75 mmol), K_2CO_3 (242 mg, 1.75 mmol) in DMF (3 mL) was stirred at rt for 0.5 h. Then the NaN_3(171 mg, 2.63 mmol) was added to the reaction mixture and stirred another 12 h. The reaction mixture was quenched by NaClO (20 mL). The mixture was extracted with EtOAc(3*10mL) and the combined organic was concentrated under reduce pressure to give **2** (210 mg, 89% yield) as yellow solid. LC-MS $(M+H)^+$ = 135

2-(5-fluoro-2-methoxyphenyl)-5-(1-(pyridin-4-ylmethyl)-1H-1,2,3-triazol-4-yl)pyridine (X22307)

A mixture of **2** (30 mg, 0.22 mmol), **Int 5 of X23488** (51 mg, 0.22 mmol), NaVc (89 mg, 0.44 mmol), $CuSO_4$(36 mg, 0.22 mmol) in ACN (2 mL) was stirred at rt for overnight. The reaction mixture was filtered and the filtrate was concentrated under reduced pressure to give the residue

which was purified by Prep-TLC (CH2Cl2:MeOH=20:1) to give **X22307** (16 mg, 20% yield) as white solid. LC-MS $(M+H)^+$ = 362

1H NMR (400 MHz, CDCl3) δ = 9.06 (dd, *J*=4.8 Hz, 3.3 Hz, 1H), 8.72 – 8.61 (m, 2H), 8.26 (dd, *J*=8.3 Hz, 2.3 Hz, 1H), 8.04 – 7.94 (m, 1H), 7.88 (s, 1H), 7.61 (dt, *J*=5.5 Hz, 2.7 Hz, 1H), 7.21 – 7.15 (m, 2H), 7.10 – 7.03 (m, 1H), 6.98 – 6.90 (m, 1H), 5.65 (s, 2H), 3.86 (s, 3H).

Synthesis of X22346

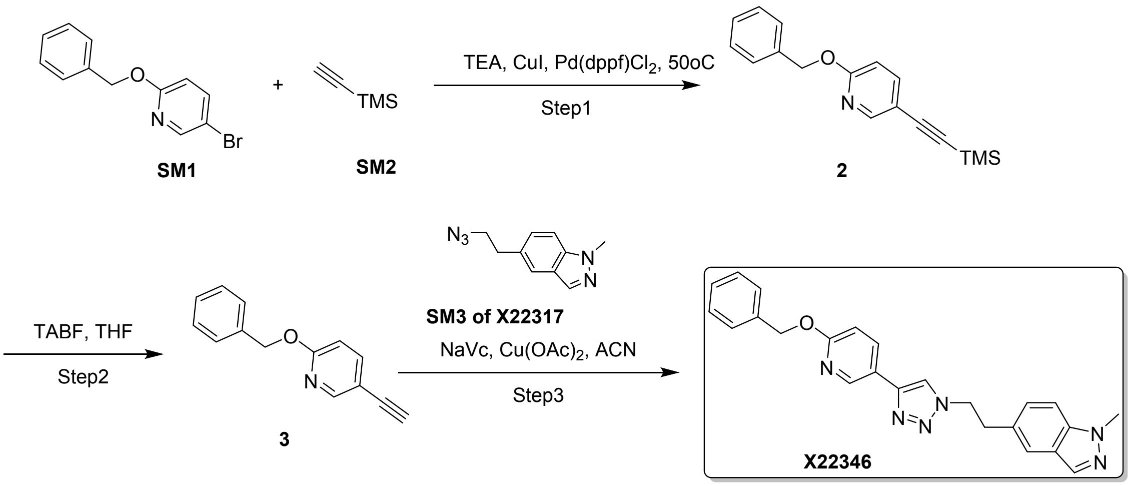

2-(benzyloxy)-5-((trimethylsilyl)ethynyl) pyridine (2)

A mixture of **SM1** (263 mg, 1 mmol), **SM2** (490 mg, 5 mmol), TEA (303 mg, 3 mmol), Pd(dppf)Cl_2 (40 mg, 0.05 mmol) and CuI (38 mg, 0.2 mmol) in THF (5mL) was stirred at 50 degrees for 4 h under N_2 atmosphere. Reaction mixture was washed with water (20 mL), extracted with dichloromethane (20 mL) for three times. the combined organic layers were dried over Na_2SO_4, filtrated, and concentrated to give the residue, which was purified by combi flash to afford **2** (150 mg, 53 %) as a yellow oil. LC-MS $(M+H)^+$= 282.1.

2-(benzyloxy)-5-ethynylpyridine (3)

To a solution of **2** (281 mg, 1 mmol) in THF (5 mL) was added TBAF (2 mL) and the solution was stirred at room temperature for 2 h. Reaction mixture was washed with water (20 mL), extracted with dichloromethane (20 mL) for three times. the combined organic layers were dried over Na_2SO_4, filtrated, and concentrated to give the residue, which was purified by combi flash to afford **3** (180 mg, 86 %) as a yellow oil. LC-MS $(M+H)^+$= 210.0.

5-(2-(4-(6-(benzyloxy) pyridin-3-yl)-1H-1,2,3-triazol-1-yl) ethyl)-1-methyl-1H-indazole (X22346)

A mixture of **3** (100 mg, 0.47 mmol), **SM3 of X22317** (95 mg, 0.47 mmol), NaVc (376 mg, 0.94 mmol) and $CuSO_4$ (76 mg, 0.47 mmol) in ACN (5 mL) was stirred at room temperature for 12 h. The mixture was diluted with water (40 mL) and extracted with dichloromethane (20 mL) for

three times. The combined organic layers were dried over Na_2SO_4, filtrated, and concentrated to give the residue, which was purified by Prep-HPLC to afford the product **X22346** (20 mg, 10 %) as a white solid. LC-MS $(M+H)^{+}$= 411.1.

1H NMR (400 MHz, DMSO-d_6) δ 8.57 – 8.53 (m, 1H), 8.49 (s, 1H), 8.06 (dd, *J* = 8.6, 2.4 Hz, 1H), 7.91 (d, *J* = 0.6 Hz, 1H), 7.54 – 7.49 (m, 2H), 7.46 – 7.39 (m, 2H), 7.38 – 7.32 (m, 2H), 7.32 – 7.26 (m, 1H), 7.26 – 7.20 (m, 1H), 6.93 (dd, *J* = 8.6, 0.4 Hz, 1H), 5.34 (s, 2H), 4.66 (t, *J* = 7.2 Hz, 2H), 3.97 (s, 3H), 3.28 – 3.24 (m, 2H).

Synthesis of X23546

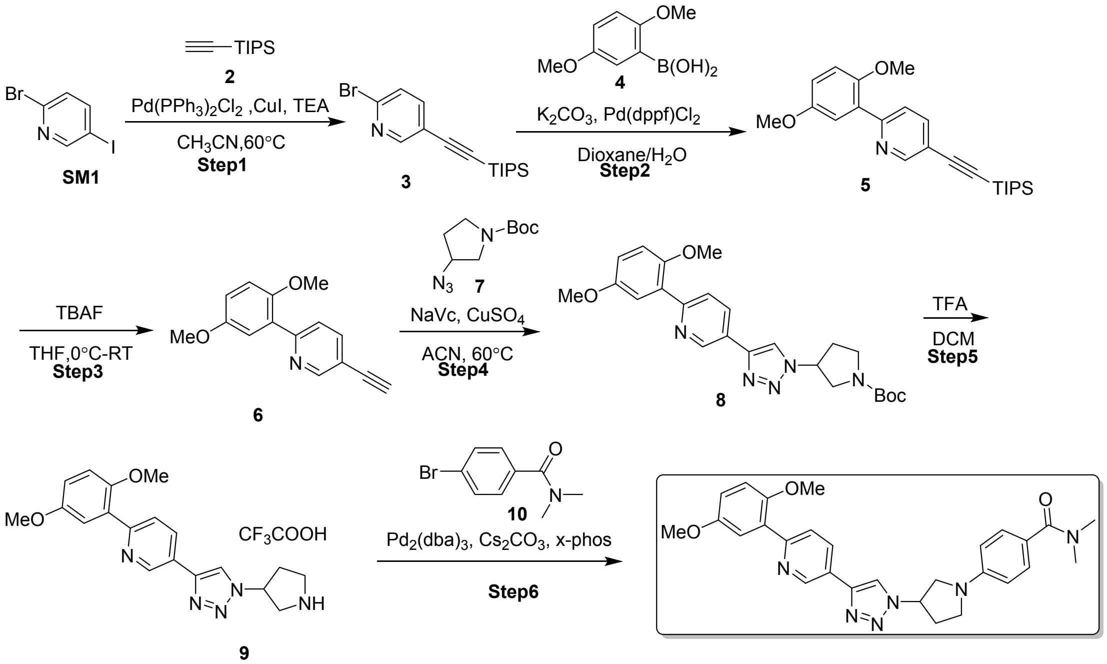

2-bromo-5-((triisopropylsilyl)ethynyl)pyridine (3)

To a solution of **SM1** (2.84 g, 10.0 mmol) in CH_3CN (50 mL) were added **2** (2 g, 11.0 mmol), $Pd(PPh_3)_2Cl_2$ (702 mg, 1.0 mmol), CuI (191 mg, 1.0 mmol) and TEA (3 g, 30.0 mmol), then stirred at 60°C for 6h. The mixture was concentrated in vacuo to remove organic, extracted by EA (60 mL x 3) and H_2O (50 mL). The combined organic layer was washed with brine (60 mL), then dried over with anhydrous Na_2SO_4. After filtration, the filtrate was concentrated, purified by silica gel chromatography (PE/EA=2/1) to give **3** (3020 mg, 89.6 %) as a white solid. LC-MS $(M+H)^{+}$ = 338.1.

^{1}H NMR (400 MHz, DMSO) δ 8.49 (dd, J = 2.4, 0.5 Hz, 1H), 7.85 - 7.82 (m, 1H), 7.68 (dd, J = 8.3, 0.6 Hz, 1H), 1.13 - 1.11 (m, 21H).

2-(2,5-dimethoxyphenyl)-5-((triisopropylsilyl)ethynyl)pyridine (5)

To a solution of **3** (677 mg, 2.0 mmol) in Dioxane/H_2O (30/5 mL) were added **4** (364 mg, 2.0 mmol), Pd(dppf)Cl_2 (147 mg, 0.2 mmol) and K_2CO_3 (553 mg, 4.0 mmol), then stirred at 80°C for 6h. The mixture was concentrated in vacuo to remove organic, extracted by EA (60 mL x 3) and H_2O (50 mL). The combined organic layer was washed with brine (100 mL), then dried over with anhydrous Na_2SO_4. After filtration, the filtrate was concentrated, purified by silica gel chromatography (PE/EA=2/1) to give **5** (720 mg, 91.1 %) as a white solid. LC-MS $(M+H)^+$ = 396.2.

^{1}H NMR (400 MHz, DMSO) δ 8.73 (dd, J = 2.1, 0.9 Hz, 1H), 7.94 - 7.88 (m, 2H), 7.37 (d, J = 3.2 Hz, 1H), 7.11 (d, J = 9.0 Hz, 1H), 7.01 (dd, J = 9.0, 3.2 Hz, 1H), 3.79 (s, 3H), 3.75 (s, 3H), 1.13 - 1.11 (m, 21H).

2-(2,5-dimethoxyphenyl)-5-ethynylpyridine (6)

To a solution of **5** (722 mg, 1.8 mmol) in THF (20 mL) was added TBAF (3.6 mmol, 3.6 mL, 1.0 M in THF) at 0°C, then stirred at RT for 2h. The mixture was extracted by EA (40 mL x 3) and H_2O (50 mL). The combined organic layer was washed with brine (50 mL), then dried over with anhydrous Na_2SO_4. After filtration, the filtrate was concentrated, purified by silica gel chromatography (PE/EA=3/1) to give **6** (400 mg, 92.1 %) as a white solid.

LC-MS $(M+H)^+$ = 240.1.

^{1}H NMR (400 MHz, DMSO) δ 8.77 - 8.75 (m, 1H), 7.93 (dd, J = 2.5, 1.6 Hz, 2H), 7.38 (d, J = 3.2 Hz, 1H), 7.11 (d, J = 9.0 Hz, 1H), 7.01 (dd, J = 9.0, 3.2 Hz, 1H), 4.48 (s, 1H), 3.80 (s, 3H), 3.75 (s, 3H).

tert-butyl 3-(4-(6-(2,5-dimethoxyphenyl)pyridin-3-yl)-1H-1,2,3-triazol-1-yl)pyrrolidine-1-carboxylate (8)

To a solution of **6** (400 mg, 1.67 mmol) in ACN (20 mL) were added **7** (391 mg, 1.84 mmol), NaVc (68 mg, 0.34 mmol) and $CuSO_4$ (28 mg, 0.17 mmol), then stirred at 60°C for 16h. The mixture was concentrated in vacuo to remove organic, extracted by EA (60 mL × 3) and H_2O (50

mL). The combined organic layer was washed with brine (100 mL), then dried over with anhydrous Na_2SO_4. After filtration, the filtrate was concentrated, purified by silica gel chromatography (PE/EA=2/1) to give **8** (660 mg, 86.1 %) as a white solid. LC-MS $(M+H)^+$ = 452.1.

2-(2,5-dimethoxyphenyl)-5-(1-(pyrrolidin-3-yl)-1H-1,2,3-triazol-4-yl)pyridine (9)

To a solution of **8** (660 mg, 1.46 mmol) in DCM/TFA (5 mL/ 5 mL) was stirred at 25°C for 3h. The mixture was concentrated in vacuo to give **9** (680 mg, 100 %) as a white solid. LC-MS $(M+H)^+$ = 352.1.

4-(3-(4-(6-(2,5-dimethoxyphenyl)pyridin-3-yl)-1H-1,2,3-triazol-1-yl)pyrrolidin-1-yl)-N,N-dimethylbenzamide (X23546)

To a solution of **9** (71 mg, 0.2 mmol) in ACN (20 mL) were added **10** (69 mg, 0.3 mmol), $Pd_2(dba)_3$ (20 mg, 0.04 mmol), X-phos (20 mg, 0.04 mmol) and Cs_2CO_3 (131 mg, 0.4 mmol), then stirred at 60°C for 16h. The mixture was concentrated in vacuo to remove organic, extracted by EA (60 mL × 3) and H_2O (50 mL). The combined organic layer was washed with brine (100 mL), then dried over with anhydrous Na_2SO_4. After filtration, the filtrate was concentrated, purified by silica gel chromatography (DCM/MeOH=20/1) to give a crude product, then purified by prep-TLC (DCM/MeOH=20/1) to give the product **X23546** (79.9 mg, 80.7 %) as a white solid. LC-MS $(M+H)^+$ = 499.2.

^{1}H NMR (400 MHz, DMSO) δ 9.14 - 9.13 (m, 1H), 8.86 (s, 1H), 8.24 (dd, *J* = 8.3, 2.3 Hz, 1H), 7.99 (d, *J* = 8.3 Hz, 1H), 7.40 (d, *J* = 3.2 Hz, 1H), 7.32 (d, *J* = 8.7 Hz, 2H), 7.11 (d, *J* = 9.0 Hz, 1H), 6.99 (dd, *J* = 9.0, 3.2 Hz, 1H), 6.63 (d, *J* = 8.8 Hz, 2H), 5.55 - 5.51 (m, 1H), 3.94 - 3.89 (m, 1H), 3.80 (s, 4H), 3.76 (s, 3H), 3.68 - 3.61 (m, 1H), 3.55 - 3.48 (m, 1H), 2.96 (s, 6H), 2.70 - 2.65 (m, 1H), 2.60 - 2.55 (m, 1H).

Synthesis of X23479

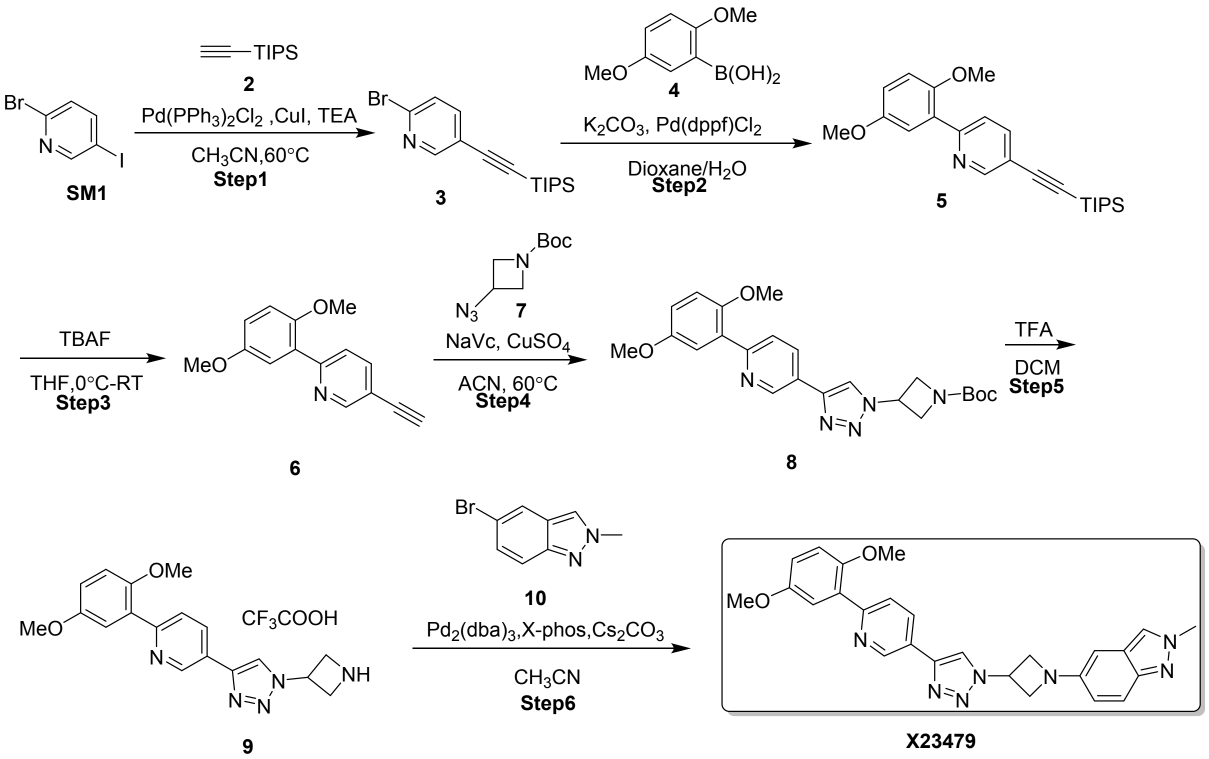

2-bromo-5-((triisopropylsilyl)ethynyl)pyridine (3)

To a solution of **SM1** (2.84 g, 10.0 mmol) in CH_3CN (50 mL) were added **2** (2 g, 11.0 mmol), $Pd(PPh_3)_2Cl_2$ (702 mg, 1.0 mmol), CuI (191 mg, 1.0 mmol) and TEA (3 g, 30.0 mmol), the mixture was stirred at 60°C for 6h. The mixture was concentrated in vacuo to remove organic, extracted by EA (60 mL x 3) and H_2O (50 mL). The combined organic layer was washed with brine (60 mL), then dried over with anhydrous Na_2SO_4. After filtration, the filtrate was concentrated, purified by silica gel chromatography (PE/EA=2/1) to give **3** (3 g, 89.6 %) as a white solid. LC-MS $(M+H)^+$ = 338.1.

2-(2,5-dimethoxyphenyl)-5-((triisopropylsilyl)ethynyl)pyridine (5)

To a solution of **3** (677 mg, 2.0 mmol) in Dioxane/H_2O (30/5 mL) were added **4** (364 mg, 2.0 mmol), $Pd(dppf)Cl_2$ (147 mg, 0.2 mmol) and K_2CO_3 (553 mg, 4.0 mmol), Then stirred at 80°C for 6h. The mixture was concentrated in vacuo to remove organic, extracted by EA (60 mL × 3) and H_2O (50 mL). The combined organic layer was washed with brine (100 mL), then dried over

with anhydrous Na_2SO_4. After filtration, the filtrate was concentrated, purified by silica gel chromatography (PE/EA=2/1) to give **5** (720 mg, 91.1 %) as a white solid.
LC-MS $(M+H)^+$ = 396.2.

2-(2,5-dimethoxyphenyl)-5-ethynylpyridine (6)

To a solution of **5** (722 mg, 1.8 mmol) in THF (20 mL) was added TBAF (3.6 mmol, 3.6 mL, 1.0 M in THF) at 0°C. Then stirred at RT for 2h. The mixture was extracted by EA (40 mL × 3) and H_2O (50 mL). The combined organic layer was washed with brine (50 mL), then dried over with anhydrous Na_2SO_4. After filtration, the filtrate was concentrated, purified by silica gel chromatography (PE/EA=3/1) to give **6** (400 mg, 92.1 %) as a white solid. LC-MS $(M+H)^+$ = 240.1.

tert-butyl 3-(4-(6-(2,5-dimethoxyphenyl)pyridin-3-yl)-1H-1,2,3-triazol-1-yl)azetidine-1-carboxylate (8)

To a solution of **6** (400 mg, 1.67 mmol) in ACN (20 mL) were added **7** (365 mg, 1.84 mmol), NaVc (68 mg, 0.34 mmol) and $CuSO_4$ (28 mg, 0.17 mmol), Then stirred at 60°C for 16h. The mixture was concentrated in vacuo to remove organic, extracted by EA (60 mL × 3) and H_2O (50 mL). The combined organic layer was washed with brine (100 mL), then dried over with anhydrous Na_2SO_4. After filtration, the filtrate was concentrated, purified by silica gel chromatography (PE/EA=2/1) to give **8** (626 mg, 85.6 %) as a white solid. LC-MS $(M+H)^+$ = 438.1.

5-(1-(azetidin-3-yl)-1H-1,2,3-triazol-4-yl)-2-(2,5-dimethoxyphenyl)pyridine (9)

To a solution of **8** (626 mg, 1.43 mmol) in DCM/TFA (5 mL/ 5 mL) was stirred at 25°C for 3h. The mixture was concentrated in vacuo to give **9** (646 mg, 100 %) as a white solid. LC-MS $(M+H)^+$ = 338.1.

Tert-5-(3-(4-(6-(2,5-dimethoxyphenyl)pyridin-3-yl)-1H-1,2,3-triazol-1-yl)azetidin-1-yl)-2-methyl-2H-indazole (X23479)

To a solution of **9** (68 mg, 0.2 mmol) in ACN (20 mL) were added **10** (64 mg, 0.3 mmol), $Pd_2(dba)_3$ (20 mg, 0.04 mmol), X-phos (20 mg, 0.04 mmol) and Cs_2CO_3 (131 mg, 0.4 mmol),

then stirred at 60°C for 16h. The mixture was concentrated in vacuo to remove organic, extracted by EA (60 mL × 3) and H_2O (50 mL). The combined organic layer was washed with brine (100 mL), then dried over with anhydrous Na_2SO_4. After filtration, the filtrate was concentrated, purified by silica gel chromatography (DCM/MeOH=20/1) to give the product **X23479** (41.7 mg, 44.3 %) as a white solid. LC-MS $(M+H)^+$ = 468.1.

^1H NMR (400 MHz, DMSO) δ 9.17 - 9.16 (m, 1H), 9.07 (s, 1H), 8.27 (dd, *J* = 8.3, 2.3 Hz, 1H), 8.07 (s, 1H), 8.01 - 7.98 (m, 1H), 7.53 (d, *J* = 9.1 Hz, 1H), 7.40 (d, *J* = 3.2 Hz, 1H), 7.11 (d, *J* = 9.0 Hz, 1H), 7.01 - 6.98 (m, 1H), 6.78 (dd, *J* = 9.1, 2.2 Hz, 1H), 6.61 (d, *J* = 1.7 Hz, 1H), 5.73 - 5.69 (m, 1H), 4.45 (t, *J* = 7.7 Hz, 2H), 4.24 - 4.20 (m, 2H), 4.10 (s, 3H), 3.81 (s, 3H), 3.77 (s, 3H).

Synthesis of X22311

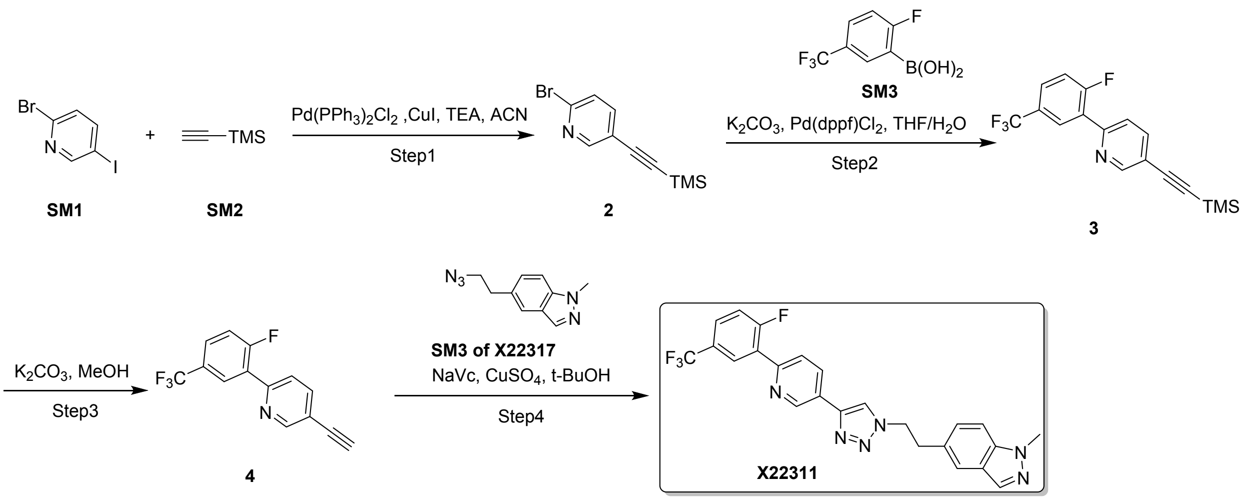

2-bromo-5-((trimethylsilyl)ethynyl)pyridine (2)

To a mixture of **SM1** (1.0 g, 3.53 mmol), **SM2** (392.0 mg, 4.0 mmol), CuI (152.0 mg, 0.8 mmol) and TEA (808.0 mg, 8.0 mmol) in anhydrous acetonitrile (20 mL) was added $Pd(PPh_3)_2Cl_2$ (247.0 mg, 0.353 mmol) and stirred at 60 degrees for 6 h under N_2 atmosphere. The mixture was filtered through a pad of celite and the filtrate was concentrated in vacuum to give the residue, which was dissolved in dichloromethane (20 mL), washed with water (10 mL) and extracted with dichloromethane (15 mL x 3). The combined organic layers were dried over Na_2SO_4,

filtered off and concentrated in vacuo to afford the residue, which was purified by flash column chromatography (petroleum ether/ethyl acetate, 1/1) to furnish **2** (600.0 mg, 67%) as a white solid. LC-MS $(M+H)^+$ =254.0

2-(2-fluoro-5-(trifluoromethyl)phenyl)-5-((trimethylsilyl)ethynyl)pyridine (3)

A mixture of **SM3** (416 mg, 2.0 mmol), **2** (506 mg, 2.0 mmol), Pd(dppf)Cl2 (140 mg, 0.2 mmol) and K2CO3 (552 mg, 4.0 mmol) in THF/H_2O (5 mL/1 mL) was stirred at 35 degrees for 12 h under N2 atmosphere. The solvent was removed to give residue, which was dissolved in dichloromethane (15 mL), diluted with water (30 mL) and extracted with dichloromethane (20 mL) for three times. The combined organic layers were dried over Na_2SO_4, filtrated and concentrated to give the residue, which was purified by combiflash to afford **3** (300 mg, 45 %) as a yellow solid. LC-MS $(M+H)^+$= 338.0

5-ethynyl-2-(2-fluoro-5-(trifluoromethyl)phenyl)pyridine (4)

A mixture of **3** (280 mg, 0.83 mmol), K_2CO_3 (458 mg, 3.32 mmol) in MeOH (6 mL) was stirred at 50 degrees for 3 h. The solvent was removed to give residue, which was dissolved in dichloromethane (20 mL), washed with water (30 mL) and extracted with dichloromethane (15 mL) for three times. The combined organic layers were dried over Na2SO4, filtrated and concentrated to give the residue, which was purified by combiflash to afford **4** (180 mg, 82 %) as a yellow solid. LC-MS $(M+H)^+$= 266.0

5-(2-(4-(6-(2-fluoro-5-(trifluoromethyl)phenyl)pyridin-3-yl)-1H-1,2,3-triazol-1-yl)ethyl)-1-methyl-1H-indazole (X22311)

A mixture of **4** (160 mg, 0.60), **SM3 of X22317** (120 mg, 0.60 mmol), NaVc (480 mg, 1.20 mmol) and CuSO4 (96 mg, 0.60 mmol) in t-BuOH (5 mL) was stirred at room temperature for 12 h. The mixture was diluted with water (40 mL) and extracted with dichloromethane (20 mL) for three times. The combined organic layers were dried over Na2SO4, filtrated and concentrated to give the residue, which was purified by Prep-TLC to afford the product **X22311** (29 mg, 10 %) as a white solid. LC-MS $(M+H)^+$= 467.0

^1H NMR (400 MHz, CDCl3) δ: 9.02 (d, J = 1.7 Hz, 1H), 8.42 – 8.35 (m, 2H), 7.96 – 7.91 (m, 2H), 7.70 (dd, J = 7.7, 3.5 Hz, 1H), 7.62 (s, 1H), 7.49 (s, 1H), 7.36 (d, J = 8.6 Hz, 1H), 7.35 -

7.28 (m, 1H), 7.16 (dd, J = 8.6, 1.5 Hz, 1H), 4.74 (t, J = 7.0 Hz, 2H), 4.08 (s, 3H), 3.41 (t, J = 7.0 Hz, 2H).

Synthesis of X21424

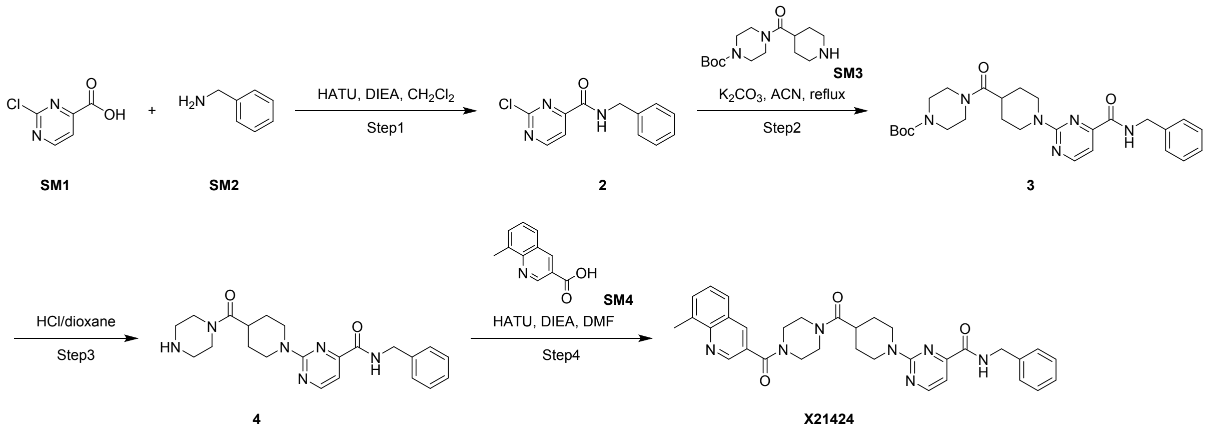

N-benzyl-2-chloropyrimidine-4-carboxamide (2)

A mixture of **SM1** (316 mg, 2.0 mmol), **SM2** (321 mg, 3.0 mmol), HATU (1.14 g, 3.0 mmol) and DIEA (516 mg, 4.0 mmol) in CH_2Cl_2 (10 mL) was stirred at room temperature for 2 h. The mixture was washed with water (20 mL) and extracted with CH_2Cl_2 (10 mL) for three times. The combined organic layers were dried over Na_2SO_4, filtrated and concentrated in vacuum to give the residue, which was purified by combiflash to afford **2** (230 mg, 47 %) as a white solid. LC-MS $(M+H)^{+}$= 248.0

tert-butyl 4-(1-(4-(benzylcarbamoyl)pyrimidin-2-yl)piperidine-4-carbonyl)piperazine-1-carboxylate (3)

A mixture of **2** (220 mg, 0.89 mmol), **SM3** (398 mg, 1.34 mmol), and K_2CO_3 (246 mg, 1.78 mmol) in ACN (13 mL) was stirred at 60 degrees for 4 h. The solvent was removed to give residue, which was dissolved in dichloromethane (10 mL), washed with water (20 mL) and extracted with dichloromethane (15 mL) for three times. The combined organic layers were dried over Na_2SO_4, filtrated and concentrated in vacuum to give the residue, which was purified by combiflash to afford **3** (270 mg, 60 %) as a white solid.LC-MS $(M+H)^{+}$= 509.2

N-benzyl-2-(4-(piperazine-1-carbonyl)piperidin-1-yl)pyrimidine-4-carboxamide (4)

To a solution of **3** (260 mg, 0.51 mmol) in dichloromethane (9 mL) was added HCl/dioxane (4M, 1 mL) and the solution was stirred at room temperature for 2 h. The reaction solution was concentrated under vacuum to give **4** (170 mg, 82 %) as a white solid. LC-MS $(M+H)^{+}$= 409.2

N-benzyl-2-(4-(4-(8-methylquinoline-3-carbonyl)piperazine-1-carbonyl)piperidin-1-yl)pyrimidine-4-carboxamide (X21424)

A mixture of **4** (160 mg, 0.39 mmol), **SM4** (110 mg, 0.59 mmol), HATU (224 mg, 0.59 mmol) and DIEA (100 mg, 0.78 mmol) in DMF (10 mL) was stirred at room temperature for 2 h. The mixture was washed with water (40 mL) and extracted with ethyl acetate (20 mL) for three times. The combined organic layers were dried over Na_2SO_4, filtrated and concentrated under vacuum to give the residue, which was purified by Prep-HPLC to afford the product **X21424** (62 mg, 28 %) as a white solid.

^{1}H NMR (400 MHz, DMSO-d_6): δ 9.33 (t, J = 6.4 Hz, 1H), 8.98 (d, J = 1.8 Hz, 1H), 8.57 (d, J = 4.8 Hz, 1H), 8.47 (s, 1H), 7.90 (d, J = 7.9 Hz, 1H), 7.72 (d, J = 7.0 Hz, 1H), 7.62 – 7.56 (m, 1H), 7.34 – 7.30 (m, 4H), 7.27 – 7.21 (m, 1H), 7.11 (d, J = 4.8 Hz, 1H), 4.84 (d, J = 0.7 Hz, 2H), 4.50 (d, J = 6.4 Hz, 2H), 3.75-3.44 (m, 8H), 3.03-2.96 (m, 3H), 2.75 (s, 3H), 1.78-1.71 (m, 2H), 1.58-1.48 (m, 2H). LC-MS $(M+H)^{+}$= 578.2

Synthesis of X22309

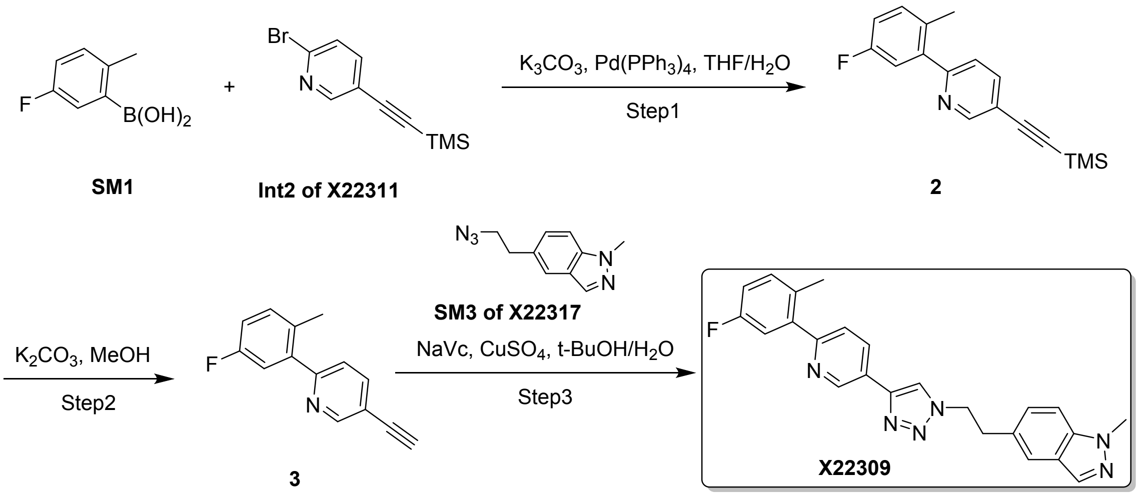

2-(5-fluoro-2-methylphenyl)-5-((trimethylsilyl)ethynyl)pyridine (2)

To a stirred mixture of **SM1** (418 mg, 2.7 mmol), **Int2 of X22311** (240 mg, 0.94 mmol) and $Pd(PPh_3)_4$ (60 mg, 0.05 mmol) in THF/H_2O (6/4.5 mL) was added K_2CO_3 (805 mg, 5.8 mmol). The reaction mixture was stirred at r.t. for 16 h under N_2 atmosphere. The reaction mixture was concentrated under vacuum to give the crude product, which was diluted with H_2O (20 mL) and extracted with EtOAc (20 mL × 3). The combined organic layer was dried over with anhydrous Na_2SO_4. After filtration, the filtrate was concentrated under vacuum to give the crude product, which was purified by flash column chromatography on silica gel to afford **2** (240 mg, 90%) as a white solid. LC-MS $(M+H)^+$ = 284.0

5-ethynyl-2-(5-fluoro-2-methylphenyl)pyridine (3)

To a stirred mixture of **2** (235 mg, 0.83 mmol) in MeOH (10 mL) was added K_2CO_3 (280 mg, 2 mmol). The reaction mixture was stirred at r.t. for 2 h. The reaction mixture was concentrated under vacuum to give the crude product, which was diluted with H_2O (20 mL) and extracted with EtOAc (20 mL × 3). The combined organic layer was dried over with anhydrous Na_2SO_4. After filtration, the filtrate was concentrated under vacuum to give the crude product, which was taken directly to afford **3** (crude, ~0.83 mmol) as a yellow oil. LC-MS $(M+H)^+$ = 212.0

5-(2-(4-(6-(5-fluoro-2-methylphenyl)pyridin-3-yl)-1H-1,2,3-triazol-1-yl)ethyl)-1-methyl-1H-indazole (X22309)

To a stirred mixture of **3** (crude, ~0.83 mmol), **SM3** of **X22317** (100 mg, 0.5 mmol) and $CuSO_4$ (90 mg, 0.36 mmol) in *t*-BuOH/H_2O (6/6 mL) was added NaVc (208 mg, 1 mmol). The reaction mixture was stirred at 60 degrees for 16 h. The reaction mixture was concentrated under vacuum to give the crude product, then the mixture was diluted with H_2O (20 mL). After filtration over a pad of diatomite (EtOAc eluent), the filtrate was separated, and the aqueous layer was extracted with EtOAc (20 mL × 3). The combined organic layer was washed with brine (30 mL), then dried over with anhydrous Na_2SO_4. After filtration, the filtrate was concentrated under vacuum to give the crude product, which was purified by prep-TLC to afford product **X22309** (50 mg,

24%) as a yellow solid. LC-MS $(M+H)^+$ = 413.1

^{1}H NMR (400 MHz, $CDCl_3$) δ 8.98 (s, 1H), 8.38 (d, *J* = 7.8 Hz, 1H), 7.91 (s, 1H), 7.63 (s, 1H), 7.54 (d, *J* = 8.3 Hz, 1H), 7.49 (s, 1H), 7.36 (d, *J* = 8.5 Hz, 1H), 7.28 (s, 1H), 7.17 (dd, *J* = 8.6, 4.2 Hz, 2H), 7.05 (td, *J* = 8.3, 2.5 Hz, 1H), 4.74 (t, *J* = 7.0 Hz, 2H), 4.08 (s, 3H), 3.41 (t, *J* = 7.0 Hz, 2H), 2.36 (s, 3H).

Synthesis of X21429

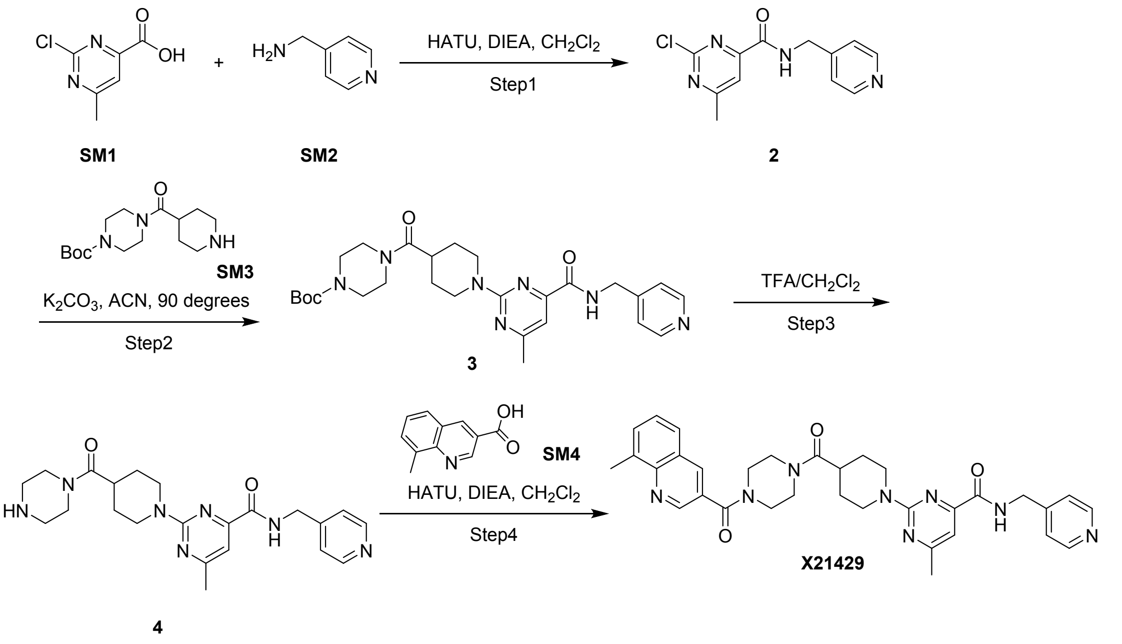

2-chloro-6-methyl-N-(pyridin-4-ylmethyl)pyrimidine-4-carboxamide (2)

To a stirred mixture of **SM1** (350 mg, 2 mmol), HATU (938 mg, 2.5 mmol) and DIEA (550 mg, 4.3 mmol) in CH_2Cl_2 (10 mL) was added **SM2** (216 mg, 2 mmol). The mixture was stirred at r.t. for 1 h. Then the reaction mixture was concentrated under vacuum to give the crude product, which was purified by flash column chromatography on silica gel to afford **2** (400 mg, 76%) as a yellow oil. LC-MS $(M+H)^+$ = 262.8

tert-butyl 4-(1-(4-methyl-6-((pyridin-4-ylmethyl)carbamoyl)pyrimidin-2-yl)piperidine-4-carbonyl)piperazine-1-carboxylate (3)

To a stirred mixture of **2** (395 mg, 1.5 mmol) and **SM3** (600 mg, 2 mmol) in CH_3CN (10 mL)

was added K_2CO_3 (600 mg, 4.3 mmol). The mixture was stirred at 90 degrees for 2 h. The reaction mixture was concentrated under vacuum to give the crude product, then diluted with H_2O (30 mL) and followed by extraction with EtOAc (20 mL × 3). The combined organic layer was dried over with anhydrous Na_2SO_4. After filtration, the filtrate was concentrated under vacuum to give the crude product, which was purified by flash column chromatography on silica gel to afford **3** (600 mg, 76%) as a yellow solid. LC-MS $(M+H)^+$ = 523.8

6-methyl-2-(4-(piperazine-1-carbonyl)piperidin-1-yl)-N-(pyridin-4-ylmethyl)pyrimidine-4-carboxamide (4)

To a stirred solution of **3** (595 mg, 1.1 mmol) in CH_2Cl_2 (10 mL) was added CF_3COOH (2 mL). The reaction solution was stirred at r.t. for 1 h, The mixture was concentrated in vacuo to give a residue, which was direct to afford **4** (crude, ~1.1 mmol) as a yellow oil. LC-MS $(M+H)^+$ = 423.7

6-methyl-2-(4-(4-(8-methylquinoline-3-carbonyl)piperazine-1-carbonyl)piperidin-1-yl)-N-(pyridin-4-ylmethyl)pyrimidine-4-carboxamide (X21429)

A solution of **4** (crude, ~0.5 mmol) in CH_2Cl_2 (10 mL) was stirred at r.t., which was added DIEA (600 mg, 4.6 mmol). Then **SM4** (200 mg, 1.1 mmol), HATU (500 mg, 1.3 mmol) was added to the mixture and the mixture was stirred at r.t. for 1 h. The reaction mixture was diluted with H_2O (20 mL) and followed by extraction with CH_2Cl_2 (20 mL × 3). The combined organic layer was washed with brine (30 mL), then dried over with anhydrous Na_2SO_4. After filtration, the filtrate was concentrated under vacuum to give the crude product, which was purified by flash column chromatography on silica gel to afford product **X21429** (50 mg, 17%) as a white solid. LC-MS $(M+H)^+$ = 592.7

^1H NMR (400 MHz, d_6-DMSO) δ 9.37 (t, J = 6.4 Hz, 1H), 8.97 (d, J = 1.6 Hz, 1H), 8.51 – 8.45 (m, 3H), 7.90 (d, J = 8.0 Hz, 1H), 7.72 (d, J = 7.2 Hz, 1H), 7.61 – 7.56 (m, 1H), 7.27 (d, J = 6.0 Hz, 2H), 7.03 (s, 1H), 4.87 (d, J = 8.8 Hz, 2H), 4.50 (d, J = 6.4 Hz, 2H), 3.76 – 3.40 (m, 8H), 2.97 (s, 3H), 2.75 (s, 3H), 2.37 (s, 3H), 1.73 (s, 2H), 1.58 – 1.47 (m, 2H).

Synthesis of X23436

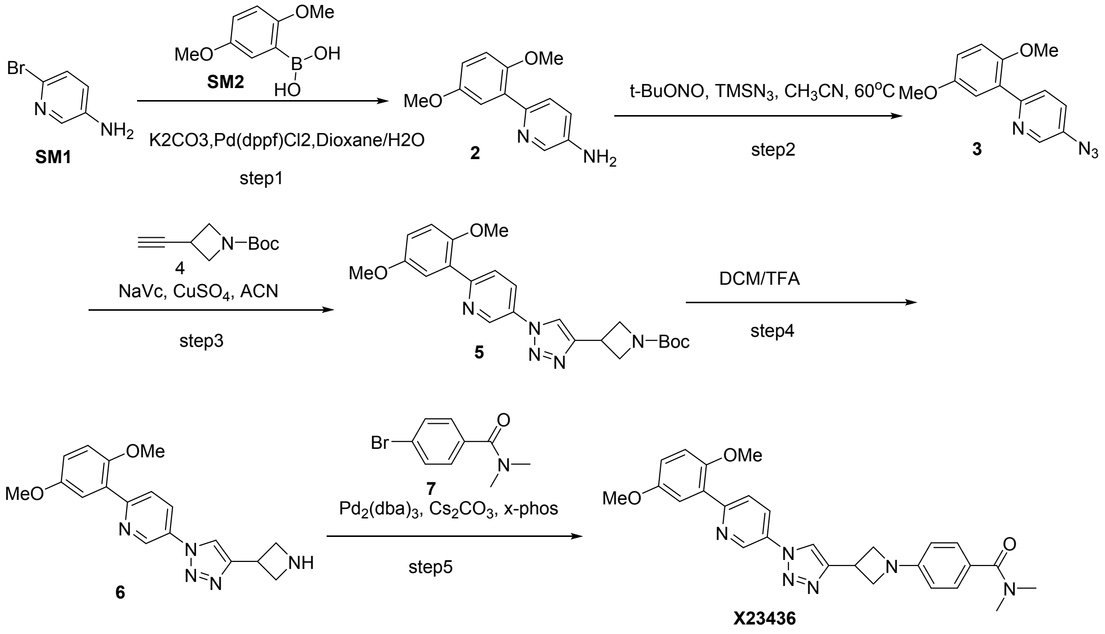

6-(2,5-dimethoxyphenyl)pyridin-3-amine (2)

To a solution of **SM1** (500 mg, 2.89 mmol) in dioxane/H_2O (4/1, 30 ml) were added **SM2** (631 mg, 3.47 mmol), K_2CO_3 (1.2 g, 8.67 mmol) and Pd(dppf)Cl_2 (234 mg, 0.29 mmol), the mixture was stirred at 90°C for 16 h. The mixture was concentrated under vacuum, the residue was diluted with H_2O (50 mL) and extracted with EtOAc (50 mL x 3). The combined organic layers were washed with brine (20 mL), dried over Na_2SO_4, filtered and concentrated in vacuo. The residue was purified by flash (DCM/MeOH=25/1) to give **2** (600 mg, 90.22%) as a yellow oil. LC-MS $(M+H)^+$ = 231.1

5-azido-2-(2,5-dimethoxyphenyl)pyridine (3)

To a solution of **2** (200 mg, 0.87 mmol) in MeCN (20 mL) were added TMSN3 (150 mg, 1.30 mmol) and t-BuONO (134 mg,1.30 mmol), the mixture was stirred at 60°C for 16 h. The mixture was concentrated under vacuum, the residue was diluted with aq. $NaHCO_3$ (10 mL) and extracted with EtOAc (50 mL x 3). The combined organic layers were washed with brine (10 mL), dried over Na_2SO_4, filtered and concentrated. The residue was purified by flash (PE/EA=7/3) to give **3** (216 mg, 64.7%) as a yellow oil. LC-MS $(M+H)^+$ = 257.1

tert-butyl 3-(1-(6-(2,5-dimethoxyphenyl)pyridin-3-yl)-1H-1,2,3-triazol-4-yl)azetidine-1-

carboxylate (5)

To a solution of **3** (216 mg, 0.84 mmol) in MeCN (20 mL) were added 4 (183 mg, 1.01 mmol), NaVc (34 mg, 0.17 mmol) and $CuSO_4$ (42 mg, 0.17 mmol), the mixture was stirred under N_2 at 80^oC for 16 h. The mixture was concentrated under vacuum, the residue was diluted with H_2O (20 mL) and extracted with EtOAc (50 mL x 3). The combined organic layers were washed with brine (10 mL), dried by Na_2SO_4, filtered and concentrated. The residue was purified by flash (PE/EA=1/1) to give **5** (321 mg, 87%) as a yellow oil. LC-MS $(M+H)^+$ = 438.2

5-(4-(azetidin-3-yl)-1H-1,2,3-triazol-1-yl)-2-(2,5-dimethoxyphenyl)pyridine (6)

To a solution of 5 (321 mg, 1.08 mmol) in DCM (20 mL) was add TFA (10 mL), the reaction mixture was stirred at rt for 2 h. The mixture was concentrated under vacuum to give **6** (300 mg, 93%) as a yellow oil. LC-MS $(M+H)^+$ = 338.2

4-(3-(1-(6-(2,5-dimethoxyphenyl)pyridin-3-yl)-1H-1,2,3-triazol-4-yl)azetidin-1-yl)-N,N-dimethylbenzamide (X23436)

To a solution of **6** (96 mg, 0.28 mmol) in MeCN (20 mL) was added **7** (97 mg, 0.43 mmol), Cs_2CO_3 (277 mg, 0.85 mmol), X-PHOS (68 mg, 0.14 mmol) and $Pd_2(dba)_3$ (41 mg, 0.071 mmol), the mixture was stirred under N_2 at 80^oC for 9 h. The mixture was concentrated under vacuum, the residue was diluted with H_2O (20 mL) and extracted with EtOAc (50 mL x 3). The combined organic layers were washed with brine (10 mL), dried over Na_2SO_4, filtered and concentrated in vacuo. The residue was purified by flash (DCM/MeOH=20/1) and prep-HPLC to give product **X23436** (51 mg, 36.99%) as white solid.

LC-MS $(M+H)^+$ = 485.2

^{1}H NMR (400 MHz, DMSO) δ 9.20 (d, *J* = 2.6 Hz, 1H), 8.96 (s, 1H), 8.34 (dd, *J* = 8.7, 2.7 Hz, 1H), 8.12 (d, *J* = 8.7 Hz, 1H), 7.40 (d, *J* = 3.2 Hz, 1H), 7.32 (d, *J*= 8.5 Hz, 2H), 7.14 (d, *J* = 9.0 Hz, 1H), 7.03 (dd, *J* = 9.0, 3.2 Hz, 1H), 6.52 (d, *J* = 8.6 Hz, 2H), 4.37 (t, *J* = 7.6 Hz, 2H), 4.26 - 4.18 (m, 1H), 4.02 (t, *J* = 6.7 Hz,2H), 3.82 (s, 3H), 3.77 (s, 3H), 2.96 (s, 6H).

Synthesis of X20377 and X20419

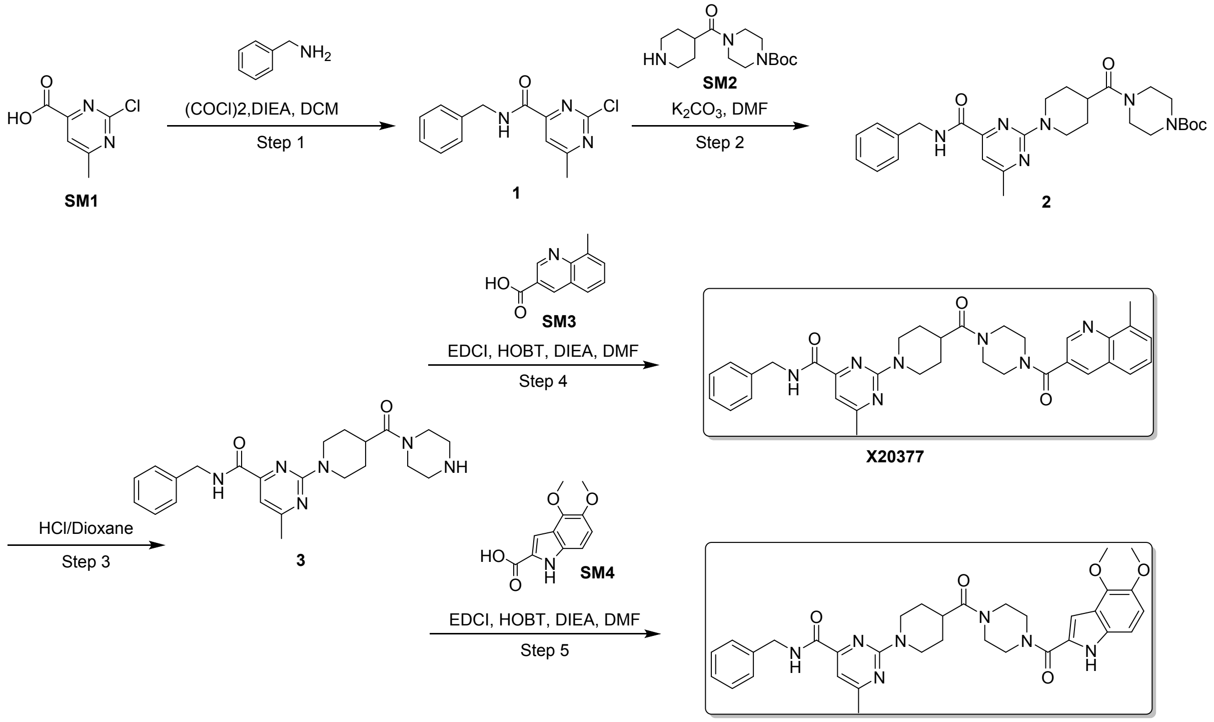

Synthesis of N-benzyl-2-chloro-6-methylpyrimidine-4-carboxamide (1)

A solution of **SM1** (344 mg, 2.0 mmol) and $(COCl)_2$ (256 mg, 2.000 mmol) in DCM (10 mL) was stirred at rt for 0.5h. The solution was concentrated and diluted with DCM (10 mL). Then DIEA(516 mg, 4.0 mmol) and benzylamine(214 mg, 2.0 mmol) was added to the above solution, the reaction mixture was stirred at rt for 2hrs. The reaction mixture was concentrated under vacuum to give crude product, the crude product was purified using Combiflash column (DCM:MeOH=100:0 to 10:1) to give **1** (400 mg, 75 %) as a white solid. LC-MS $(M+H)^+$ =262.

Synthesis of tert-butyl 4-(1-(4-(benzylcarbamoyl)-6-methylpyrimidin-2-yl)piperidine-4-carbonyl)piperazine-1-carboxylate (2)

A solution of **1** (392 mg, 1.5 mmol), **SM2** (446 mg, 1.5 mmol) and K_2CO_3 (414 mg, 3.00 mmol) in DMF (6 mL) was stirred at 80°C for 3h. The reaction mixture was concentrated under vacuum to give crude product, the crude product was purified using Combiflash column (DCM:MeOH=100:1 to 10:1) to give **2** (400 mg, 51 %) as a white solid. LC-MS $(M+H)^+$ =523.

Synthesis of N-benzyl-6-methyl-2-(4-(piperazine-1-carbonyl)piperidin-1-yl)pyrimidine-4-carboxamide (3)

A solution of **2** (400 mg, 0.767 mmol) in HCl/dioxane/DCM (5 mL/5 mL) was stirred at r.t for 1h. The reaction mixture was concentrated under vacuum to give **3** (324 mg, crude) as a yellow solid, which was directly used for next step without further purification. LC-MS $(M+H)^+$ =423.

Synthesis of N-benzyl-6-methyl-2-(4-(4-(8-methylquinoline-3-carbonyl)piperazine-1-carbonyl)piperidin-1-yl)pyrimidine-4-carboxamide (X20377)

A solution of **SM3** (72 mg, 0.384 mmol), **3** (162 mg, 0.384 mmol), EDCI (74 mg, 0.384 mmol), HOBT (52 mg, 0.384 mmol) and DIEA (74 mg, 1.152 mmol) in DMF (4 mL) was stirred at rt overnight. The reaction mixture was diluted with H_2O (30 mL), and extracted with Ethyl Acetate (30 mLx3), the combined organic layers were washed with brine (10 mLx3), then dried over with anhydrous Na_2SO_4. After filtration, the solution was concentrated under vacuum to give crude product, the crude product was purified by Prep-HPLC to give **X20377** (45 mg, 21 %) as a white solid. LC-MS $(M+H)^+$ =592.

^{1}H NMR (400 MHz, MeOD) δ 9.04 (s, 1H), 8.68 (s, 1H), 7.97 (d, *J* = 8.1 Hz, 1H), 7.83 (d, *J* = 6.9 Hz, 1H), 7.68 (t, *J* = 7.7 Hz, 1H), 7.31 (dd, *J* = 8.5, 5.4 Hz, 4H), 7.24 (td, *J* = 5.6, 2.6 Hz, 1H), 7.09 (s, 1H), 5.01 – 4.89 (m, 2H), 4.58 (s, 2H), 4.08 – 3.52 (m, 8H), 3.14 – 2.89 (m, 3H), 2.82 (s, 3H), 2.47 (s, 3H), 1.99 – 1.57 (m, 4H).

Synthesis of N-benzyl-2-(4-(4-(4,5-dimethoxy-1H-indole-2-carbonyl)piperazine-1-carbonyl)piperidin-1-yl)-6-methylpyrimidine-4-carboxamide (X20419)

A solution of **SM4** (85 mg, 0.384 mmol), **3** (162 mg, 0.384 mmol), EDCI (74 mg, 0.384 mmol), HOBT (52 mg, 0.384 mmol) and DIEA (74 mg, 1.152 mmol) in DMF (4 mL) was stirred at rt overnight. The reaction mixture was diluted with H_2O (30 mL), and extracted with Ethyl Acetate (30 mLx3), the combined organic layers were washed with brine (10 mLx3), then dried over with anhydrous Na_2SO_4. After filtration, the solution was concentrated under vacuum to give crude product, the crude product was purified by Prep-HPLC to give **X20419** (55 mg, 23 %) as a white solid. LC-MS $(M+H)^+$ =626.

^{1}H NMR (400 MHz, DMSO) δ 11.53 (s, 1H), 9.28 (t, *J* = 6.4 Hz, 1H), 7.40 – 7.18 (m, 5H), 7.12 – 6.98 (m, 3H), 6.79 (d, *J* = 1.6 Hz, 1H), 4.86 (d, *J* = 12.9 Hz, 2H), 4.49 (d, *J* = 6.5 Hz, 2H), 3.92 (s, 3H), 3.90 -

3.60 (m, 8H), 3.57 (s, 3H), 2.96 (t, J = 11.8 Hz, 3H), 2.37 (s, 3H), 1.75 (d, J = 11.2 Hz, 2H), 1.62 – 1.44 (m, 2H).

Synthesis of X22324

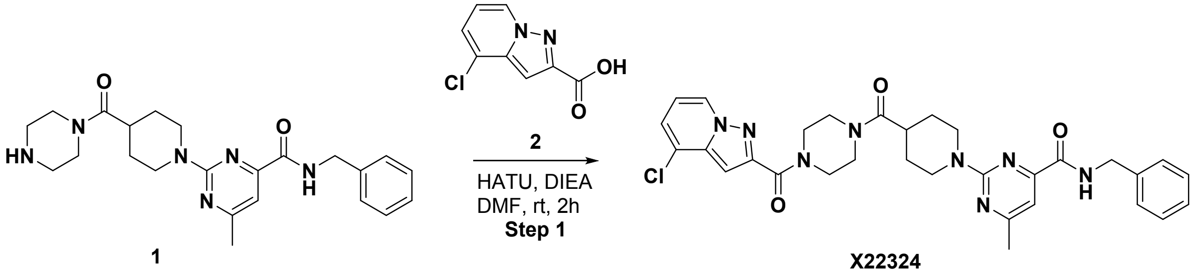

Synthesis of N-benzyl-2-(4-(4-(4-chloropyrazolo[1,5-a]pyridine-2-carbonyl)piperazine-1-carbonyl)piperidin-1-yl)-6-methylpyrimidine-4-carboxamide (X22324):

To a stirred solution of *N*-benzyl-6-methyl-2-(4-(piperazine-1-carbonyl)piperidin-1-yl)pyrimidine-4-carboxamide (**1**, 150 mg, 0.36 mmol, 1.00 equiv) and 4-chloropyrazolo[1,5-a]pyridine-2-carboxylic acid (**2**, 70 mg, 0.36 mmol, 1.00 equiv) in DMF (3 mL) were added HATU (203 mg, 0.53 mmol, 1.50 equiv) and DIEA (115 mg, 0.89 mmol, 2.50 equiv). The reaction mixture was stirred at room temperature for 2 h. The resulted solution was purified using Prep-HPLC with the following conditions (Column: XBridge Prep OBD C18 Column, 30*150 mm, 5 µm; Mobile Phase A: Water (20 mmol/L NH_4HCO_3), Mobile Phase B: ACN; Flow rate: 60 mL/min; Gradient: 35% B to 65% B in 7.00 min, 65% B; Wave Length: 220 nm; RT: 6.35 min) to afford *N*-benzyl-2-(4-(4-(4-chloropyrazolo[1,5-a]pyridine-2-carbonyl)piperazine-1-carbonyl)piperidin-1-yl)-6-methylpyrimidine-4-carboxamide (**X22324**, 28.30 mg, 13.3%) as a white solid. $[M+H]^+$ = 601.

^{1}H NMR (DMSO-d_6, 400 MHz) δ 1.48-1.56 (2H, m), 1.73 (2H, s), 2.51 (3H, s), 2.95-3.02 (3H, m), 3.52-3.88 (8H, m), 4.48 (2H, s), 4.87 (2H, s), 6.98-7.06 (3H, m), 7.22-7.34 (5H, m), 7.53 (1H, s), 8.75 (1H, s), 9.27 (1H, s).

Synthesis of X22239

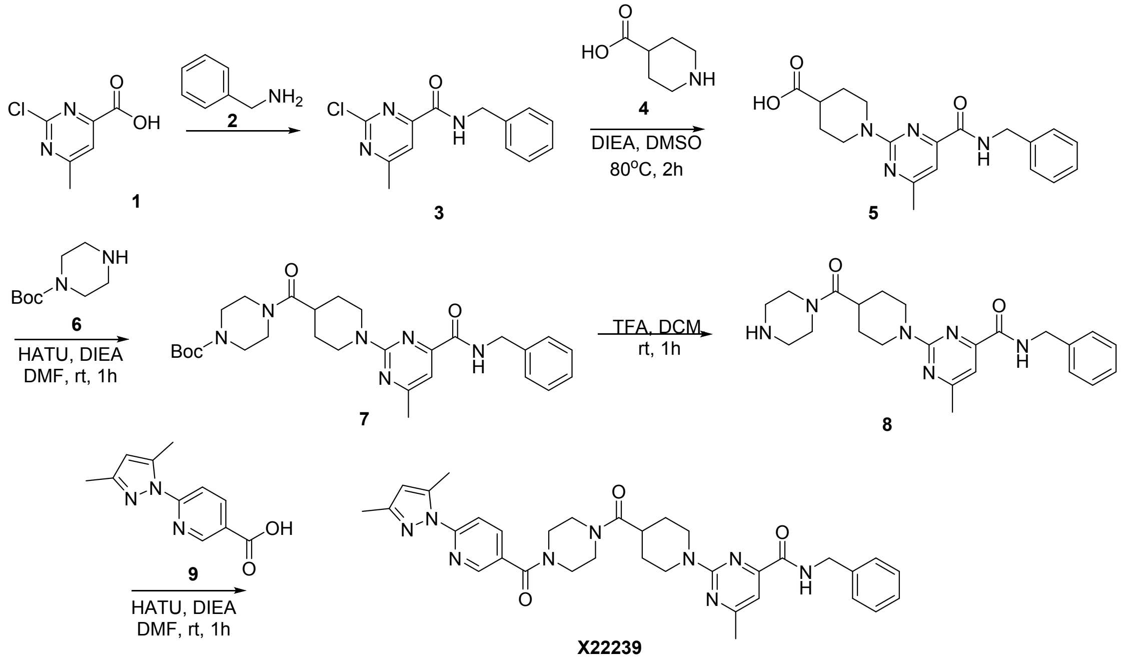

Synthesis of tert-butyl N-benzyl-2-chloro-6-methylpyrimidine-4-carboxamide (3):

To a stirred solution 2-chloro-6-methylpyrimidine-4-carboxylic acid (**1**, 500 mg, 2.89 mmol, 1.0 0 equiv) and benzylamine (**1**, 466 mg, 4.35 mmol, 1.50 equiv) in THF (5 mL) were added DMF (0.2 mL, 2.58 mmol) at 0°C for 1 h. To the above mixture was added oxalyl chloride (441 mg, 3.4 8 mmol, 1.20 equiv) at room temperature. The resulting mixture was stirred for additional 1 h at r oom temperature. The resulting mixture was concentrated under reduced pressure. The residue w as purified by reverse flash chromatography with the following conditions: column, C18 silica ge l; mobile phase, MeCN in Water, 0% to 100% gradient in 10 min; detector, UV 254 nm. This res ulted in *N*-benzyl-2-chloro-6-methylpyrimidine-4-carboxamide (**3**, 400 mg, 52.8%) as a yellow s olid. $[M+H]^+$ =262

Synthesis of 1-[4-(benzylcarbamoyl)-6-methylpyrimidin-2-yl]piperidine-4-carboxylic acid (5):

To a stirred solution N-benzyl-2-chloro-6-methylpyrimidine-4-carboxamide (**3**, 400 mg, 1.53 mmol, 1.00 equiv) and isonipecotic acid (296 mg, 2.29 mmol, 1.50 equiv) in DMSO (5 mL) were added DIEA (593 mg, 4.58 mmol, 3.00 equiv) at 80°C for 2.h. The residue was purified by reverse flash chromatography with the following conditions: column, C18 silica gel; mobile phase, MeCN in Water, 10% to 50% gradient in 10 min; detector, UV 254 nm. This resulted in 1-[4-(benzylcarbamoyl)-6-methylpyrimidin-2-yl]piperidine-4-carboxylic acid (**5**, 530 mg,

97.8%) as a yellow solid. $[M+H]^+$ =355

Synthesis of tert-butyl 4-{1-[4-(benzylcarbamoyl)-6-methylpyrimidin-2-yl]piperidine-4-carbonyl}piperazine-1-carboxylate (7):

To a stirred solution 1-[4-(benzylcarbamoyl)-6-methylpyrimidin-2-yl]piperidine-4-carboxylic acid (**5**, 520 mg, 0.90 mmol, 1.00 equiv) and tert-butyl piperazine-1-carboxylate (410 mg, 1.36 mmol, 1.50 equiv) in DMF (5 mL) were added HATU (668 mg, 1.08 mmol, 1.20 equiv) and DIEA (567 mg, 1.08 mmol, 3.00 equiv) at room temperature for 1 h. The residue was purified by reverse flash chromatography with the following conditions: column, C18 silica gel; mobile phase, MeCN in Water , 0 to 100% gradient in 10 min; detector, UV 254 nm. This resulted in tert-butyl 4-{1-[4-(benzylcarbamoyl)-6-methylpyrimidin-2-yl]piperidine-4-carbonyl}piperazine-1-carboxylate (**7**, 400.00 mg, 42.4%) as a yellow solid. $[M+H]^+$ = 523

Synthesis of N-benzyl-6-methyl-2-[4-(piperazine-1-carbonyl)piperidin-1-yl]pyrimidine-4-carboxamide (8):

A solution tert-butyl 4-{1-[4-(benzylcarbamoyl)-6-methylpyrimidin-2-yl]piperidine-4-carbonyl}piperazine-1-carboxylate (**7**, 140 mg, 0.27 mmol, 1.00 equiv) and TFA (1 mL) in DCM (3 mL) was stirred for 1 h at room temperature. The resulting mixture was concentrated under reduced pressure. This resulted in N-benzyl-6-methyl-2-[4-(piperazine-1-carbonyl)piperidin-1-yl]pyrimidine-4-carboxamide (**8**, 70 mg, 61.9%) as a yellow solid. $[M+H]^+$ = 423

Synthesis of N-benzyl-2-(4-{4-[6-(3,5-dimethylpyrazol-1-yl)pyridine-3-carbonyl]piperazine-1-carbonyl}piperidin-1-yl)-6-methylpyrimidine-4-carboxamide (X22239):

To a stirred solution *N*-benzyl-6-methyl-2-[4-(piperazine-1-carbonyl)piperidin-1-yl]pyrimidine-4-carboxamide (**8**, 60 mg, 0.14 mmol, 1.00 equiv) and 6-(3,5-dimethylpyrazol-1-yl)pyridine-3-carboxylic acid (46.3 mg, 0.21 mmol, 1.50 equiv) in DMF (2 mL) were added HATU (64.79 mg, 0.17 mmol, 1.20 equiv) and DIEA (55.1 mg, 0.47 mmol, 3.00 equiv) at room temperature for 1h. The residue was purified by reverse flash chromatography with the following conditions: column, C18 silica gel; mobile phase, MeCN in Water (0.1% FA), 0% to 100% gradient in 10

min; detector, UV 254 nm. Column: XBridge Prep OBD C18 Column, 30*150 mm, 5µm; Mobile Phase A: Water(10 mmol/L NH_4HCO_3), Mobile Phase B: ACN; Flow rate: 60 mL/min; Gradient: 34% B to 51% B in 8 min, 51% B; Wave Length: 220 nm; RT1(min): 7.70. This resulted in *N*-benzyl-2-(4-{4-[6-(3,5-dimethylpyrazol-1-yl)pyridine-3-carbonyl]piperazine-1-carbonyl}piperidin-1-yl)-6-methylpyrimidine-4-carboxamide (**X22239**, 18.3 mg, 20.7%) as a white solid. $[M+H]^+$ = 622

^{1}H NMR (DMSO-d6,400 MHz) δ 1.42–1.60 (2H, m), 1.68–1.81 (2H, m), 2.22 (3H, s), 2.37 (3H, s), 2.62 (3H s), 2.93 - 2.99 (3H, m), 3.55–3.89 (8H .m), 4.49 (2H, d), 4.85-5.01 (2H, m), 6.16 (1H, s), 7.03 (1H, s), 7.19–7.27 (1H, m), 7.32-7.41 (4H, m), 7.90 (1H, d), 8.02 (1H, d), 8.52 (1H, s), 9.27 (1H, t).

Synthesis of X22275

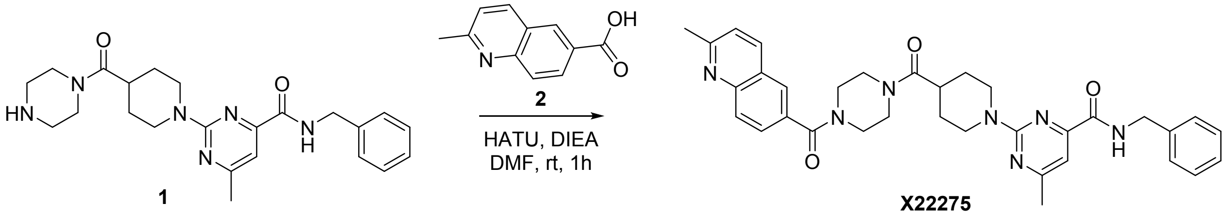

Synthesis of N-benzyl-6-methyl-2-{4-[4-(2-methylquinoline-6-carbonyl)piperazine-1-carbonyl]piperidin-1-yl}pyrimidine-4-carboxamide (X22275):

To a stirred solution N-benzyl-6-methyl-2-[4-(piperazine-1-carbonyl)piperidin-1-yl]pyrimidine-4-carboxamide (**1**, 150 mg, 0.36 mmol, 1.00 equiv) and 2-methylquinoline-6-carboxylic acid (66.5 mg, 0.36 mmol, 1.00 equiv) in DMF (2 mL) were added HATU (162 mg, 0.43 mmol, 1.20 equiv) and DIEA (229 mg, 1.78 mmol, 5.00 equiv) at room temperature for 1 h. The mixture was purified using flash chromatography with the following conditions (Mobile Phase A: Water, Mobile Phase B: ACN; Flow rate: 60 mL/min; Gradient: 0% B to 100% B in 30 min; 254/220 nm).This resulted in *N*-benzyl-6-methyl-2-{4-[4-(2-methylquinoline-6-carbonyl)piperazine-1-carbonyl]piperidin-1-yl}pyrimidine-4-carboxamide (**X22275**, 37.9 mg, 18.0%) as a white solid. $[M+H]^+$ = 592.

^{1}H NMR (DMSO-d_6, 400 MHz) δ 1.45–1.60 (2H, m), 1.73–1.81 (2H, m), 2.37 (3H, s), 2.69 (3H, s), 2.86–3.09 (3H, m), 3.55-3.81 (8H, m), 4.49 (2H, m), 4.65–4.90 (2H, m), 7.03 (1H, s), 7.15–7.27 (1H, m), 7.28–7.39 (4H, m), 7.41–7.60 (1H, m), 7.74 (1H, d), 7.98–8.01 (1H, d), 8.04 (1H,

s), 8.28–8.40 (1H, m), 9.26 (1H, t).

Synthesis of X22276

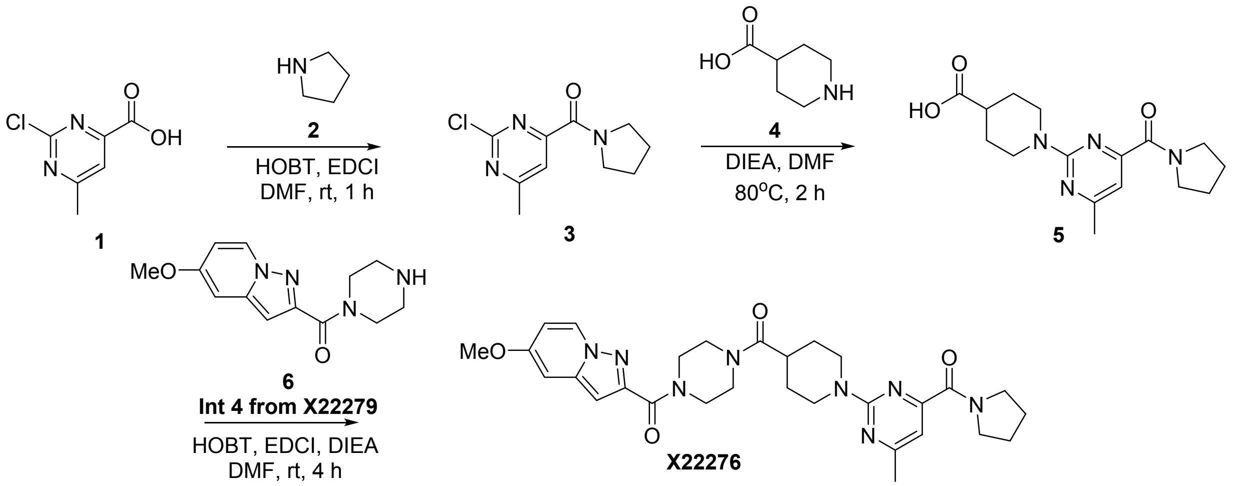

Synthesis of 2-chloro-4-methyl-6-(pyrrolidine-1-carbonyl)pyrimidine (3):

To a solution of 2-chloro-6-methylpyrimidine-4-carboxylic acid (**1**, 500 mg, 2.89 mmol, 1.00 equiv) and pyrrolidine (**2**, 309 mg, 4.34 mmol, 1.50 equiv) in DMF (5 mL) was added HOBT (470 mg, 3.47 mmol, 1.20 equiv) and EDCI (667 mg, 3.47 mmol, 1.20 equiv). The reaction mixture was stirred at room temperature for 1 h. The mixture was purified using C18 flash chromatography with the following conditions (Mobile Phase A: water. Mobile Phase B: ACN; Flow rate: 60mL/min; Grsdient: 0% B to 100% B in 30 min; 254/220 nm). This resulted in 2-chloro-4-methyl-6-(pyrrolidine-1-carbonyl)pyrimidine (**3**, 500 mg, 61.1%) as a yellow solid. $[M+H]^+ = 226$

Synthesis of 1-[4-methyl-6-(pyrrolidine-1-carbonyl)pyrimidin-2-yl]piperidine-4-carboxylic acid (5):

To a solution of 2-chloro-4-methyl-6-(pyrrolidine-1-carbonyl)pyrimidine (**3**, 500 mg, 2.21 mmol, 1.00 equiv) and isonipecotic acid (**4**, 343 mg, 2.65 mmol, 1.20 equiv) in DMF (1 mL) was added DIEA (859 mg, 6.64 mmol, 3 equiv). The reaction mixture was stirred at 80°C for 2 h. The mixture was purified using C18 flash chromatography with the following conditions (Mobile Phase A: water. Mobile Phase B: ACN; Flow rate: 60mL/min; Grsdient: 0% B to 70% B in 30 min; 254/220 nm). This resulted in 1-[4-methyl-6-(pyrrolidine-1-carbonyl)pyrimidin-2-yl]piperidine-4-carboxylic acid (**5**, 410 mg, 58.1%) as a yellow oil.

$[M+H]^+ = 319$

Synthesis of 2-[4-(4-{5-methoxypyrazolo[1,5-a]pyridine-2-carbonyl}piperazine-1-carbonyl)piperidin-1-yl]-4-methyl-6-(pyrrolidine-1-carbonyl)pyrimidine (X22276):

To a solution of 1-[4-methyl-6-(pyrrolidine-1-carbonyl)pyrimidin-2-yl]piperidine-4-carboxylic acid (**5**, 200 mg, 0.62 mmol, 1.00 equiv) and 1-{5-methoxypyrazolo[1,5-a]pyridine-2-carbonyl}piperazine (**6**, 245 mg, 0.94 mmol, 1.50 equiv) in DMF (3 mL) was added HOBT (102 mg, 0.75 mmol, 1.20 equiv), EDCI (145 mg, 0.75 mmol, 1.20 equiv) and DIEA (97 mg, 0.75 mmol, 1.20 equiv). The reaction mixture was stirred at room temperature for 3 h. The mixture was purified using Prep-HPLC with the following conditions (Column: XBridge Prep OBD C18 Column, 30*150 mm, 5µm; Mobile Phase A: water (10 mmol/L NH_4HCO_3), Mobile Phase B: ACN; Flow rate: 60 mL/min; Gradient: 15% B to 45% B in 7 min, 45% B; Wave Length: 220 nm; RT: 7.63 min). This resulted in 2-[4-(4-{5-methoxypyrazolo[1,5-a]pyridine-2-carbonyl}piperazine-1-carbonyl)piperidin-1-yl]-4-methyl-6-(pyrrolidine-1-carbonyl)pyrimidine (**X22276**, 44.3 mg, 12.6%) as a white solid. $[M+H]^+ = 561$.

^{1}H NMR (400 MHz, DMSO-d_6) δ 1.41-1.60 (2H, m), 1.69-1.78 (2H, m), 1.84 (4H, s), 2.33 (3H, s), 2.90-3.10 (3H, m), 3.41-3.49 (2H, m), 3.56 (4H, s), 3.62-3.76 (6H, m), 3.88 (3H, s), 4.67 (2H, d), 6.70 (1H, s), 6.70-6.80 (1H, m), 7.21 (1H, d), 8.21 (1H, s), 8.65 (1H, d).

Synthesis of X22277

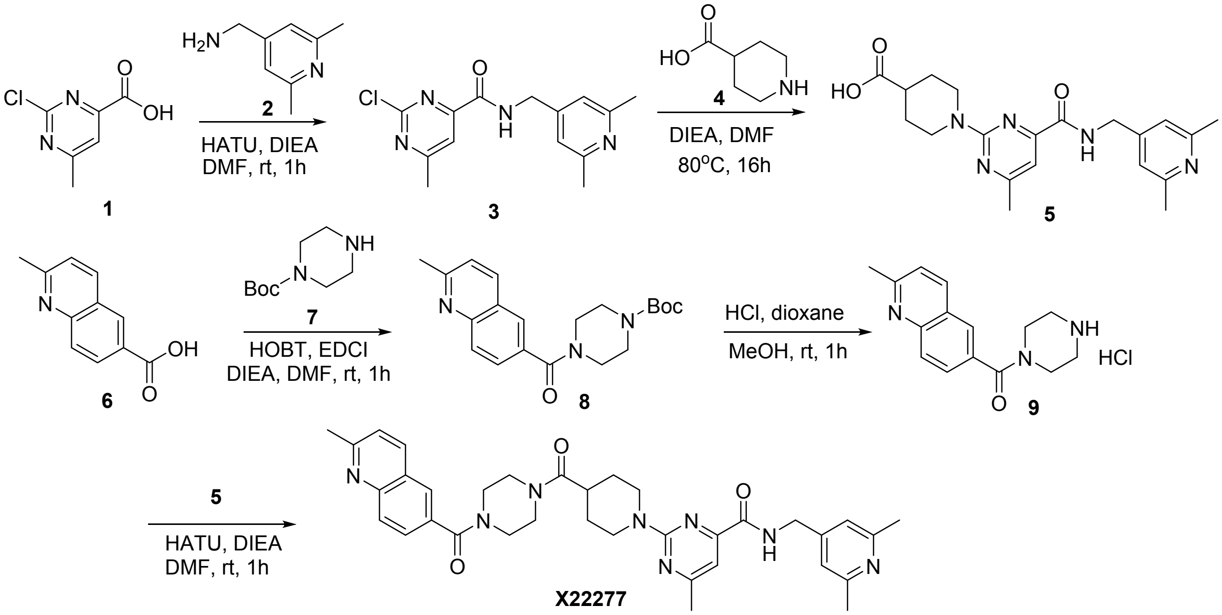

Synthesis of 2-chloro-N-[(2,6-dimethylpyridin-4-yl)methyl]-6-methylpyrimidine-4-carboxamide (3):

To a solution of 2-chloro-6-methylpyrimidine-4-carboxylic acid (**1**, 300 mg, 1.73 mmol, 1.00 equiv) in DMF (3 mL) were added 1-(2,6-dimethylpyridin-4-yl)methanamine (**2**, 355 mg, 2.60 mmol, 1.50 equiv), HATU (992 mg, 2.60 mmol, 1.50 equiv) and DIEA (674 mg, 5.21 mmol, 3 equiv). The resulting mixture was stirred at room temperature for 1 h. The resulted solution was purified using C18 flash chromatography with the following conditions (Mobile Phase A: Water, Mobile Phase B: ACN; Flow rate: 60 mL/min; Gradient: 0% B to 100% B in 30 min; 254/220 nm). This resulted in 2-chloro-*N*-[(2,6-dimethylpyridin-4-yl)methyl]-6-methylpyrimidine-4-carboxamide (**3**, 280 mg, 55.4%) as a white solid. $[M+H]^+ = 291$

Synthesis of 1-(4-{[(2,6-dimethylpyridin-4-yl)methyl]carbamoyl}-6-methylpyrimidin-2-yl)piperidine-4-carboxylic acid (5):

To a solution of 2-chloro-*N*-[(2,6-dimethylpyridin-4-yl)methyl]-6-methylpyrimidine-4-carboxamide (**3**, 270 mg, 0.92 mmol, 1.00 equiv) in DMF (3 mL) were added isonipecotic acid (**4**, 144 mg, 1.11 mmol, 1.20 equiv) and DIEA (360 mg, 2.78 mmol, 3 equiv). The resulting mixture was stirred at 80°C for 16 h. The resulted solution was purified using C18 flash chromatography with the following conditions (Mobile Phase A: Water, Mobile Phase B: ACN; Flow rate: 60 mL/min; Gradient: 0% B to 100% B in 30 min; 254/220 nm). This resulted in 1-(4-

{[(2,6-dimethylpyridin-4-yl)methyl]carbamoyl}-6-methylpyrimidin-2-yl)piperidine-4-carboxylic acid (**5**, 220.00 mg, 61.7%) as a white solid. $[M+H]^+$ = 384

Synthesis of tert-butyl 4-(2-methylquinoline-6-carbonyl)piperazine-1-carboxylate (8):

To a solution of 2-methylquinoline-6-carboxylic acid (**6**, 1.00 g, 5.34 mmol, 1.00 equiv) in DMF (10 mL) were added tert-butyl piperazine-1-carboxylate (**7**, 1.49 g, 8.01 mmol, 1.50 equiv), HOBT (1.08 g, 8.01 mmol, 1.50 equiv), EDCI (1.54 g, 8.01 mmol, 1.50 equiv) and DIEA (2.07 g, 16.02 mmol, 3.00 equiv). The resulting mixture was stirred at room temperature for 1 h. The resulted solution was purified using C18 flash chromatography with the following conditions (Mobile Phase A: Water, Mobile Phase B: ACN; Flow rate: 60 mL/min; Gradient: 0% B to 100% B in 30 min; 254/220 nm). This resulted in tert-butyl 4-(2-methylquinoline-6-carbonyl)piperazine-1-carboxylate (**8**, 2.30 g, 80.9%) as a white solid. $[M+H]^+$ = 356

Synthesis of 2-methyl-6-(piperazine-1-carbonyl)quinoline hydrochloride (9):

To a solution of tert-butyl 4-(2-methylquinoline-6-carbonyl)piperazine-1-carboxylate (**8**, 800 mg, 2.25 mmol, 1.00 equiv) in MeOH (4 mL) was added HCl (4M in 1,4-dioxane, 4 mL). The reaction mixture was stirred at room temperature for 1 h. The resulting mixture was concentrated under vacuum. This resulted in 2-methyl-6-(piperazine-1-carbonyl)quinoline hydrochloride (**9**, 800 mg, crude) as a white solid. $[M+H]^+$ = 256

Synthesis of N-[(2,6-dimethylpyridin-4-yl)methyl]-6-methyl-2-{4-[4-(2-methylquinoline-6-carbonyl)piperazine-1-carbonyl]piperidin-1-yl}pyrimidine-4-carboxamide (X22277):

To a solution of 2-methyl-6-(piperazine-1-carbonyl)quinoline hydrochloride (**9**, 210 mg, 0.82 mmol, 1.00 equiv) in DMF (2 mL) was added 1-(4-{[(2,6-dimethylpyridin-4-yl)methyl]carbamoyl}-6-methylpyrimidin-2-yl)piperidine-4-carboxylic acid (**5**, 473 mg, 1.23 mmol, 1.50 equiv), HATU (469 mg, 1.23 mmol, 1.50 equiv) and DIEA (319 mg, 2.46 mmol, 3.00 equiv). The reaction mixture was stirred at room temperature for 1 h. The resulted solution was purified using prep-HPLC with the following conditions (Column: XBridge Prep OBD C18 Column, 30*150 mm, 5μm; Mobile Phase A: Water (10 mmol/L NH_4HCO_3), Mobile Phase B:

ACN; Flow rate: 60 mL/min; Gradient: 37% B to 52% B in 8 min; Wave Length: 254 nm; RT: 3.78 min). This resulted in *N*-[(2,6-dimethylpyridin-4-yl)methyl]-6-methyl-2-{4-[4-(2-methylquinoline-6-carbonyl)piperazine-1-carbonyl]piperidin-1-yl}pyrimidine-4-carboxamide (**X22277**, 38.40 mg, 7.5%) as a white solid. $[M+H]^+ = 621$.

^1H NMR (400 MHz, CD_3OD, 20°C): δ 1.62-1.78 (2H, m), 1.79-1.92 (2H, m), 2.38-2.53 (9H, m), 2.78 (3H, s), 2.90-3.12 (3H, m), 3.49-4.10 (8H, m), 4.55 (2H, s), 4.95-5.15 (2H, m), 6.99-7.12 (3H, m), 7.49-7.59 (1H, m), 7.78-7.87 (1H, m), 8.00-8.13 (2H, m), 8.29-8.38 (1H, m).

Synthesis of X22279

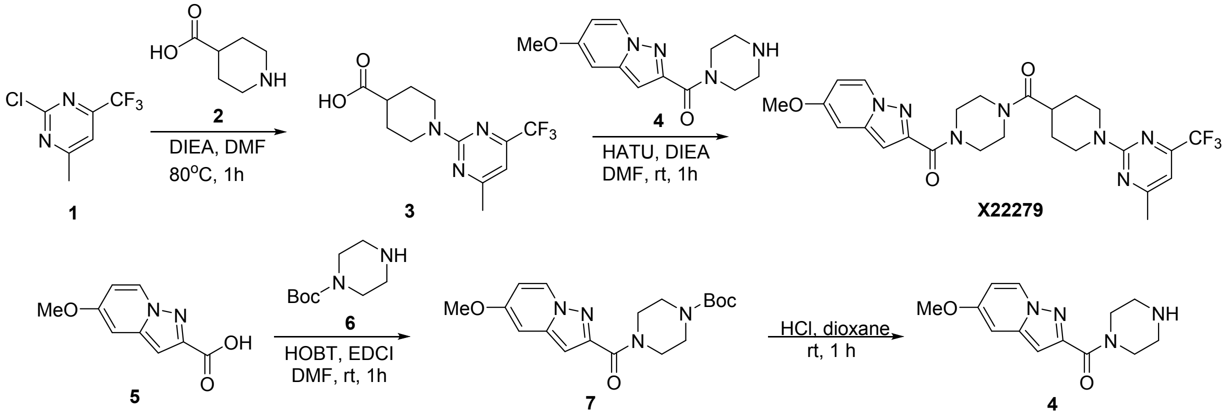

Synthesis of 1-[4-methyl-6-(trifluoromethyl)pyrimidin-2-yl]piperidine-4-carboxylic acid (3):

To a stirred solution of 2-chloro-4-methyl-6-(trifluoromethyl)pyrimidine (**1**, 300 mg, 1.52 mmol, 1.00 equiv) and isonipecotic acid (**2**, 197 mg, 1.52 mmol, 1.00 equiv) in DMF (3 mL) was added DIEA (3945 mg, 3.05 mmol, 2.00 equiv) dropwise at rt. The resulting mixture was stirred at 80°C for 1 h under nitrogen atmosphere. The mixture was allowed to cool down to rt. The residue was purified by reverse flash chromatography with the following conditions: column, C18 silica gel; mobile phase, ACN in water, 10% to 100% gradient in 20 min; detector, UV 254 nm to afford 1-[4-methyl-6-(trifluoromethyl)pyrimidin-2-yl]piperidine-4-carboxylic acid (**3**, 30 mg, 74.7%) as a white solid. $[M+H]^+ = 290$

Synthesis of tert-butyl 4-{5-methoxypyrazolo[1,5-a]pyridine-2-carbonyl}piperazine-1-carboxylate (7):

To a solution of 5-methoxypyrazolo[1,5-a]pyridine-2-carboxylic acid (**5**, 500 mg, 2.60 mmol, 1.00 equiv) and tert-butyl piperazine-1-carboxylate (**6**, 727 mg, 3.90 mmol, 1.50 equiv) in DMF

(5 mL) was added HOBT (422 mg, 3.12 mmol, 1.20 equiv) and EDCI (599 mg, 3.12 mmol, 1.20 equiv). The reaction mixture was stirred at room temperature for 1 h. The resulted solution was purified using C18 flash chromatography with the following conditions (Mobile Phase A: Water, Mobile Phase B: ACN; Flow rate: 80 mL/min; Gradient: 0% B to 100% B in 30 min; 254/220 nm). This resulted in tert-butyl 4-{5-methoxypyrazolo[1,5-a]pyridine-2-carbonyl}piperazine-1-carboxylate (**7**, 800 mg, 85.3%) as a yellow solid. $[M+H]^+$ = 361.

Synthesis of 1-{5-methoxypyrazolo[1,5-a]pyridine-2-carbonyl}piperazine (4):

A mixture of tert-butyl 4-{5-methoxypyrazolo[1,5-a]pyridine-2-carbonyl}piperazine-1-carboxylate (**7**, 1.00 g, 2.77 mmol, 1.00 equiv) in 4M HCl in 1,4-dioxane (5 mL) was stirred at room temperature for 1 h. The resulting mixture was concentrated under reduced pressure. This resulted in 1-{5-methoxypyrazolo[1,5-a]pyridine-2-carbonyl}piperazine (**4**, 1.00 g, crude) as a white solid. $[M+H]^+$ = 261.

Synthesis of 2-[4-(4-{5-methoxypyrazolo[1,5-a]pyridine-2-carbonyl}piperazine-1-carbonyl)piperidin-1-yl]-4-methyl-6-(trifluoromethyl)pyrimidine; formic acid (X22279):

To a stirred solution of 1-[4-methyl-6-(trifluoromethyl)pyrimidin-2-yl]piperidine-4-carboxylic acid (**3**, 100 mg, 0.34 mmol, 1.00 equiv) and 1-{5-methoxypyrazolo[1,5-a]pyridine-2-carbonyl}piperazine (**4**, 90 mg, 0.34 mmol, 1.00 equiv) in DMF (3 mL) were added DIEA (89 mg, 0.69 mmol, 2.00 equiv) and HATU (197 mg, 0.51 mmol, 1.50 equiv) at rt. The resulting mixture was stirred for 1h at rt under nitrogen atmosphere. The crude product was purified by Prep-HPLC with the following conditions (Column: XSelect CSH Fluoro Phenyl, 30*150 mm, 5μm; Mobile Phase A: Water(0.1%FA), Mobile Phase B: ACN; Flow rate: 60 mL/min; Gradient: 42% B to 52% B in 7 min, 52% B; Wave Length: 254; 220 nm; RT1(min): 5.98; Number Of Runs: 0) to afford 2-[4-(4-{5-methoxypyrazolo[1,5-a]pyridine-2-carbonyl}piperazine-1-carbonyl)piperidin-1-yl]-4-methyl-6-(trifluoromethyl)pyrimidine(**X22279**, formic acid, 32.8 mg, 16.2%) as a white solid. $[M+H]^+$ = 532.

^{1}H NMR (400 MHz, DMSO-d_6) δ 8.65 (d, 1H), 8.22 (s, 1H), 7.21 (d, 1H), 6.93 (s, 1H), 6.75 (dd, 1H), 4.67 (d, 2H), 3.88 (s, 3H), 3.76-3.62 (m, 6H), 3.60-3.53 (m, 2H), 3.10-2.96 (m, 3H), 2.41 (s, 3H), 1.81-1.72 (m, 2H), 1.58-1.44 (m, 2H).

Synthesis of X22327

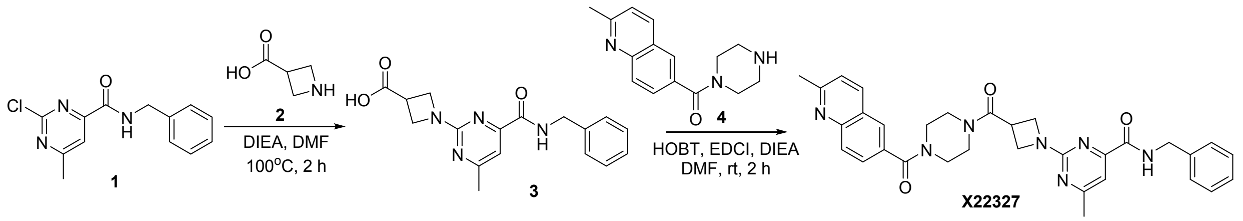

Synthesis of 1-[4-(benzylcarbamoyl)-6-methylpyrimidin-2-yl]azetidine-3-carboxylic acid (3):

To a stirred solution of N-benzyl-2-chloro-6-methylpyrimidine-4-carboxamide (**1**, 500 mg, 1.91 mmol, 1.00 equiv) and 3-azetidinecarboxylic acid (**2**, 290 mg, 2.86 mmol, 1.50 equiv) in DMF (1 mL) was added DIEA (741 mg, 5.73 mmol, 3.00 equiv). The reaction was heated at 100°C for 2 h. The mixture was purified using C18 flash chromatography with the following conditions (Mobile Phase A: water. Mobile Phase B: ACN; Flow rate: 60mL/min; Grsdient: 0% B to 60% B in 30 min; 254/220 nm). This resulted in 1-[4-(benzylcarbamoyl)-6-methylpyrimidin-2-yl]azetidine-3-carboxylic acid (**3**, 370 mg, 59.3%) as a white solid. $[M+H]^+$ = 327

Synthesis of N-benzyl-6-methyl-2-{3-[4-(2-methylquinoline-6-carbonyl)piperazine-1-carbonyl]azetidin-1-yl}pyrimidine-4-carboxamide (X22327):

To a solution of 1-[4-(benzylcarbamoyl)-6-methylpyrimidin-2-yl]azetidine-3-carboxylic acid (**3**, 200 mg, 0.61 mmol, 1.00 equiv) and 2-methyl-6-(piperazine-1-carbonyl)quinoline (**4**, 188 mg, 0.73 mmol, 1.20 equiv) in DMF (3.00 mL) was added HOBT (99 mg, 0.73 mmol, 1.20 equiv), EDCI (141 mg, 0.73 mmol, 1.20 equiv) and DIEA (238 mg, 1.83 mmol, 3.00 equiv). The reaction mixture was stirred at room temperature for 2 h. The mixture was purified using Prep-HPLC with the following conditions (Column: XBridge Shield RP18 OBD Column, 30*150 mm, 5 µm; Mobile Phase A: water (10 mmol/L NH_4HCO_3), Mobile Phase B: ACN; Flow rate: 60 mL/min; Gradient: 26% B to 46% B in 8 min, 46% B; Wave Length: 220 nm; RT: 7.68 min). This resulted in **X22327** (23.8 mg, 6.9%) as a white solid. $[M+H]^+$ = 564.

^{1}H NMR (400 MHz, DMSO-d_6, 20°C) δ 2.38 (3H, d), 2.69 (3H, s), 3.35-3.75 (8H, m), 3.86 (1H, s), 4.23 (3H, s), 4.49 (2H, d), 7.11 (1H, s), 7.20-7.27 (1H, m), 7.32 (4H, m), 7.50 (1H, d), 7.74 (1H, m), 7.98 (1H, d), 8.03 (1H, d), 8.33 (1H, d), 9.13 (1H, s).

Synthesis of X22280

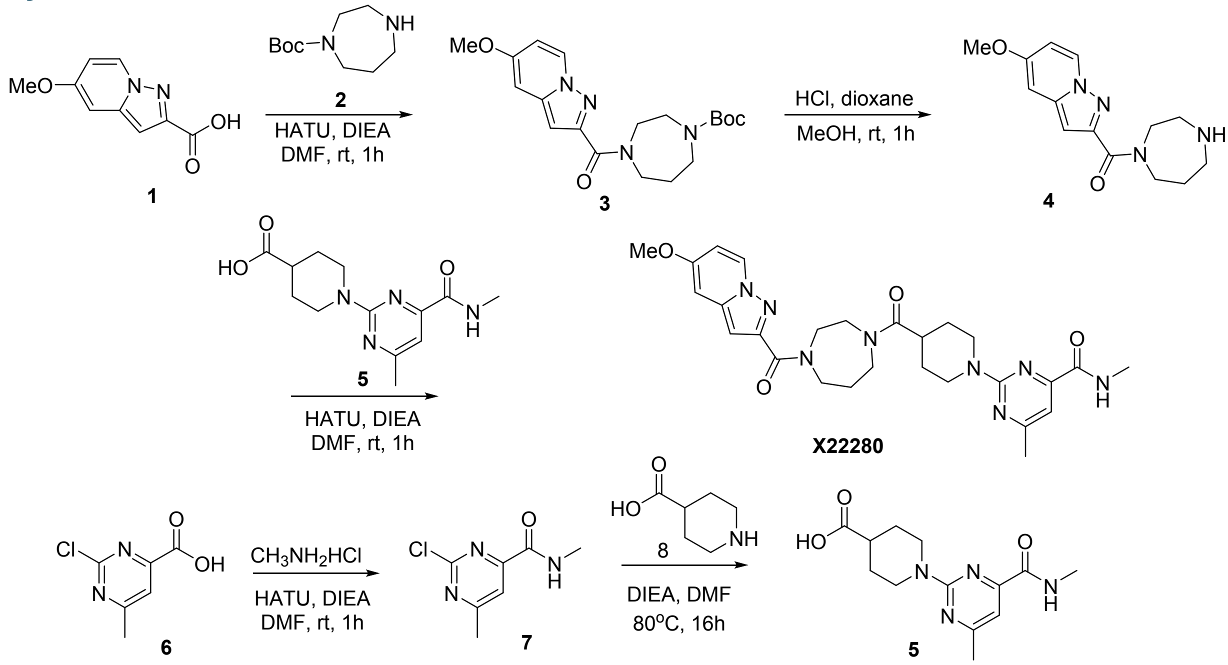

Synthesis of tert-butyl 4-{5-methoxypyrazolo[1,5-a]pyridine-2-carbonyl}-1,4-diazepane-1-carboxylate (3):

To a solution of 5-methoxypyrazolo[1,5-a]pyridine-2-carboxylic acid (**1**, 400 mg, 2.08 mmol, 1.00 equiv) in DMF (4 mL) was added tert-butyl 1,4-diazepane-1-carboxylate (**2**, 500 mg, 2.49 mmol, 1.20 equiv), HATU (1.18 g, 3.12 mmol, 1.50 equiv) and DIEA (807 mg, 6.24 mmol, 3.00 equiv). The resulting mixture was stirred at room temperature for 1 h. The resulted solution was purified using C18 flash chromatography with the following conditions (Mobile Phase A: Water, Mobile Phase B: ACN; Flow rate: 60 mL/min; Gradient: 0% B to 100% B in 30 min; 254/220 nm). This resulted in tert-butyl 4-{5-methoxypyrazolo[1,5-a]pyridine-2-carbonyl}-1,4-diazepane-1-carboxylate (**3**, 700 mg, 89.8%) as a colorless oil. $[M+H]^+$ = 375

Synthesis of 1-{5-methoxypyrazolo[1,5-a]pyridine-2-carbonyl}-1,4-diazepane hydrochloride (4):

To a solution of tert-butyl 4-{5-methoxypyrazolo[1,5-a]pyridine-2-carbonyl}-1,4-diazepane-1-carboxylate (**3**, 250 mg, 0.66 mmol, 1.00 equiv) in MeOH (2 mL) was added HCl (4M in 1,4-dioxane, 2 mL). The resulting mixture was stirred at room temperature for 1 h. The solvent was removed under vacuum. This resulted in 1-{5-methoxypyrazolo[1,5-a]pyridine-2-carbonyl}-1,4-diazepane hydrochloride (**4**, 300 mg, crude) as a white solid. $[M+H]^+$ = 275

Synthesis of 2-chloro-N,6-dimethylpyrimidine-4-carboxamide (7):

To a solution of 2-chloro-6-methylpyrimidine-4-carboxylic acid (**6**, 1.50 g, 8.69 mmol, 1.00 equiv) in DMF (15 mL) was added CH_3NH_2HCl (704.27 mg, 10.4 mmol, 1.20 equiv), HATU (4.96 g, 13.0 mmol, 1.50 equiv) and DIEA (3.37 g, 26.1 mmol, 3.00 equiv). The resulting mixture was stirred at room temperature for 1 h. The resulted solution was purified using C18 flash chromatography with the following conditions (Mobile Phase A: Water, Mobile Phase B: ACN; Flow rate: 60 mL/min; Gradient: 0% B to 100% B in 30 min; 254/220 nm). This resulted in 2-chloro-N,6-dimethylpyrimidine-4-carboxamide (**7**, 1.20 g, 74.3%) as a yellow solid. $[M+H]^+$ = 186

Synthesis of 1-[4-methyl-6-(methylcarbamoyl)pyrimidin-2-yl]piperidine-4-carboxylic acid (5):

To a solution of 2-chloro-N,6-dimethylpyrimidine-4-carboxamide (**7**, 300 mg, 1.61 mmol, 1.00 equiv) in DMF (4 mL) was added isonipecotic acid (**8**, 313 mg, 2.42 mmol, 1.50 equiv) and DIEA (627 mg, 4.84 mmol, 3.00 equiv). The resulting mixture was stirred at 80°C for 16 h. The resulted solution was purified using C18 flash chromatography with the following conditions (Mobile Phase A: Water, Mobile Phase B: ACN; Flow rate: 60 mL/min; Gradient: 0% B to 100% B in 30 min; 254/220 nm). This resulted in 1-[4-methyl-6-(methylcarbamoyl)pyrimidin-2-yl]piperidine-4-carboxylic acid (**5**, 230 mg, 51.1%) as a yellow solid. $[M+H]^+$ = 279.

Synthesis of 2-[4-(4-{5-methoxypyrazolo[1,5-a]pyridine-2-carbonyl}-1,4-diazepane-1-carbonyl)piperidin-1-yl]-N,6-dimethylpyrimidine-4-carboxamide (X22280):

To a solution of 1-{5-methoxypyrazolo[1,5-a]pyridine-2-carbonyl}-1,4-diazepane hydrochloride (**4**, 168 mg, 0.53 mmol, 1.50 equiv) in DMF (3 mL) was added 1-[4-methyl-6-(methylcarbamoyl)pyrimidin-2-yl]piperidine-4-carboxylic acid (**5**, 100 mg, 0.35 mmol, 1.00 equiv), HATU (205 mg, 0.53 mmol, 1.50 equiv) and DIEA (139 mg, 1.07 mmol, 3.00 equiv). The resulting mixture was stirred at room temperature for 1 h. The resulted solution was purified using C18 flash chromatography with the following conditions (Mobile Phase A: Water, Mobile Phase B: ACN; Flow rate: 60 mL/min; Gradient: 0% B to 100% B in 30 min; 254/220 nm). This resulted in 2-[4-(4-{5-methoxypyrazolo[1,5-a]pyridine-2-carbonyl}-1,4-diazepane-1-carbonyl)piperidin-1-yl]-N,6-dimethylpyrimidine-4-carboxamide (**X22280**, 27.9 mg, 7.8%) as a white solid. $[M+H]^+$ = 535.

^1H NMR (400 MHz, DMSO-d_6, 20°C): δ 1.32-1.50 (2H, m), 1.51-1.69 (2H, m), 1.70-1.78 (1H, m), 1.90 (1H, s), 2.34 (3H, s), 2.79 (3H, d), 2.81-3.03 (3H, m), 3.52 (1H, s), 3.62-3.75 (4H, m), 3.76-3.93 (6H, m), 4.55-5.00 (2H, m), 6.71-6.76 (1H, m), 6.98 (1H, s), 7.16-7.42 (1H, m), 8.10-8.25 (1H, m), 8.57-8.72 (2H, m).

Synthesis of X21774

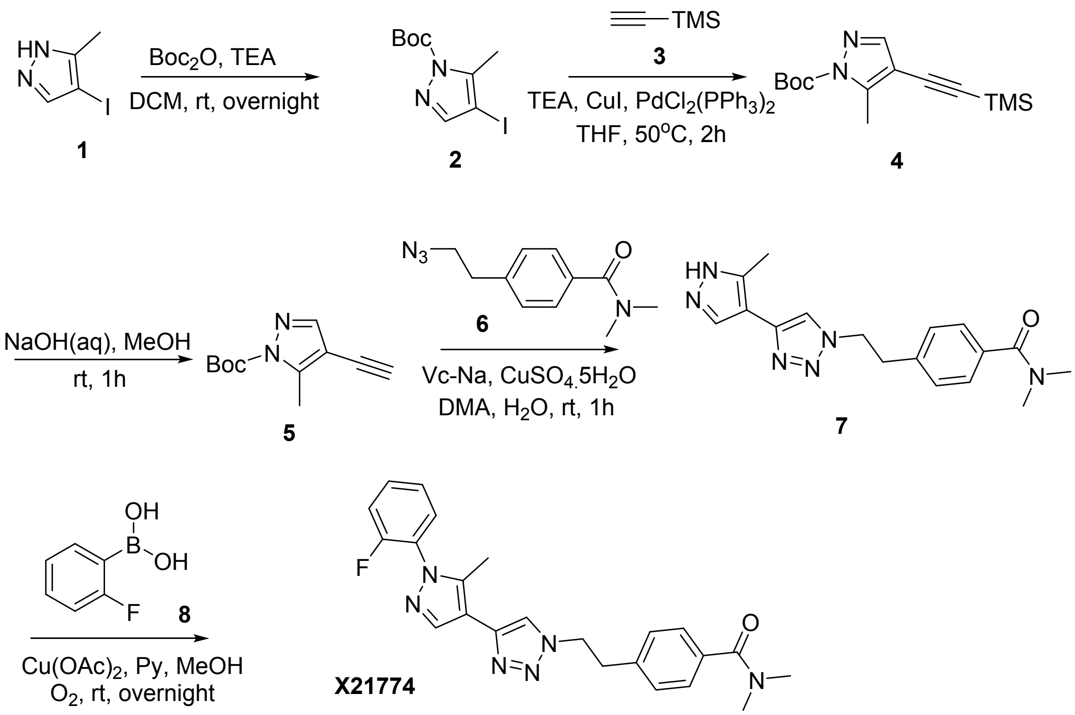

Synthesis of tert-butyl 4-iodo-5-methylpyrazole-1-carboxylate (2):

To a stirred solution of 4-iodo-3-methyl-2H-pyrazole (**1**, 500 mg, 2.40 mmol, 1.00 equiv) in DCM (5.00 mL) were added TEA (487 mg, 4.80 mmol, 2.00 equiv) and Boc_2O (630 mg, 2.89 mmol, 1.20 equiv). The mixture was stirred at room temperature overnight. The resulting mixture was concentrated under reduced pressure. The residue was purified using C18 chromatography with the following conditions (Mobile Phase A: water, Mobile Phase B: ACN; Flow rate: 60 mL/min; Gradient: 0% B to 60% B in 30 min; 254/220 nm) to afford tert-butyl 4-iodo-5-methylpyrazole-1-carboxylate (**2**, 640 mg, 86.4%) as a white solid.

$[M\text{-}Boc+H]^+ = 209$.

Synthesis of tert-butyl 5-methyl-4-[2-(trimethylsilyl)ethynyl]pyrazole-1-carboxylate (4):

To a stirred solution of tert-butyl 4-iodo-5-methylpyrazole-1-carboxylate (**2**, 500 mg, 1.62 mmol, 1.00 equiv) in THF (5 mL) were added trimethylsilylacetylene (**3**, 319 mg, 3.24 mmol, 2.00 equiv), TEA (328 mg, 3.24 mmol, 2.00 equiv), CuI (155 mg, 0.81 mmol, 0.50 equiv) and $Pd(PPh_3)_2Cl_2$ (23 mg, 0.03 mmol, 0.02 equiv) at room temperature under N_2. The resulting mixture was stirred at 50°C for 2 hours under N_2. The resulting mixture was concentrated under reduced pressure. The residue was purified by silica gel column chromatography, eluted with PE : EA =40:1. This resulted in tert-butyl 5-methyl-4-[2-(trimethylsilyl)ethynyl]pyrazole-1-carboxylate (**4**, 420.00 mg, 92.9%) as a light yellow solid.

$[M+H]^+ = 279$.

Synthesis of tert-butyl 4-ethynyl-5-methylpyrazole-1-carboxylate (5):

A solution of tert-butyl 5-methyl-4-[2-(trimethylsilyl)ethynyl]pyrazole-1-carboxylate (**4**, 400 mg, 1.43 mmol, 1.00 equiv) in NaOH (2 M, 1 mL) and MeOH (2 mL) was stirred at room temperature for 1 hour. The mixture was purified using C18 chromatography with the following conditions (Mobile Phase A: water, Mobile Phase B: ACN; Flow rate: 60 mL/min; Gradient: 0% B to 60% B in 30 min; 254/220 nm). This resulted in tert-butyl 4-ethynyl-5-methylpyrazole-1-carboxylate (**5**, 290 mg, 97.8%) as a yellow solid. $[M\text{-}Boc+H]^+ = 107$

Synthesis of N,N-dimethyl-4-(2-(4-(5-methyl-1H-pyrazol-4-yl)-1H-1,2,3-triazol-1-yl)ethyl)benzamide (7):

To a stirred solution of tert-butyl 4-ethynyl-5-methylpyrazole-1-carboxylate (**5**, 280 mg, 1.35 mmol, 1.00 equiv) and 4-(2-azidoethyl)-N,N-dimethylbenzamide (**6**, 296 mg, 1.35 mmol, 1.00 equiv) in DMA (3 mL) and H_2O (1 mL) were added $CuSO_4.5H_2O$ (169 mg, 0.67 mmol, 0.50 equiv) and sodium (2*S*)-2-[(1*S*)-1,2-dihydroxyethyl]-4-hydroxy-5-oxooxolan-3-olate (541 mg, 2.71 mmol, 2.00 equiv). The mixture was stirred at room temperature for 1 hour. The mixture was purified using C18 chromatography with the following conditions (Mobile Phase A: water, Mobile Phase B: ACN; Flow rate: 60 mL/min; Gradient: 0% B to 60% B in 30 min; 254/220

nm). This resulted in *N,N*-dimethyl-4-(2-(4-(5-methyl-1H-pyrazol-4-yl)-1H-1,2,3-triazol-1-yl)ethyl)benzamide (**7**, 180 mg, 40.8%) as a yellow solid. [M+H]+ = 325

Synthesis of 4-(2-{4-[1-(2-fluorophenyl)-5-methylpyrazol-4-yl]-1,2,3-triazol-1-yl}ethyl)-N,N-dimethylbenzamide (X21774):

To a stirred mixture of *N,N*-dimethyl-4-(2-(4-(5-methyl-1H-pyrazol-4-yl)-1H-1,2,3-triazol-1-yl)ethyl)benzamide (**7**, 100 mg, 0.31 mmol, 1.00 equiv) and 2-fluorophenylboronic acid (**8**, 216 mg, 1.54 mmol, 5.00 equiv) in MeOH (1 mL) were added $Cu(OAc)_2$ (168 mg, 0.92 mmol, 3.00 equiv) and Pyridine (4.9 mg, 0.06 mmol, 0.20 equiv) at room temperature under O_2. The resulting mixture was stirred at room temperature overnight under O_2. The mixture was purified using flash chromatography with the following conditions (Mobile Phase A: Water, Mobile Phase B: ACN; Flow rate: 60 mL/min; Gradient: 0% B to 65% B in 50 min; 254/220 nm). This resulted in 4-(2-{4-[1-(2-fluorophenyl)-5-methylpyrazol-4-yl]-1,2,3-triazol-1-yl}ethyl)-N,N-dimethylbenzamide (20.00 mg, crude). The crude product was purified using Prep-HPLC with the following conditions (Column: XBridge Shield RP18 OBD Column, 30*150 mm, 5 µm; Mobile Phase A: Water (10 mmol/L NH_4HCO_3 +0.1% $NH_3.H_2O$), Mobile Phase B: ACN; Flow rate: 60 mL/min; Gradient: 25% B to 50% B in 8 min, Wave Length: 220 nm; RT: 7.22 min) to afford 4-(2-{4-[1-(2-fluorophenyl)-5-methylpyrazol-4-yl]-1,2,3-triazol-1-yl}ethyl)-*N,N*-dimethylbenzamide (**X21774**, 9.50 mg, 7.4%) as a white solid.

$[M+H]^+$ = 419 ^{1}H NMR (Methanol-d_4, 400 MHz) δ 2.45 (3H, s), 2.97 (3H, s), 3.09 (3H, s), 3.35 (2H, d), 4.76 (2H, t), 7.29 (2H, d), 7.32 – 7.45 (5H, m), 7.77 – 7.85 (1H, m), 8.00 (1H, s), 8.33 (1H, d).

Synthesis of X21475

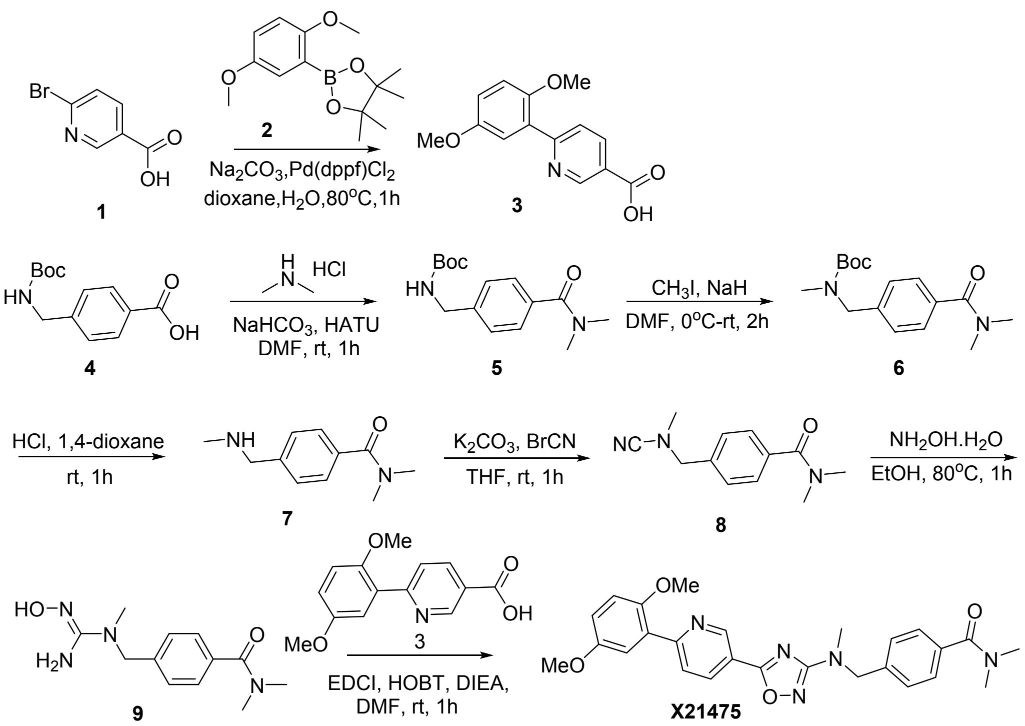

***Synthesis of 6-(2,5-dimethoxyphenyl)pyridine-3-carboxylic acid (3)*:**

To a stirred solution of 6-bromopyridine-3-carboxylic acid (**1**, 200 mg, 1.00 mmol, 1.00 equiv) and 2-(2,5-dimethoxyphenyl)-4,4,5,5-tetramethyl-1,3,2-dioxaborolane (**2**, 523 mg, 2.00 mmol, 2.00 equiv) in 1,4-dioxane (1.5 mL) and H2O (0.5 mL) were added Na_2CO_3 (315 mg, 3.00 mmol, 3.00 equiv) and $Pd(dppf)Cl_2CH_2Cl_2$ (80.7 mg, 0.10 mmol, 0.10 equiv) under nitrogen atmosphere. The resulting mixture was stirred at 80°C for 1 h under nitrogen atmosphere. The crude product was purified by reverse phase flash with the following conditions (Mobile Phase A: Water, Mobile Phase B: ACN; Flow rate: 60 mL/min; Gradient: 0% B to 100% B in 25 min; 254/220 nm) to afford 6-(2,5-dimethoxyphenyl)pyridine-3-carboxylic acid (**3**, 250 mg, 97.4%) as an orange solid. $[M+H]^+$ =260

Synthesis of tert-butyl N-{[4-(dimethylcarbamoyl)phenyl]methyl}carbamate (5):

To a stirred solution of 4-{[(tert-butoxycarbonyl)amino]methyl}benzoic acid (**4**, 1.00 g, 4.00 mmol, 1.00 equiv) and dimethylamine hydrochloride (650 mg, 8.00 mmol, 2.00 equiv) in DMF (10 mL) were added $NaHCO_3$ (1.34 g, 16.0 mmol, 4.00 equiv) and HATU (1.82 g, 4.78 mmol, 1.20 equiv). The resulting mixture was stirred at room temperature for 1 h. The crude product was purified by reverse phase flash with the following conditions (Mobile Phase A: Water,

Mobile Phase B: ACN; Flow rate: 60 mL/min; Gradient: 0% B to 100% B in 25 min; 254/220 nm) to afford tert-butyl *N*-{[4-(dimethylcarbamoyl)phenyl]methyl}carbamate (**5**, 1.00 g, 90.3%) as a white solid. $[M+H]^+$ =279

Synthesis of tert-butyl N-{[4-(dimethylcarbamoyl)phenyl]methyl}-N-methylcarbamate (6):

To a stirred solution of tert-butyl *N*-{[4-(dimethylcarbamoyl)phenyl]methyl}carbamate (**5**, 500 mg, 1.80 mmol, 1.00 equiv) in DMF (5 mL) was added NaH (144 mg, 3.60 mmol, 2.00 equiv, 60%) at 0°C. The resulting mixture was stirred at room temperature for 1 h. To the above mixture was added CH_3I (306 mg, 2.16 mmol, 1.20 equiv). The resulting mixture was stirred at room temperature for 1 h. The reaction was quenched with sat. NH_4Cl (aq.). The crude product was purified by reverse phase flash with the following conditions (Mobile Phase A: Water, Mobile Phase B: ACN; Flow rate: 60 mL/min; Gradient: 0% B to 100% B in 25 min; 254/220 nm) to afford tert-butyl N-{[4-(dimethylcarbamoyl)phenyl]methyl}-*N*-methylcarbamate (**6**, 340 mg, 64.7%) as a white solid. $[M+H]^+$ =293

Synthesis of N,N-dimethyl-4-[(methylamino)methyl]benzamide (7):

A solution of tert-butyl N-{[4-(dimethylcarbamoyl)phenyl]methyl}-N-methylcarbamate (**6**, 300.00 mg, 1.03 mmol, 1.00 equiv) in 4M HCl in 1,4-dioxane (3 mL) was stirred at room temperature for 1 h. The resulting mixture was concentrated under reduced pressure. This resulted in *N,N*-dimethyl-4-[(methylamino)methyl]benzamide (**7**, 180 mg, 91.2%) as a white solid. $[M+H]^+$ =193

Synthesis of 4-{[cyano(methyl)amino]methyl}-N,N-dimethylbenzamide (8):

To a stirred solution of *N,N*-dimethyl-4-[(methylamino)methyl]benzamide (**7**, 180 mg, 0.94 mmol, 1.00 equiv) in THF (2 mL) were added K_2CO_3 (259 mg, 1.87 mmol, 2.00 equiv) and BrCN (198 mg, 1.87 mmol, 2.0 equiv). The resulting mixture was stirred at room temperature for 1 h. The resulting mixture was concentrated under reduced pressure. The crude product was purified by reverse phase flash with the following conditions (Mobile Phase A: Water, Mobile Phase B: ACN; Flow rate: 60 mL/min; Gradient: 0% B to 100% B in 25 min; 254/220 nm) to afford 4-{[cyano(methyl)amino]methyl}-*N,N*-dimethylbenzamide (**8**, 200 mg, 98.3%) as a white

solid. $[M+H]^+$ =218

Synthesis of 4-{[(E)-N''-hydroxy-N-methylcarbamimidamido]methyl}-N,N-dimethylbenzamide (9):

A solution of 4-{[cyano(methyl)amino]methyl}-*N,N*-dimethylbenzamide (**8**, 200 mg, 0.92 mmol, 1.00 equiv) in EtOH (2 mL) and $NH_2OH.H_2O$ (1 mL) was stirred at 80°C for 1 h. The crude product was purified by reverse phase flash with the following conditions (Mobile Phase A: Water, Mobile Phase B: ACN; Flow rate: 60 mL/min; Gradient: 0% B to 100% B in 25 min; 254/220 nm) to afford 4-{[(E)-*N''*-hydroxy-*N*-methylcarbamimidamido]methyl}-*N,N*-dimethylbenzamide (**9**, 150 mg, 65.1%) as a yellow oil. $[M+H]^+$ =251

Synthesis of 4-[({5-[6-(2,5-dimethoxyphenyl)pyridin-3-yl]-1,2,4-oxadiazol-3-yl}(methyl)amino)methyl]-N,N-dimethylbenzamide (X21475):

To a stirred solution of 4-{[(E)-N''-hydroxy-N-methylcarbamimidamido]methyl}-N,N-dimethylbenzamide (**9**, 140 mg, 0.56 mmol, 1.00 equiv) and 6-(2,5-dimethoxyphenyl)pyridine-3-carboxylic acid (**3**, 145 mg, 0.56 mmol, 1.00 equiv) in DMF (2 mL) were added EDCI (214 mg, 1.12 mmol, 2.00 equiv), HOBT (151 mg, 1.12 mmol, 2.00 equiv) and DIEA (217 mg, 1.68 mmol, 3.00 equiv). The resulting mixture was stirred at room temperature for 1 h. The crude product (50 mg) was purified by Prep-HPLC with the following conditions (Column: XBridge Prep OBD C18 Column, 30*150 mm, 5 μ m; Mobile Phase A: Water(10 mmol/L NH_4HCO_3), Mobile Phase B: ACN; Flow rate: 60 mL/min; Gradient: 35% B to 65% B in 8 min; Wave Length: 220 nm; RT: 7.47 min) to afford 4-[({5-[6-(2,5-dimethoxyphenyl)pyridin-3-yl]-1,2,4-oxadiazol-3-yl}(methyl)amino)methyl]-*N,N*-dimethylbenzamide (**X21475**, 27.2 mg, 10.2%) as a white solid. $[M+H]^+$ =474.

^1H NMR (DMSO-d_6, 400 MHz) δ 2.91 - 2.97 (6H, m), 3.01 (3H, s), 3.78 (3H, s), 3.83 (3H, s), 4.70 (2H, s), 7.07 (1H, d), 7.16 (1H, d), 7.40 (4H, s), 7.35 – 7.48 (1H, m), 8.15 (1H, d), 8.42 (1H, m), 9.28 (1H, d).

Synthesis of X21498

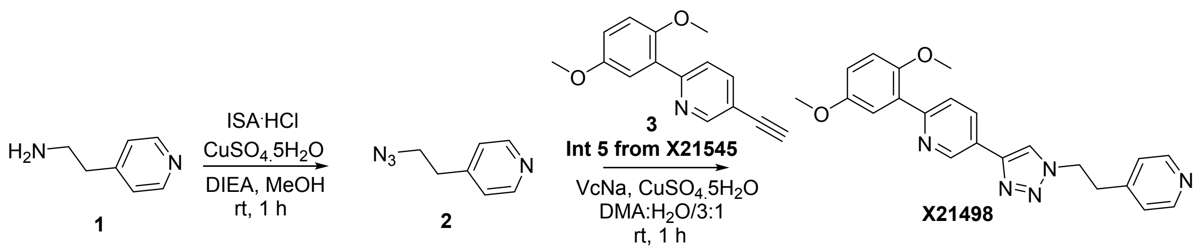

Synthesis of 4-(2-azidoethyl)pyridine (2):

To a stirred solution of 4-pyridine ethaneamine (**1**, 300 mg, 2.45 mmol, 1.00 equiv) in MeOH (6 mL) were added $CuSO_4 \cdot 5H_2O$ (307 mg, 1.22 mmol, 0.50 equiv), 1H-imidazole-1-sulfonyl azide hydrochloride (1.03 g, 4.91 mmol, 2.00 equiv) and DIEA (635 mg, 4.91 mmol, 2.00 equiv). The mixture was stirred at room temperature for 1 h. The resulting mixture was extracted with EA (3 x 10 mL). The combined organic layers were washed with brine (1 x 10 mL), dried over anhydrous Na_2SO_4. After filtration, the filtrate was concentrated under reduced pressure. This resulted in 4-(2-azidoethyl)pyridine (**2**, 308.30 mg, crude) as a yellow oil. $[M+H]^+$ = 149

Synthesis of 2-(2,5-dimethoxyphenyl)-5-{1-[2-(pyridin-4-yl)ethyl]-1,2,3-triazol-4-yl}pyridine (X21498):

To a stirred solution of 4-(2-azidoethyl)pyridine (**2**, 300 mg, 2.02 mmol, 1.00 equiv) and 2-(2,5-dimethoxyphenyl)-5-ethynylpyridine (**3**, 727 mg, 3.03 mmol, 1.50 equiv) in DMA (3 mL) and H_2O (1 mL) were added sodium (2*S*)-2-[(1*S*)-1,2-dihydroxyethyl]-4-hydroxy-5-oxooxolan-3-olate (484 mg, 2.43 mmol, 1.20 equiv) and $CuSO_4 \cdot 5H_2O$ (253 mg, 1.01 mmol, 0.50 equiv). The mixture was stirred at room temperature for 1 h. The mixture was purified using Prep-HPLC with the following conditions (Column: YMC-Actus Triart C18 ExRS, 30*150 mm, 5 µm; Mobile Phase A: water (10 mmol/L NH_4HCO_3), Mobile Phase B: ACN; Flow rate: 60 mL/min; Gradient: 55% B to 75% B in 8 min, 75% B; Wave Length: 254/220 nm; RT: 7.27 min) to afford 2-(2,5-dimethoxyphenyl)-5-{1-[2-(pyridin-4-yl)ethyl]-1,2,3-triazol-4-yl}pyridine (**X21498**, 15.40 mg, 2.0%) as a yellow solid. $[M+H]^+$ = 388

^{1}H NMR (400 MHz, DMSO-d_6, 20°C): δ 3.26 (2H, t), 3.74 (6H, d), 4.75 (2H, t), 6.95-7.01 (1H, m), 7.09 (1H, d), 7.20-7.26 (2H, m), 7.28 (1H, d), 7.93 (1H, d), 8.13-8.19 (1H, m), 8.36-8.42 (2H, m), 8.57 (1H, s), 8.99-9.04 (1H, m).

Synthesis of X21542

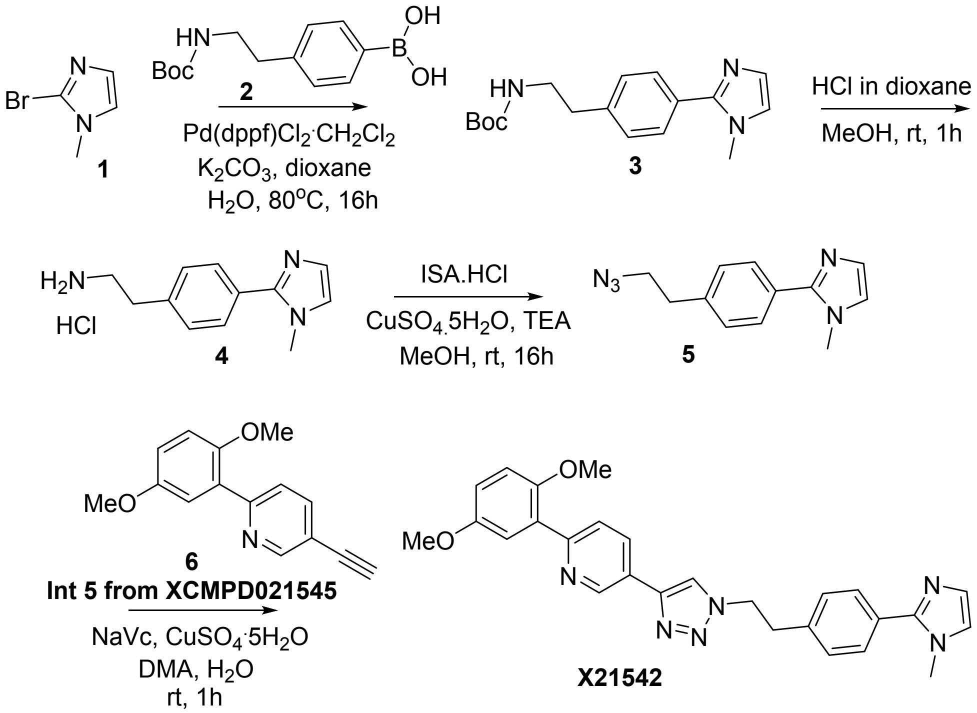

Synthesis of tert-butyl N-{2-[4-(1-methylimidazol-2-yl)phenyl]ethyl}carbamate (3):

To a solution of 2-bromo-1-methylimidazole (**1**, 200 mg, 1.24 mmol, 1.00 equiv) in dioxane (5 mL) were added 4-{2-[(tert-butoxycarbonyl)amino]ethyl}phenylboronic acid (**2**, 988 mg, 3.73 mmol, 3.00 equiv), $Pd(dppf)Cl_2 \cdot CH_2Cl_2$ (101 mg, 0.12 mmol, 0.10 equiv), K_2CO_3 (515 mg, 3.73 mmol, 3.00 equiv) and H_2O (2 mL). The reaction mixture was heated at 80°C for 16 h under nitrogen atmosphere. The solvent was removed under vacuum. The residue was purified using C18 flash chromatography with the following conditions (Mobile Phase A: Water, Mobile Phase B: ACN; Flow rate: 60 mL/min; Gradient: 0% B to 100% B in 30 min; 254/220 nm). This resulted in tert-butyl N-{2-[4-(1-methylimidazol-2-yl)phenyl]ethyl}carbamate (**3**, 150 mg, 40.1%) as a brown solid. $[M+H]^+ = 302$

Synthesis of 2-[4-(1-methylimidazol-2-yl)phenyl]ethanamine hydrochloride (4):

To a solution of tert-butyl N-{2-[4-(1-methylimidazol-2-yl)phenyl]ethyl}carbamate (**3**, 140 mg, 0.47 mmol, 1.00 equiv) in MeOH (1 mL) was added HCl (4M in dioxane, 3 mL) and the resulting solution was stirred at room temperature for 1 h. The solvent was removed under vacuum to give 2-[4-(1-methylimidazol-2-yl)phenyl]ethanamine hydrochloride (**4**, 140 mg,

crude) as a yellow solid. $[M+H]^+ = 202$

Synthesis of 2-[4-(2-azidoethyl)phenyl]-1-methylimidazole (5):

To a solution of 2-[4-(1-methylimidazol-2-yl)phenyl]ethanamine hydrochloride (**4**, 140 mg, 0.59 mmol, 1.00 equiv) in MeOH (5 mL) were added 1H-imidazole-1-sulfonyl azide hydrochloride (370 mg, 1.77 mmol, 3.00 equiv), $CuSO_4 \cdot 5H_2O$ (294 mg, 1.18 mmol, 2.00 equiv) and TEA (179 mg, 1.77 mmol, 3.00 equiv). The reaction mixture was stirred at room temperature for 16 h. The resulted solution was poured into EA and it was washed with brine, dried over Na_2SO_4. After filtration, the solvent was removed under vacuum to give 2-[4-(2-azidoethyl)phenyl]-1-methylimidazole (**5**, 120 mg, 89.7%) as a yellow oil.$[M+H]^+ = 228$

Synthesis of 2-(2,5-dimethoxyphenyl)-5-(1-{2-[4-(1-methylimidazol-2-yl)phenyl]ethyl}-1,2,3-triazol-4-yl)pyridine (X21542):

To a solution of 2-[4-(2-azidoethyl)phenyl]-1-methylimidazole (**5**, 110 mg, 0.48 mmol, 1.00 equiv) in DMA (3.00 mL) were added 2-(2,5-dimethoxyphenyl)-5-ethynylpyridine (**6**, 232 mg, 0.97 mmol, 2.00 equiv), $CuSO_4.5H_2O$ (121 mg, 0.48 mmol, 1.00 equiv), Sodium ascorbate (145 mg, 0.73 mmol, 1.50 equiv) and H_2O (2 mL). The reaction mixture was stirred at room temperature for 1 h. The resulted solution was purified using C18 flash chromatography with the following conditions (Mobile Phase A: Water, Mobile Phase B: ACN; Flow rate: 60 mL/min; Gradient: 0% B to 100% B in 30 min; 254/220 nm). The crude was purified using prep-HPLC with following conditions: Column: XBridge Prep OBD C18 Column, 30*150 mm, 5µm; Mobile Phase A: Water (10 mmol/L NH_4HCO_3), Mobile Phase B: ACN; Flow rate: 60 mL/min; Gradient: 24% B to 54% B in 8 min; Wave Length: 220 nm; RT(min): 7.47. This resulted in 2-(2,5-dimethoxyphenyl)-5-(1-{2-[4-(1-methylimidazol-2-yl)phenyl]ethyl}-1,2,3-triazol-4-yl)pyridine (**X21542**, 9.60 mg, 4.3%) as a white solid. $[M+H]^+ = 467$

^{1}H NMR (400 MHz, DMSO-d_6, 20°C): δ 3.29-3.31 (2H, m), 3.72 (3H, s), 3.77 (3H, s), 3.81 (3H, s), 4.76 (2H, t), 6.95 (1H, s), 6.96-7.01 (1H, m), 7.12 (1H, d), 7.23 (1H, d), 7.31-7.36 (2H, m), 7.40 (1H, d), 7.57-7.65 (2H, m), 7.97 (1H, d), 8.20 (1H, d), 8.70 (1H, s), 9.19 (1H, d).

Synthesis of X21543

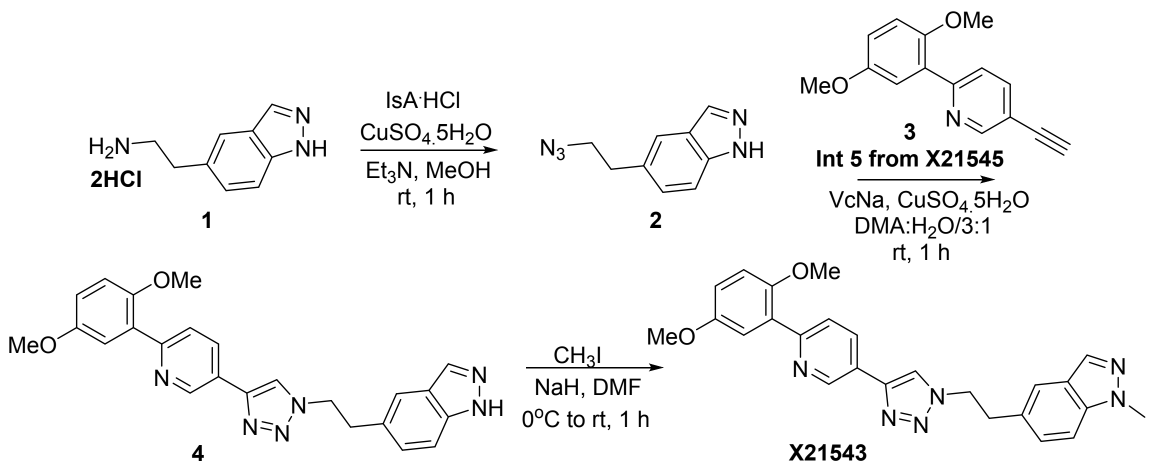

Synthesis of 5-(2-azidoethyl)-1H-indazole (2):

To a stirred solution of 2-(1H-indazol-5-yl)ethanamine dihydrochloride (**1**, 300 mg, 1.86 mmol, 1.00 equiv) in MeOH (5 mL) were added $CuSO_4 \cdot 5H_2O$ (232 mg, 0.93 mmol, 0.50 equiv), 1H-imidazole-1-sulfonyl azide hydrochloride (780 mg, 3.72 mmol, 2.00 equiv) and Et_3N (753 mg, 7.44 mmol, 4.00 equiv). The mixture was stirred at room temperature for 1 h. The resulting mixture was extracted with EA (3 x 10 mL). The combined organic layers were washed with brine (1 x 10 mL), dried over anhydrous Na_2SO_4. After filtration, the filtrate was concentrated under reduced pressure. This resulted in 5-(2-azidoethyl)-1H-indazole (**2**, 420 mg, crude) as a dark green oil. $[M+H]^+$ = 188

Synthesis of 5-(2-{4-[6-(2,5-dimethoxyphenyl)pyridin-3-yl]-1,2,3-triazol-1-yl}ethyl)-1H-indazole (4):

To a stirred solution of 5-(2-azidoethyl)-1H-indazole (**2**, 300 mg, 1.60 mmol, 1.00 equiv) and 2-(2,5-dimethoxyphenyl)-5-ethynylpyridine (**3**, 460 mg, 1.92 mmol, 1.20 equiv) in DMA (3 mL) and H_2O (1 mL) were added $CuSO_4 \cdot 5H_2O$ (200 mg, 0.80 mmol, 0.50 equiv) and sodium (2S)-2-[(1S)-1,2-dihydroxyethyl]-4-hydroxy-5-oxooxolan-3-olate (42.5 mg, 0.21 mmol, 2.00 equiv). The mixture was stirred at room temperature for 1 h. The mixture was purified using C18 chromatography with the following conditions (Mobile Phase A: water, Mobile Phase B: ACN; Flow rate: 60 mL/min; Gradient: 0% B to 60% B in 30 min; 254/220 nm) to afford 5-(2-{4-[6-(2,5-dimethoxyphenyl)pyridin-3-yl]-1,2,3-triazol-1-yl}ethyl)-1H-indazole (**4**, 195.6 mg, 28.6%) as a yellow oil. $[M+H]^+$ = 427

Synthesis of 5-(2-{4-[6-(2,5-dimethoxyphenyl)pyridin-3-yl]-1,2,3-triazol-1-yl}ethyl)-1-methylindazole (X21543):

To a stirred solution of 5-(2-{4-[6-(2,5-dimethoxyphenyl)pyridin-3-yl]-1,2,3-triazol-1-yl}ethyl)-1H-indazole (**4**, 150 mg, 0.35 mmol, 1.00 equiv) in DMF (2 mL) was added NaH (60%, 28 mg, 0.74 mmol, 2.00 equiv) at 0°C. The mixture was stirred at 0°C for 5 min. To the mixture was added CH_3I (60 mg, 0.42 mmol, 1.20 equiv) at 0°C. The mixture was stirred at room temperature for 1 h. The reaction was quenched with Water at 0°C. The residue was purified using Prep-HPLC with the following conditions (Column: XSelect CSH Prep C18 OBD Column, 19*250 mm, 5 µm; Mobile Phase A: water (0.05% TFA), Mobile Phase B: ACN; Flow rate: 30 mL/min; Gradient: 29% B to 39% B in 7 min, 39% B; Wave Length: 254; 220 nm; RT: 6.78 min) to afford 5-(2-{4-[6-(2,5-dimethoxyphenyl)pyridin-3-yl]-1,2,3-triazol-1-yl}ethyl)-1-methylindazole (**X21543**, 44.10 mg, 28.5%) as a yellow solid. $[M+H]^+ = 441$

^{1}H NMR (400 MHz, DMSO-d_6, 20°C): δ 3.35 (2H, t), 3.79 (6H, d), 4.01 (3H, s), 4.75 (2H, t), 7.01-7.06 (1H, m), 7.13 (1H, d), 7.26-7.31 (1H, m), 7.38 (1H, d), 7.54-7.59 (2H, m), 7.96 (1H, d), 8.00-8.07 (1H, m), 8.27-8.34 (1H, m), 8.72 (1H, s), 9.08-9.12 (1H, m).

Synthesis of X21544

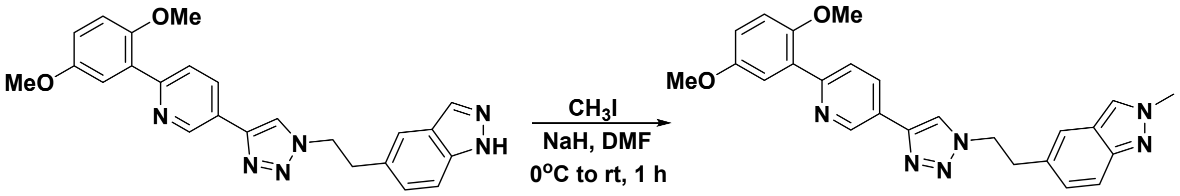

1 X21544

Synthesis of 5-(2-{4-[6-(2,5-dimethoxyphenyl)pyridin-3-yl]-1,2,3-triazol-1-yl}ethyl)-2-methylindazole (X21544):

To a stirred solution of 5-(2-{4-[6-(2,5-dimethoxyphenyl)pyridin-3-yl]-1,2,3-triazol-1-yl}ethyl)-1H-indazole (**1**, 150 mg, 0.35 mmol, 1.00 equiv) in DMF (2 mL) was added NaH (60%, 28 mg, 0.70 mmol, 2.00 equiv) at 0°C. The mixture was stirred at 0°C for 5 min. To the mixture was added CH_3I (60 mg, 0.42 mmol, 1.20 equiv) at 0°C. The mixture was stirred at room temperature

for 1 h. The reaction was quenched with Water at 0°C. The residue was purified using Prep-HPLC with the following conditions (Column: XSelect CSH Prep C18 OBD Column, 19*250 mm, 5 µm; Mobile Phase A: water (0.05% TFA), Mobile Phase B: ACN; Flow rate: 30 mL/min; Gradient: 29% B to 39% B in 7 min, 39% B; Wave Length: 254; 220 nm; RT: 5.33 min) to afford 5-(2-{4-[6-(2,5-dimethoxyphenyl)pyridin-3-yl]-1,2,3-triazol-1-yl}ethyl)-2-methylindazole (**X21544**, 42.2 mg, 27.2%) as a yellow solid. $[M+H]^+$ = 441

^{1}H NMR (400 MHz, DMSO-d_6, 20°C): δ 3.29 (2H, t), 3.80 (6H, d), 4.12 (3H, s), 4.75 (2H, t), 7.03-7.09 (1H, m), 7.12-7.18 (2H, m), 7.38 (1H, d), 7.47 (1H, s), 7.53 (1H, d), 8.07 (1H, d), 8.23 (1H, s), 8.33-8.40 (1H, m), 8.73 (1H, s), 9.12 (1H, s).

Synthesis of X21499

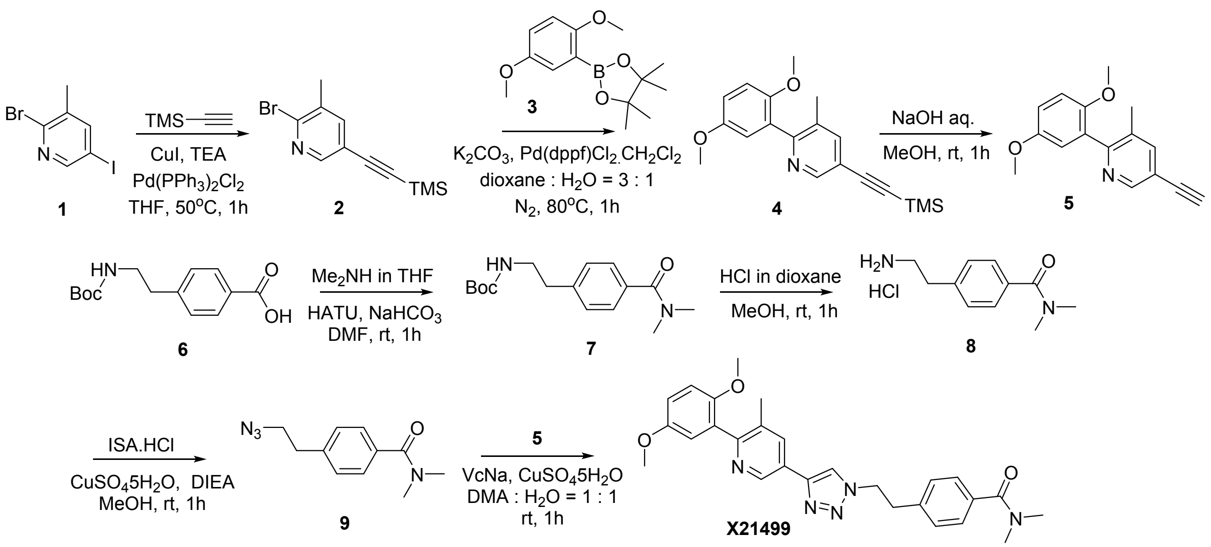

Synthesis of 2-bromo-3-methyl-5-[2-(trimethylsilyl)ethynyl]pyridine (2):

To a solution of 2-bromo-5-iodo-3-methylpyridine (**1**, 1.00 g, 3.36 mmol, 1.00 equiv) in THF (5 mL) was added CuI (320 mg, 1.68 mmol, 0.50 equiv), trimethylsilylacetylene (396 mg, 4.03

mmol, 1.20 equiv), TEA (680 mg, 6.71 mmol, 2.00 equiv) and $Pd(PPh_3)_2Cl_2$ (471 mg, 0.67 mmol, 0.20 equiv). The reaction mixture was heated at 50°C for 1 h under nitrogen atmosphere. After evaporation, the residue was purified using C18 flash chromatography with the following conditions (Mobile Phase A: Water, Mobile Phase B: ACN; Flow rate: 80 mL/min; Gradient: 0% B to 100% B in 30 min; 254/220 nm). This resulted in 2-bromo-3-methyl-5-[2-(trimethylsilyl)ethynyl]pyridine (**2**, 460 mg, 51.1%) as a brown oil. $[M+H]^+$ = 268

Synthesis of 2-(2,5-dimethoxyphenyl)-3-methyl-5-[2-(trimethylsilyl)ethynyl]pyridine (4):
To a solution of 2-bromo-3-methyl-5-[2-(trimethylsilyl)ethynyl]pyridine (**2**, 430 mg, 1.60 mmol, 1.00 equiv) in dioxane (4 mL) was added 2-(2,5-dimethoxyphenyl)-4,4,5,5-tetramethyl-1,3,2-dioxaborolane (**3**, 550 mg, 2.08 mmol, 1.30 equiv), K_2CO_3 (886 mg, 6.41 mmol, 4.00 equiv), $Pd(dppf)Cl_2CH_2Cl_2$ (131 mg, 0.16 mmol, 0.10 equiv) and H_2O (1 mL). The reaction mixture was heated at 80°C for 1 h under nitrogen atmosphere. After evaporation, the residue was purified using C18 flash chromatography with the following conditions (Mobile Phase A: Water, Mobile Phase B: ACN; Flow rate: 80 mL/min; Gradient: 0% B to 100% B in 30 min; 254/220 nm). This resulted in 2-(2,5-dimethoxyphenyl)-3-methyl-5-[2-(trimethylsilyl)ethynyl]pyridine (**4**, 300 mg, 57.5%) as a colorless oil. $[M+H]^+$ = 326

Synthesis of 2-(2,5-dimethoxyphenyl)-5-ethynyl-3-methylpyridine (5):
To a solution of 2-(2,5-dimethoxyphenyl)-3-methyl-5-[2-(trimethylsilyl)ethynyl]pyridine (**4**, 240 mg, 0.74 mmol, 1.00 equiv) in MeOH (3 mL) was added NaOH aq. (40 mg, 1.00 mmol, 1.35 equiv, in 1 mL of H_2O). The reaction mixture was stirred at room temperature for 1 h. The resulting mixture was extracted with EA. The combined organic layers were washed with brine, dried over anhydrous Na_2SO_4. After filtration, the filtrate was concentrated under reduced pressure. This resulted in 2-(2,5-dimethoxyphenyl)-5-ethynyl-3-methylpyridine (**5**, 150 mg, 80.3%) as a brown solid. $[M+H]^+$ = 254

Synthesis of tert-butyl N-{2-[4-(dimethylcarbamoyl)phenyl]ethyl}carbamate (7):
To a solution of tris(4-{2-[(tert-butoxycarbonyl)amino]ethyl}benzoic acid) (**6**, 400 mg, 1.51 mmol, 1.00 equiv) in DMF (3 mL) was added dimethylamine (2M in THF, 1.51 mL, 3.02 mmol, 2.00 equiv), HATU (861 mg, 2.26 mmol, 1.50 equiv) and $NaHCO_3$ (634 mg).The reaction

mixture was stirred at room temperature for 1 h. The resulted solution was purified using C18 flash chromatography with the following conditions (Mobile Phase A: Water, Mobile Phase B: ACN; Flow rate: 80 mL/min; 254/220 nm). This resulted in tert-butyl *N*-{2-[4-(dimethylcarbamoyl)phenyl]ethyl}carbamate (**7**, 395 mg, 89.5%) as a white solid. $[M+H-56]^+$ = 237

Synthesis of 4-(2-aminoethyl)-N,N-dimethylbenzamide hydrochloride (8):

To a solution of tert-butyl *N*-{2-[4-(dimethylcarbamoyl)phenyl]ethyl}carbamate (**7**, 370 mg, 8.21 mmol, 1.00 equiv) in MeOH (5 mL) was added HCl (4M in 1,4-dioxane, 5 mL). The reaction mixture was stirred at room temperature for 1 h. The resulting mixture was concentrated under reduced pressure. This resulted in 4-(2-aminoethyl)-N,N-dimethylbenzamide hydrochloride (**8**, 400 mg, crude) as a brown oil. $[M+H]^+$ = 193

Synthesis of 4-(2-azidoethyl)-N,N-dimethylbenzamide (9):

To a solution of 4-(2-aminoethyl)-N,N-dimethylbenzamide hydrochloride (**8**, 200 mg, 1.04 mmol, 1.00 equiv) in MeOH (3 mL) was added DIEA (269 mg, 2.08 mmol, 2.00 equiv), $CuSO_4.5H_2O$ (130 mg, 0.52 mmol, 0.50 equiv) and 1H-imidazole-1-sulfonyl azide hydrochloride (435 mg, 2.08 mmol, 2.00 equiv). The reaction mixture was stirred at room temperature for 1 h. The resulting mixture was extracted with EtOAc. The combined organic layers were washed with brine, dried over anhydrous Na_2SO_4. After filtration, the filtrate was concentrated under reduced pressure. This resulted in 4-(2-azidoethyl)-*N,N*-dimethylbenzamide (**9**, 140 mg, 61.7%) as a white solid. $[M+H]^+$ = 219

Synthesis of 4-(2-{4-[6-(2,5-dimethoxyphenyl)-5-methylpyridin-3-yl]-1,2,3-triazol-1-yl}ethyl)-N,N-dimethylbenzamide (X21499):

To a solution of 4-(2-azidoethyl)-*N,N*-dimethylbenzamide (**9**, 112 mg, 0.51 mmol, 1.30 equiv) in DMA (1 mL) was added 2-(2,5-dimethoxyphenyl)-5-ethynyl-3-methylpyridine (**5**, 100 mg, 0.39 mmol, 1.00 equiv), $CuSO_4.5H_2O$ (296 mg, 1.18 mmol, 3.00 equiv), sodium ascorbate (236 mg, 1.18 mmol, 3.00 equiv) and H_2O (1 mL). The reaction mixture was stirred at room temperature for 1 h. The resulting mixture was concentrated under vacuum. The residue was purified using prep-HPLC with following conditions: Column: XBridge Prep OBD C18 Column, 30*150 mm,

5µm; Mobile Phase A: Water (10 mmol/L NH_4HCO_3), Mobile Phase B: ACN; Flow rate: 60 mL/min; Gradient: 23% B to 53% B in 8 min, 53% B; Wave Length: 220 nm; RT(min): 7.27. This resulted in 4-(2-{4-[6-(2,5-dimethoxyphenyl)-5-methylpyridin-3-yl]-1,2,3-triazol-1-yl}ethyl)-*N,N*-dimethylbenzamide (**X21499,** 21.6 mg, 11.6%) as a white solid. $[M+H]^+$ = 472
^1H NMR (400 MHz, DMSO-d_6, 20°C): δ 2.14 (3H, s), 2.88 (6H, d), 3.20-3.30 (2H, m), 3.70 (6H, d), 4.65-4.80 (2H, m), 6.78 (1H, s), 6.91-7.01 (1H, m), 7.08 (1H, d), 7.24-7.46 (4H, m), 8.04 (1H, s), 8.63 (1H, s), 8.83 (1H, s).

Synthesis of X21545

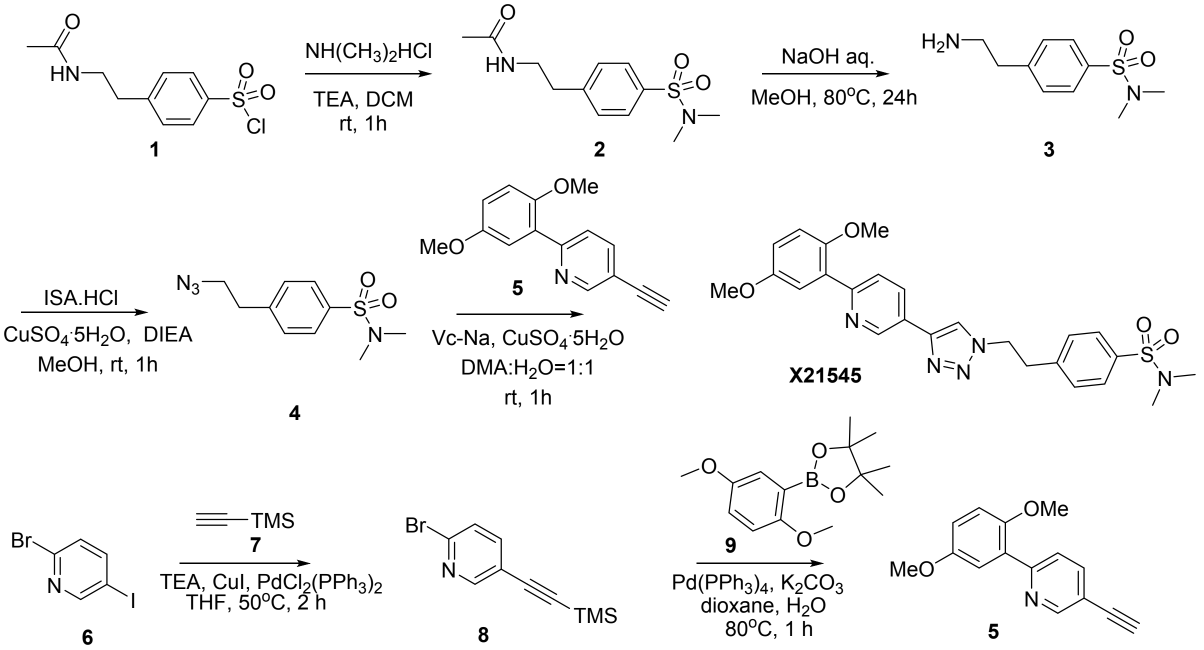

Synthesis of N-{2-[4-(dimethylsulfamoyl)phenyl]ethyl}acetamide (2):

To a solution of 4-(2-acetamidoethyl)benzenesulfonyl chloride (**1**, 300 mg, 1.14 mmol, 1.00 equiv) in DCM (5 mL) was added dimethylamine hydrochloride (140 mg, 1.71 mmol, 1.50 equiv) and TEA (348 mg, 3.43 mmol, 3.00 equiv). The resulting mixture was stirred at room temperature for 1 h. The solvent was removed under vacuum. The residue was purified using C18 flash chromatography with the following conditions (Mobile Phase A: Water, Mobile Phase B: ACN; Flow rate: 60 mL/min; Gradient: 0% B to 100% B in 30 min; 254/220 nm). This resulted in *N*-{2-[4-(dimethylsulfamoyl)phenyl]ethyl}acetamide (**2**, 200 mg, 64.5%) as a white solid. $[M+H]^+$ = 271

Synthesis of 4-(2-aminoethyl)-N,N-dimethylbenzenesulfonamide (3):

To a solution of *N*-{2-[4-(dimethylsulfamoyl)phenyl]ethyl}acetamide (**2**, 190 mg, 0.70 mmol, 1.00 equiv) in MeOH (2 mL) was added aq. NaOH (2M, 2 mL). The resulting mixture was stirred at 80°C for 24 h. The mixture was acidified to pH 7. The resulting solution was purified using C18 flash chromatography with the following conditions (Mobile Phase A: Water, Mobile Phase B: ACN; Flow rate: 60 mL/min; Gradient: 0% B to 100% B in 30 min; 254/220 nm). This resulted in 4-(2-aminoethyl)-*N,N*-dimethylbenzenesulfonamide (**3**, 110 mg, 68.5%) as a white solid. $[M+H]^+$ = 229

Synthesis of 4-(2-azidoethyl)-N,N-dimethylbenzenesulfonamide (4):

To a solution of 4-(2-aminoethyl)-*N,N*-dimethylbenzenesulfonamide (**3**, 100 mg, 0.43 mmol, 1.00 equiv) in MeOH (4 mL) was added $CuSO_4 \cdot 5H_2O$ (54.7 mg, 0.21 mmol, 0.50 equiv), ISA·HCl (183 mg, 0.87 mmol, 2.00 equiv) and DIEA (170 mg, 1.31 mmol, 3.00 equiv). The reaction mixture was stirred at room temperature for 1 h. The resulting mixture was diluted with EtOAc (30 mL) and it was washed with water (3x10 mL). The organic layer was dried over anhydrous Na_2SO_4. After filtration, the filtrate was concentrated under reduced pressure. This resulted in 4-(2-azidoethyl)-*N,N*-dimethylbenzenesulfonamide (**4**, 110 mg, crude) as a yellow oil. $[M+H]^+$ = 255

Synthesis of 2-bromo-5-[2-(trimethylsilyl)ethynyl]pyridine (8):

To a stirred solution of 2-bromo-5-iodopyridine (**6**, 10.0 g, 35.2 mmol, 1.00 equiv) in THF (30 mL) was added trimethylsilylacetylene (**7**, 4.15 g, 42.3 mmol, 1.20 equiv), TEA (7.13 g, 70.4 mmol, 2.00 equiv), CuI (3.35 g, 17.6 mmol, 0.50 equiv) and $PdCl_2(PPh_3)_2$ (1.98 g, 2.81 mmol, 0.08 equiv). The resulting mixture was stirred at 50°C for 2 h under nitrogen atmosphere. The resulting mixture was concentrated under reduced pressure. The residue was purified using silica gel column chromatography, eluted with PE / EA (40:1) to afford 2-bromo-5-[2-(trimethylsilyl)ethynyl]pyridine (**8**, 4.60 g, 51.2%) as a light yellow solid.

Synthesis of 2-(2,5-dimethoxyphenyl)-5-ethynylpyridine (5):

To a stirred solution of 2-(2,5-dimethoxyphenyl)-4,4,5,5-tetramethyl-1,3,2-dioxaborolane (**9**, 1.20 g, 4.54 mmol, 1.00 equiv) and 2-bromo-5-[2-(trimethylsilyl)ethynyl]pyridine (**8**, 1.50 g, 5.90 mmol, 1.30 equiv) in dioxane (3.00 mL)/H_2O (1.00 mL) were added $Pd(PPh_3)_4$ (370 mg, 0.31 mmol, 0.07 equiv) and K_2CO_3 (2.51 g, 18.2 mmol, 4.00 equiv). The resulting mixture was stirred at 80°C for 1 h under nitrogen atmosphere. The resulting mixture was concentrated under reduced pressure. The residue was purified using C18 flash chromatography with the following conditions (Mobile Phase A: Water, Mobile Phase B: ACN; Flow rate: 60 mL/min; Gradient: 0% B to 100% B in 30 min; 254/220 nm) to afford 2-(2,5-dimethoxyphenyl)-5-ethynylpyridine (**5**, 860 mg, 77.2%) as a brown oil. $[M+H]^+$ = 240

Synthesis of 4-(2-{4-[6-(2,5-dimethoxyphenyl)pyridin-3-yl]-1,2,3-triazol-1-yl}ethyl)-N,N-dimethylbenzenesulfonamide (X21545):

To a solution of 4-(2-azidoethyl)-*N,N*-dimethylbenzenesulfonamide (**4**, 100 mg, 0.39 mmol, 1.00 equiv) in DMA (2 mL)/H_2O (2 mL) was added 2-(2,5-dimethoxyphenyl)-5-ethynylpyridine (**5**, 113 mg, 0.47 mmol, 1.20 equiv), $CuSO_4{\cdot}5H_2O$ (295 mg, 1.17 mmol, 3.00 equiv) and VcNa (234 mg, 1.17 mmol, 3.00 equiv). The reaction mixture was stirred at room temperature for 1 h. The resulted solution was purified using prep-HPLC with the following conditions (Column: XBridge Prep OBD C18 Column, 30*150 mm, 5 μm; Mobile Phase A: Water (10 mmol/L NH_4HCO_3), Mobile Phase B: ACN; Flow rate: 60 mL/min; Gradient: 30% B to 60% B in 8 min; Wave Length: 220 nm; RT: 7.50 min). This resulted in 4-(2-{4-[6-(2,5-dimethoxyphenyl)pyridin-3-yl]-1,2,3-triazol-1-yl}ethyl)-*N,N*-dimethylbenzenesulfonamide (**X21545**, 24.8 mg, 11.4% for 2 steps) as an off-white solid. $[M+H]^+$ = 494

^{1}H NMR (400 MHz, DMSO-d_6, 20°C): δ 2.59 (6H, s), 3.36 (2H, t), 3.73-3.80 (6H, m), 4.77 (2H, t), 6.99 (1H, d), 7.00-7.12 (1H, m), 7.38 (1H, d), 7.50 (2H, d), 7.66 (2H, d), 7.97 (1H, d), 8.16-8.19 (1H, m), 8.64 (1H, s), 9.07 (1H, s).

Synthesis of X20403

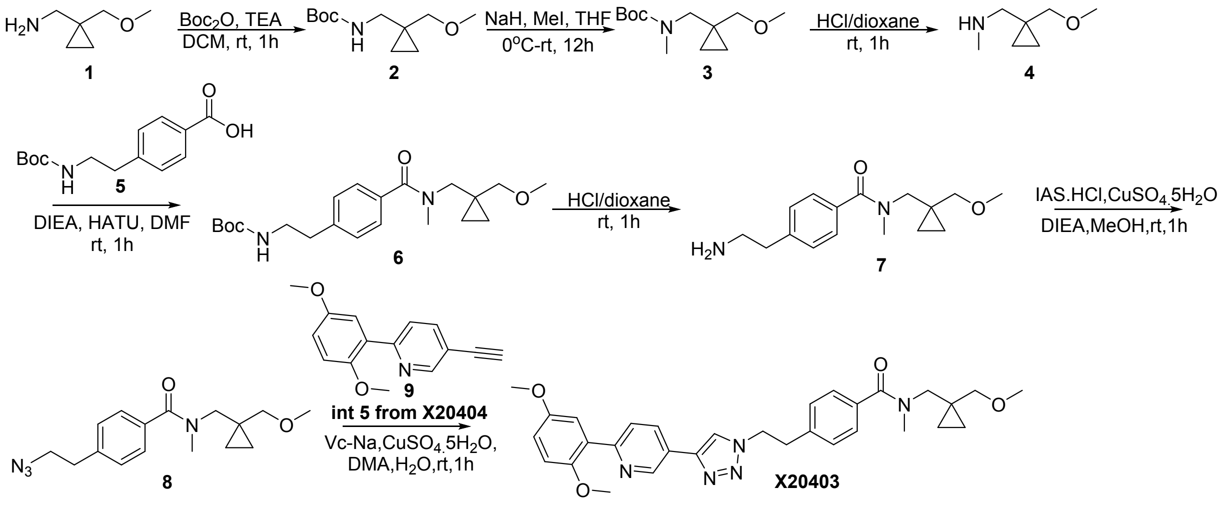

Synthesis of tert-butyl N-[[1-(methoxymethyl)cyclopropyl]methyl]carbamate (2):

To a stirred mixture of 1-[1-(methoxymethyl)cyclopropyl]methanamine (**1**, 400 mg, 3.47 mmol, 1.00 equiv) and TEA (703 mg, 6.95 mmol, 2.00 equiv) in DCM (4 mL) were added di-tert-butyl dicarbonate (834 mg, 3.82 mmol, 1.10 equiv). The resulting mixture was stirred at room temperature for 1 h. The resulting mixture was diluted with water and extracted with CH_2Cl_2, the combined organic layers were washed with brine, dried over anhydrous Na_2SO_4. After filtration, the filtrate was concentrated under reduced pressure to afford tert-butyl *N*-[[1-(methoxymethyl)cyclopropyl]methyl]carbamate (**2**, 750 mg, crude) as a light yellow oil. $[M+H]^+$ =216

Synthesis of tert-butyl N-[[1-(methoxymethyl)cyclopropyl]methyl]-N-methylcarbamate (3):

To a stirred mixture of tert-butyl *N*-[[1-(methoxymethyl)cyclopropyl]methyl]carbamate (**2**, 750 mg, 3.48 mmol, 1.00 equiv) in THF (10 mL) were added NaH (167 mg, 4.18 mmol, 1.20 equiv, 60%) in portions at 0°C. The resulting mixture was stirred at room temperature for 0.5 h under nitrogen atmosphere. Then were added MeI (544 mg, 3.83 mmol, 1.10 equiv), the resulting mixture was stirred at room temperature for 12 h. The reaction was quenched with sat. NH_4Cl (aq.), the resulting mixture was concentrated under reduced pressure. The residue was diluted with water, extracted with CH_2Cl_2, dried over anhydrous Na_2SO_4. After filtration, the filtrate was concentrated under reduced pressure to afford tert-butyl *N*-[[1-(methoxymethyl)cyclopropyl]methyl]-*N*-methylcarbamate (**3**, 800 mg, crude) as a light yellow oil.$[M+H]^+$ =230

Synthesis of [[1-(methoxymethyl)cyclopropyl]methyl](methyl)amine (4):

A solution of tert-butyl *N*-[[1-(methoxymethyl)cyclopropyl]methyl]-*N*-methylcarbamate (**3**, 800 mg, 3.49 mmol, 1.00 equiv) in HCl (4M in 1,4-dioxane, 10 mL). The resulting mixture was stirred at room temperature for 1 h. The resulting mixture was concentrated under reduced pressure to afford [[1-(methoxymethyl)cyclopropyl]methyl](methyl)amine (**4**, 450 mg, crude) as a yellow oil. The crude product was used in the next step directly without further purification. $[M+H]^+$ =130

Synthesis of tert-butyl N-[2-[4-([[1-(methoxymethyl)cyclopropyl]methyl](methyl)carbamoyl)phenyl]ethyl]carbamate (6):

To a stirred mixture of [[1-(methoxymethyl)cyclopropyl]methyl](methyl)amine (**4**, 450 mg, 3.48 mmol, 1.00 equiv) and 4-[2-[(tert-butoxycarbonyl)amino]ethyl]benzoic acid (**5**, 920 mg, 3.48 mmol, 1.00 equiv) in DMF (5 mL) were added DIEA (1.35 g, 10 mmol, 3.00 equiv) and HATU (1.59 g, 4.18 mmol, 1.20 equiv). The resulting mixture was stirred at room temperature for 1 h. The reaction mixture was purified using flash chromatography with the following conditions (Mobile Phase A: Water, Mobile Phase B: ACN; Flow rate: 60 mL/min; Gradient: 0% B to 80% B in 50 min; 254/220 nm) to afford tert-butyl *N*-[2-[4-([[1-(methoxymethyl)cyclopropyl]methyl](methyl)carbamoyl)phenyl]ethyl]carbamate (**6**, 1.00 g, 76.3%) as a light yellow oil. $[M+H]^+$ =377

Synthesis of 4-(2-aminoethyl)-N-[[1-(methoxymethyl)cyclopropyl]methyl]-N-methylbenzamide (7):

A solution of tert-butyl N-[2-[4-([[1-(methoxymethyl)cyclopropyl]methyl](methyl)carbamoyl)phenyl]ethyl]carbamate (**6**, 500 mg, 1.33 mmol, 1.00 equiv) in HCl (4M in 1,4-dioxane, 5 mL) was stirred at room temperature for 1 h, the resulting mixture was concentrated under reduced pressure. To afford 4-(2-aminoethyl)-*N*-[[1-(methoxymethyl)cyclopropyl]methyl]-*N*-methylbenzamide (**7**, 350 mg, crude) as a light yellow oil. The crude product was used in the next step directly without further purification. $[M+H]^+$ =277

Synthesis of 4-(2-azidoethyl)-N-[[1-(methoxymethyl)cyclopropyl]methyl]-N-methylbenzamide (8):

To a stirred mixture of 4-(2-aminoethyl)-N-[[1-(methoxymethyl)cyclopropyl]methyl]-*N*-methylbenzamide (**7**, 350 mg, 1.27 mmol, 1.00 equiv) and $CuSO_4.5H_2O$ (158 mg, 0.63 mmol, 0.50 equiv) in MeOH (5 mL) were added DIEA (327 mg, 2.53 mmol, 2 equiv) and ISA.HCl (529 mg, 2.53 mmol, 2 equiv). The resulting mixture was stirred at room temperature for 1 h. The resulting mixture was concentrated under reduced pressure. The residue was diluted with water and extracted with EtOAc. The combined organic layers were washed with brine, dried over anhydrous Na_2SO_4. After filtration, the filtrate was concentrated under reduced pressure to afford 4-(2-azidoethyl)-*N*-[[1-(methoxymethyl)cyclopropyl]methyl]-*N*-methylbenzamide (**8**, 300 mg, 78.3%) as a light yellow oil. $[M+H]^+$ =303

Synthesis of 4-(2-[4-[6-(2,5-dimethoxyphenyl)pyridin-3-yl]-1,2,3-triazol-1-yl]ethyl)-N-[[1-(methoxymethyl)cyclopropyl]methyl]-N-methylbenzamide (X20403):

To a stirred mixture of 4-(2-azidoethyl)-*N*-[[1-(methoxymethyl)cyclopropyl]methyl]-*N*-methylbenzamide (**8**, 200 mg, 0.66 mmol, 1.00 equiv) and 2-(2,5-dimethoxyphenyl)-5-ethynylpyridine (**9**, 158 mg, 0.66 mmol, 1.00 equiv) in DMA (2 mL) and H_2O (2 mL) were added Vc-Na (393 mg, 1.98 mmol, 3.00 equiv) and $CuSO_4.5H_2O$ (495 mg, 1.98 mmol, 3.00 equiv). The resulting mixture was stirred at room temperature for 1 h. The resulting mixture was purified using Prep-HPLC with the following conditions (Column: XBridge Prep OBD C18 Column, 30×150 mm 5 um; Mobile Phase A: Water (10 mmol/L NH_4HCO_3 +0.1% $NH_3.H_2O$), Mobile Phase B: ACN; Flow rate: 60 mL/min; Gradient: 30% B to 60% B in 8 min; 220 nm; RT: 7.23 min) to afford 4-(2-[4-[6-(2,5-dimethoxyphenyl)pyridin-3-yl]-1,2,3-triazol-1-yl]ethyl)-*N*-[[1-(methoxymethyl)cyclopropyl]methyl]-*N*-methylbenzamide (**X20403**, 68.0 mg, 19.0%) as a light yellow oil. $[M+H]^+$ =542

^{1}H NMR (DMSO-d_6, 400 MHz) δ 0.21 – 0.64 (4H, m), 2.96 (5H, t), 3.15 – 3.31 (6H, m), 3.46 (1H, s), 3.77 (3H, s), 3.81 (3H, s), 4.74 (2H, t), 7.00 (1H, dd), 7.11 (1H, d), 7.28 (4H, s), 7.40 (1H, d), 7.98 (1H, dd), 8.18 (1H, dd), 8.63 (1H, d), 9.07 (1H, s)

Synthesis of X21546

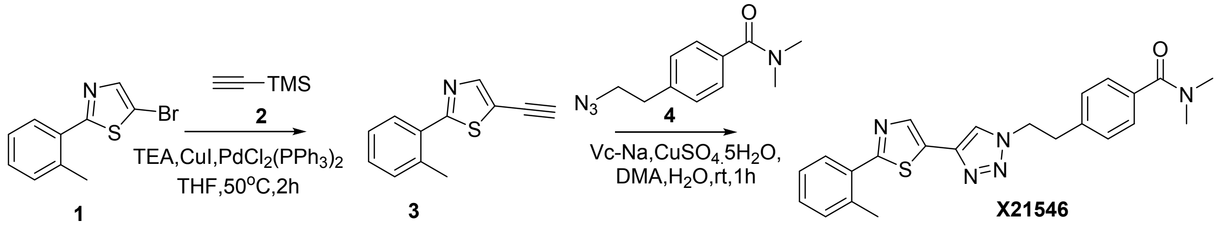

Synthesis of 5-ethynyl-2-(2-methylphenyl)-1,3-thiazole (3):

To a stirred mixture of 5-bromo-2-(2-methylphenyl)-1,3-thiazole (**1**, 400 mg, 1.57 mmol, 1.00 equiv) and TEA (478 mg, 4.72 mmol, 3.00 equiv) in THF (4 mL) were added trimethylsilylacetylene (**2**, 155 mg, 1.57 mmol, 1.00 equiv) and CuI (150 mg, 0.78 mmol, 0.50 equiv) and $Pd(PPh_3)_2Cl_2$ (110 mg, 0.15 mmol, 0.10 equiv) at room temperature under nitrogen atmosphere. The resulting mixture was stirred at 50^oC for 2 h under nitrogen atmosphere. The resulting mixture was extracted with EtOAc. The combined organic layers were washed with brine, dried over anhydrous Na_2SO_4. After filtration, the filtrate was concentrated under reduced pressure. The residue was purified by silica gel column chromatography, eluted with PE / EA (40:1) to afford 5-ethynyl-2-(2-methylphenyl)-1,3-thiazole (**3**, 350 mg, crude) as a light yellow solid. $[M+H]^+ = 200$

Synthesis of N,N-dimethyl-4-(2-{4-[2-(2-methylphenyl)-1,3-thiazol-5-yl]-1,2,3-triazol-1-yl}ethyl)benzamide (X21546):

To a stirred mixture of 5-ethynyl-2-(2-methylphenyl)-1,3-thiazole (**3**, 250.00 mg, 1.25 mmol, 1.00 equiv) and 4-(2-azidoethyl)-*N,N*-dimethylbenzamide (**4**, 274 mg, 1.25 mmol, 1.00 equiv) in DMA (1.5 mL) and H_2O (1.5 mL) were added $CuSO_4.5H_2O$ (940 mg, 3.76 mmol, 3.00 equiv) and sodium ascorbate (500 mg, 2.51 mmol, 2.00 equiv) at room temperature. The resulting mixture was stirred at room temperature for 1 h. The crude was purified using Prep-HPLC with the following conditions (Column: YMC-Actus Triart C18 ExRS, 30*150 mm, 5 µm; Mobile Phase A: water (10 mmol/L NH_4HCO_3), Mobile Phase B: ACN; Flow rate: 60 mL/min; Gradient: 30% B to 60% B in 8 min, 60% B; Wave Length: 254/220 nm; Rt: 7.35 min) to afford *N,N*-dimethyl-4-(2-{4-[2-(2-methylphenyl)-1,3-thiazol-5-yl]-1,2,3-triazol-1-yl}ethyl)benzamide (**X21546**, 18.5 mg, 3.5%) as a white solid.

$[M+H]^+$ = 418

^{1}H NMR (400 MHz, CD_3OD, 20°C): δ 2.56 (3H, s), 2.98 (3H, s), 3.09 (3H, d), 3.33-3.38 (2H, m), 4.78 (2H, t), 7.30 (2H, t), 7.32-7.42 (5H, m), 7.69 (1H, d), 8.13 (1H, s), 8.24 (1H, s).

Synthesis of X21478

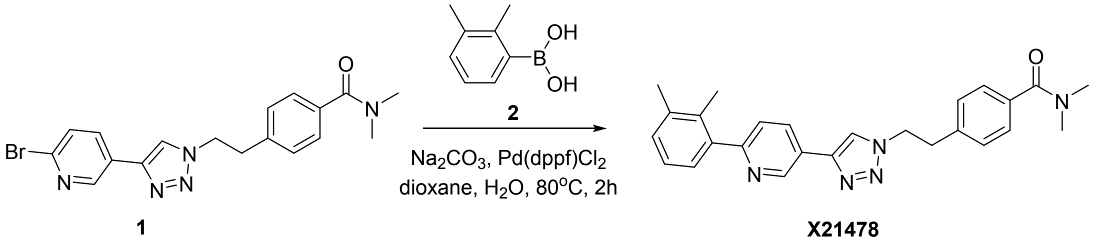

Synthesis of 4-(2-{4-[6-(2,3-dimethylphenyl)pyridin-3-yl]-1,2,3-triazol-1-yl}ethyl)-N,N-dimethylbenzamide (X21478):

To a stirred solution of 4-{2-[4-(6-bromopyridin-3-yl)-1,2,3-triazol-1-yl]ethyl}-*N,N*-dimethylbenzamide (**1**, 100 mg, 0.25 mmol, 1.00 equiv) and 2,3-dimethylphenylboronic acid (**2**, 37.5 mg, 0.25 mmol, 1.00 equiv) in 1,4-dioxane (0.60 mL) and H_2O (0.2 mL) were added Na_2CO_3 (79 mg, 0.75 mmol, 3 equiv) and Pd(dppf)$Cl_2 \cdot CH_2Cl_2$ (20.4 mg, 0.02 mmol, 0.10 equiv). The resulting mixture was stirred at 80°C for 2 h under nitrogen atmosphere. The resulting mixture was concentrated under reduced pressure. The residue was dissolved in DMF (1 mL). The mixture was purified using Prep-HPLC with the following conditions (Column: XBridge Prep OBD C18 Column, 30*150 mm, 5 μm; Mobile Phase A: water (10 mmol/L NH_4HCO_3 + 0.1% $NH_3 \cdot H_2O$), Mobile Phase B: ACN; Flow rate: 60 mL/min; Gradient: 4% B to 30% B in 8 min, 30% B; Wave Length: 220 nm; RT: 7.23 min) to afford 4-(2-{4-[6-(2,3-dimethylphenyl)pyridin-3-yl]-1,2,3-triazol-1-yl}ethyl)-*N,N*-dimethylbenzamide (**X21478**, 20.4 mg, 19.1%) as a white solid. $[M+H]^+$ = 426

^{1}H NMR (400 MHz, DMSO-d_6, 20°C): δ 2.19 (3H, s), 2.32 (3H, s), 2.87-2.95 (6H, m), 3.26-3.32 (2H, m), 4.73 (2H, t), 7.16-7.25 (3H, m), 7.27-7.33 (4H, m), 7.53 (1H, t), 8.22-8.24 (1H, m), 8.68 (1H, s), 9.07 (1H, d).

Synthesis of X21435

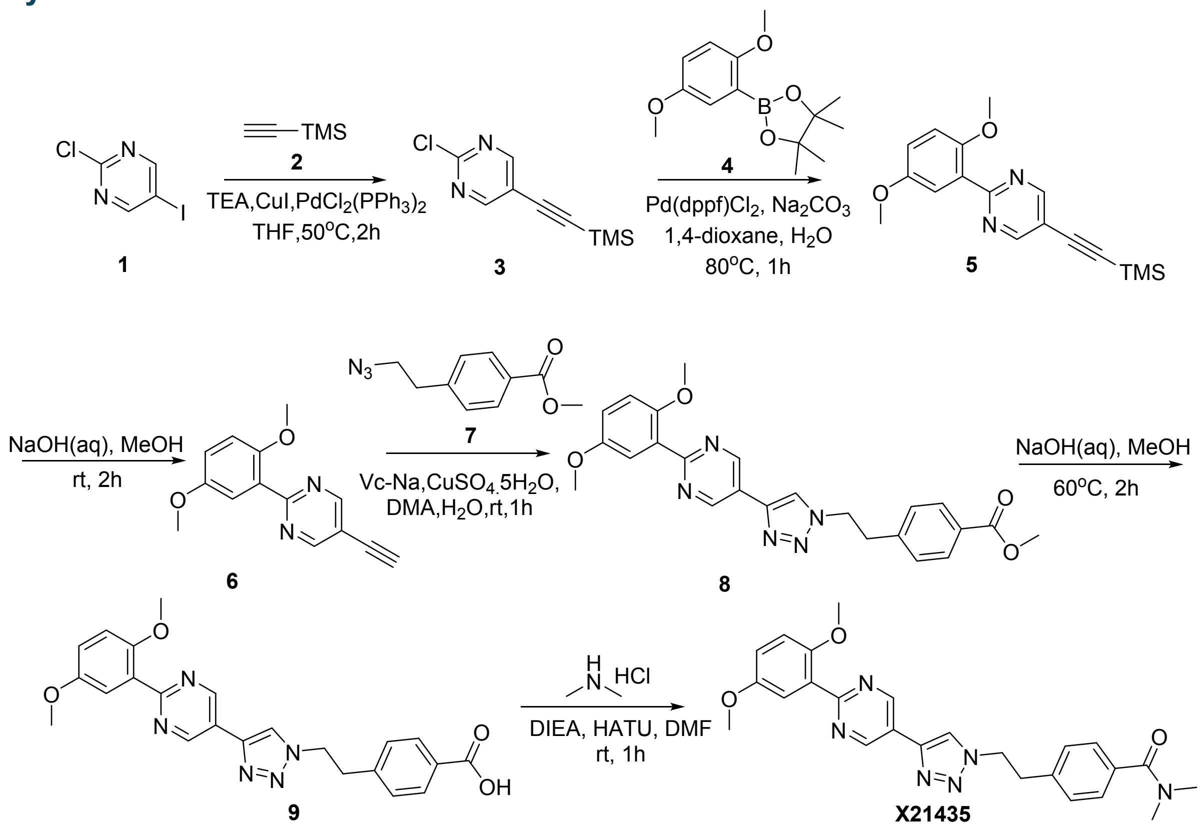

Synthesis of 2-chloro-5-[2-(trimethylsilyl)ethynyl]pyrimidine (3):

To a stirred solution of 2-chloro-5-iodopyrimidine (**1**, 500 mg, 2.08 mmol, 1.00 equiv) and trimethylsilylacetylene (**2**, 409 mg, 4.16 mmol, 2.00 equiv) in THF (10 mL) were added TEA (842 mg, 8.32 mmol, 4 equiv), CuI (396. mg, 2.08 mmol, 1.00 equiv) and $PdCl_2(PPh_3)_2$ (146 mg, 0.20 mmol, 0.10 equiv) at room temperature under nitrogen atmosphere. The resulting mixture was stirred at 50°C for 2 h under nitrogen atmosphere. Solvent was removed under vacuum. The residue was purified using C18 chromatography with the following conditions (Mobile Phase A: water, Mobile Phase B: ACN; Flow rate: 60 mL/min; Gradient: 0% B to 60% B in 30 min; 254/220 nm) to afford 2-chloro-5-[2-(trimethylsilyl)ethynyl]pyrimidine (**3**, 410 mg, 93.3%) as a brown solid. $[M+H]^+ = 211$

Synthesis of 2-(2,5-dimethoxyphenyl)-5-[2-(trimethylsilyl)ethynyl]pyrimidine (5):

To a stirred solution of 2-chloro-5-[2-(trimethylsilyl)ethynyl]pyrimidine (**3**, 400 mg, 1.89 mmol, 1.00 equiv) and 2-(2,5-dimethoxyphenyl)-4,4,5,5-tetramethyl-1,3,2-dioxaborolane (**4**, 501.34

mg, 1.89 mmol, 1.00 equiv) in 1,4-dioxane (3 mL) and H_2O (1 mL) were added Na_2CO_3 (402 mg, 3.79 mmol, 2.00 equiv) and Pd(dppf)Cl_2 (139 mg, 0.10 mmol, 0.10 equiv). The mixture was stirred at 80°C for 1 h under nitrogen atmosphere. The resulting mixture was concentrated under reduced pressure. The residue was purified using C18 chromatography with the following conditions (Mobile Phase A: water, Mobile Phase B: ACN; Flow rate: 60 mL/min; Gradient: 0% B to 80% B in 30 min; 254/220 nm) to afford 2-(2,5-dimethoxyphenyl)-5-[2-(trimethylsilyl)ethynyl]pyrimidine (**5**, 320 mg, 53.9%) as a yellow solid. $[M+H]^+$ = 313

Synthesis of 2-(2,5-dimethoxyphenyl)-5-ethynylpyrimidine (7):

To a stirred solution of 2-(2,5-dimethoxyphenyl)-5-[2-(trimethylsilyl)ethynyl]pyrimidine (**5**, 340 mg, 1.08 mmol, 1.00 equiv) in MeOH (2 mL) was added aq. NaOH (2M, 1 mL). The mixture was stirred at room temperature for 2 h. The mixture was acidified to pH 4 with HCl (aq.). The mixture was extracted with EtOAc. The combined organic layers were washed with brine, dried over anhydrous Na_2SO_4. After filtration, the filtrate was concentrated under reduced pressure. This resulted in 2-(2,5-dimethoxyphenyl)-5-ethynylpyrimidine (**6**, 260 mg, crude) as a brown oil. $[M+H]^+$ = 241

Synthesis of methyl 4-(2-{4-[2-(2,5-dimethoxyphenyl)pyrimidin-5-yl]-1,2,3-triazol-1-yl}ethyl)benzoate (8):

To a stirred mixture of 2-(2,5-dimethoxyphenyl)-5-ethynylpyrimidine (**6**, 240 mg, 0.99 mmol, 1.00 equiv) and methyl 4-(2-azidoethyl)benzoate (**7**, 205 mg, 0.99 mmol, 1.00 equiv) in DMA (2 mL) and H_2O (2 mL) were added $CuSO_4.5H_2O$ (748 mg, 3.00 mmol, 3.00 equiv) and VcNa (594 mg, 2.99 mmol, 3.00 equiv) at room temperature for 1h. The residue was purified by reverse flash chromatography with the following conditions: column, silica gel; mobile phase, MeCN in water, 10% to 50% gradient in 10 min; detector, UV 254 nm to afford methyl 4-(2-{4-[2-(2,5-dimethoxyphenyl)pyrimidin-5-yl]-1,2,3-triazol-1-yl}ethyl)benzoate (**8**, 260 mg, 58.4%) as a white solid. $[M+H]^+$ = 446

Synthesis of 4-(2-{4-[2-(2,5-dimethoxyphenyl)pyrimidin-5-yl]-1,2,3-triazol-1-yl}ethyl)benzoic acid (9):

To a stirred solution of methyl 4-(2-{4-[2-(2,5-dimethoxyphenyl)pyrimidin-5-yl]-1,2,3-triazol-1-

yl}ethyl)benzoate (**8**, 250 mg, 0.51 mmol, 1.00 equiv) in MeOH (2 mL) was added aq. NaOH (2M, 1. mL). The mixture was stirred at 60°C for 2 h. The mixture was acidified to pH 4 with HCl (aq.). The resulting mixture was concentrated under reduced pressure. The residue was purified using C18 chromatography with the following conditions (Mobile Phase A: water, Mobile Phase B: ACN; Flow rate: 60 mL/min; Gradient: 0% B to 60% B in 30 min; 254/220 nm) to afford 4-(2-{4-[2-(2,5-dimethoxyphenyl)pyrimidin-5-yl]-1,2,3-triazol-1-yl}ethyl)benzoic acid (**9**, 200 mg, 82.6%) as a yellow oil. $[M+H]^+ = 432$

Synthesis of 4-(2-{4-[2-(2,5-dimethoxyphenyl)pyrimidin-5-yl]-1,2,3-triazol-1-yl}ethyl)-N,N-dimethylbenzamide (X21435):

To a stirred solution of 4-(2-{4-[2-(2,5-dimethoxyphenyl)pyrimidin-5-yl]-1,2,3-triazol-1-yl}ethyl)benzoic acid (**9**, 190 mg, 0.44 mmol, 1.00 equiv) and dimethylamine hydrochloride (35.9 mg, 0.44 mmol, 1.00 equiv) in DMF (2 mL) were added HATU (201 mg, 0.52 mmol, 1.20 equiv) and DIEA (171 mg, 1.32 mmol, 3.00 equiv). The mixture was stirred at room temperature for 1 h. The mixture was purified using Prep-HPLC with the following conditions (Column: XB ridge Prep OBD C18 Column, 30 × 150 mm, 5 um; Mobile Phase A: water (10 mmol/L NH_4HCO_3 + 0.1%$NH_3 \cdot H_2O$), Mobile Phase B: ACN; Flow rate: 60 mL/min; Gradient: 20% B to 50% B in 8 min; 220 nm; RT: 7.23 min) to afford 4-(2-{4-[2-(2,5-dimethoxyphenyl)pyrimidin-5-yl]-1,2,3-triazol-1-yl}ethyl)-*N,N*-dimethylbenzamide (**X21435**, 20.3 mg, 10.1%) as a white solid. $[M+H]^+ = 459$

^{1}H NMR (400 MHz, DSMO-d6): δ 2.96 (6H, d), 3.26-3.32 (2H, m), 3.70-3.76 (6H, m), 4.76 (2H, t), 7.03-7.07 (1H, m), 7.10 (1H, d), 7.19 (1H, d), 7.27-7.33 (4H, m), 8.76 (1H, s), 9.27 (2H, s).

Synthesis of X21502

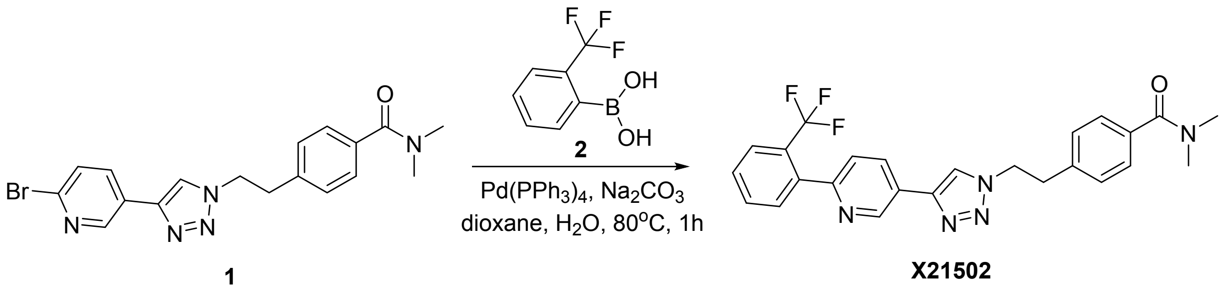

Synthesis of N,N-dimethyl-4-[2-(4-{6-[2-(trifluoromethyl)phenyl]pyridin-3-yl}-1,2,3-triazol-1-

yl)ethyl]benzamide (X21502):

To a stirred solution of 4-{2-[4-(6-bromopyridin-3-yl)-1,2,3-triazol-1-yl]ethyl}-*N,N*-dimethylbenzamide (**1**, 100 mg, 0.25 mmol, 1.00 equiv) and 2-(trifluoromethyl)phenylboronic acid (**2**, 47.5 mg, 0.25 mmol, 1.00 equiv) in 1,4-dioxane (0.9 mL) and H_2O (0.3 mL) were added Na_2CO_3 (53 mg, 0.50 mmol, 2.00 equiv) and $Pd(PPh_3)_4$ (17.5 mg, 0.02 mmol, 0.10 equiv). The mixture was stirred at 80°C for 1 h under nitrogen atmosphere. The resulting mixture was concentrated under reduced pressure. The crude was purified using Prep-HPLC with the following conditions (Column: YMC-Actus Triart C18 ExRS, 30*150 mm, 5 µm; Mobile Phase A: water (10 mmol/L NH_4HCO_3+0.1%$NH_3 \cdot H_2O$), Mobile Phase B: ACN; Flow rate: 60 mL/min; Gradient: 30% B to 52% B in 8 min, 52% B; Wave Length: 254/220 nm; RT: 7.55 min) to afford *N,N*-dimethyl-4-[2-(4-{6-[2-(trifluoromethyl)phenyl]pyridin-3-yl}-1,2,3-triazol-1-yl)ethyl]benzamide (**X21502,** 15.4 mg, 13.2%) as a white solid. $[M+H]^+$ = 466

^1H NMR (400 MHz, DMSO-d_6, 20°C): δ 2.95 (6H, d), 3.28-3.30 (2H, m), 4.74 (2H, s), 7.30 (4H, t), 7.59-7.87 (5H, m), 8.27 (1H, s), 8.71 (1H, s), 9.07 (1H, s).

Synthesis of X21558

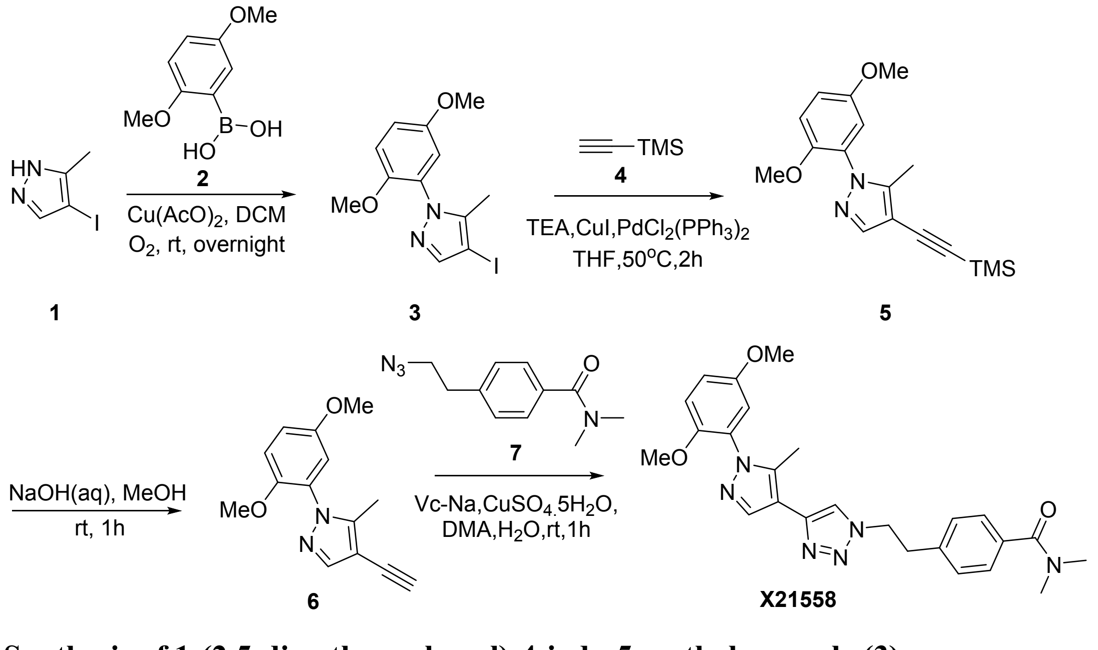

Synthesis of 1-(2,5-dimethoxyphenyl)-4-iodo-5-methylpyrazole (3):

To a stirred mixture of 4-iodo-3-methyl-2H-pyrazole (**1**, 1.00 g, 4.80 mmol, 1.00 equiv) and 2,5-dimethoxyphenylboronic acid (**2**, 1.75 g, 9.61 mmol, 2.00 equiv) in DCM (10.00 mL) was added

$Cu(AcO)_2$ (440 mg, 2.40 mmol, 0.50 equiv). The resulting mixture was stirred overnight at room temperature under Oxygen. The mixture was purified using C18 chromatography with the following conditions (Mobile Phase A: water, Mobile Phase B: ACN; Flow rate: 60 mL/min; Gradient: 0% B to 60% B in 30 min; 254/220 nm) to afford 1-(2,5-dimethoxyphenyl)-4-iodo-5-methylpyrazole (**3**, 1.70 g, crude) as a light yellow oil. $[M+H-56]^+$ = 345

Synthesis of 1-(2,5-dimethoxyphenyl)-5-methyl-4-[2-(trimethylsilyl)ethynyl]pyrazole (5):

To a stirred mixture of 1-(2,5-dimethoxyphenyl)-4-iodo-5-methylpyrazole (**3**, 1.00 g, 2.90 mmol, 1.00 equiv) and trimethylsilylacetylene (**4**, 570.79 mg, 5.81 mmol, 2.00 equiv) in THF (10.00 mL) were added TEA (882.08 mg, 8.71 mmol, 3.00 equiv) and CuI (277 mg, 1.45 mmol, 0.50 equiv) and $Pd(PPh_3)_2Cl_2$ (204 mg, 0.29 mmol, 0.10 equiv) at room temperature under nitrogen atmosphere. The resulting mixture was stirred at 50°C for 2 h under nitrogen atmosphere. The resulting mixture was concentrated under reduced pressure. The residue was dissolved in MeOH. The mixture was purified using C18 chromatography with the following conditions (Mobile Phase A: water, Mobile Phase B: ACN; Flow rate: 60 mL/min; Gradient: 0% B to 60% B in 30 min; 254/220 nm) to afford 1-(2,5-dimethoxyphenyl)-5-methyl-4-[2-(trimethylsilyl)ethynyl]pyrazole (**5**, 200 mg, 21.8%) as a brown solid. $[M+H]^+$ = 315

Synthesis of 1-(2,5-dimethoxyphenyl)-4-ethynyl-5-methylpyrazole (6):

To a stirred solution of 1-(2,5-dimethoxyphenyl)-5-methyl-4-[2-(trimethylsilyl)ethynyl]pyrazole (**5**, 190 mg, 0.60 mmol, 1.00 equiv) in MeOH (1 mL) was added NaOH (2M, 1 mL) at room temperature. The resulting mixture was stirred at room temperature for 1 h. The resulting mixture was diluted with water. The resulting mixture was extracted with EtOAc. The combined organic layers were washed with brine, dried over anhydrous Na_2SO_4. After filtration, the filtrate was concentrated under reduced pressure. This resulted in 1-(2,5-dimethoxyphenyl)-4-ethynyl-5-methylpyrazole (**6**, 140 mg, 95.6%) as a light yellow solid. $[M+H]^+$ = 243

Synthesis of 4-(2-{4-[1-(2,5-dimethoxyphenyl)-5-methylpyrazol-4-yl]-1,2,3-triazol-1-yl}ethyl)-N,N-dimethylbenzamide (X21558):

To a stirred mixture of 1-(2,5-dimethoxyphenyl)-4-ethynyl-5-methylpyrazole (**6**, 130 mg, 0.53 mmol, 1.00 equiv) and 4-(2-azidoethyl)-N,N-dimethylbenzamide (**7**, 117 mg, 0.53 mmol, 1.00

equiv) in DMA (1 mL) and H_2O (1 mL) were added $CuSO_4.5H_2O$ (402 mg, 1.61 mmol, 3.00 equiv) and sodium ascorbate (321 mg, 1.61 mmol, 3.00 equiv) at room temperature. The resulting mixture was stirred at room temperature for 1 h. The residue was purified using Prep-HPLC with the following conditions (Column: XSelect CSH Prep C18 OBD Column, 19*250 mm, 5 µm; Mobile Phase A: water (10 mmol/L NH_4HCO_3), Mobile Phase B: ACN; Flow rate: 60 mL/min; Gradient: 24% B to 48% B in 8 min, 48% B; Wave Length: 254; 220 nm; RT: 7.48 min) to afford 4-(2-{4-[1-(2,5-dimethoxyphenyl)-5-methylpyrazol-4-yl]-1,2,3-triazol-1-yl}ethyl)-*N,N*-dimethylbenzamide (**X21558**, 22.3 mg, 9.0%) as a light yellow solid. $[M+H]^+$ = 461

^{1}H NMR (400 MHz, CD_3OD, 20°C): δ 2.42 (3H, s), 2.97 (3H, s), 3.09 (3H, s), 3.32-3.37 (2H, m), 3.82-3.87 (6H, m), 4.74-4.88 (2H, m), 6.92-6.95 (1H, m), 7.16 (1H, d), 7.25-7.30 (3H, m), 7.30-7.37 (2H, m), 7.97 (1H, s), 8.40 (1H, s).

Synthesis of X21497

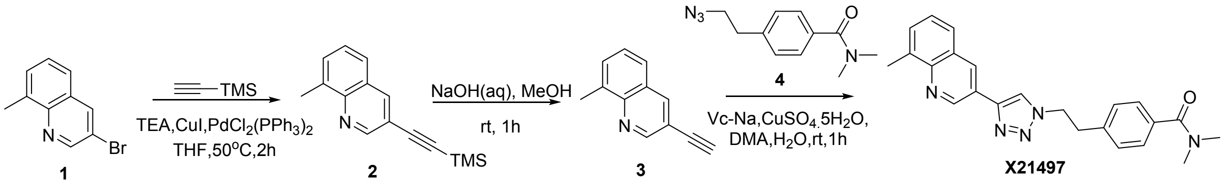

Synthesis of 8-methyl-3-[2-(trimethylsilyl)ethynyl]quinoline (2):

To a stirred mixture of 3-bromo-8-methylquinoline (**1**, 400 mg, 1.80 mmol, 1.00 equiv) and TEA (365 mg, 3.60 mmol, 2.00 equiv) in THF (4 mL) were added trimethylsilylacetylene (212 mg, 2.16 mmol, 1.20 equiv) and CuI (172 mg, 0.90 mmol, 0.50 equiv) and $Pd(PPh_3)_2Cl_2$ (126 mg, 0.18 mmol, 0.10 equiv) at room temperature under nitrogen atmosphere. The resulting mixture was stirred at 50°C for 2 h under nitrogen atmosphere. The resulting mixture was extracted with EtOAc. The combined organic layers were washed with brine, dried over anhydrous Na_2SO_4. After filtration, the filtrate was concentrated under reduced pressure. The residue was purified by silica gel column chromatography, eluted with PE / EA (40:1) to afford 8-methyl-3-[2-(trimethylsilyl)ethynyl]quinoline (**2**, 210 mg, 48.7%) as a light yellow oil. $[M+H]^+$ = 240

Synthesis of 3-ethynyl-8-methylquinoline (3):

To a stirred solution of 8-methyl-3-[2-(trimethylsilyl)ethynyl]quinoline (**2**, 200 mg, 0.83 mmol,

1.00 equiv) in MeOH (1.5 mL) was added NaOH (2M) (0.5 mL). The mixture was stirred at room temperature for 1 h. The mixture was purified using C18 chromatography with the following conditions (Mobile Phase A: water, Mobile Phase B: ACN; Flow rate: 60 mL/min; Gradient: 0% B to 60% B in 30 min; 254/220 nm) to afford 3-ethynyl-8-methylquinoline (**3**, 130 mg, 93.06%) as a brown solid. $[M+H]^+$ = 168

Synthesis of N,N-dimethyl-4-{2-[4-(8-methylquinolin-3-yl)-1,2,3-triazol-1-yl]ethyl}benzamide (X21497):

To a stirred mixture of 3-ethynyl-8-methylquinoline (**3**, 120.00 mg, 0.71 mmol, 1.00 equiv) and 4-(2-azidoethyl)-*N,N*-dimethylbenzamide (**4**, 157 mg, 0.71 mmol, 1.00 equiv) in DMA (1 mL) and H_2O (1 mL) were added $CuSO_4.5H_2O$ (538 mg, 2.15 mmol, 3.00 equiv) and sodium ascorbate (429 mg, 2.15 mmol, 3.00 equiv) at room temperature. The resulting mixture was stirred at room temperature for 1 h. The mixture was purified using flash chromatography with the following conditions (Mobile Phase A: Water, Mobile Phase B: ACN; Flow rate: 60 mL/min; Gradient: 0% B to 65% B in 50 min; 254/220 nm). This resulted in *N,N*-dimethyl-4-{2-[4-(8-methylquinolin-3-yl)-1,2,3-triazol-1-yl]ethyl}benzamide (**X21497**, 24.7 mg, 8.9%) as a light yellow solid. $[M+H]^+$ = 386

^{1}H NMR (400 MHz, DMSO-d6): δ 2.74 (3H, t), 2.95 (6H, t), 3.28-3.34 (2H, m), 4.75 (2H, t), 7.29-7.34 (4H, m), 7.52 (1H, t), 7.60 (1H, d), 7.86 (1H, d), 8.70 (1H, d), 8.76 (1H, s), 9.35 (1H, d).
